# Regional burden of chronic kidney disease in North Africa and Middle East during 1990–2019; Results from Global Burden of Disease study 2019

**DOI:** 10.3389/fpubh.2022.1015902

**Published:** 2022-10-11

**Authors:** Ozra Tabatabaei-Malazy, Sahar Saeedi Moghaddam, Patricia Khashayar, Mohammad Keykhaei, Yeganeh Sharifnejad Tehrani, Mohammad-Reza Malekpour, Zahra Esfahani, Mohammad-Mahdi Rashidi, Ali Golestani, Parnian Shobeiri, Mana Moghimi, Fateme Gorgani, Elham Abdolhamidi, Farshad Farzadfar, Bagher Larijani

**Affiliations:** ^1^Non-Communicable Diseases Research Center, Endocrinology and Metabolism Population Sciences Institute, Tehran University of Medical Sciences, Tehran, Iran; ^2^Endocrinology and Metabolism Research Center, Endocrinology and Metabolism Clinical Sciences Institute, Tehran University of Medical Sciences, Tehran, Iran; ^3^Osteoporosis Research Center, Endocrinology and Metabolism Population Sciences Institute, Tehran University of Medical Sciences, Tehran, Iran; ^4^Center for Microsystems Technology, Imec and Ghent University, Gent, Belgium; ^5^Feinberg School of Medicine, Feinberg Cardiovascular Research Institute, Northwestern University, Chicago, IL, United States; ^6^Department of Biostatistics, University of Social Welfare and Rehabilitation Sciences, Tehran, Iran

**Keywords:** chronic kidney disease, Burden of Disease, diabetes, hypertension, body mass index

## Abstract

**Objectives:**

Updating burden data of chronic kidney disease (CKD) as one of the most prevalent non-communicable diseases is essential for proper provision of healthcare by policymakers. We aimed to estimate the burden of CKD and its attributed burden in North Africa and Middle East region (NAME) during 1990–2019.

**Methods:**

The CKD-related Global Burden of Disease (GBD) 2019 estimates were extracted from Health Metrics and Evaluation (IHME) website.

**Results:**

In 2019, 2,034,879 new CKD cases (95% Uncertainty interval 1,875,830 to 2,202,724) with an age-standardized incidence rate of 447.5 (415.1 to 482.8) per 100,000 was reported, showing a 70.9% increase in the past 30 years. CKD led to 111,812 deaths (96,421 to 130,853) with an age-standardized rate of 30.4 (26.3 to 35.4) per 100,000. The highest increase and decrease in the mortality rate were estimated in Morocco 21.8% (−8.9 to 51.6) and Kuwait −41.5% (−51.2 to −29.1). In 2019, CKD was responsible for 744.4 (646.1 to 851.8) age-standardized disability-adjusted life years (DALYs), mostly contributed to “other and unspecified causes” [237.2 (191.1 to 288.4)], type 2 diabetes [205.9 (162.4 to 253.6)], and hypertension [203.3 (165.8 to 243)]. An increase was noted in DALYs from ages 25–29 and surged with an accelerating pattern by age. Kidney dysfunction, high systolic blood pressure, and high body mass index ranked as the top three risk factors for the disorder.

**Conclusions:**

Our study raised an alarm regarding the increasing CKD burden in NAME. There is an urgency to deal with hypertension and overweight/obesity at the primary care level, implementing CKD screening for at-risk groups, and facilitating the accessibility to appropriate treatments.

## Introduction

Chronic kidney disease (CKD) is one of the leading non-communicable diseases (NCDs) associated with high morbidity and mortality, especially it is considered as a risk factor for cardiovascular diseases (CVDs) (1, 2). It is diagnosed by reduced estimated glomerular filtration rate to <60 mL/min/1.73 m^2^, albuminuria (at least 30 mg/24 h), or persistence increase in kidney damage markers for >three months (3). The estimated global prevalence of CKD is 10–16% of the general population, which increases by age (4, 5). CKD prevalence in low or middle-income countries is higher than that in high-income countries (6). Moreover, its global mortality rate and disability-adjusted life years (DALYs) were estimated to be at 1.2 and 35 million based on the Global Burden of Disease (GBD) study 2016, respectively (7).

So far, several risk factors such as diabetes, hypertension, and glomerulonephritis have been identified for the CKD development. The population growth and aging, accompanied by increasing prevalence of diabetes, hypertension and glomerulonephritis, have worsened the health status (5, 6, 8, 9). The increasing prevalence of CKD with age along with the longevity of older CKD patients has increased the chances of kidney failure. CKD imposes a heavy burden on the health systems, due to the need for intensive treatments for kidney failure (e.g., renal dialysis or kidney transplant) (10). Thereby, having access to specific health measures are crucial for policymakers to have a proper estimation of health status and the ability to compare the condition, especially assessing time trend between or within neighboring countries in the region. DALYs is the main summary measure of the health status in this regard (11, 12). Incidence, prevalence, mortality, years of life lost (YLLs), and years lived with disability (YLDs) due to CKD based on the country, age group, sex, and socio-demographic status are other valuable measures for the policymakers.

Although several studies have assessed the burden of CKD (7, 12, 13), lack of comprehensive and reliable published data regarding the condition in North Africa and Middle East (NAME) region is glaring. Hence, we aim to estimate the historical and prospective status of all CKD-related health measures as well as the attributed burden due to its risk factors in the countries of the NAME region during 1990–2019.

## Methods

Primary epidemiologic indices were retrieved from the GBD 1990–2019 database, which is publicly available on the Institute for Health Metrics and Evaluation (IHME) website; http://www.healthdata.org. The secondary indices, including incidence, prevalence, mortality, YLLs, YLDs, and DALYs, for the region that consists of 21 countries: Afghanistan, Algeria, Bahrain, Egypt, Iran (the Islamic Republic of), Iraq, Jordan, Kuwait, Lebanon, Libya, Morocco, Oman, Palestine, Qatar, Saudi Arabia, Sudan, Syrian Arab Republic, Tunisia, Turkey, United Arab Emirates, and Yemen, were also extracted. Details of GBD study 2019 and general process of the disease burden estimation on 369 diseases and injuries in 204 countries and territories are described elsewhere (12). Comparative risk assessment (CRA) methodology was used to estimate the attributed burden to risk factors (13) such as non-optimal temperature, lead exposure, following a diet high in sodium, high fasting plasma glucose, high body-mass index (BMI), high systolic blood pressure (SBP), and kidney dysfunction.

The International Classification of Diseases (ICD)-10 codes were applied to define CKD mortality and non-fatality cases (12, 14). The estimation of CKD-related GBD was based on the data from the death registration system, surveys, reports, disease registries, and scientific literature. The rates were expressed as age-standardized based on the GBD reference population. Uncertainty intervals (UIs) of 95% were calculated with the 2.5th and 97.5th percentiles of 1,000 drawn by age, sex, residency, and year.

Decomposition analysis was applied to determine the contribution of changes in the incidence rate, population growth, and population aging on the emergence of new cases in the same time interval. Percent changes were calculated as the difference between the burden calculated in 2019 and that in 1990 divided by the 1990 data. Overall, the age was reported as 5-year-based groups (except for <1 year, 1–4 years old, and 95 plus years) and was then categorized into five groups, including 0–9, 10–24, 25–49, 50–74, and 75 plus years old. All figures were depicted by R version 3.4.2.

## Results

CKD new cases in the region increased from 505,955 in 1990 to 2,034,879 cases in 2019. A 302.2% increase was estimated in new cases in both sexes, prominently among males (347.7%) vs. females (269.3%) (Table 1). Moreover, the highest increase was noted in Qatar 1,350.5% (1,723.7% in males, 865.2% in females), followed by the United Arab Emirates 1,219.4% (1,484.8% in males, 820.9% in females). Based on the decomposition analysis, except for Afghanistan, population growth, age structure and incidence rate changes had a positive effect on the overall changes noted in the number of new cases (Supplementary Table 1). From 1990 to 2019, the age-standardized incidence rate (ASIR) of CKD (per 100,000) increased from 261.9 to 447.5 for both sexes, showing a 70.9% surge (Table 1). All countries reported positive percent changes. The highest increase in ASIR for both sexes was 105.3%, reported during the same time period in Morocco. This change was 134.1% for males compared to 86.1% for females, with a ratio of 1.6. The lowest ASIR change in both sexes was reported in Iran (34.7%), 37.3% for males and 30.5% for females, with a ratio of 1.2 (Supplementary Table 2).

**Table 1:** All ages number and age-standardized rate of health measures of CKD based on sex during 1990–2019 in NAME region.

**Measure**	**Metric**	**Year**						**% Change (1990–2019)**		
		**1990**			**2019**			
		**Both**	**Female**	**Male**	**Both**	**Female**	**Male**	**Both**	**Female**	**Male**
Incidence	All ages number	505,955 (466,448–550,245)	293,801 (269,833–320,905)	212,154 (195,911–230,418)	2,034,879 (1,875,830–2,202,724)	1,085,077 (1,001,214–1,173,981)	949,802 (872,451–1,028,465)	302.2 (291.8–313)	269.3 (258.8–280.4)	347.7 (335.3–361.5)
	Age-standardized rate (per 100,000)	261.9 (241.0–285.9)	306.9 (281.7–337.7)	218.2 (200.8–237.0)	447.5 (415.1–482.8)	485.5 (449.6–523.4)	411.4 (379.1–445.6)	70.9 (66.9–75.0)	58.2 (53.7–63.1)	88.5 (83.9–93.6)
Prevalence	All ages number	15,774,468 (14,732,341–16,764,819)	8,883,786 (8,285,409–9,458,129)	6,890,682 (6,425,771–7,333,886)	49,799,389 (46,524,529–52,818,543)	27,021,113 (25,254,840–28,679,291)	22,778,276 (21,265,704–24,154,988)	215.7 (209.3–222.5)	204.2 (198–210.8)	230.6 (222.3–239.1)
	Age-standardized rate (per 100,000)	7,697.1 (7,182.7–8,168.8)	8,781.1 (8,183.7–9,346)	6,623.5 (6,189–7,040.7)	10,589.7 (9,930.4–11,229.5)	11,694 (10,966.6–12,387.6)	9,551.2 (8,943.6–10,160.7)	37.6 (35.0–40.3)	33.2 (30.6–36.0)	44.2 (40.8–47.9)
Deaths	All ages number	52,596 (47,638–61,837)	26,193 (23,483–32,521)	26,403 (23,592–31,174)	111,812 (96,421–130,853)	56,399 (46,447–65,192)	55,413 (47,430–68,977)	112.6 (75.7–152)	115.3 (71.4–152)	109.9 (69–167.1)
	Age-standardized rate (per 100,000)	34.7 (30.7–44.1)	33.6 (29.7–45.5)	36.2 (31.1–45.6)	30.4 (26.3–35.4)	30.9 (25.7–35.5)	29.9 (25.7–36.9)	−12.6 (−29.4–3.9)	−8.2 (−31.1–7.5)	−17.3 (−35.8–6.4)
DALYs	All ages number	1,767,039 (1,616,931–1,930,042)	917,432 (827,090–1,034,476)	849,607 (775,036–927,852)	3,381,323 (2,920,288–3,897,905)	1,719,368 (1,446,769–1,971,737)	1,661,955 (1,417,431–2,036,842)	91.4 (67.1–118.3)	87.4 (57.1–116.1)	95.6 (70.1–134.9)
	Age-standardized rate (per 100,000)	834.9 (762.3–947.2)	853.6 (771.8–1,006.8)	819.2 (737.4–939.5)	744.4 (646.1–851.8)	770.0 (653.6–879.5)	720.3 (622.1–872.5)	−10.8 (−23.9–2.4)	−9.8 (−25.3–3.3)	−12.1 (−27.1–7.5)
YLLs	All ages number	1,530,882 (1,392,713–1,677,162)	777,338 (696,320–884,579)	753,544 (683,435–825,732)	2,616,337 (2,211,230–3,108,096)	1,307,651 (1,072,931–1,534,513)	1,308,687 (1,107,296–1,646,563)	70.9 (46–101.4)	68.2 (36–100.4)	73.7 (46.7–115.8)
	Age-standardized rate (per 100,000)	731.4 (665.2 to 840.8)	730.5 (657 to 873.7)	735.1 (660.4–852.8)	590.3 (502.9–694.7)	602.8 (494.7–698.9)	578.4 (494.5–722.5)	−19.3 (−32.7– −4.3)	−17.5 (−34.2– −2.8)	−21.3 (−36.7– 0.2)
YLDs	All ages number	236,157 (172,238–310,169)	140,094 (102,121–183,926)	96,063 (69,130–126,698)	764,986 (559,360–1,004,981)	411,717 (301,269–533,770)	353,268 (250,414–474,091)	223.9 (203.6–246.2)	193.9 (176.3–213)	267.7 (242.1–293.4)
	Age-standardized rate (per 100,000)	103.5 (76.1–135.6)	123.1 (90.8–162.0)	84.1 (60.9–111.5)	154 (113.1–202.3)	167.3 (123.8–215.9)	141.9 (101.3–191.7)	48.8 (41.3–56.6)	35.8 (28.8–42.9)	68.8 (59.0–78.4)

Data in parentheses are 95% Uncertainty Intervals (95% UIs); DALYs, Disability-Adjusted Life Years; YLLs, Years of Life Lost; YLDs, Years Lived with Disability.

During 1990–2019, the age-standardized prevalence rate (ASPR) of CKD (per 100,000) increased from 7,697.1 to 10,589.7, with a 37.6% change for both sexes (Table 1). All countries reported positive percent changes. In addition, at the country level in the same interval, the highest increase in ASPR for both sexes was 51.7% in Morocco. This change was 61.0% for males compared to 45.7% for females, with a ratio of 1.3. The lowest ASPR change for both sexes was reported in Iran 19.6%; 20.7% for males and 18.6% for females with a ratio of 1.1 (Supplementary Table 2).

CKD mortality was 52,596 and 111,812 in 1990 and 2019, respectively. The age-standardized mortality rate (ASMR) due to CKD decreased by 12.6% from 1990 to 2019 (Table 1). The ASMR increased in Morocco (21.8%), Oman (18.0%), Egypt (11.5%), Saudi Arabia (10.4%), and Libya (2.1%) (Supplementary Table 2). The highest and lowest reductions in ASMR were noted in Kuwait (−41.5%) and Sudan (−0.5%), respectively. The lowest ASMR change ratio of males (−36.4%) to females (−47.4%) was 0.8 in Kuwait. However, the highest such ratio change was 1.7 in Sudan [males (−3.4%) to females (2.0%)] (Supplementary Table 2).

In 2019, the age-standardized rates of YLDs (ASYLDR), YLLs (ASYLLR), and DALYs (ASDR) of CKD were 154.0, 590.3, and 744.4 per 100,000, respectively. During 1990–2019, the regional ASYLDR increased by 48.8%, whereas the ASYLLR and the ASDR decreased by −19.3 and −10.8%, respectively (Table 1). The lowest and highest accelerations in the ASYLDR was 11.8% for Iran and 86.7% for Morocco. The lowest and highest reductions in the ASYLLR, on the other hand, were 2.1% for Libya and 49.1% for Kuwait, correspondingly. The highest and lowest reductions in the ASDR were 35.4% for Kuwait and 1.5% for Tunisia, respectively (Supplementary Table 2).

Down- and upward time trends were also observed in the studied health measures for each country between 1990 and 2019. As for the CKD DALYs, the largest contribution in terms of the age-standardized rate of any cause in 2019 was due to “other and unspecified causes” accounting for 237.2 rate per 100,000. This accounted for 197.6 and 278.4 ASDR per 100,000 in males and females, respectively. The CKD-related regional ASDR rate due to diabetes type 1, diabetes type 2, hypertension, and glomerulonephritis in 2019 was 34.0, 205.9, 203.3, and 64.0 per 100,000, respectively.

**Figure 1:**
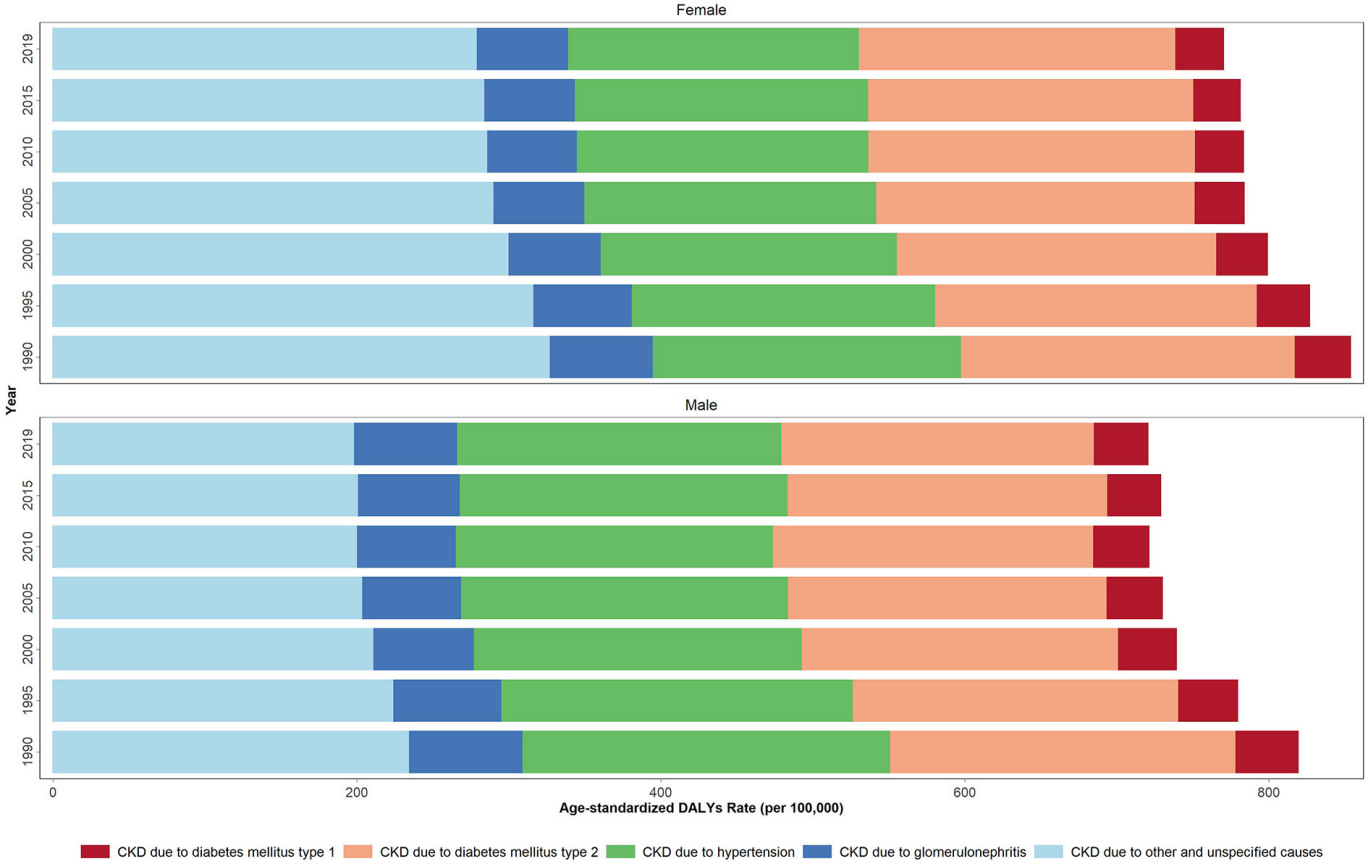
Age-standardized DALYs rate due to CKD by underlying causes in 1990, 1995, 2000, 2005, 2010 and 2019 in the NAME region.

Between 1990 and 2019, a reduction was noted in the ASDR for all underlying causes for CKD (Figure 1, Supplementary Table 3). The CKD-related ASPR due to all causes increased from 1990 to 2019 among both sexes. In the same time period, the acceleration in the ASPR rates due to diabetes type 2 and other causes was more significant among females; but among males for the cases secondary to glomerulonephritis. Accordingly, similar results were obtained for other studied health indicators (Supplementary Table 3, Supplementary Figures 1A–E).

**Figure 2:**
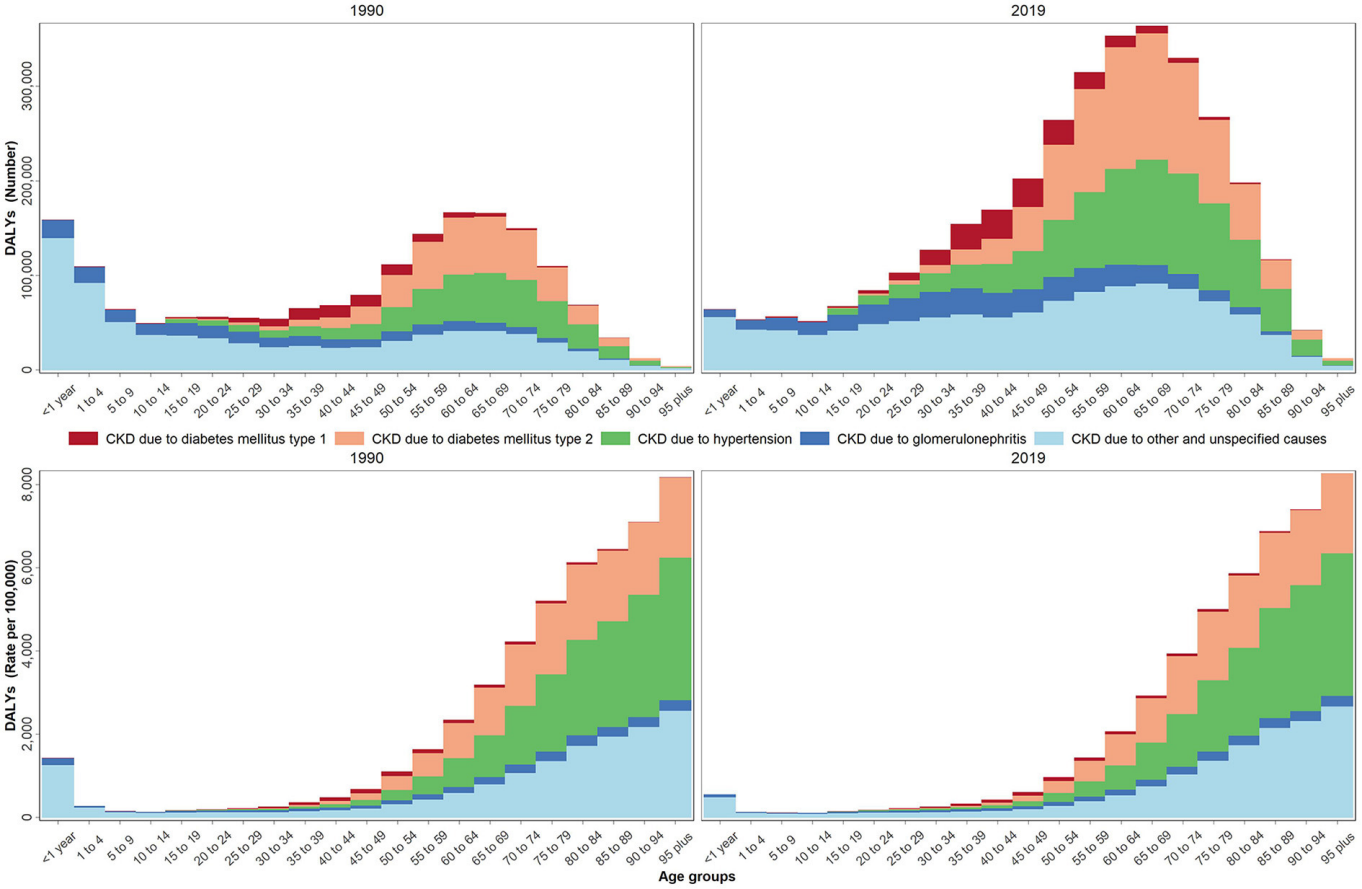
DALYs number and rate by age groups due to CKD by underlying causes in 1990 and 2019 in the NAME region.

Age distribution of all studied health indicators during 1990–2019 are shown in Supplementary Figures 2A–E. The age-based assessments showed an increase in the incidence rate starting at the 35–39 age group and reaching its highest rate at the 70–74 age group. A decrease, however, was noted at the 80^+^ age group (invert U shape), Supplementary Figure 2A. The increase reported in the prevalence rate, on the other hand, stared at the 25–29 age group and continued in an accelerating pattern, Supplementary Figure 2B. The mortality rate had a different pattern from the incidence and prevalence rates. The increase in the mortality rate started in the 50–54 age group and continued in an accelerating pattern by age, Supplementary Figure 2C. The DALYs rate had a pattern similar to the prevalence rate, Figure 2. Based on the aggregated age groups, the highest and lowest mortality numbers in 2019 were observed in the 50–74 (52014) and 0–9 age groups (1497), accordingly (Table 2, Supplementary Table 4).

**Table 2:** Health measures of CKD based on age groups and sex during 1990-2019 in NAME region.

**Measure**	**Metric**	**Age**	**Year**						**% Change (1990**–**2019)**		
			**1990**			**2019**			
			**Both**	**Female**	**Male**	**Both**	**Female**	**Male**	**Both**	**Female**	**Male**
Deaths	Number	0–9	3,524 (1,875–4,681)	1,900 (928–2,647)	1,624 (806–2,278)	1,497 (1,179–1,841)	703 (526–899)	794 (614–1,030)	−57.5 (−69.2–−20.3)	−63 (−75–−26.2)	−51.1 (−67.5–7.6)
		10–24	1,644 (1,462–1,837)	947 (818–1,080)	697 (617–799)	1,605 (1,320–1,964)	876 (701–1,091)	729 (574–984)	−2.4 (−19.5–21.2)	−7.6 (−29–17.5)	4.6 (−18–41.1)
		25–49	5,004 (4,499–5,536)	2,548 (2,243–2,911)	2,456 (2,166–2,776)	10,224 (8,302–12,475)	4,937 (3,764–6,051)	5,287 (4,291–6,844)	104.3 (71–146.4)	93.8 (50.4–139.3)	115.3 (74.6–176.7)
		50–74	25,431 (22,939–30,068)	12,263 (10,911–15,681)	13,169 (11,665–15,740)	52,014 (43,555–62,568)	26,048 (20,837–30,944)	25,967 (21,539–33,228)	104.5 (61.9–148.1)	112.4 (57.9–157.4)	97.2 (49.1–158.9)
		75 plus	16,993 (14,522–23,260)	8,535 (7,300–12,856)	8,457 (6,907–11,603)	46,471 (39,854–53,777)	23,836 (20,018–27,540)	22,635 (19,238–27,795)	173.5 (113.5–225.4)	179.3 (96.1–231.4)	167.6 (101.6–245.5)
	Rate	0–9	3.5 (1.8–4.6)	3.8 (1.9–5.4)	3.1 (1.6–4.4)	1.3 (1–1.5)	1.2 (0.9–1.6)	1.3 (1–1.7)	−63.9 (−73.9– −32.3)	−68.4 (−78.7– −37)	−58.6 (−72.5– −8.9)
		10–24	1.5 (1.3–1.7)	1.8 (1.5–2)	1.2 (1.1–1.4)	1 (0.8–1.2)	1.1 (0.9–1.4)	0.9 (0.7–1.2)	−33.2 (−44.9– −17)	−36.1 (−50.9– −18.8)	−29 (−44.3– −4.2)
		25–49	5.3 (4.8–5.9)	5.6 (4.9–6.4)	5.1 (4.5–5.7)	4.5 (3.6–5.4)	4.6 (3.5–5.6)	4.4 (3.6–5.7)	−15.8 (−29.6–1.5)	−17.9 (−36.3–1.3)	−13.5 (−29.8–11.2)
		50–74	72.7 (65.6–86)	72 (64–92)	73.5 (65.1–87.8)	59 (49.4–71)	60.9 (48.7–72.4)	57.3 (47.5–73.3)	−18.8 (−35.7– −1.5)	−15.4 (−37.1–2.6)	−22 (−41.1–2.4)
		75 plus	431.9 (369.1–591.2)	411.1 (351.6–619.2)	455.1 (371.7–624.4)	418 (358.5–483.7)	422.9 (355.1–488.6)	413 (351–507.1)	−3.2 (−24.4–15.2)	2.9 (−27.8–22.1)	−9.3 (−31.6–17.1)
DALYs	Number	0–9	33,0287 (184,902–428,896)	178,270 (93,550–242,247)	152,017 (79,546–210,509)	171,683 (136,688–211,827)	83,500 (64,055–106,391)	88,183 (68,653–111,028)	−48 (−61.1– −8.5)	−53.2 (−67.8– −12.7)	−42 (−60.8–15.4)
		10–24	159,058 (139,954–182,635)	93,793 (80,775–108,490)	65,265 (56,552–76,177)	201,063 (159,529–250,944)	114,597 (90,343–143,348)	86,466 (66,155–111,963)	26.4 (7.4–48.5)	22.2 (0.8–44.9)	32.5 (9.5–63.9)
		25–49	318,486 (283,164–356,231)	171,060 (148,284–196,008)	147,427 (130,421–166,356)	752,106 (622,175–910,908)	387,552 (313,965–469,712)	364,554 (300,917–458,858)	136.2 (106.4–170.4)	126.6 (90.2–162.6)	147.3 (110.9–197.9)
		50–74	734,180 (667,078–851,169)	359,434 (322,439–441,343)	374,746 (333,813–439,535)	1,623,286 (1,374,042–1,910,759)	807,369 (662,622–942,036)	815,917 (687,193–1,018,426)	121.1 (80.1–161)	124.6 (72.7–165.5)	117.7 (72.5–176.9)
		75 plus	225,028 (194,414–297,141)	114,875 (99,578–163,341)	110,153 (91,989–147,996)	633,185 (557,738–727,143)	326,350 (280,034–371,441)	306,835 (263,343–371,242)	181.4 (124.8–227)	184.1 (109.5–229.7)	178.6 (112–249.7)
	Rate	0–9	150.6 (84.3–195.6)	166.6 (87.4–226.4)	135.4 (70.9–187.6)	68.5 (54.5–84.5)	68.5 (52.6–87.3)	68.5 (53.3–86.2)	−54.5 (−66–−19.9)	−58.9 (−71.7– −23.3)	−49.4 (−65.8–0.6)
		10–24	144 (126.7–165.4)	174 (149.9–201.3)	115.4 (100–134.7)	124.7 (98.9–155.6)	146.9 (115.8–183.7)	103.8 (79.4–134.5)	−13.4 (−26.5–1.7)	−15.6 (−30.4–0.1)	−10 (−25.7–11.3)
		25–49	337.6 (300.1–377.6)	373.5 (323.8–428)	303.7 (268.7–342.7)	328.5 (271.7–397.8)	358.4 (290.3–434.3)	301.7 (249–379.7)	−2.7 (−15–11.4)	−4.1 (−19.4–11.2)	−0.7 (−15.3–19.7)
		50–74	1,106.1 (1,005–1,282.4)	1,111.6 (997.1–1,364.9)	1,101 (980.7–1,291.4)	967.5 (818.9–1,138.8)	992.2 (814.3–1,157.6)	944.2 (795.3–1,178.6)	−12.5 (−28.8–3.2)	−10.7 (−31.4–5.5)	−14.2 (−32.1–9.1)
		75 plus	3,902.8 (3,371.8–5,153.5)	3,733.9 (3,236.7–5,309.2)	4,096 (3,420.6–5,503.2)	3,750.4 (3,303.6–4,307)	3,819 (3,277–4,346.7)	3,680.2 (3,158.5–4,452.7)	−3.9 (−23.2–11.7)	2.3 (−24.6–18.7)	−10.2 (−31.6–12.8)

Data in parentheses are 95% Uncertainty Intervals (95% UIs); DALYs, Disability-Adjusted Life Years.

**Figure 3:**
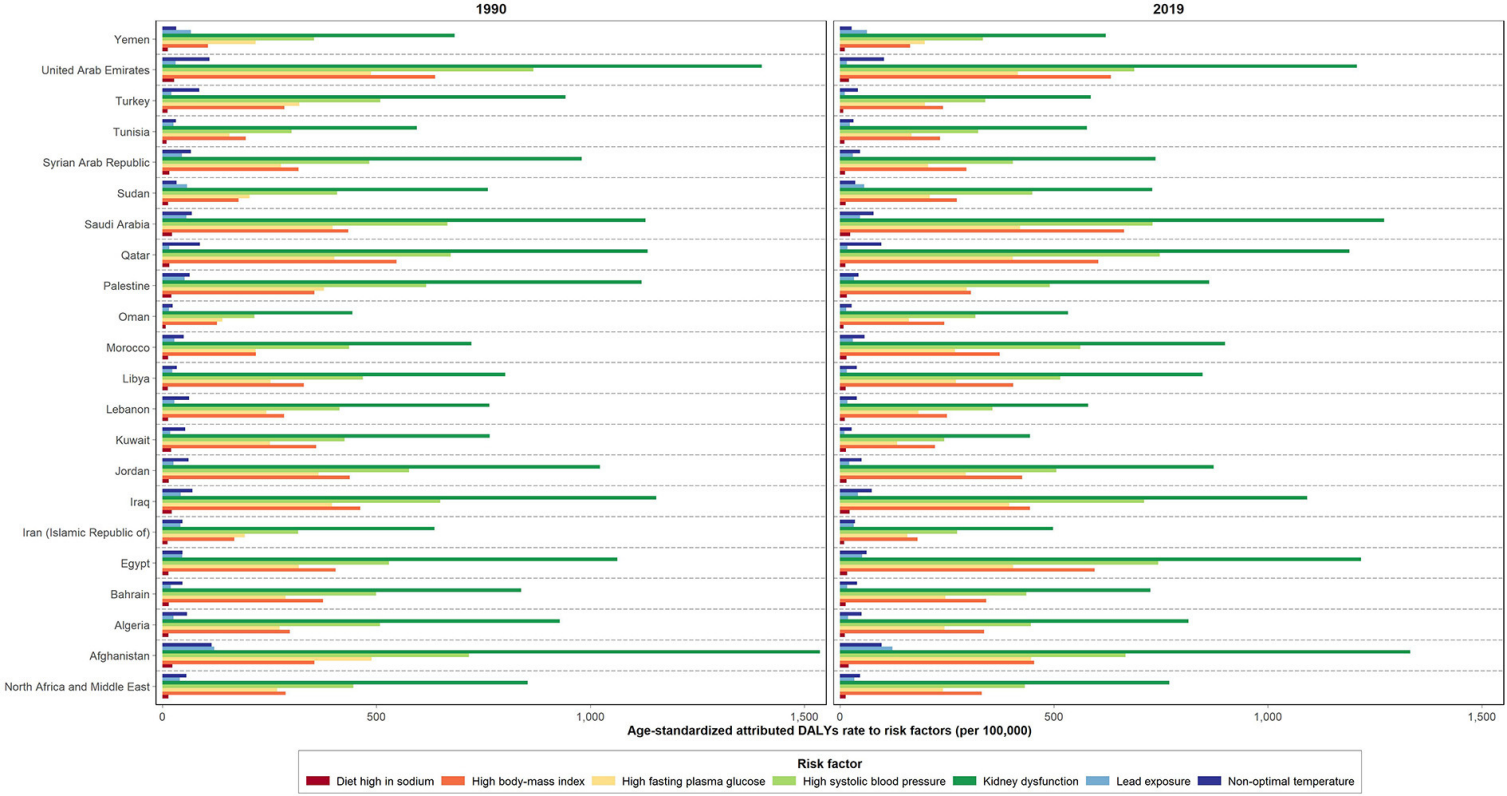
Age-standardized attributed DALYs rate due to CKD by risk factors in 1990 and 2019 in NAME region and its countries.

Despite the decrease noted in most burden measures, the age-standardized attributed YLDs rate for all CKD risk factors increased during the past 30 years (48.8%) (Table 3). Kidney dysfunction, high SBP, and high BMI ranked as the top three ASDR attributed risk factors in the region, accounting for 744.4, 424.7, and 300.1 rates per 100,000, correspondingly. This is while following a diet high in sodium accounted for the lowest attributable burden (10.3) in 2019. Kidney dysfunction, high SBP, and high BMI were also the top three risk factors for the regional CKD ASMR, responsible for 30.4, 20.0, and 12.1 attributed rates per 100,000, respectively. Following a diet high in sodium, on the other hand, accounted for the lowest attributed ASMR (0.4 per 100,000) in the same time period. From 1990 to 2019, an increase was noted in the regional ASDR and ASMR attributed to high BMI along with a decrease in that for other risk factors. The highest change, presented as decrease or increase, in the regional ASDR was due to non-optimal temperature (−16.0%) and high BMI (22.3%), respectively. Accordingly, similar changes were reported in the ASMR attributed to kidney dysfunction (−12.6%) and high BMI (18.7%), respectively. In 2019, high proportion of the CKD-related ASDR and ASMR in the region was attributed to kidney dysfunction, with the highest rates noted in Afghanistan and Saudi Arabia; 1230.4, and 52.9 per 100,000, correspondingly. Consequently, the lowest proportion of the CKD-related ASDR and ASMR attributed to following a diet high in sodium was reported in Turkey; 0.2 and 5.7, respectively (Figure 3, Supplementary Table 5, and Supplementary Figure 3).

**Table 3:** All ages number and age-standardized rate of health measures of attributed burden to CKD risk factors based on sex during 1990–2019 in NAME region.

**Measure**	**Age (metric)**	**Year**						**% Change (1990–2019)**		
		**1990**			**2019**			
		**Both**	**Female**	**Male**	**Both**	**Female**	**Male**	**Both**	**Female**	**Male**
Deaths	All ages (number)	52,596 (47,638–61,837)	26,193 (23,483–32,521)	26,403 (23,592–31,174)	111,812 (96,421–130,853)	56,399 (46,447–65,192)	55,413 (47,430–68,977)	112.6 (75.7–152)	115.3 (71.4–152)	109.9 (69–167.1)
	Age-standardized rate (per 100,000)	34.7 (30.7–44.1)	33.6 (29.7–45.5)	36.2 (31.1–45.6)	30.4 (26.3–35.4)	30.9 (25.7–35.5)	29.9 (25.7–36.9)	−12.6 (−29.4–3.9)	−8.2 (−31.1–7.5)	−17.3 (−35.8–6.4)
DALYs	All ages (number)	1,767,039 (1,616,931–1,930,042)	917,432 (827,090–1,034,476)	849,607 (775,036–927,852)	3,381,323 (2,920,288–3,897,905)	1,719,368 (1,446,769–1,971,737)	1,661,955 (1,417,431–2,036,842)	91.4 (67.1–118.3)	87.4 (57.1–116.1)	95.6 (70.1–134.9)
	Age-standardized rate (per 100,000)	834.9 (762.3–947.2)	853.6 (771.8–1,006.8)	819.2 (737.4–939.5)	744.4 (646.1–851.8)	770.0 (653.6–879.5)	720.3 (622.1–872.5)	−10.8 (−23.9–2.4)	−9.8 (−25.3–3.3)	−12.1 (−27.1–7.5)
YLLs	All ages (number)	1,530,882 (1,392,713–1,677,162)	777,338 (696,320–884,579)	753,544 (683,435–825,732)	2,616,337 (2,211,230–3,108,096)	1,307,651 (1,072,931–1,534,513)	1,308,687 (1,107,296–1,646,563)	70.9 (46–101.4)	68.2 (36–100.4)	73.7 (46.7–115.8)
	Age-standardized rate (per 100,000)	731.4 (665.2–840.8)	730.5 (657–873.7)	735.1 (660.4–852.8)	590.3 (502.9–694.7)	602.8 (494.7–698.9)	578.4 (494.5–722.5)	−19.3 (−32.7– −4.3)	−17.5 (−34.2– −2.8)	−21.3 (−36.7–0.2)
YLDs	All ages (number)	236,157 (172,238–310,169)	140,094 (102,121–183,926)	96,063 (69,130–126,698)	764,986 (559,360–1004,981)	411,717 (301,269–533,770)	353,268 (250,414–474,091)	223.9 (203.6–246.2)	193.9 (176.3–213)	267.7 (242.1–293.4)
	Age-standardized rate (per 100,000)	103.5 (76.1–135.6)	123.1 (90.8–162.0)	84.1 (60.9–111.5)	154.0 (113.1–202.3)	167.3 (123.8–215.9)	141.9 (101.3–191.7)	48.8 (41.3–56.6)	35.8 (28.8–42.9)	68.8 (59.0–78.4)

Data in parentheses are 95% Uncertainty Intervals (95% UIs); DALYs, Disability-Adjusted Life Years; YLLs, Years of Life Lost; YLDs, Years Lived with Disability.

## Discussion

To our knowledge, this is the first study to estimate the health measures and burden attributable to CKD risk factors in the NAME region. These results revealed an alarming increase in the regional burden and prevalence of CKD during past three decades, particularly among males, and those aged between 70 and 74 years. Moreover, the top three risk factors attributed to the CKD burden were kidney dysfunction, hypertension, and high BMI.

Overall, NAME is facing several health challenges apart from those secondary to the ongoing wars in the region. Several risk factors such as malnutrition, tobacco smoking, SBP, ambient air pollution, and environmental challenges, such as lack of water, sandstorms and rising temperature, have affected the health status in the region, leading to an increase in DALYs (14). Based on the GBD study, child and mother malnutrition, smoking, and lack of water risk factors' DALYs in the NAME region were estimated as 6.33, 5.11, and 4.11% in 2017, respectively (15). Moreover, the broad variation reported in the per capita gross national product of NAME countries is also influencing DALYs (16). These facts have put a strain on the limited resources and the positive impact of the health gains achieved so far.

In 2019, nearly 700 million individuals suffered from CKD globally, among them 7% (50 million) were from the NAME region. On the other hand, CKD resulted in 18.37 deaths per 100,000 persons, accounting for >3% of total death. It could be estimated that in 2040, 2.2–4.0 million deaths (17), with 0.6–1.2 million of them being due to CKD, will happen in NAME.

Since 1990, the CKD-related ASIR, ASPR, and ASYLDR have increased by nearly two-four folds in the region. The highest proportion of CKD DALYs was due to “other and unspecified causes” followed by “diabetes type 2” and “hypertension,” respectively. This is in line with the findings of the GBD 2019 (12), in which diabetes was shown as one of the main causes of the increased DALYs, especially among older adults and females, in the past three decades. Low accessibility and not efficient quality of healthcare in the NAME region compared to the European and Western Pacific regions are among the main contributors to these findings (18, 19). In addition, a considerable increase in the burden attributed to the CKD risk factors was due to the high SBP and high BMI rates in the region (13). Based on our findings, the ratio of all estimates was higher among females. This gender disparity can be explained partially by risk exposures. However, all the estimates also call for urgent measures to address the burden of diabetes, hypertension, and overweight /obesity in the region.

The ASMR, ASDR, and ASYLLR of CKD have decreased during the past 30 years. Moreover, the ranking of CKD-related death has increased from 10^th^ in 1990 to 5^th^ in 2019, based on the GBD 2019 (20). The decrease noted in the global ASDR for CKD during the same period is accompanied by a decrease in the ASMR rather than the prevalence rate, which has increased in the same time period. This can be explained due to the upward shift noted in the CKD rank among the 50–74 and older age groups (11). However, intra-country variations were noted in the ASMR of the region. The decline noted in the ASMR secondary to cardiovascular diseases was partially behind this reduction, according to the GBD 2019 report (12). The paradox between the rising metabolic risks and decreasing age-standardized CKD mortality rate can be secondary to disparities in access to care (21). Accordingly, the highest and lowest reductions were reported in Kuwait and Sudan, respectively. In addition, we found a decrease in the ASMR rate, with the highest reduction noted among males, pointing out gender disparities in access to CKD treatments (22).

In the NAME region, the ASDR decreased in most countries except for Egypt, Libya, Morocco, Oman, and Saudi Arabia. This was mainly due to the increasing change percent of “high BMI.” According to the annual report published by the Ministry of Health (MOH) of Oman, nearly 3/4 of the registered hospital deaths were ascribed to chronic and cardiovascular diseases in 2014 (23). The results of the national NCDs screening program of the Omani adults (aged ≥40 years) in 2009 also indicated most of them (70.9%) being overweight/ obese, and others suffering from hypertension (14.4%), diabetes (8%), and CKD (9.9%) (24). Following the sharp surge in the prevalence of CKD in last three decades, the disease was considered as of the main reasons of death in Oman, based on the 2016 WHO report (25).

Kidney transplantation is the gold standard management strategy for people with end-stage renal diseases. The incidence rate of renal transplants and its variations by sex and region in the same time period are reported elsewhere (26). The median global incidence rate of renal transplantation was reported as 155 per million populations, with the rate varying in the region. The global incidence rate of renal transplantation was estimated at 140 per million populations (ranging from 22 to 493 in the regions of Latin America, North Africa/Middle East, South/East/Southeast Asia, and Southern sub-Saharan Africa). The incidence rate was higher among men in all countries; in Jordan, however, more women (70 vs. 51 per million populations) received renal transplant (26).

The highest proportion of the CKD-related ASMR, especially among males, was attributed to high BMI. This might be ascribed to an ethnic predisposition to insulin resistance coupled with high-caloric unhealthy diets in the NAME countries (27). However, based on the GBD 2019 report, high BMI was one of leading risks of CKD in 2019 (28). National studies from the region have shown a positive association between obesity and chronic diseases such as diabetes type 2, hypertension, dyslipidemia, and other risk factors of CVDs, resulting in an increase in the economic cost and burden of high BMI co-existing with CKD (26, 29). These studies highlight the importance of reducing the prevalence of overweight/ obesity in the region.

In other words, the GBD data indicate multi-factors, namely aging, high prevalence of risk factors such as high BMI, geography, availability of/access to appropriate care and management, to be responsible for the variations noted in the CKD deaths reported around the world (1). A strong association is also established between high BMI and new-onset CKD (30). Higher BMI is associated with compensatory hyperfiltration due to the heightened metabolic demands, increased intraglomerular pressure, progression in GFR loss over time, increased rate of nephrolithiasis, and several malignancies in kidney (31, 32). Due to the asymptomatic nature of CKD, the reduction of modifiable CKD risk factors including obesity and diabetes through establishing screening clinics, educating physicians, raising public awareness, and lifestyle modifications, is the most important approach to lower its burden. One of the best approaches is screening individuals by taking urine and blood samples, especially in high-risk groups such as patients with diabetes, hypertension, and elderly subjects. Previously, the cost-effectiveness of such preventive strategies to reduce the burden of CKD has been evaluated (33). It is expected that diagnosis and appropriate referrals to specialists would play an important role in reducing the burden of CKD worldwide. In addition, policymakers should focus on public education and awareness as the main preventive measures in this regard.

The current study has several strengths. Our study includes one important innovation. We update the previous list of avertable burden conditions and insert other health measures including burden attributable to CKD risk factors. This approach allows for the identification of causes contributing to changes in the CKD burden in NAME. Moreover, the estimation of valuable and reliable health measures of CKD in the region allows policymakers to compare how neighboring countries have succeeded (or not) and help them to implement appropriate interventional programs in line with the WHO Global NCD Action Plan (25 by 25) and Sustainable Development Goal 4 (SDG4) (33, 34). The main limitation of our study is under-estimation of CKD due to the unavailability of data, and high number of undiagnosed cases for several countries in the region. The vital registration system is not established for many of the NAME countries. This is while the GBD methodology applies different statistical methods to decrease the estimation bias. Some of the GBD methodologies, including standardized methods and uncertainty intervals for each estimate, are among those helping to reduce the effect of data quality.

Our study provokes an alarm for the increasing global rank of the age-standardized prevalence rate of CKD in NAME from fifth to first, contributing to a substantial proportion of the disease burden. This highlights the need for the effective management of CKD risk factors, especially hypertension and overweight/obesity at primary care levels, implementation of screening measures for at-risk groups for CKD, and facilitation of the accessibility of patients to appropriate treatments.

## Data availability statement

Publicly available datasets were analyzed in this study. This data can be found at: https://vizhub.healthdata.org/gbd-results/ name of the repository: GBD Results tool.

## Ethics statement

The studies involving human participants were reviewed and approved by Endocrine and Metabolism Research Institute, Tehran University of Medical Sciences with the ethics code of IR.TUMS.EMRI.REC.1400.024. Written informed consent for participation was not required for this study in accordance with the national legislation and the institutional requirements.

## Author contributions

FF and BL designed the study. SS, YT, ZE, and PS extracted data and analyzed. OT-M and SS wrote the draft of the manuscript and interpreted data. OT-M, PK, MK, M-RM, M-MR, AG, MM, FG, and EA revised manuscript. All authors read and approved the final manuscript.

## Funding

The GBD study received funding from the Bill and Melinda Gates Foundation. Funders had no role in the design and conduct of the study; collection, management, analysis, and interpretation of the data; preparation, review, or final approval of the manuscript; and decision to submit the manuscript for publication.

## Conflict of interest

The authors declare that the research was conducted in the absence of any commercial or financial relationships that could be construed as a potential conflict of interest.

## Publisher's note

All claims expressed in this article are solely those of the authors and do not necessarily represent those of their affiliated organizations, or those of the publisher, the editors and the reviewers. Any product that may be evaluated in this article, or claim that may be made by its manufacturer, is not guaranteed or endorsed by the publisher.

## Abbreviations

ASDR: Age-standardized DALYs rate
ASIR: Age-standardized incidence rate
ASMR: Age-standardized mortality rate
ASPR: Age-standardized prevalence rate
ASYLDR: Age-standardized YLDs rate
ASYLLR: Age-standardized YLLs rate
BMI: Body-mass index
CVDs: Cardiovascular diseases
CKD: Chronic kidney disease
CRA: Comparative risk assessment
DALYs: Disability-adjusted life years
GBD: Global Burden of Disease
IHME: Institute for Health Metrics and Evaluation
ICD: International Classification of Diseases
NCDs: Non-communicable diseases
NAME: North Africa and the Middle East region
SDG4: Sustainable Development Goal 4
SBP: Systolic blood pressure
UIs: Uncertainty intervals
WHO: World Health Organization
YLDs: Years lived with disability
YLLs: Years of life lost.
