# Supplementary material for: Regional burden of chronic kidney disease in North Africa and Middle East during 1990–2019; Results from Global Burden of Disease study 2019
Source: Front Public Health. 2022 Oct 11;10:1015902. doi: 10.3389/fpubh.2022.1015902 (PMC9592811; doi:10.3389/fpubh.2022.1015902)
Supplement: Supplementary file 1 [file Data_Sheet_1.PDF]

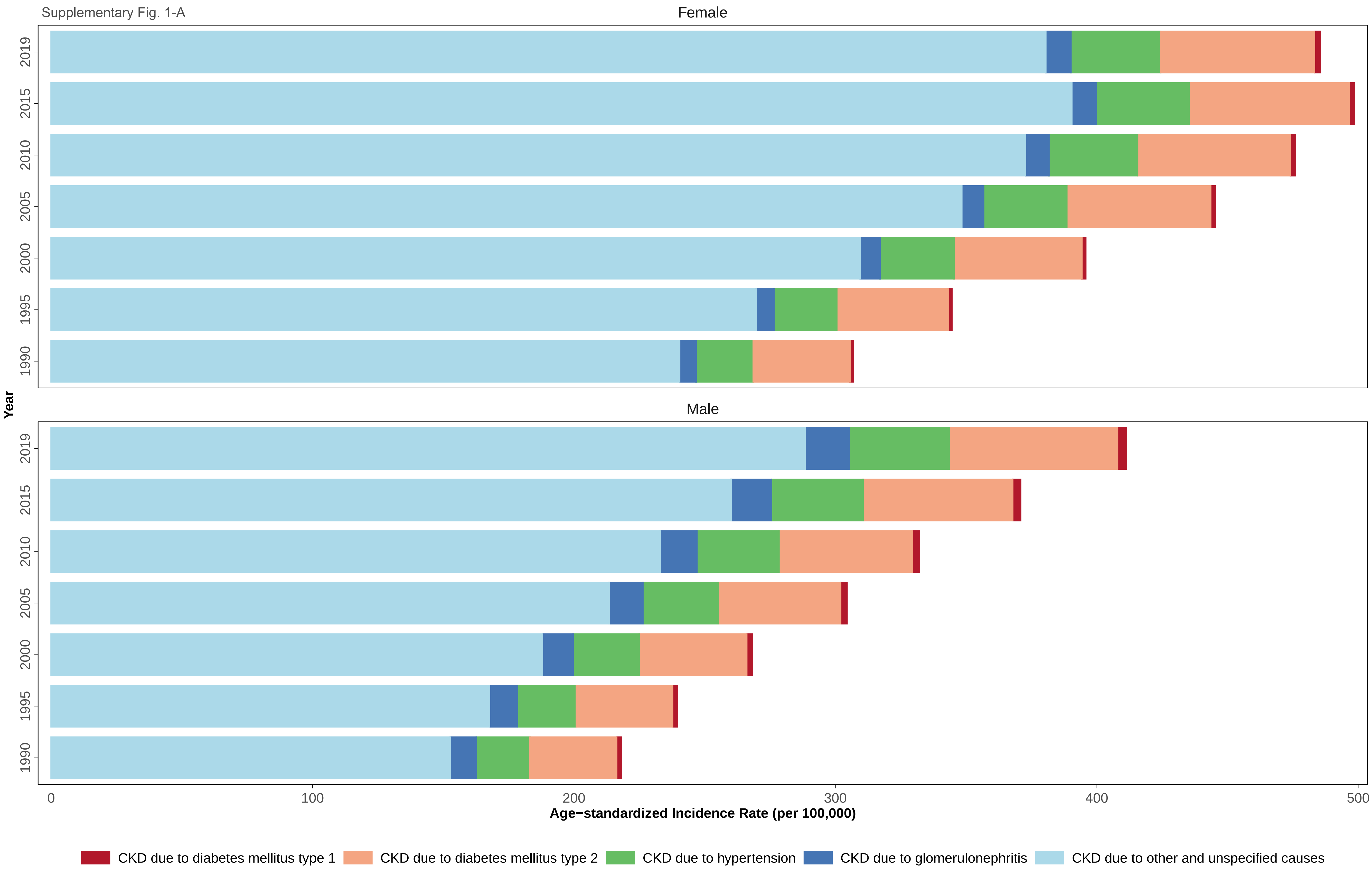
Supplementary Fig. 1-A
Female
Male
Year
2019
2015
2010
2005
2000
1995
1990
0
100
200
300
400
500
Age−standardized Incidence Rate (per 100,000)
CKD due to diabetes mellitus type 1
CKD due to diabetes mellitus type 2
CKD due to hypertension
CKD due to glomerulonephritis
CKD due to other and unspecified causes

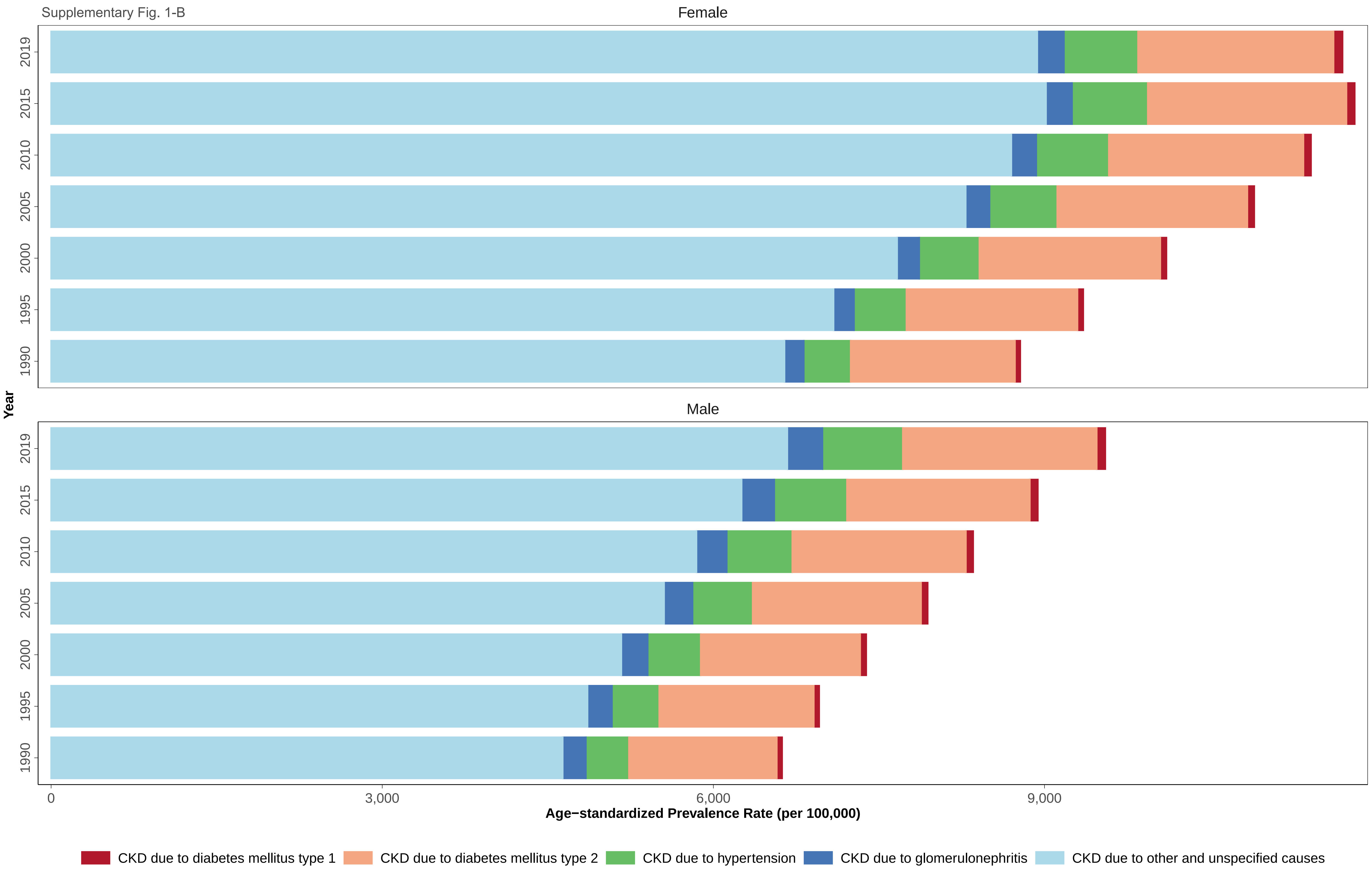
Supplementary Fig. 1-B
Female
Male
Year
2019
2015
2010
2005
2000
1995
1990
0
3,000
6,000
9,000
Age−standardized Prevalence Rate (per 100,000)
CKD due to diabetes mellitus type 1
CKD due to diabetes mellitus type 2
CKD due to hypertension
CKD due to glomerulonephritis
CKD due to other and unspecified causes

Supplementary Fig. 1-C

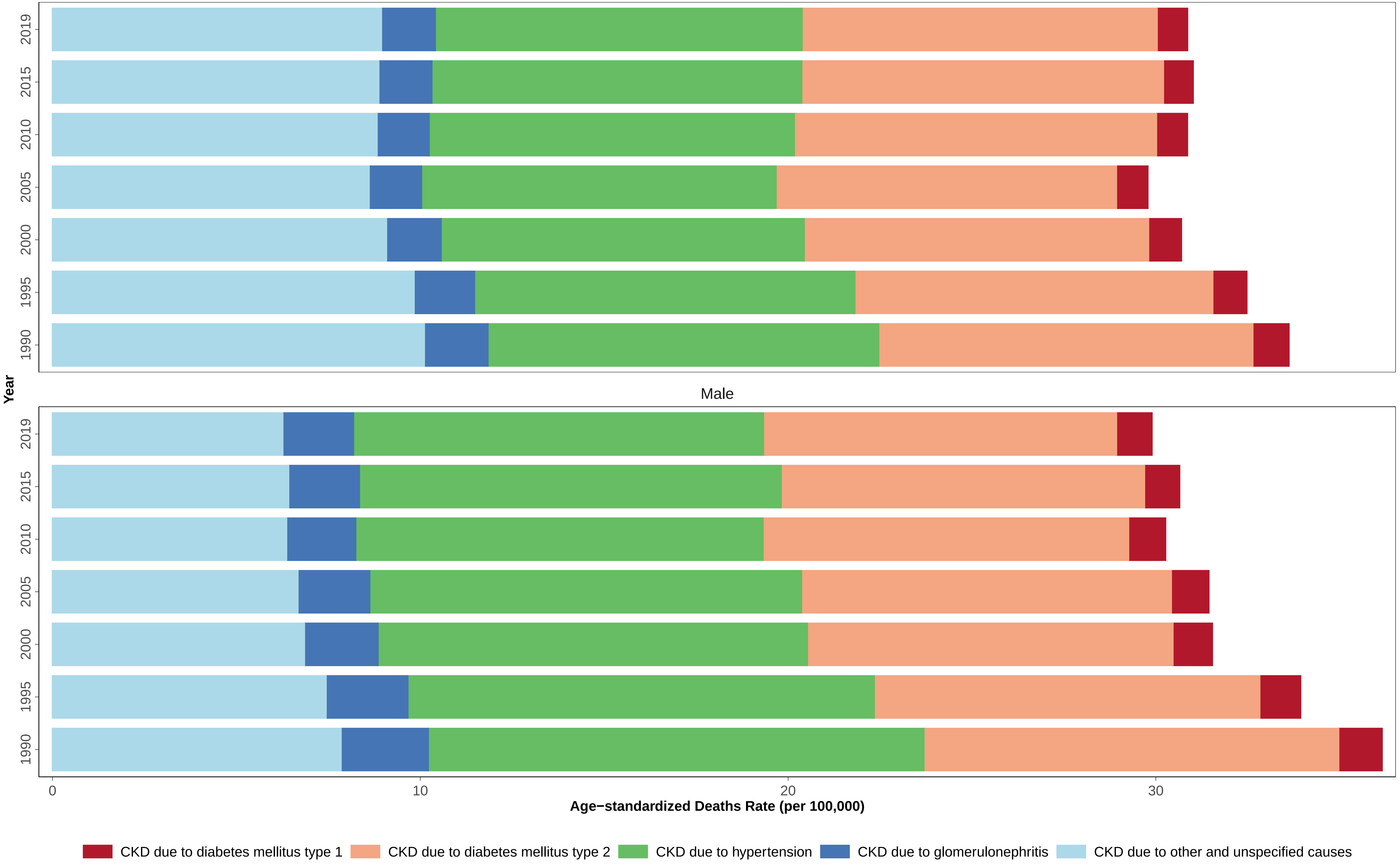

Supplementary Fig. 1-D

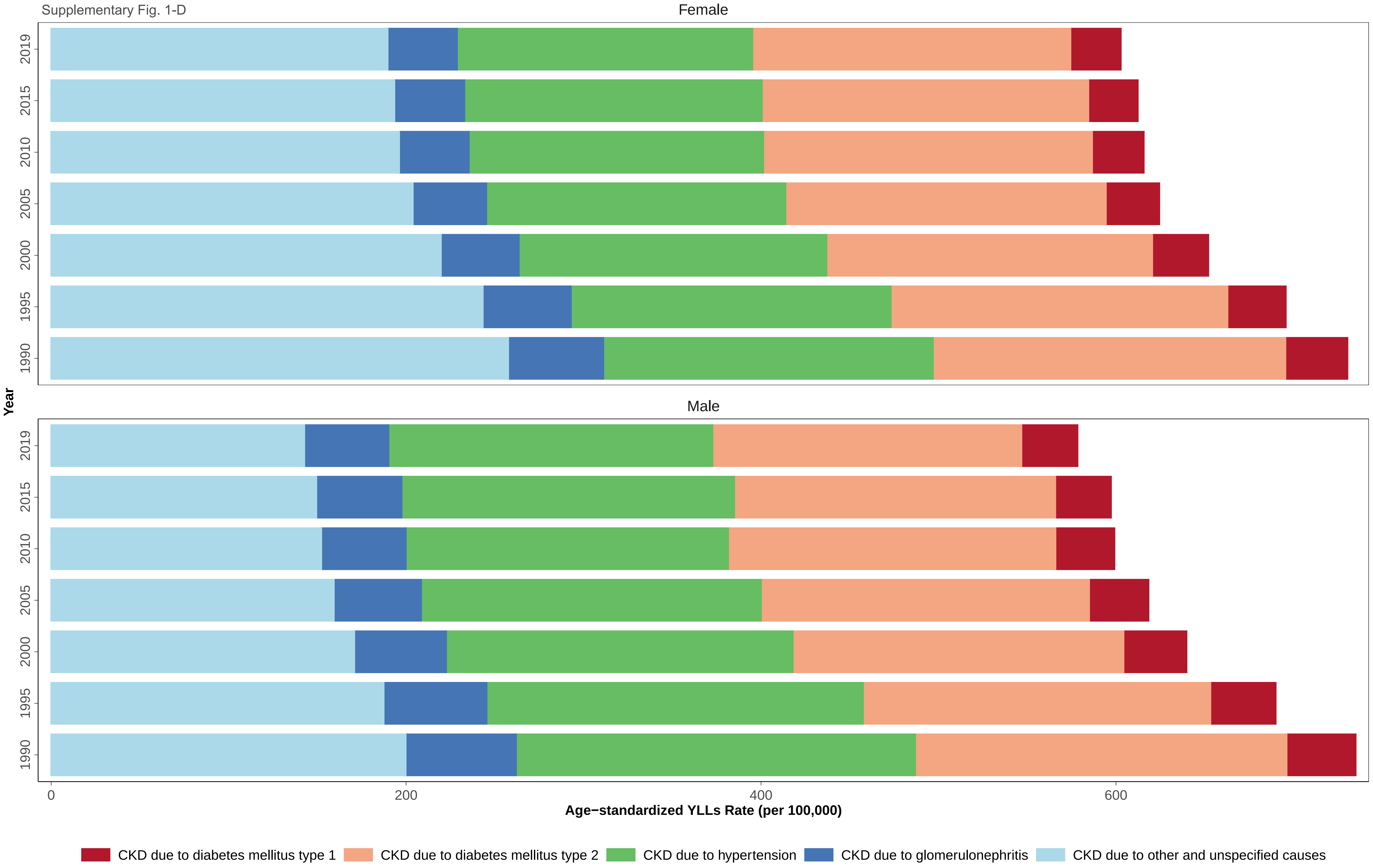

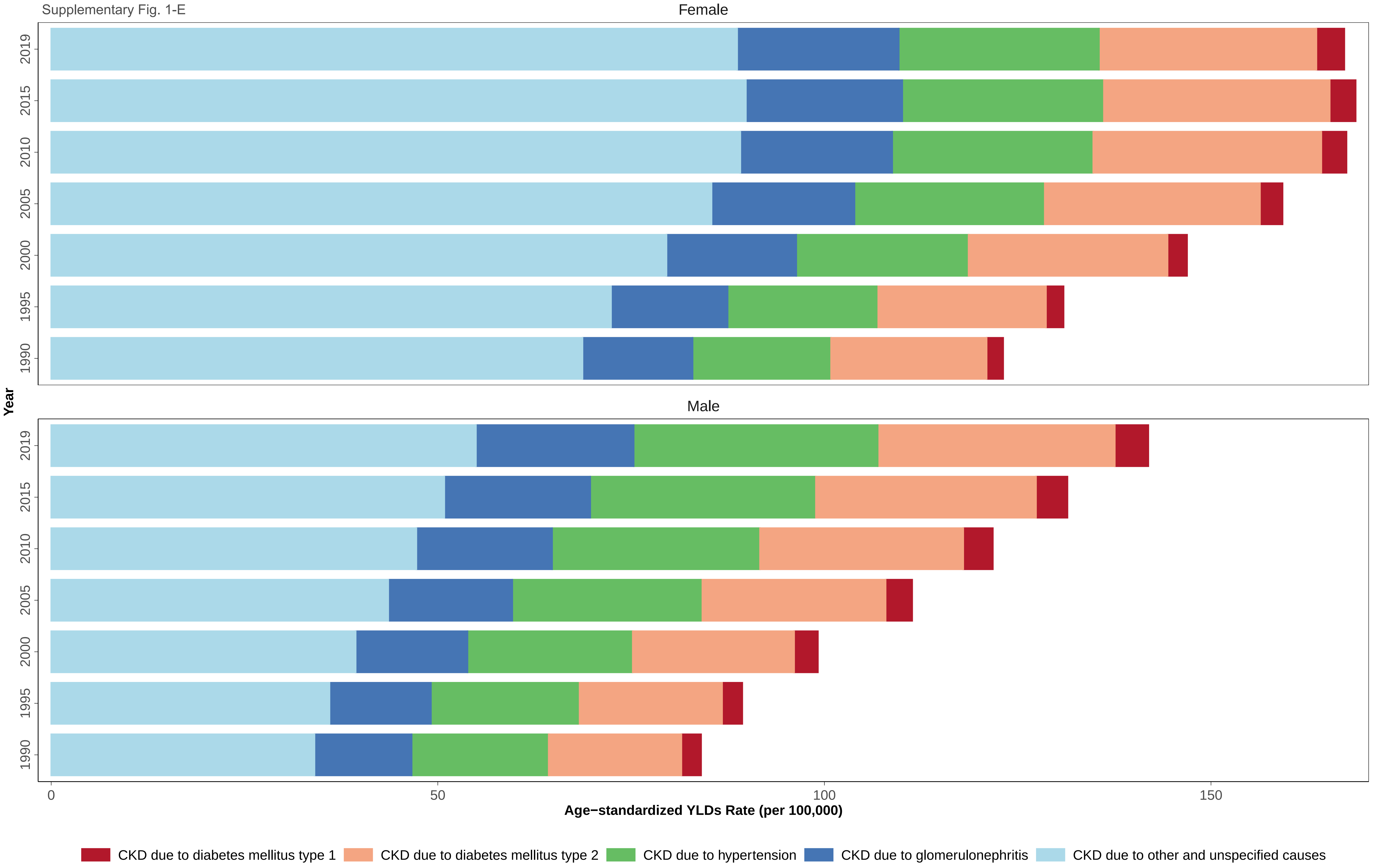

Supplementary Fig. 1-E
Female
Male
Year
2019
2015
2010
2005
2000
1995
1990
0
50
100
150
Age−standardized YLDs Rate (per 100,000)
CKD due to diabetes mellitus type 1
CKD due to diabetes mellitus type 2
CKD due to hypertension
CKD due to glomerulonephritis
CKD due to other and unspecified causes
