# Supplementary material for: Regional burden of chronic kidney disease in North Africa and Middle East during 1990–2019; Results from Global Burden of Disease study 2019
Source: Front Public Health. 2022 Oct 11;10:1015902. doi: 10.3389/fpubh.2022.1015902 (PMC9592811; doi:10.3389/fpubh.2022.1015902)
Supplement: Supplementary file 2 [file Data_Sheet_2.PDF]

Supplementary Fig. 2-A

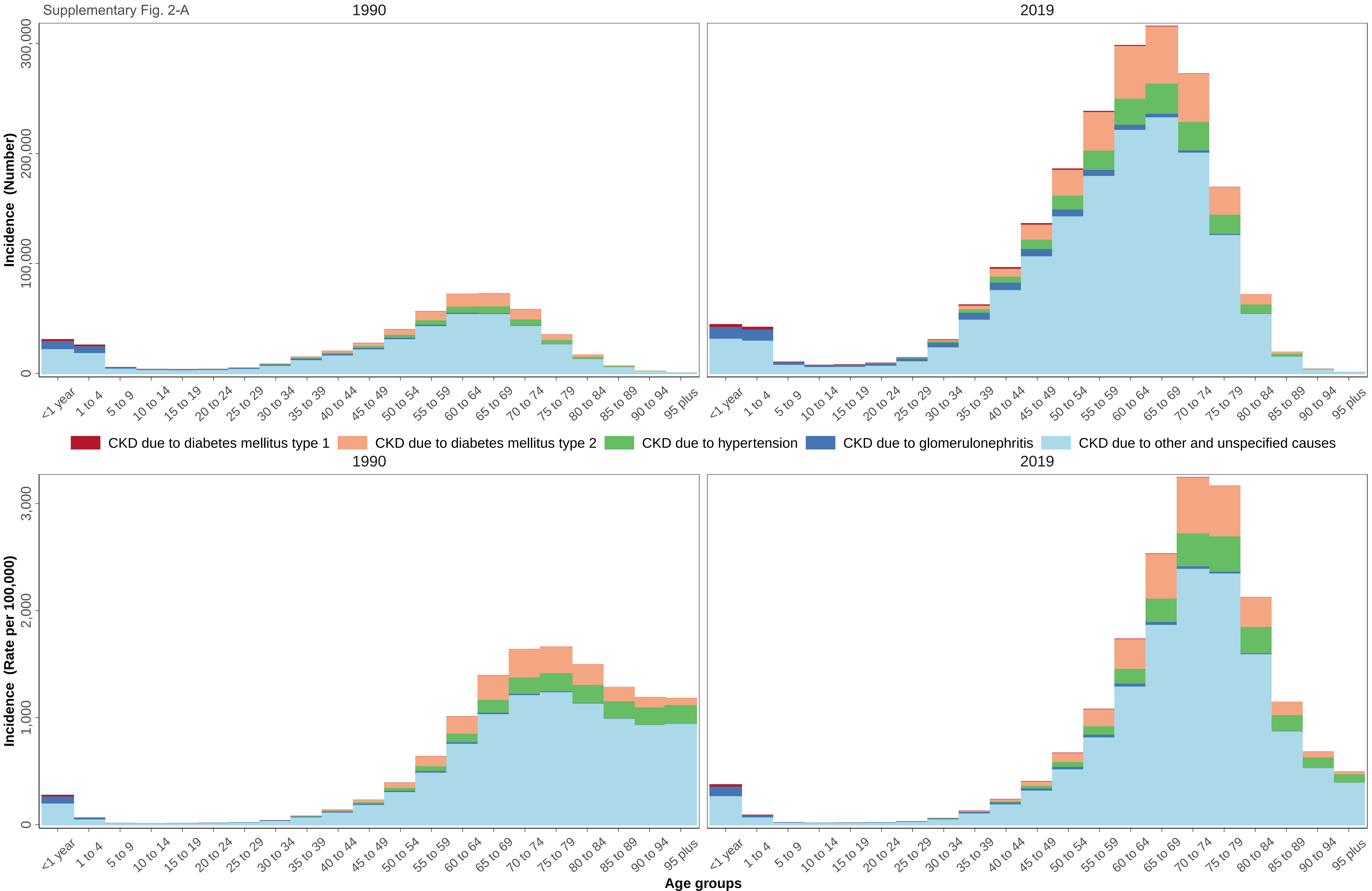

Supplementary Fig. 2-B

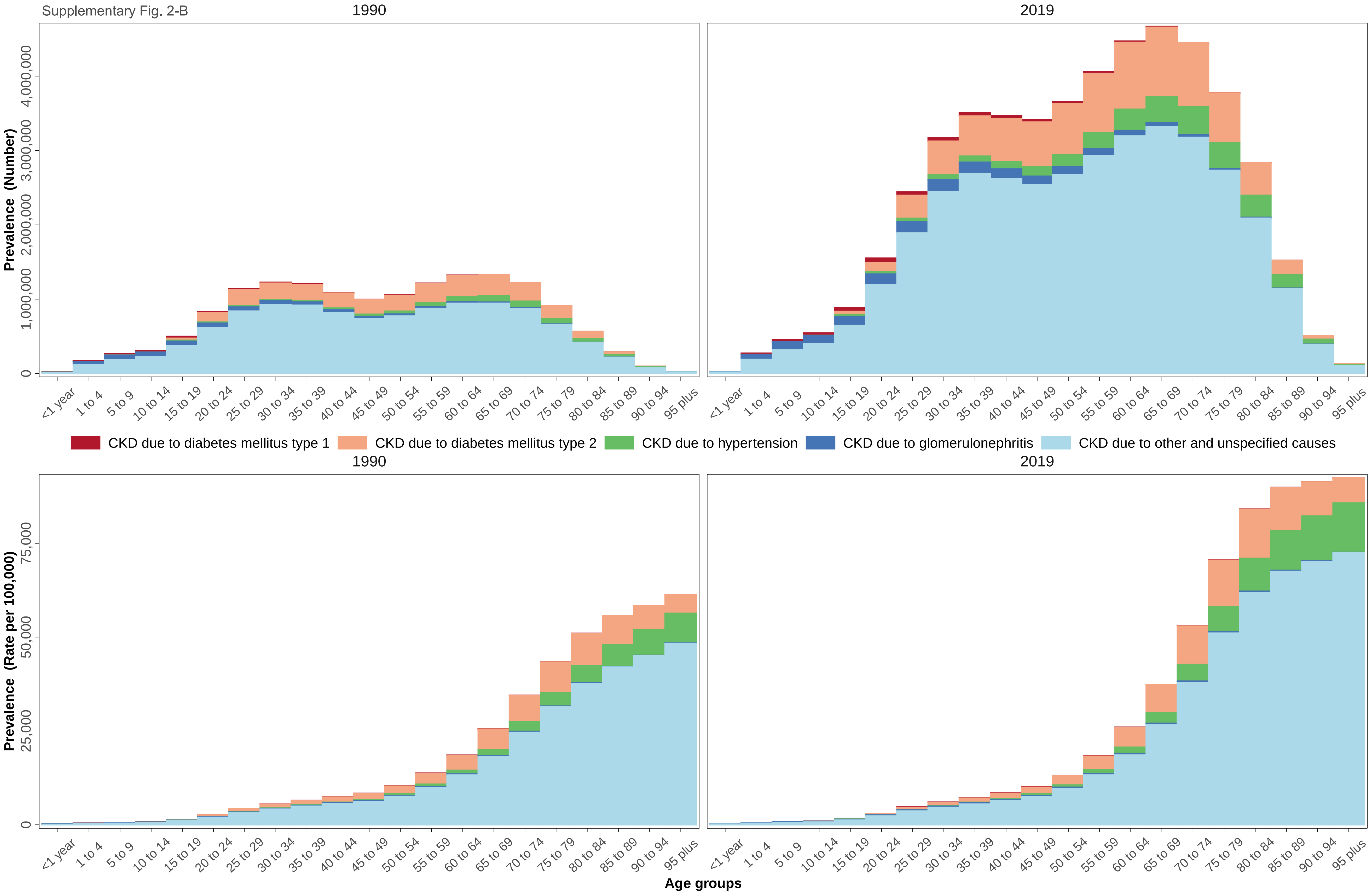

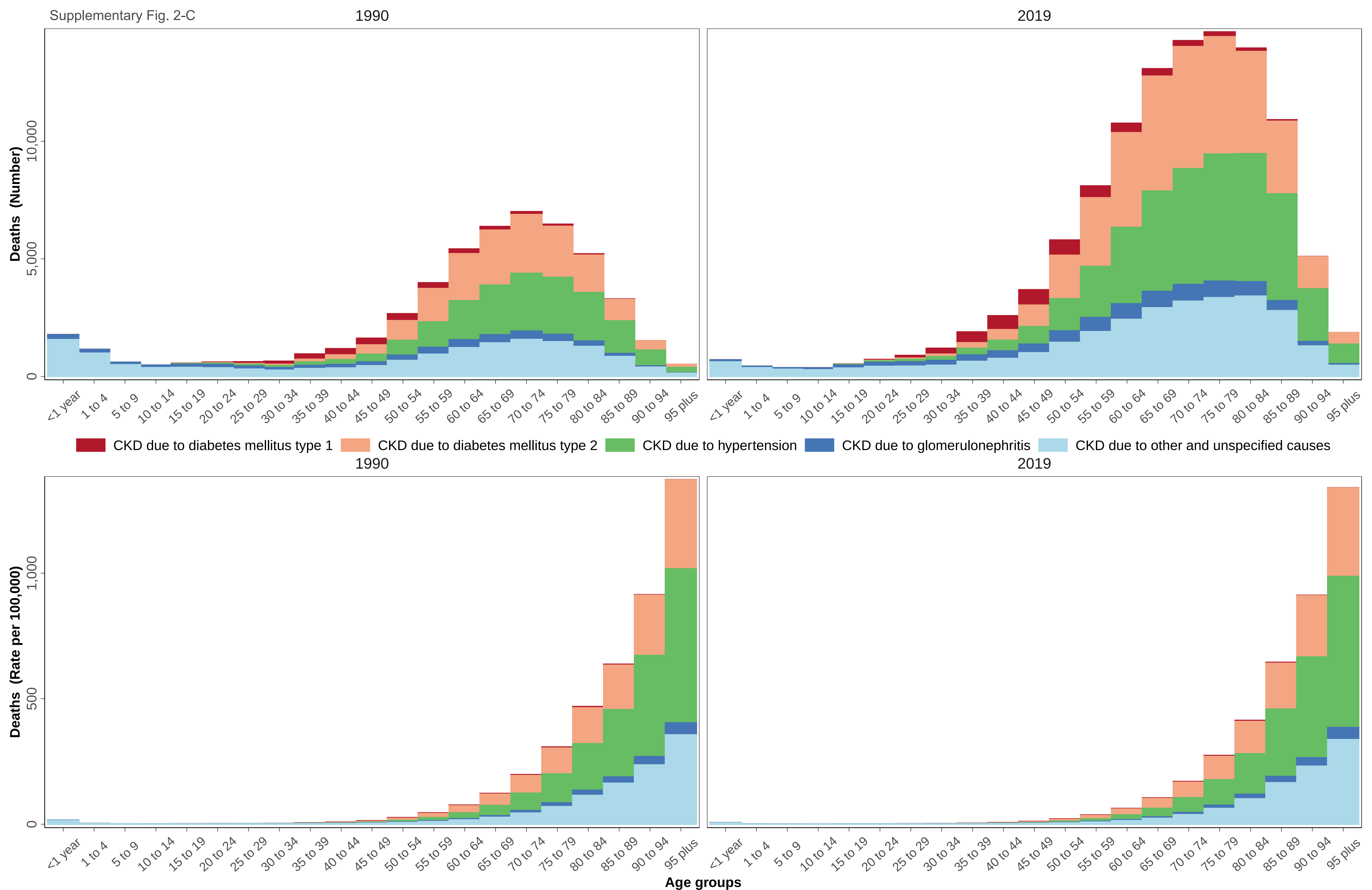
Supplementary Fig. 2-C
1990
2019
Deaths (Number)
10,000
5,000
0
<1 year
1 to 4
5 to 9
10 to 14
15 to 19
20 to 24
25 to 29
30 to 34
35 to 39
40 to 44
45 to 49
50 to 54
55 to 59
60 to 64
65 to 69
70 to 74
75 to 79
80 to 84
85 to 89
90 to 94
95 plus
CKD due to diabetes mellitus type 1
CKD due to diabetes mellitus type 2
CKD due to hypertension
CKD due to glomerulonephritis
CKD due to other and unspecified causes
Deaths (Rate per 100,000)
1,000
500
Age groups

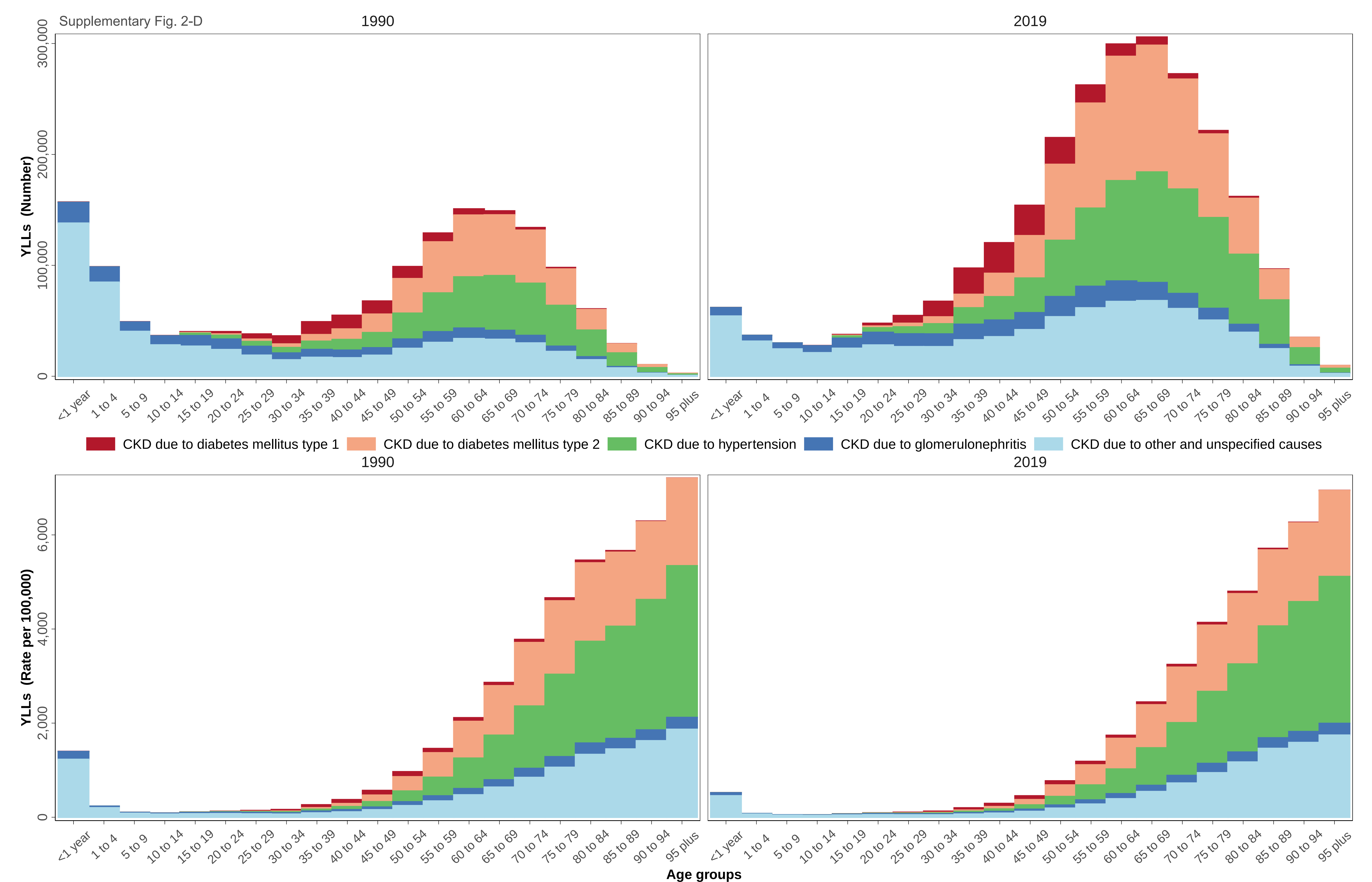
Supplementary Fig. 2-D
1990
2019
YLLs (Number)
300,000
200,000
100,000
0
<1 year
1 to 4
5 to 9
10 to 14
15 to 19
20 to 24
25 to 29
30 to 34
35 to 39
40 to 44
45 to 49
50 to 54
55 to 59
60 to 64
65 to 69
70 to 74
75 to 79
80 to 84
85 to 89
90 to 94
95 plus
CKD due to diabetes mellitus type 1
CKD due to diabetes mellitus type 2
CKD due to hypertension
CKD due to glomerulonephritis
CKD due to other and unspecified causes
YLLs (Rate per 100,000)
6,000
4,000
2,000
Age groups

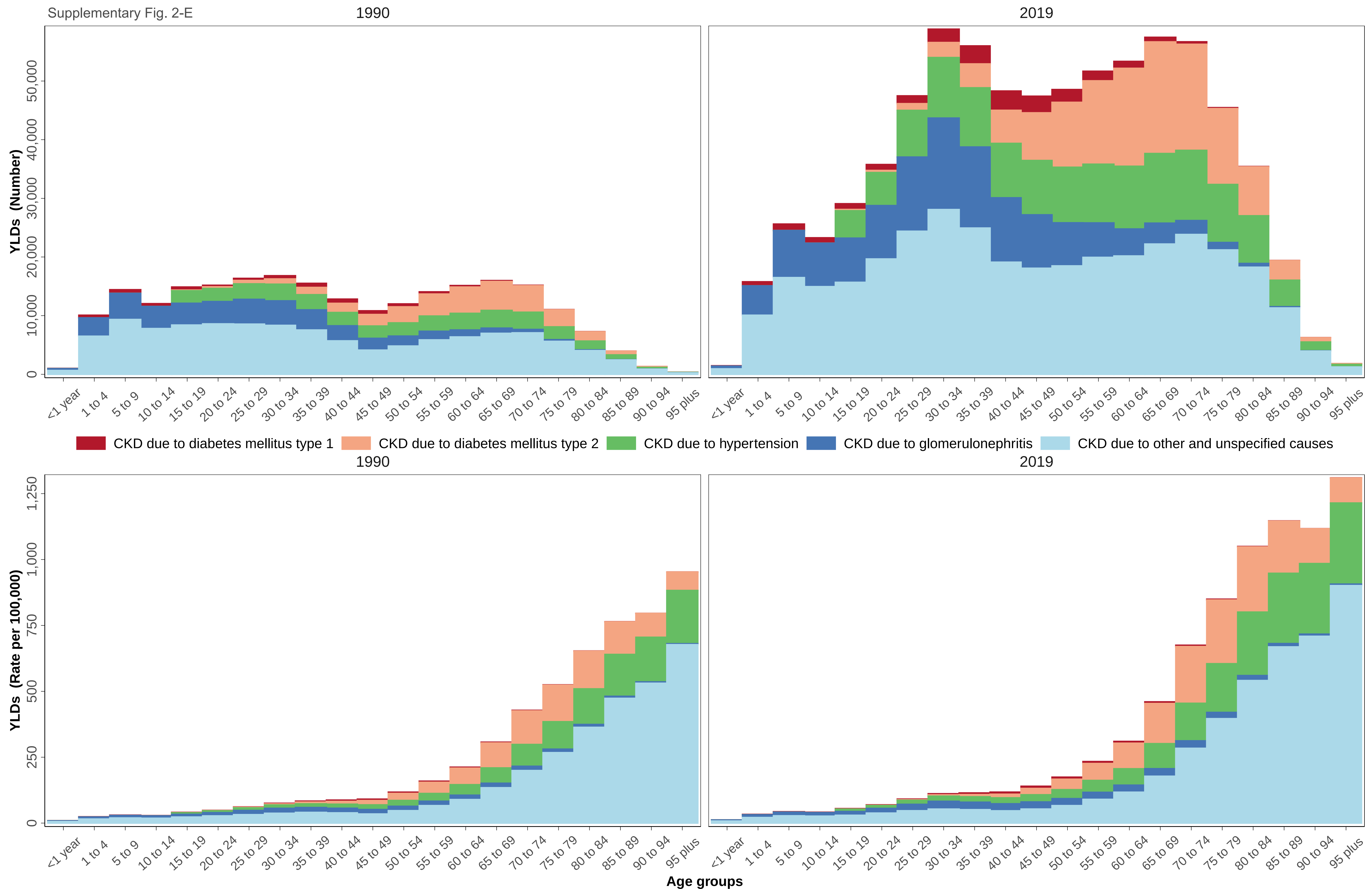

Supplementary Fig. 2-E
1990
2019
YLDs (Number)
0
10,000
20,000
30,000
40,000
50,000
<1 year
1 to 4
5 to 9
10 to 14
15 to 19
20 to 24
25 to 29
30 to 34
35 to 39
40 to 44
45 to 49
50 to 54
55 to 59
60 to 64
65 to 69
70 to 74
75 to 79
80 to 84
85 to 89
90 to 94
95 plus
CKD due to diabetes mellitus type 1
CKD due to diabetes mellitus type 2
CKD due to hypertension
CKD due to glomerulonephritis
CKD due to other and unspecified causes
YLDs (Rate per 100,000)
0
250
500
750
1,000
1,250
Age groups
