# Supplementary figures and images for: Regional burden of chronic kidney disease in North Africa and Middle East during 1990–2019; Results from Global Burden of Disease study 2019
Source: Front Public Health. 2022 Oct 11;10:1015902. doi: 10.3389/fpubh.2022.1015902 (PMC9592811; doi:10.3389/fpubh.2022.1015902)

Supplementary Fig. 3-A

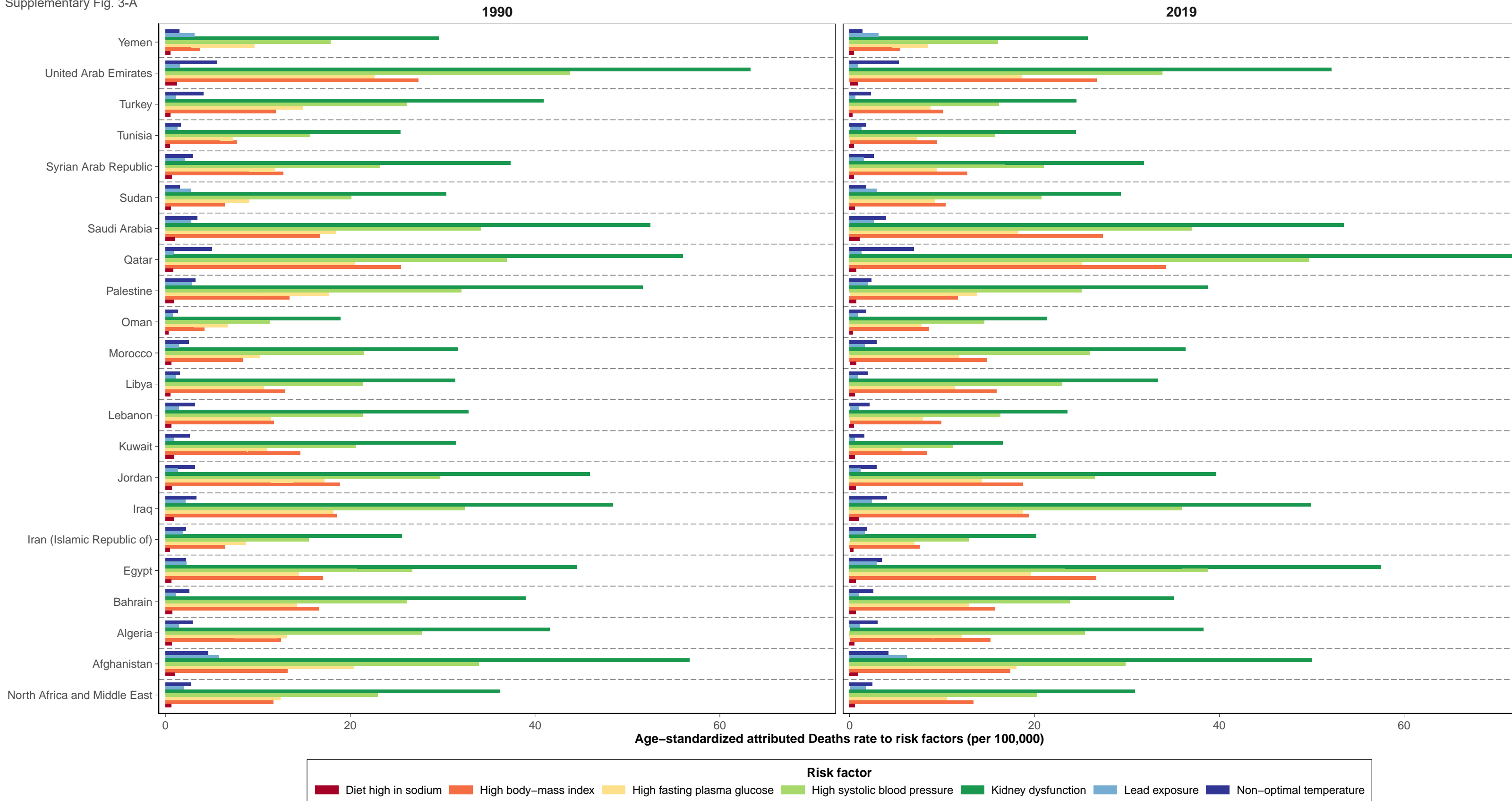

Supplementary Fig. 3-B

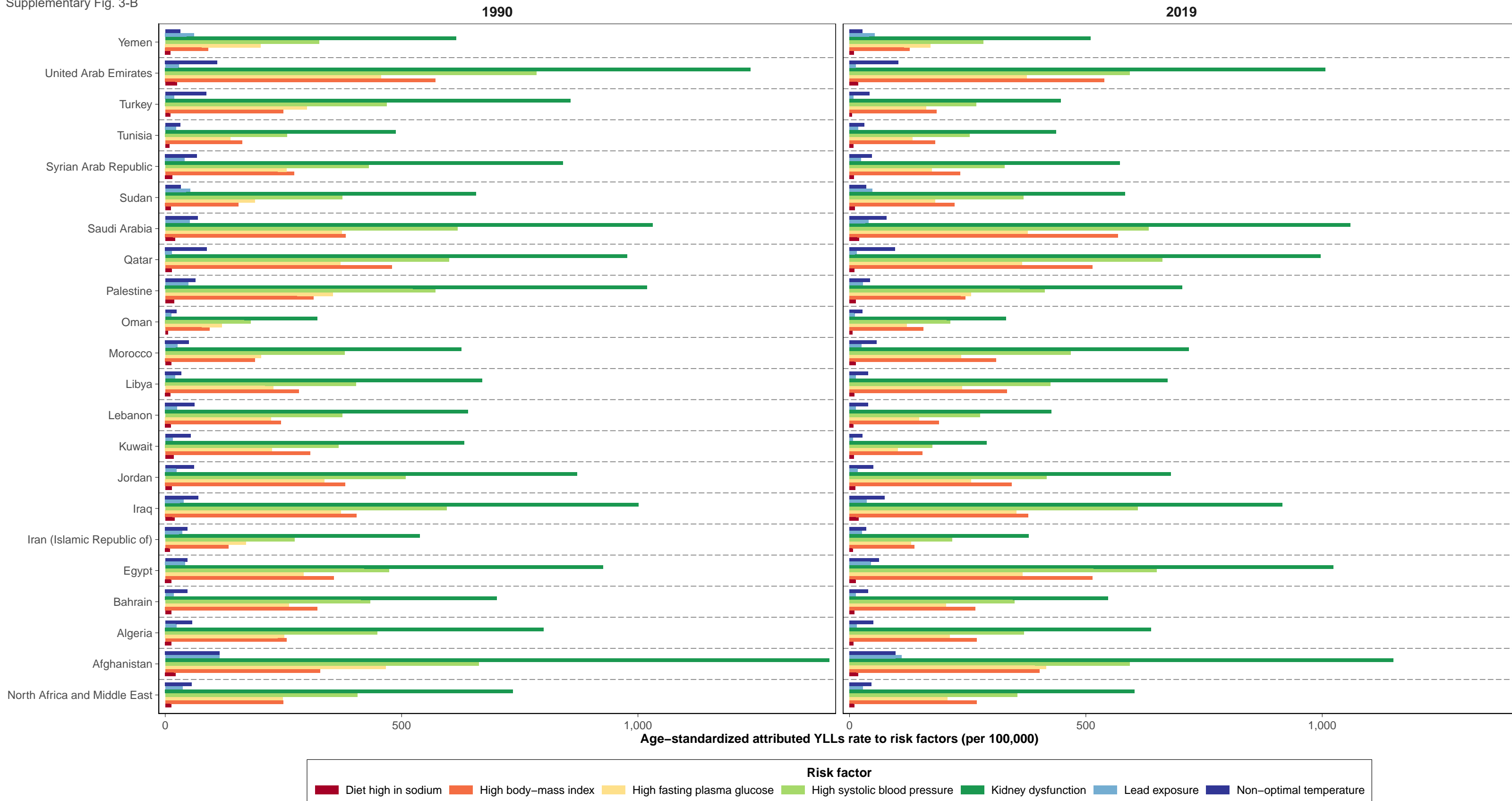

Supplementary Fig. 3-C

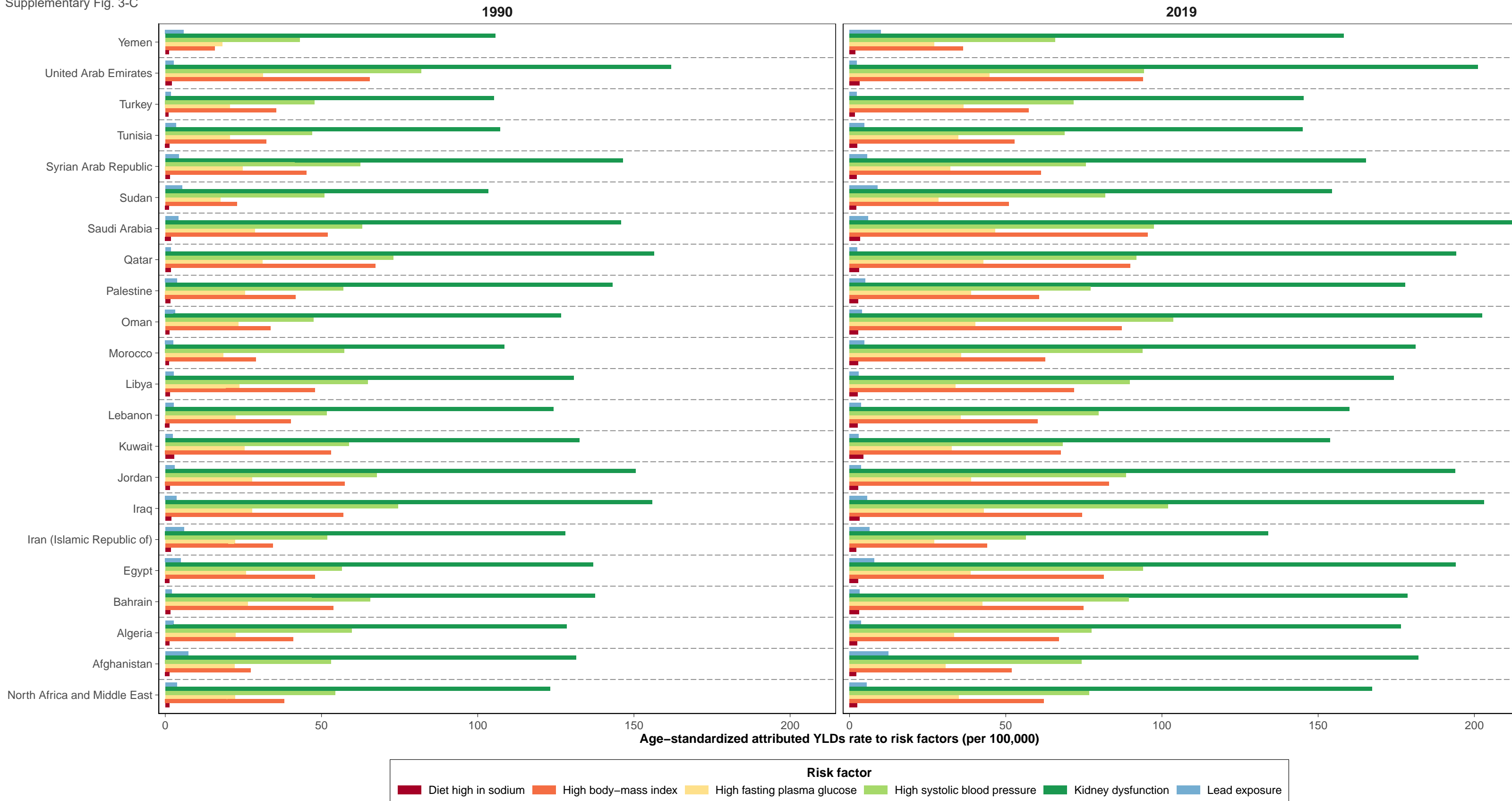

Supplement: Supplementary file 3 [file Data_Sheet_3.PDF]
