# Supplementary material for: Regional burden of chronic kidney disease in North Africa and Middle East during 1990–2019; Results from Global Burden of Disease study 2019
Source: Front Public Health. 2022 Oct 11;10:1015902. doi: 10.3389/fpubh.2022.1015902 (PMC9592811; doi:10.3389/fpubh.2022.1015902)
Supplement: Supplementary file 4 [file Data_Sheet_4.PDF]

Location		Sex	New cases		Expected new cases in 2019		% 1990 - 2019 new cases change cause			% 1990 - 2019 new cases overall change
			1990	2019	Population growth	Population growth + Aging	Population growth	Age structure change	Incidence rate change	
**North Africa and Middle East**		Both	505955	2034879	892628	1188921	76.4%	58.6%	167.2%	302.2%
		Female	293801	1085077	510668	682438	73.8%	58.5%	137%	269.3%
		Male	212154	949802	379564	505016	78.9%	59.1%	209.7%	347.7%
**Country**	Afghanistan	Both	18971	59393	63592	39205	235.2%	-128.6%	106.4%	213.1%
		Female	11084	34565	35805	24173	223%	-104.9%	93.8%	211.8%
		Male	7886	24827	27418	15059	247.7%	-156.7%	123.9%	214.8%
	Algeria	Both	37764	158844	62506	94450	65.5%	84.6%	170.5%	320.6%
		Female	23144	86904	38233	55945	65.2%	76.5%	133.8%	275.5%
		Male	14620	71940	24245	37856	65.8%	93.1%	233.1%	392.1%
	Bahrain	Both	634	5864	1799	3434	184%	258%	383.4%	825.4%
		Female	338	2297	863	1529	155.2%	197.2%	227.2%	579.6%
		Male	296	3566	901	1800	204.8%	304.2%	597.4%	1106.4%
	Egypt	Both	88968	326824	158247	187578	77.9%	33%	156.5%	267.3%
		Female	55704	172877	97735	109329	75.5%	20.8%	114.1%	210.3%
		Male	33264	153947	59933	75071	80.2%	45.5%	237.1%	362.8%
	Iran (Islamic Republic of)	Both	97326	315531	140150	234431	44%	96.9%	83.3%	224.2%
		Female	53577	176075	77658	135270	44.9%	107.5%	76.2%	228.6%
		Male	43749	139456	62603	101199	43.1%	88.2%	87.4%	218.8%
	Iraq	Both	27839	123488	66636	77904	139.4%	40.5%	163.7%	343.6%
		Female	16483	67056	39299	45911	138.4%	40.1%	128.3%	306.8%
		Male	11356	56433	27285	31908	140.3%	40.7%	216%	396.9%
	Jordan	Both	4802	36095	14809	21513	208.4%	139.6%	303.7%	651.7%
		Female	2955	18977	8914	12886	201.6%	134.4%	206.1%	542.1%
		Male	1846	17117	5809	8487	214.6%	145.1%	467.4%	827.1%
	Kuwait	Both	2285	12962	5748	8961	151.6%	140.6%	175.1%	467.4%
		Female	1131	6281	3095	4983	173.6%	166.9%	114.7%	455.2%
		Male	1153	6682	2713	4259	135.2%	134.1%	210%	479.3%
	Lebanon	Both	6617	25367	10459	14101	58.1%	55%	170.3%	283.4%
		Female	3991	14643	6475	9141	62.2%	66.8%	137.9%	266.9%
		Male	2626	10724	4044	5181	54%	43.3%	211.1%	308.4%
	Libya	Both	5866	23535	9326	14345	59%	85.6%	156.7%	301.2%
		Female	3328	12825	5361	8500	61.1%	94.3%	130%	285.4%
		Male	2538	10710	3986	6016	57%	80%	184.9%	321.9%
	Morocco	Both	31937	139947	45389	67567	42.1%	69.4%	226.6%	338.2%
		Female	19181	75761	27018	40369	40.9%	69.6%	184.5%	295%
		Male	12755	64186	18291	27113	43.4%	69.2%	290.7%	403.2%

Location	Sex	New cases		Expected new cases in 2019		% 1990 - 2019 new cases change cause			% 1990 - 2019 new cases overall change
		1990	2019	Population growth	Population growth + Aging	Population growth	Age structure change	Incidence rate change	
Oman	Both	1780	9021	4198	4597	135.9%	22.4%	248.6%	406.9%
	Female	954	4023	1920	2225	101.3%	31.9%	188.4%	321.6%
	Male	826	4999	2152	2328	160.6%	21.3%	323.6%	505.5%
Palestine	Both	3290	12685	7878	8482	139.4%	18.4%	127.8%	285.6%
	Female	1984	6773	4696	4814	136.7%	6%	98.7%	241.4%
	Male	1306	5913	3163	3562	142.1%	30.6%	179.9%	352.6%
Qatar	Both	458	6640	2946	3897	543.5%	207.8%	599.1%	1350.5%
	Female	199	1921	979	1324	392.2%	172.9%	300.1%	865.2%
	Male	259	4719	1860	2446	618.7%	226.7%	878.2%	1723.7%
Saudi Arabia	Both	22017	125644	49031	65998	122.7%	77.1%	270.9%	470.7%
	Female	11317	56547	23793	32911	110.2%	80.6%	208.9%	399.7%
	Male	10700	69097	24881	33165	132.5%	77.4%	335.8%	545.7%
Sudan	Both	22528	77084	45515	44055	102%	-6.5%	146.6%	242.2%
	Female	12815	39558	25795	24515	101.3%	-10%	117.4%	208.7%
	Male	9714	37527	19696	19357	102.8%	-3.5%	187%	286.3%
Syrian Arab Republic	Both	18046	57116	20280	35691	12.4%	85.4%	118.7%	216.5%
	Female	10542	31426	12440	21566	18%	86.6%	93.5%	198.1%
	Male	7504	25690	8030	14380	7%	84.6%	150.7%	242.3%
Tunisia	Both	13989	57591	19181	32588	37.1%	95.8%	178.7%	311.7%
	Female	8062	31569	11255	19769	39.6%	105.6%	146.4%	291.6%
	Male	5927	26022	7983	13114	34.7%	86.6%	217.8%	339.1%
Turkey	Both	85801	379089	116788	198510	36.1%	95.2%	210.5%	341.8%
	Female	48582	207136	66377	113773	36.6%	97.6%	192.2%	326.4%
	Male	37219	171954	50475	84863	35.6%	92.4%	234%	362%
United Arab Emirates	Both	2122	28002	10477	17983	393.7%	353.7%	472.1%	1219.4%
	Female	848	7813	3249	5397	282.9%	253.3%	284.7%	820.9%
	Male	1274	20190	7058	12160	454.1%	400.4%	630.3%	1484.8%
Yemen	Both	12575	52089	28856	31596	129.5%	21.8%	163%	314.2%
	Female	7384	28950	17076	18339	131.2%	17.1%	143.7%	292.1%
	Male	5191	23139	11823	13214	127.7%	26.8%	191.2%	345.7%
