# Supplementary material for: Regional burden of chronic kidney disease in North Africa and Middle East during 1990–2019; Results from Global Burden of Disease study 2019
Source: Front Public Health. 2022 Oct 11;10:1015902. doi: 10.3389/fpubh.2022.1015902 (PMC9592811; doi:10.3389/fpubh.2022.1015902)
Supplement: Supplementary file 5 [file Data_Sheet_5.PDF]

Country	Measure	Age-standardized rate (per 100,000)						% Change (1990 to 2019)		
		1990			2019					
		Both	Female	Male	Both	Female	Male	Both	Female	Male
Afghanistan	Incidence	242.22 (220.19 to 267.02)	287.3 (260.03 to 318.63)	199.59 (181.26 to 218.77)	385.21 (350.54 to 424.45)	419.63 (380.84 to 466.88)	348.45 (316.44 to 384.17)	59 (52.9 to 66.1)	46.1 (39.1 to 55.8)	74.6 (66.1 to 83.4)
	Prevalence	7202 (6684.12 to 7702.91)	8337.6 (7724.13 to 8958.07)	6038.82 (5607.34 to 6450.77)	9399.45 (8719.93 to 10084.15)	10535.92 (9750.22 to 11381.38)	8254.16 (7668.78 to 8846.13)	30.5 (26.9 to 34.7)	26.4 (22.4 to 31.3)	36.7 (31.6 to 42.2)
	Deaths	55.94 (42.01 to 81.56)	54.6 (39.76 to 87.78)	56.69 (40.36 to 83.67)	49.37 (37.14 to 74.78)	50.06 (35.16 to 84.94)	48.5 (34.08 to 75.08)	-11.7 (-33.2 to 12.5)	-8.3 (-38.8 to 24.9)	-14.4 (-35 to 8.2)
	DALYs	1436.89 (1121.17 to 1871.28)	1536.31 (1177.51 to 2125.22)	1316.09 (987.59 to 1747.69)	1230.37 (958.38 to 1680.46)	1332.78 (1004.51 to 2027.76)	1127.75 (838.61 to 1582.04)	-14.4 (-34.6 to 8.7)	-13.2 (-39.8 to 14.4)	-14.3 (-34.3 to 10)
	YLLs	1333.56 (1024.72 to 1745.66)	1404.83 (1058.3 to 1992.49)	1242.77 (920.17 to 1681.45)	1077.91 (821.64 to 1523.53)	1150.72 (830.61 to 1850.97)	1004.15 (721.86 to 1464.05)	-19.2 (-39.9 to 4.7)	-18.1 (-45.3 to 12.3)	-19.2 (-39.8 to 6.8)
	YLDs	103.33 (75.78 to 135.41)	131.48 (96.16 to 172.76)	73.32 (52.19 to 98.71)	152.46 (111.77 to 202.44)	182.05 (133.69 to 237.05)	123.6 (87.14 to 170.69)	47.5 (36.7 to 59.1)	38.5 (26.8 to 51.1)	68.6 (51 to 88.1)
Algeria	Incidence	270.77 (246.98 to 299.05)	324.05 (293.59 to 360.19)	215.7 (195.75 to 238.02)	453.09 (416.04 to 493.02)	498.47 (458.4 to 541.73)	408.53 (371.6 to 450.16)	67.3 (60.8 to 73.7)	53.8 (46.4 to 61.9)	89.4 (78.4 to 100.6)
	Prevalence	7757.7 (7236.3 to 8306.92)	9033.16 (8387.26 to 9698.29)	6525.29 (6099.91 to 6971.21)	10666 (9928.08 to 11407.85)	11942.07 (11119.76 to 12745.91)	9431.75 (8743.75 to 10136.71)	37.5 (33.3 to 41.3)	32.2 (27.6 to 36.9)	44.5 (38.6 to 50.7)
	Deaths	38.73 (30.35 to 52.07)	41.59 (29.92 to 66.88)	36.54 (28.62 to 47.42)	31.57 (24.78 to 41.16)	38.26 (28.6 to 54.35)	26.83 (20.54 to 35.7)	-18.5 (-36.6 to 5.9)	-8 (-31.6 to 23.1)	-26.6 (-45.4 to 1.9)
	DALYs	839.66 (668.56 to 1130.07)	928.59 (701.22 to 1448.44)	757.92 (606.65 to 965.86)	701.53 (567.99 to 889.89)	814.68 (634.16 to 1082.32)	604.46 (475.09 to 793.44)	-16.5 (-34 to 4.9)	-12.3 (-34.4 to 12.1)	-20.2 (-38.9 to 5.9)
	YLLs	736.53 (573.89 to 1018.1)	800.16 (580.4 to 1314.47)	678.96 (529.74 to 884.79)	544.25 (424.32 to 720.35)	638.2 (471.56 to 900.48)	465.81 (353.71 to 641.74)	-26.1 (-43.8 to -3.3)	-20.2 (-42.3 to 7.1)	-31.4 (-50 to -3.1)
	YLDs	103.13 (75.42 to 137.36)	128.43 (94.06 to 172.16)	78.96 (56.26 to 104.75)	157.28 (113.64 to 209.64)	176.49 (129.91 to 232.69)	138.65 (96.92 to 188.89)	52.5 (39.5 to 66.2)	37.4 (23.3 to 51.9)	75.6 (55.9 to 98.1)

Country	Measure	Age-standardized rate (per 100,000)						% Change (1990 to 2019)		
		1990			2019					
		Both	Female	Male	Both	Female	Male	Both	Female	Male
Bahrain	Incidence	305.9 (280.84 to 334.32)	365.35 (333.64 to 399.99)	259.03 (234.58 to 284.73)	502.93 (462.61 to 547.12)	537.64 (495.64 to 581.17)	484.36 (442.42 to 529.22)	64.4 (56.7 to 72.3)	47.2 (39.2 to 55.3)	87 (75.4 to 98.6)
	Prevalence	8242.7 (7701.66 to 8781.87)	9655.11 (8967.62 to 10331.7)	7120.38 (6648.18 to 7566.87)	11346.5 (10610.04 to 12130.92)	12548.52 (11732.04 to 13376.91)	10625.92 (9877.1 to 11424.8)	37.7 (33.2 to 42.1)	30 (24.9 to 35.2)	49.2 (42.7 to 55.6)
	Deaths	38.45 (31.97 to 46.81)	39 (30.4 to 48.19)	38.56 (31.2 to 51.06)	34.82 (27.94 to 42.19)	35.06 (27.23 to 42.58)	34.82 (27.27 to 44.83)	-9.5 (-29.6 to 13.1)	-10.1 (-34 to 16)	-9.7 (-31.4 to 17.1)
	DALYs	781.67 (663.36 to 936.29)	838.28 (673.39 to 1025.72)	743.8 (613.21 to 960.54)	711.89 (588.82 to 844.68)	725.55 (589.93 to 865.63)	707.16 (570.76 to 875.91)	-8.9 (-26.2 to 11)	-13.4 (-32 to 7.9)	-4.9 (-24.9 to 18.3)
	YLLs	669.58 (556.61 to 817.38)	700.74 (543.23 to 865.25)	651.74 (525.92 to 865.53)	540.74 (434.43 to 663.67)	546.88 (423.36 to 669.52)	540.66 (421.89 to 697.7)	-19.2 (-37.2 to 2.2)	-22 (-42.4 to 3)	-17 (-36.2 to 8.4)
	YLDs	112.09 (81.83 to 147.93)	137.54 (100.02 to 181.43)	92.06 (65.81 to 123.61)	171.16 (122.34 to 228.68)	178.67 (129.66 to 235.44)	166.5 (114.97 to 228.84)	52.7 (37.6 to 68.9)	29.9 (15.4 to 45.3)	80.9 (60 to 103.7)
Egypt	Incidence	275.6 (251.68 to 303.43)	344.75 (312.87 to 382.49)	206.75 (187.77 to 226.9)	473.45 (434.48 to 516.23)	530.89 (485.27 to 575.35)	421.61 (384.56 to 463.38)	71.8 (66.1 to 78.1)	54 (47.1 to 61.2)	103.9 (93.6 to 114.1)
	Prevalence	7727.2 (7179.19 to 8259.92)	9152.72 (8499.55 to 9868.91)	6303.45 (5868.17 to 6721.77)	10929.08 (10176.81 to 11678.59)	12417.16 (11528.24 to 13298.44)	9680.27 (8979.43 to 10400.59)	41.4 (37.8 to 45.4)	35.7 (31.3 to 40.2)	53.6 (47.5 to 60.4)
	Deaths	39.39 (27.32 to 49.32)	44.48 (25.51 to 58.94)	34.19 (26.87 to 44.77)	43.93 (26.96 to 61.79)	57.52 (26.85 to 81.21)	35.99 (23.17 to 58.5)	11.5 (-17 to 44.5)	29.3 (-13.3 to 67.9)	5.2 (-28.3 to 45.7)
	DALYs	916.35 (675.75 to 1090.29)	1062.84 (672.72 to 1346.43)	770.43 (626.65 to 985.63)	1009.46 (660.38 to 1386.31)	1217.98 (664.54 to 1653.14)	862.38 (577.16 to 1319.46)	10.2 (-13.8 to 37.8)	14.6 (-15.7 to 45)	11.9 (-17.6 to 46.9)
	YLLs	807.9 (569.11 to 974.64)	925.99 (543.86 to 1198.42)	690.16 (545.35 to 902.49)	837.02 (493.06 to 1200.31)	1023.91 (470.08 to 1463.85)	708.67 (432.18 to 1170.5)	3.6 (-23.6 to 35.4)	10.6 (-25 to 45.2)	2.7 (-30.8 to 42.8)
	YLDs	108.45 (79.97 to 144.2)	136.86 (99.81 to 181.32)	80.27 (57.91 to 108.11)	172.44 (126.24 to 228.81)	194.07 (142.8 to 254.31)	153.71 (109.48 to 208.66)	59 (45.8 to 73.2)	41.8 (28.1 to 56.5)	91.5 (71 to 113.7)

Country	Measure	Age-standardized rate (per 100,000)						% Change (1990 to 2019)		
		1990			2019					
		Both	Female	Male	Both	Female	Male	Both	Female	Male
Iran (Islamic Republic of)	Incidence	312.23 (284.08 to 342.21)	358.33 (325.89 to 395.44)	270.82 (246.25 to 297.71)	420.53 (387.18 to 456.71)	467.58 (430.5 to 505.92)	371.76 (342.17 to 404.45)	34.7 (30.8 to 38.8)	30.5 (25.6 to 35.5)	37.3 (33.6 to 41.3)
	Prevalence	9008.18 (8386.73 to 9607.92)	10067.73 (9364.73 to 10744.79)	7969.96 (7428.28 to 8476.37)	10773.42 (10101.1 to 11422.81)	11941.71 (11186.09 to 12660.97)	9617.82 (9031.23 to 10215.65)	19.6 (17.7 to 21.8)	18.6 (16.3 to 21.1)	20.7 (18.8 to 22.9)
	Deaths	24.11 (21.79 to 28.8)	22.64 (19.56 to 31.52)	25.58 (22.49 to 28.39)	19.77 (17.77 to 21.21)	19.39 (17.35 to 20.92)	20.17 (18.01 to 22.03)	-18 (-35.4 to -10.8)	-14.3 (-42.8 to -3.3)	-21.2 (-31.2 to -9)
	DALYs	620.34 (569.14 to 676.86)	602.05 (537.89 to 694.94)	636.01 (564.4 to 696.04)	486.68 (443.93 to 530.46)	475.62 (427.17 to 522.38)	498.03 (455.21 to 545.91)	-21.5 (-28.8 to -15.4)	-21 (-33.2 to -13.5)	-21.7 (-28.6 to -11.4)
	YLLs	507.44 (463.24 to 556.81)	474 (419.73 to 569.46)	538.79 (471.68 to 590.83)	360.47 (333.67 to 382.16)	341.61 (312.46 to 365.75)	379.22 (348.64 to 409.94)	-29 (-36.4 to -22.5)	-27.9 (-42.3 to -18.9)	-29.6 (-36.4 to -18.4)
	YLDs	112.9 (82.68 to 148.1)	128.05 (94.04 to 168.23)	97.22 (70.68 to 127.73)	126.21 (91.53 to 165.66)	134.02 (97.51 to 176.3)	118.81 (85.21 to 158.84)	11.8 (4.9 to 18.9)	4.7 (-3.7 to 12.8)	22.2 (13.5 to 30.2)
Iraq	Incidence	302.88 (276.4 to 333.27)	352.49 (320.13 to 389.56)	252.06 (228 to 279.42)	484.35 (445.33 to 526.24)	518.19 (477.78 to 563.82)	451.06 (410.21 to 492.24)	59.9 (53.8 to 65.9)	47 (40 to 54.1)	78.9 (69.9 to 89.5)
	Prevalence	8584.87 (8016.51 to 9162.67)	9848.36 (9204.14 to 10519.87)	7288.12 (6776.58 to 7816.93)	11560.45 (10782.49 to 12343)	12653.02 (11795.81 to 13527.19)	10461.05 (9715.55 to 11245.04)	34.7 (30.8 to 38.3)	28.5 (24.3 to 33.2)	43.5 (37.7 to 49.1)
	Deaths	46.27 (37.12 to 61.87)	44.64 (33.52 to 66.33)	48.44 (37.34 to 68.84)	41.86 (33.19 to 53.49)	35.34 (27.56 to 45.77)	49.93 (36.9 to 72.49)	-9.5 (-30.1 to 11.5)	-20.8 (-43.5 to 7.8)	3.1 (-24 to 35.6)
	DALYs	1126.83 (933.87 to 1407.75)	1154.23 (904.06 to 1603.06)	1104.95 (875.39 to 1414.94)	991.2 (797.31 to 1215.04)	905.37 (709.55 to 1133.98)	1091.92 (833.62 to 1403.18)	-12 (-30.2 to 8.2)	-21.6 (-41.4 to 3.2)	-1.2 (-24.6 to 27.1)
	YLLs	996.98 (808.42 to 1279.53)	998.43 (753.7 to 1438.19)	1001.28 (774.72 to 1310.77)	802.01 (615.85 to 1021.42)	702.24 (523.99 to 941.05)	916 (676.71 to 1226.47)	-19.6 (-38.9 to 2.9)	-29.7 (-50.2 to -2)	-8.5 (-33.5 to 21.7)
	YLDs	129.85 (94.65 to 171.37)	155.81 (114.03 to 204.35)	103.66 (74.25 to 138.69)	189.19 (138.29 to 248.21)	203.14 (149.5 to 262.16)	175.93 (124.91 to 238.81)	45.7 (34.2 to 57.5)	30.4 (18.4 to 42.9)	69.7 (52.5 to 89.4)

Country	Measure	Age-standardized rate (per 100,000)						% Change (1990 to 2019)		
		1990			2019					
		Both	Female	Male	Both	Female	Male	Both	Female	Male
Jordan	Incidence	296.93 (273.34 to 322.44)	375.66 (344.51 to 407.31)	221.28 (200.31 to 244.28)	494.06 (459.04 to 531.83)	544.45 (503.59 to 585.79)	448.01 (410.68 to 491.27)	66.4 (58.1 to 74.5)	44.9 (36.2 to 53.4)	102.5 (87.8 to 118.1)
	Prevalence	8305.13 (7738.57 to 8832.38)	9870.14 (9190.59 to 10540.84)	6779 (6299.45 to 7254.87)	11379.38 (10691.51 to 12088.59)	12699.49 (11903.15 to 13522.99)	10219.61 (9555.73 to 10985.74)	37 (32.5 to 41.7)	28.7 (23.4 to 34)	50.8 (43.8 to 58.7)
	Deaths	40.92 (34.47 to 48.38)	45.91 (36.02 to 57.53)	35.91 (29.44 to 43.21)	36.65 (30.74 to 43.21)	39.66 (31.45 to 48.37)	34.04 (27.04 to 42.68)	-10.4 (-29.2 to 11.9)	-13.6 (-39.2 to 15.4)	-5.2 (-30.5 to 29.2)
	DALYs	899.41 (777.58 to 1042.36)	1021.92 (851.81 to 1234.9)	780.25 (653.25 to 932.23)	825.82 (707.85 to 957.14)	873.45 (720.84 to 1055.86)	782.83 (634.95 to 950.9)	-8.2 (-25.3 to 11.2)	-14.5 (-35.6 to 8.6)	0.3 (-22.7 to 32.2)
	YLLs	780.95 (661.23 to 912)	871.35 (693.97 to 1078.8)	692.67 (565.4 to 841.1)	653.07 (545.61 to 774.73)	679.58 (533.23 to 846.11)	627.89 (494.15 to 786.04)	-16.4 (-34.1 to 5.4)	-22 (-45.5 to 4)	-9.4 (-33.6 to 24.5)
	YLDs	118.45 (86.1 to 156.32)	150.57 (109.29 to 197.9)	87.58 (62.19 to 119.08)	172.74 (125.07 to 230.74)	193.86 (140.78 to 255.54)	154.94 (108.07 to 212.06)	45.8 (33.3 to 59.4)	28.8 (15.6 to 42.7)	76.9 (57.8 to 100.1)
Kuwait	Incidence	299.9 (275.14 to 330.1)	390.91 (356.3 to 433.03)	246.79 (221.65 to 275.91)	449.98 (411.7 to 494.48)	508.26 (465.91 to 553.27)	407.72 (369.46 to 450.71)	50 (38.4 to 62.6)	30 (19.4 to 41.4)	65.2 (48.9 to 82.6)
	Prevalence	8430.94 (7905.48 to 8988.16)	10315.05 (9630.93 to 11052.51)	7177.71 (6660.98 to 7704.96)	10651.88 (9899.15 to 11411.11)	12191.18 (11334.24 to 13047.81)	9520.45 (8847.53 to 10254.27)	26.3 (20.1 to 33.2)	18.2 (11.6 to 25.1)	32.6 (24.6 to 41.4)
	Deaths	27.67 (23.81 to 30.52)	31.47 (24.18 to 36.46)	25.06 (21.94 to 28.48)	16.18 (13.28 to 19.5)	16.54 (12.54 to 21.3)	15.95 (12.8 to 19.61)	-41.5 (-51.2 to -29.1)	-47.4 (-59.2 to -27.5)	-36.4 (-48.3 to -21.4)
	DALYs	646.93 (579.49 to 704.41)	764.3 (630.54 to 860.41)	572.98 (514.77 to 640.69)	418.09 (349.86 to 488.74)	444.11 (364.02 to 541.61)	397.17 (324.96 to 475.24)	-35.4 (-44.2 to -24.8)	-41.9 (-52 to -25.9)	-30.7 (-41.9 to -17.8)
	YLLs	542.12 (473.68 to 594.26)	631.81 (496.38 to 726.48)	486.99 (435.49 to 545.45)	275.73 (229.07 to 332.92)	290.26 (225.76 to 373.15)	265.38 (210.9 to 330.67)	-49.1 (-57.8 to -37.7)	-54.1 (-64.5 to -35.8)	-45.5 (-56.3 to -31.5)
	YLDs	104.81 (76.23 to 139.21)	132.49 (97.47 to 172.3)	85.99 (60.79 to 116.53)	142.36 (101.83 to 190.39)	153.85 (109.47 to 203.15)	131.79 (91.62 to 180.4)	35.8 (21.9 to 50.5)	16.1 (3.4 to 30.9)	53.3 (33.9 to 74.9)

Country	Measure	Age-standardized rate (per 100,000)						% Change (1990 to 2019)		
		1990			2019					
		Both	Female	Male	Both	Female	Male	Both	Female	Male
Lebanon	Incidence	271 (246.74 to 299.53)	323.69 (294.71 to 357.31)	218.38 (197.23 to 240.34)	487.66 (448.31 to 529.31)	519 (476.54 to 560.96)	451.64 (414.16 to 496.42)	79.9 (71.4 to 89.8)	60.3 (51.8 to 70.5)	106.8 (93.8 to 121.4)
	Prevalence	7957.24 (7432.22 to 8474.92)	9167.21 (8545.3 to 9816.26)	6668.1 (6209.66 to 7126.2)	11328.15 (10576.76 to 12055.5)	12323.77 (11550.61 to 13103.54)	10185.04 (9485.57 to 10978.3)	42.4 (37.8 to 47.6)	34.4 (29.2 to 39.7)	52.7 (45.9 to 60.2)
	Deaths	32.44 (27.01 to 39.57)	32.22 (25.57 to 42.66)	32.81 (27.28 to 39.84)	23.32 (16.78 to 30.26)	23.09 (15.76 to 27.85)	23.56 (14.18 to 37.71)	-28.1 (-49.7 to -5.9)	-28.3 (-51 to -7.3)	-28.2 (-57.3 to 13.9)
	DALYs	736.36 (629.66 to 867.2)	763.9 (628.35 to 954.61)	708.38 (594.86 to 846.17)	574.66 (449.11 to 717.08)	579.65 (441.31 to 689.62)	570.73 (404.1 to 847.63)	-22 (-40.1 to -3.2)	-24.1 (-43 to -6.1)	-19.4 (-43.2 to 16.1)
	YLLs	633.61 (532.66 to 762.52)	639.74 (515.46 to 818.15)	628.41 (520.48 to 762.47)	423.15 (307.18 to 555.68)	419.65 (287.52 to 512.82)	427.58 (261.2 to 693.6)	-33.2 (-52.4 to -11.5)	-34.4 (-54.7 to -14.3)	-32 (-57.2 to 7.1)
	YLDs	102.75 (74.87 to 137.29)	124.15 (89.75 to 163.49)	79.97 (56.54 to 108.26)	151.51 (108.62 to 200.59)	160 (115.21 to 210.2)	143.15 (99.22 to 193.05)	47.5 (33.8 to 61.1)	28.9 (15.2 to 44.4)	79 (58.2 to 100.6)
Libya	Incidence	273.41 (249.21 to 301.4)	328.74 (298.59 to 364.87)	225.25 (204.43 to 250)	451.25 (412.76 to 494.26)	497.71 (456.17 to 541.15)	406.76 (369.5 to 450.89)	65 (58.7 to 71.2)	51.4 (44.4 to 59.7)	80.6 (70.6 to 91.2)
	Prevalence	7960.59 (7436.03 to 8527.08)	9302.06 (8678.89 to 9976.09)	6805.68 (6341.74 to 7298.81)	10798.89 (10030.29 to 11535.03)	12121.81 (11253.66 to 12945.54)	9546.75 (8865.35 to 10266.7)	35.7 (32.2 to 39.2)	30.3 (26.3 to 34.9)	40.3 (35.1 to 46)
	Deaths	29.75 (22.44 to 39.08)	31.34 (22.36 to 45.19)	28.43 (20.74 to 38.95)	30.37 (20.49 to 41.27)	33.35 (20.75 to 46.22)	27.42 (18.56 to 43.45)	2.1 (-25.3 to 34.9)	6.4 (-26.5 to 42.8)	-3.6 (-32.5 to 40.8)
	DALYs	717.84 (571.93 to 880.67)	800.92 (617.69 to 1021.08)	647.4 (497.65 to 839.46)	755.7 (552.53 to 970.69)	847.51 (581.04 to 1100.49)	667.41 (494.61 to 970.9)	5.3 (-18.2 to 33.6)	5.8 (-21.9 to 35.6)	3.1 (-23.1 to 42.4)
	YLLs	613.06 (469.18 to 770.83)	670.21 (488.24 to 880.33)	565.1 (423.63 to 753.99)	599.98 (403.11 to 810.46)	673.28 (417.27 to 921.58)	529.06 (362.2 to 824.43)	-2.1 (-29 to 31.4)	0.5 (-31.5 to 35.7)	-6.4 (-35.4 to 36.8)
	YLDs	104.77 (76.16 to 139.11)	130.71 (95.11 to 173.47)	82.3 (58.05 to 111.01)	155.72 (113.5 to 207.01)	174.23 (125.61 to 231.29)	138.35 (98.33 to 188.99)	48.6 (36.6 to 61.5)	33.3 (19.1 to 47.7)	68.1 (50 to 88.2)

Country	Measure	Age-standardized rate (per 100,000)						% Change (1990 to 2019)		
		1990			2019					
		Both	Female	Male	Both	Female	Male	Both	Female	Male
Morocco	Incidence	212.54 (192.23 to 234.94)	251.21 (224.98 to 280.2)	173.25 (156.53 to 191.24)	436.35 (400.91 to 477.28)	467.51 (427 to 514.08)	405.57 (368.35 to 445.87)	105.3 (97.6 to 114.1)	86.1 (76.3 to 96.2)	134.1 (122 to 148.3)
	Prevalence	6908.18 (6411.46 to 7364.2)	7915.94 (7308.91 to 8487.98)	5847.23 (5431.36 to 6252.48)	10481.87 (9787.98 to 11222.33)	11535.6 (10781.41 to 12369.5)	9411.19 (8757.7 to 10103.2)	51.7 (47.2 to 57)	45.7 (40.5 to 51.4)	61 (54.4 to 68.4)
	Deaths	29.43 (24.56 to 41.35)	27.84 (22.23 to 43.32)	31.67 (24.67 to 45.26)	35.84 (27.87 to 46.25)	36.33 (27.35 to 49.01)	35.42 (25.11 to 50.18)	21.8 (-8.9 to 51.6)	30.5 (-9.2 to 71.2)	11.9 (-18 to 48.9)
	DALYs	705.98 (615.6 to 865.71)	721.86 (603.02 to 936.44)	693.7 (567.19 to 885.43)	844.57 (673.16 to 1035.89)	899.73 (705.6 to 1101.66)	790.62 (593.63 to 1025.34)	19.6 (-7 to 44.6)	24.6 (-8.6 to 58.6)	14 (-12.8 to 43.6)
	YLLs	617.54 (531.48 to 776.4)	613.4 (495.98 to 828.08)	626.16 (502.72 to 816.28)	679.42 (523.68 to 859.06)	718.49 (530.49 to 906.35)	641.6 (450.53 to 874.43)	10 (-18 to 37.8)	17.1 (-20.1 to 56.5)	2.5 (-25.8 to 34.1)
	YLDs	88.44 (63.93 to 117.39)	108.46 (78.96 to 141.38)	67.54 (47.95 to 91.12)	165.15 (119.26 to 219.06)	181.24 (131.11 to 239.64)	149.02 (104.78 to 203.66)	86.7 (70.9 to 102.3)	67.1 (49.9 to 84.7)	120.6 (96.7 to 148.1)
Oman	Incidence	226.36 (204.92 to 250.11)	268.25 (240.97 to 299.78)	191.55 (172.49 to 212.58)	442.9 (405.04 to 481.49)	480.84 (438.17 to 523.05)	416.69 (378.64 to 455.2)	95.7 (86.4 to 105.2)	79.3 (68.4 to 91.6)	117.5 (102.7 to 131.9)
	Prevalence	7111.12 (6611.3 to 7585.48)	8252.7 (7633.31 to 8865.3)	6223.03 (5771.89 to 6657.73)	10466.55 (9764.79 to 11155.6)	11766.8 (10953.16 to 12563.18)	9659.66 (9009.39 to 10336.31)	47.2 (42.4 to 52.2)	42.6 (36.8 to 48.7)	55.2 (48.6 to 62.6)
	Deaths	16.82 (12.93 to 22.29)	16.19 (12.38 to 21.6)	18.93 (14.09 to 26.87)	19.85 (17.05 to 22.82)	19.4 (15.59 to 23.23)	21.39 (17.41 to 25.95)	18 (-15.1 to 58.5)	19.8 (-17.5 to 62.4)	13 (-28.3 to 65.7)
	DALYs	413.4 (332.63 to 518.6)	443.83 (356.07 to 561.18)	402.04 (311 to 542.65)	491.3 (428.61 to 558.94)	533.29 (452.53 to 617.89)	471.98 (394.23 to 554.34)	18.8 (-6.6 to 46.5)	20.2 (-6 to 47.7)	17.4 (-16.3 to 56.6)
	YLLs	311.33 (240.71 to 412.95)	317.24 (239.53 to 424.75)	321.5 (236.91 to 463.26)	314.66 (271.94 to 362.03)	330.77 (269.25 to 393.85)	313.48 (256.39 to 382.85)	1.1 (-26.3 to 35.9)	4.3 (-25.9 to 42.1)	-2.5 (-35.4 to 43.3)
	YLDs	102.06 (73.96 to 134.48)	126.59 (92.27 to 164.9)	80.54 (58.41 to 109.06)	176.64 (130.15 to 234.06)	202.52 (149.24 to 267.46)	158.5 (113.79 to 215.17)	73.1 (59.2 to 87.9)	60 (46.1 to 75)	96.8 (73.8 to 120.3)

Country	Measure	Age-standardized rate (per 100,000)						% Change (1990 to 2019)		
		1990			2019					
		Both	Female	Male	Both	Female	Male	Both	Female	Male
Palestine	Incidence	308.03 (282.16 to 336.99)	345.41 (313.26 to 381.59)	263.36 (239.16 to 287.05)	474.96 (436.76 to 517.17)	498.95 (457.7 to 546.07)	452.51 (411.23 to 495.78)	54.2 (48.1 to 61.1)	44.5 (36.4 to 53.5)	71.8 (59.7 to 83)
	Prevalence	8480.38 (7902.96 to 9037.79)	9488.56 (8790.4 to 10137.35)	7296.42 (6799.31 to 7813.66)	11080.75 (10377.76 to 11858.57)	11944.25 (11196.22 to 12806.11)	10185.38 (9456.63 to 10934.39)	30.7 (27 to 34.6)	25.9 (20.9 to 31)	39.6 (33 to 45.8)
	Deaths	46.81 (37.83 to 57.37)	43.62 (34.04 to 54.27)	51.65 (41.71 to 64.57)	33.58 (28.52 to 38.58)	30.82 (25.89 to 35.97)	38.74 (32.02 to 45.18)	-28.3 (-44.8 to -8.8)	-29.3 (-45.7 to -7.2)	-25 (-44.2 to -2.9)
	DALYs	1093.45 (884.26 to 1330.85)	1082.49 (863.87 to 1325.44)	1119.79 (892.66 to 1396.26)	809.92 (707.33 to 918.09)	775.59 (672.27 to 886.64)	862.71 (741.53 to 999.46)	-25.9 (-40.9 to -7.8)	-28.4 (-42.7 to -9.9)	-23 (-39.7 to -1.9)
	YLLs	970.46 (768.31 to 1204.23)	939.31 (732.57 to 1172.3)	1019.37 (797.92 to 1297.23)	641.43 (547.17 to 737.03)	597.68 (504.98 to 697.02)	703.58 (589.92 to 823.15)	-33.9 (-48.9 to -14.5)	-36.4 (-51 to -16.1)	-31 (-48 to -9.1)
	YLDs	122.99 (90.23 to 161.34)	143.18 (104.53 to 188.61)	100.43 (71.91 to 132.64)	168.49 (124.52 to 224.13)	177.91 (131.68 to 232.15)	159.13 (112.71 to 219)	37 (26.1 to 49.1)	24.3 (11.7 to 37.6)	58.5 (41 to 78.3)
Qatar	Incidence	318.15 (287.27 to 350.25)	420.29 (378.22 to 463.63)	271.38 (243.99 to 300.87)	506.35 (463.47 to 551.97)	568.41 (521.17 to 615.44)	486.64 (443.76 to 534.31)	59.2 (52.2 to 66.4)	35.2 (27.6 to 43.2)	79.3 (69.6 to 89.7)
	Prevalence	8537.86 (7938.22 to 9148.05)	10544.21 (9754.68 to 11373.63)	7345.95 (6817.93 to 7870.31)	11255.77 (10468.36 to 12087.69)	13125.03 (12234.22 to 14054.66)	10650.73 (9874.45 to 11466.08)	31.8 (27.1 to 36.2)	24.5 (19.3 to 29.4)	45 (38.8 to 50.9)
	Deaths	48.93 (38.41 to 84.35)	55.99 (40.49 to 107.59)	44.24 (32.81 to 65.69)	45.88 (37.09 to 55.98)	71.84 (57.57 to 87.5)	37.72 (29.17 to 47.5)	-6.2 (-41.8 to 28.1)	28.3 (-32.2 to 81.9)	-14.7 (-41.5 to 20.2)
	DALYs	927.8 (751.55 to 1439.48)	1133.41 (864.84 to 1959.99)	809.76 (625.87 to 1128.64)	803.55 (662.78 to 963.84)	1190.82 (971 to 1450.53)	675.84 (542.89 to 838.27)	-13.4 (-40.4 to 12)	5.1 (-36.6 to 40.3)	-16.5 (-38.9 to 11.6)
	YLLs	807.02 (639.04 to 1312.05)	976.96 (711.85 to 1797.81)	710.22 (529.27 to 1010.35)	629.65 (503.54 to 778.06)	996.67 (791.52 to 1246.46)	508.15 (385.9 to 654.97)	-22 (-48.8 to 5.9)	2 (-42.5 to 44.4)	-28.5 (-49.9 to 1.4)
	YLDs	120.78 (87.34 to 163.55)	156.46 (114.2 to 206.57)	99.54 (70.46 to 135.96)	173.9 (124.72 to 233.62)	194.15 (141.36 to 249.46)	167.69 (115.98 to 229.32)	44 (28.6 to 59.7)	24.1 (10.5 to 38.3)	68.5 (49.4 to 90)

Country	Measure	Age-standardized rate (per 100,000)						% Change (1990 to 2019)		
		1990			2019					
		Both	Female	Male	Both	Female	Male	Both	Female	Male
Saudi Arabia	Incidence	307.34 (282.74 to 334.59)	376.7 (341.73 to 413.76)	259.55 (235.46 to 284.29)	561.38 (524.55 to 598.58)	606.66 (563.24 to 651.09)	531.48 (491.82 to 570.49)	82.7 (73.9 to 92.1)	61 (50.5 to 72.6)	104.8 (91.8 to 117.7)
	Prevalence	8298.56 (7741.28 to 8826.33)	9905.98 (9194.53 to 10582.71)	7142.02 (6660.18 to 7634.98)	12520.39 (11768.47 to 13282.93)	14082.72 (13148.43 to 14997.17)	11489.76 (10721.25 to 12224.02)	50.9 (45.3 to 56.9)	42.2 (34.9 to 50.3)	60.9 (53.5 to 68.9)
	Deaths	47.9 (37.69 to 59.35)	43.52 (34.57 to 54.38)	52.44 (39.19 to 67.19)	52.88 (42.82 to 63.11)	53.46 (40.25 to 68.07)	52.74 (42.43 to 64.57)	10.4 (-20.8 to 50.2)	22.8 (-18 to 69.8)	0.6 (-33.1 to 47.6)
	DALYs	1106.77 (880.73 to 1366.69)	1095.78 (868.41 to 1360.26)	1128.82 (860.96 to 1447.48)	1197.72 (982.79 to 1421.32)	1272.11 (1002.17 to 1585.51)	1149.35 (931.21 to 1396.48)	8.2 (-20.7 to 42.6)	16.1 (-18.6 to 56.7)	1.8 (-29.9 to 45.6)
	YLLs	988.84 (767.81 to 1249.49)	949.9 (739.59 to 1206.31)	1031.16 (761.99 to 1341.38)	1003.48 (798.39 to 1220.61)	1059.57 (802.41 to 1361.19)	967.77 (765.97 to 1217.04)	1.5 (-28.7 to 39.7)	11.5 (-26.3 to 58.6)	-6.1 (-38 to 41.3)
	YLDs	117.93 (85.61 to 153.76)	145.88 (106.3 to 189.79)	97.66 (70.59 to 129.1)	194.24 (140.77 to 258.26)	212.54 (157.51 to 281.64)	181.57 (126.91 to 245.8)	64.7 (49.7 to 81.6)	45.7 (29.7 to 64)	85.9 (64.8 to 109.2)
Sudan	Incidence	209.29 (190.01 to 230.8)	248.83 (224.35 to 277.39)	173.79 (157.86 to 191.19)	374.99 (339.59 to 413.83)	409.31 (370.43 to 448.21)	346.13 (312.41 to 384.44)	79.2 (72.4 to 86.1)	64.5 (56.2 to 72.4)	99.2 (88.3 to 110.5)
	Prevalence	6814.9 (6333.45 to 7291.31)	7796.87 (7240.35 to 8370.27)	5857.18 (5423.36 to 6284.88)	9400.88 (8723.18 to 10074.08)	10410.69 (9682.64 to 11166)	8463.02 (7826.53 to 9100.39)	37.9 (33.6 to 42.1)	33.5 (28.8 to 38.6)	44.5 (38.5 to 50.6)
	Deaths	28.53 (22.13 to 40.32)	26.84 (19.84 to 40.65)	30.38 (23.23 to 45.46)	28.39 (20.33 to 44.3)	27.37 (17.98 to 40.7)	29.35 (19.11 to 51.75)	-0.5 (-33.1 to 45.8)	2 (-40.6 to 41.1)	-3.4 (-35.9 to 60.7)
	DALYs	737.96 (596.02 to 942.15)	760.2 (580.18 to 1037.6)	717.11 (571.7 to 950.38)	717.3 (535.03 to 1013.09)	729.69 (534.33 to 974.25)	704.4 (494.21 to 1139.19)	-2.8 (-31.4 to 36.2)	-4 (-37.9 to 28.1)	-1.8 (-30.6 to 54.7)
	YLLs	653.8 (517.32 to 856.39)	656.86 (484.79 to 927.95)	651.82 (503.96 to 882.34)	579.67 (409.08 to 865.65)	575.21 (379.31 to 812.63)	583.21 (379.8 to 1005.36)	-11.3 (-41.1 to 32)	-12.4 (-49 to 23.4)	-10.5 (-40.9 to 51.7)
	YLDs	84.17 (61.26 to 111.66)	103.34 (74.36 to 139.33)	65.29 (47.12 to 87.52)	137.64 (100.02 to 183.35)	154.48 (112.6 to 201.76)	121.19 (86.66 to 164.87)	63.5 (51.2 to 77.2)	49.5 (35.4 to 64.9)	85.6 (65.4 to 108.6)

Country	Measure	Age-standardized rate (per 100,000)						% Change (1990 to 2019)		
		1990			2019					
		Both	Female	Male	Both	Female	Male	Both	Female	Male
Syrian Arab Republic	Incidence	278.72 (254.79 to 307.18)	342.05 (310.94 to 378.53)	221.66 (201.2 to 244.78)	442.35 (409.46 to 480.45)	490.33 (452.33 to 533.32)	394.23 (361.51 to 434.32)	58.7 (49.6 to 67.1)	43.3 (34.4 to 52.2)	77.9 (62.8 to 93.3)
	Prevalence	8003.11 (7468.89 to 8515.47)	9365.68 (8714.16 to 10045.6)	6747.59 (6294.58 to 7214.17)	10470.89 (9813.87 to 11159.25)	11669.82 (10855.49 to 12511.55)	9275.91 (8649 to 9921.45)	30.8 (26.2 to 35.5)	24.6 (19.2 to 29.9)	37.5 (30.2 to 45.3)
	Deaths	36.99 (30.44 to 46.13)	37.35 (30.21 to 47.54)	36.68 (28.93 to 48.74)	27.78 (22.07 to 34.58)	31.82 (25.08 to 39.26)	25.69 (19.56 to 32.74)	-24.9 (-46.2 to 1.1)	-14.8 (-39.5 to 14.8)	-30 (-53.6 to 0.6)
	DALYs	955.07 (803.08 to 1143.63)	979.6 (803.09 to 1178.59)	931.7 (761.66 to 1132.95)	684.86 (556.83 to 836.76)	737.17 (600.46 to 900.72)	646.08 (511.17 to 805.61)	-28.3 (-44.4 to -6.3)	-24.7 (-42.3 to -1.2)	-30.7 (-49.3 to -5.1)
	YLLs	837.42 (689.93 to 1021.58)	833.15 (668.23 to 1028.91)	840.81 (674.3 to 1046.97)	535.35 (417.39 to 690.96)	571.78 (437.11 to 743.74)	513.55 (387.2 to 666.11)	-36.1 (-53.5 to -11.5)	-31.4 (-51.1 to -3.7)	-38.9 (-57.9 to -11.8)
	YLDs	117.65 (85.55 to 155.17)	146.45 (107.43 to 192.16)	90.89 (65.11 to 121.49)	149.5 (108.97 to 196.71)	165.38 (120.5 to 216.32)	132.53 (94.4 to 177.47)	27.1 (16.1 to 39.9)	12.9 (1.3 to 26.4)	45.8 (28.1 to 65.3)
Tunisia	Incidence	257.83 (234.41 to 283.58)	305.98 (276.32 to 336.23)	213.24 (192.8 to 236.27)	452.53 (414.56 to 489.31)	487.11 (446.19 to 527.84)	417.93 (380.49 to 454.34)	75.5 (67.6 to 83.4)	59.2 (50.2 to 69)	96 (84.3 to 108.4)
	Prevalence	7543.05 (7036.1 to 8018.23)	8670.61 (8094.17 to 9235.35)	6435.58 (5967.6 to 6880.76)	10559.96 (9861.45 to 11220.71)	11568.43 (10751.76 to 12333.84)	9500.46 (8849.57 to 10177.38)	40 (35.9 to 44.4)	33.4 (28.4 to 38.8)	47.6 (41.7 to 54.5)
	Deaths	24.28 (20.58 to 29.37)	23.46 (19.56 to 31.69)	25.44 (20.45 to 31.17)	23.62 (16.87 to 31.07)	22.93 (15.8 to 30.82)	24.51 (16.81 to 33.9)	-2.7 (-31.9 to 31.3)	-2.2 (-33.1 to 34.8)	-3.6 (-36.6 to 37.5)
	DALYs	577.18 (504.01 to 666.43)	594.25 (511.67 to 737.4)	562.49 (475.99 to 665.44)	568.57 (441 to 718.86)	561.59 (430.51 to 712.98)	577.24 (440.36 to 756.71)	-1.5 (-25 to 25.5)	-5.5 (-28 to 21.5)	2.6 (-24.8 to 37.5)
	YLLs	483.01 (414.93 to 566.7)	487.13 (412.18 to 624.39)	481.25 (395.51 to 586.2)	426.12 (304.37 to 571.15)	416.54 (290.75 to 559.41)	437.3 (305.53 to 614.63)	-11.8 (-38.7 to 20.6)	-14.5 (-41.9 to 17.4)	-9.1 (-38.6 to 30.5)
	YLDs	94.17 (68.24 to 124.38)	107.12 (77.38 to 141.62)	81.24 (58.22 to 108.34)	142.45 (103.66 to 189.24)	145.05 (106.5 to 189.87)	139.94 (99.05 to 190.64)	51.3 (38.7 to 65.2)	35.4 (21.5 to 51.1)	72.3 (54.5 to 92.9)

Country	Measure	Age-standardized rate (per 100,000)						% Change (1990 to 2019)		
		1990			2019					
		Both	Female	Male	Both	Female	Male	Both	Female	Male
Turkey	Incidence	228.86 (212.13 to 247.99)	248.94 (230.08 to 273.35)	206.86 (191.62 to 222.78)	432.17 (396.21 to 469.84)	449.78 (411.8 to 488.13)	413.1 (377.7 to 456.53)	88.8 (75.9 to 102.3)	80.7 (67 to 96.1)	99.7 (83.9 to 118.7)
	Prevalence	7115.45 (6664.07 to 7598.65)	7832.68 (7315.65 to 8402.25)	6360.73 (5951.91 to 6779.45)	10097.36 (9441.69 to 10766.51)	10811.69 (10102.45 to 11528.07)	9355.9 (8690.02 to 10080.48)	41.9 (35.8 to 48.8)	38 (31.5 to 46)	47.1 (39.1 to 56.7)
	Deaths	35.19 (28.26 to 50.25)	30.58 (24.27 to 46.45)	40.9 (30.66 to 60.62)	23.48 (18.99 to 28.71)	22.58 (17.98 to 27.85)	24.52 (19.55 to 30.12)	-33.3 (-56.2 to -9.6)	-26.2 (-54.3 to -0.1)	-40 (-62.7 to -11.3)
	DALYs	853.91 (725.99 to 1083.24)	778.1 (649.38 to 1036.9)	941.5 (750.56 to 1240.26)	562.02 (473.61 to 667.56)	540.16 (453.4 to 645.14)	585.97 (486.63 to 698.57)	-34.2 (-51.3 to -17.5)	-30.6 (-50.3 to -12.2)	-37.8 (-55.9 to -16.3)
	YLLs	758.74 (631.14 to 987.24)	672.9 (551.12 to 930.57)	856.75 (672.39 to 1151.1)	420.26 (341.3 to 513.64)	394.86 (316.41 to 488.27)	446.9 (357.01 to 553.39)	-44.6 (-60.6 to -27)	-41.3 (-60.2 to -21.8)	-47.8 (-65 to -26.7)
	YLDs	95.17 (69.92 to 125.59)	105.2 (77.73 to 139.64)	84.75 (61.59 to 112.03)	141.76 (102.53 to 187.18)	145.3 (106.42 to 191.96)	139.07 (97.04 to 187.52)	48.9 (35.5 to 64.1)	38.1 (23.4 to 56.8)	64.1 (44 to 84.8)
United Arab Emirates	Incidence	344.25 (313.56 to 375.57)	419.73 (381.71 to 461.16)	299.48 (271.65 to 328.37)	516.47 (476.76 to 558.41)	586.01 (543.59 to 630.16)	492.96 (452.28 to 535.53)	50 (44.1 to 56.2)	39.6 (33.1 to 46.2)	64.6 (56 to 72.8)
	Prevalence	8827.32 (8219.28 to 9454.45)	10597.97 (9810.55 to 11409.17)	7833.23 (7307.88 to 8386.24)	11504.82 (10751.58 to 12295.57)	13296.62 (12438.69 to 14189.47)	10816.03 (10062.93 to 11634.87)	30.3 (26.6 to 34.1)	25.5 (21.1 to 29.8)	38.1 (32.4 to 43.9)
	Deaths	61.23 (37.47 to 75.7)	63.31 (38.26 to 86.68)	59.96 (35.33 to 78.07)	48.09 (29.07 to 73.62)	52.13 (25.95 to 70.12)	46.41 (28.55 to 80.16)	-21.5 (-46.6 to 11.6)	-17.7 (-51.6 to 19.6)	-22.6 (-47.6 to 21.2)
	DALYs	1343.16 (954.68 to 1636.83)	1400.05 (959.99 to 1849.67)	1312.1 (880.12 to 1667.57)	1136.93 (787.65 to 1645.06)	1207.92 (757.01 to 1568.74)	1108.08 (756.85 to 1769.29)	-15.4 (-40.1 to 17.9)	-13.7 (-46.2 to 20.1)	-15.5 (-40.1 to 26.9)
	YLLs	1212.49 (823.9 to 1503.44)	1238.24 (801.13 to 1686.89)	1199.1 (756.64 to 1564.44)	952.77 (612.57 to 1458.93)	1006.82 (573.65 to 1378.52)	930.98 (594.53 to 1595.4)	-21.4 (-47.3 to 14.1)	-18.7 (-53.5 to 19.6)	-22.4 (-48.4 to 23.6)
	YLDs	130.67 (94.9 to 173.21)	161.81 (118.39 to 210.68)	113.01 (81.4 to 154.31)	184.16 (132.98 to 246.9)	201.1 (148.37 to 264.93)	177.1 (124.85 to 242.72)	40.9 (28.8 to 54.1)	24.3 (12.6 to 37.3)	56.7 (40.6 to 75.2)

Country	Measure	Age-standardized rate (per 100,000)						% Change (1990 to 2019)		
		1990			2019					
		Both	Female	Male	Both	Female	Male	Both	Female	Male
Yemen	Incidence	207.26 (188.54 to 229.36)	237.33 (213.24 to 266.38)	174.15 (157.77 to 192.85)	348.08 (311.61 to 388.8)	379.3 (333.6 to 440.32)	316.79 (284.97 to 355.15)	67.9 (58.3 to 82.2)	59.8 (45.5 to 83.6)	81.9 (71.3 to 91.9)
	Prevalence	6688.89 (6206.4 to 7150.42)	7503.11 (6932.81 to 8049.71)	5750.88 (5353.87 to 6160.9)	8814.93 (8124.86 to 9490.76)	9746.42 (8925.3 to 10754.6)	7860.22 (7244.6 to 8476.38)	31.8 (26.4 to 39.2)	29.9 (22.8 to 42)	36.7 (30.9 to 42.8)
	Deaths	24.8 (18.61 to 34.23)	22.14 (15.83 to 34.53)	29.64 (21.86 to 42.07)	23.19 (18.02 to 30.53)	20.97 (15.95 to 28.93)	25.74 (19.02 to 35.29)	-6.5 (-28.5 to 22.8)	-5.3 (-32.5 to 31.5)	-13.2 (-34.2 to 17.4)
	DALYs	641.24 (494.49 to 835.55)	618.94 (457.25 to 906.13)	682.65 (515.54 to 913.62)	611.56 (486.8 to 769.32)	604.25 (477.51 to 798.39)	621.25 (480.76 to 794.18)	-4.6 (-26.1 to 22.9)	-2.4 (-28.8 to 28.3)	-9 (-30.5 to 21.9)
	YLLs	553.4 (411.93 to 747.83)	513.36 (358.1 to 793.4)	615.14 (454.21 to 841.31)	476.57 (362.33 to 632.43)	446.08 (331.55 to 628.83)	509.7 (377.44 to 686.77)	-13.9 (-36.1 to 15.9)	-13.1 (-38.4 to 20.8)	-17.1 (-39.1 to 15.4)
	YLDs	87.84 (64.03 to 115.54)	105.58 (76.95 to 140.5)	67.51 (47.17 to 90.19)	134.99 (97.73 to 177.95)	158.18 (112.84 to 212.84)	111.55 (79.99 to 152.57)	53.7 (39.6 to 71)	49.8 (33 to 76.2)	65.2 (48.1 to 83.9)

Data in parentheses are 95% Uncertainty Intervals (95% UIs)
