# Supplementary material for: Regional burden of chronic kidney disease in North Africa and Middle East during 1990–2019; Results from Global Burden of Disease study 2019
Source: Front Public Health. 2022 Oct 11;10:1015902. doi: 10.3389/fpubh.2022.1015902 (PMC9592811; doi:10.3389/fpubh.2022.1015902)
Supplement: Supplementary file 6 [file Data_Sheet_6.PDF]

Cause	Location	Measure	Age-standardized rate (per 100,000)						% Change (1990 to 2019)		
			1990			2019					
			Both	Female	Male	Both	Female	Male	Both	Female	Male
Chronic kidney disease due to diabetes mellitus type 1	North Africa and Middle East	Incidence	1.6 (1.3 to 2)	1.3 (1 to 1.7)	1.8 (1.5 to 2.3)	2.9 (2.3 to 3.5)	2.2 (1.8 to 2.9)	3.4 (2.8 to 4.2)	82.2 (66.6 to 98.8)	76.1 (58.7 to 94.6)	85.5 (69.1 to 103.4)
		Prevalence	48 (41.1 to 56.7)	47.8 (40.9 to 57.9)	48.2 (41.1 to 57.3)	79.3 (67.8 to 94.6)	81.4 (68.2 to 99)	77.6 (66.3 to 92.3)	65.2 (52.8 to 78.1)	70.2 (55.6 to 86.7)	61.1 (47.5 to 73.7)
		Deaths	1.1 (0.7 to 1.6)	1 (0.6 to 1.4)	1.2 (0.7 to 1.8)	0.9 (0.6 to 1.4)	0.8 (0.5 to 1.2)	1 (0.6 to 1.5)	-17 (-31.7 to 0.3)	-15.8 (-35.4 to 2)	-18.3 (-35.3 to 5.3)
		DALYs	39.3 (25.9 to 55.6)	37.1 (24.5 to 53.1)	41.5 (27 to 59.7)	34 (22.3 to 49.5)	32 (20.8 to 46.8)	35.9 (23.2 to 53.4)	-13.5 (-27 to 2.3)	-13.8 (-31 to 3.5)	-13.3 (-28.4 to 8.2)
		YLLs	37 (23.8 to 53.2)	35 (22.7 to 50.6)	38.9 (24.9 to 56.9)	30 (18.9 to 45.1)	28.4 (17.5 to 42.3)	31.6 (19.4 to 48.8)	-18.8 (-32.3 to -2)	-18.9 (-37 to -1.3)	-18.8 (-34.4 to 4)
		YLDs	2.3 (1.6 to 3.2)	2.1 (1.5 to 3)	2.5 (1.7 to 3.5)	4 (2.6 to 5.6)	3.6 (2.4 to 5.1)	4.3 (2.8 to 6)	69.5 (53.3 to 86.8)	68.8 (49.8 to 89.9)	70.1 (53.5 to 88.5)
	Afghanistan	Incidence	1.3 (0.6 to 2.5)	1.1 (0.4 to 2.6)	1.4 (0.7 to 2.7)	2 (0.9 to 3.9)	1.7 (0.6 to 3.9)	2.3 (1.1 to 4.4)	58.1 (46.4 to 73.5)	49.4 (37.9 to 64.5)	63.2 (47.3 to 84.2)
		Prevalence	34.6 (22.4 to 51.8)	33.7 (20.2 to 53.5)	35.7 (22.3 to 54.6)	51.4 (32.5 to 79.7)	50 (28.6 to 82.4)	53.1 (32.6 to 83.2)	48.7 (33.2 to 64.6)	48.4 (32.1 to 66.2)	48.8 (29.6 to 69.1)
		Deaths	2.3 (1.4 to 3.7)	2.3 (1.4 to 3.9)	2.2 (1.2 to 3.6)	1.9 (1.1 to 3.1)	2 (1.1 to 3.5)	1.8 (1 to 2.9)	-17.9 (-42.7 to 12.6)	-15.2 (-47.5 to 24.4)	-16.8 (-42.9 to 16.9)
		DALYs	86.8 (51.3 to 138.4)	94.2 (53.4 to 158.5)	75.8 (42.8 to 120.7)	70.9 (43.1 to 116.2)	78.5 (45 to 139.4)	64.3 (36.2 to 104.2)	-18.3 (-43.2 to 14.9)	-16.7 (-47.8 to 24.4)	-15.2 (-41.8 to 23.9)
		YLLs	84.9 (49.7 to 136.7)	92.4 (51.8 to 157.1)	73.8 (41.7 to 118.6)	68.1 (40.5 to 112.6)	75.8 (42 to 136.8)	61.3 (34.1 to 100.7)	-19.8 (-45 to 14.1)	-18 (-49.4 to 23.1)	-17 (-44.1 to 22.6)
		YLDs	1.9 (1.1 to 3.1)	1.8 (1 to 3.1)	2 (1.2 to 3.1)	2.8 (1.6 to 4.8)	2.6 (1.4 to 5)	3 (1.7 to 5)	49.9 (29.4 to 69.1)	49.3 (24.6 to 71.3)	51.5 (28.7 to 74.2)

Cause	Location	Measure	Age-standardized rate (per 100,000)						% Change (1990 to 2019)		
			1990			2019					
			Both	Female	Male	Both	Female	Male	Both	Female	Male
	Algeria	Incidence	1.4 (0.6 to 3)	1.2 (0.5 to 2.9)	1.5 (0.7 to 3.1)	2.6 (1.3 to 5.1)	2.1 (0.9 to 4.7)	3 (1.5 to 5.7)	87 (66.5 to 123.8)	73.6 (56.2 to 100.3)	96.9 (70.3 to 142.1)
		Prevalence	39.7 (25.2 to 65.4)	41.1 (24.4 to 67.7)	38.4 (22.6 to 64.5)	70.5 (45.3 to 112.2)	72.1 (43.5 to 117.9)	68.9 (42 to 114)	77.6 (62.8 to 94.1)	75.4 (57.5 to 95)	79.5 (61.4 to 100.5)
		Deaths	1.1 (0.6 to 1.8)	1.1 (0.6 to 2.1)	1 (0.6 to 1.7)	0.8 (0.5 to 1.2)	0.9 (0.5 to 1.4)	0.7 (0.4 to 1.2)	-27 (-47.3 to 0.1)	-23.9 (-47 to 8.6)	-30.5 (-52.9 to 1.3)
		DALYs	39.3 (23.6 to 64.2)	42.9 (24.5 to 78)	35.9 (21.8 to 55.6)	29.9 (19 to 45.4)	32.9 (20.3 to 52.1)	26.9 (16.7 to 42.5)	-24 (-45.1 to 4.8)	-23.4 (-47.4 to 9.2)	-25 (-49.3 to 8)
		YLLs	37.6 (22.2 to 62.4)	41.2 (23 to 76)	34.1 (20 to 53)	26.6 (15.9 to 41.7)	29.8 (17.2 to 48.2)	23.4 (13.4 to 38.4)	-29.3 (-50.1 to -0.8)	-27.8 (-50.7 to 5)	-31.4 (-54.6 to 2.4)
		YLDs	1.8 (1 to 2.8)	1.7 (1 to 2.9)	1.8 (1.1 to 3.1)	3.3 (2 to 5.5)	3.1 (1.7 to 5.5)	3.6 (2 to 5.9)	90.2 (65.1 to 115.6)	83.6 (53.7 to 114.4)	95 (65.9 to 124.2)
	Bahrain	Incidence	1.2 (0.5 to 2.4)	1 (0.4 to 2.4)	1.3 (0.6 to 2.6)	2.3 (1.1 to 4.3)	1.8 (0.7 to 4.1)	2.6 (1.2 to 4.9)	92.6 (71.9 to 130.1)	74.8 (57.5 to 98.8)	99.6 (76.8 to 139.6)
		Prevalence	36.3 (23.5 to 57.8)	38.8 (23.5 to 65.6)	34.9 (21.4 to 55.4)	60.5 (39.1 to 96.9)	64.1 (39.3 to 107.8)	58.4 (35.8 to 94.3)	66.5 (51.6 to 83.5)	65.2 (49 to 83.6)	67.3 (47.9 to 89)
		Deaths	0.9 (0.5 to 1.4)	0.9 (0.5 to 1.4)	0.9 (0.6 to 1.6)	0.7 (0.4 to 1.1)	0.6 (0.4 to 1)	0.8 (0.5 to 1.2)	-21.3 (-40.4 to 1.8)	-26.6 (-47.7 to 1.3)	-19.3 (-39.4 to 7.7)
		DALYs	27.6 (17.3 to 41.9)	27.5 (16.9 to 42.5)	28.1 (17.3 to 45.7)	22.8 (15 to 33.6)	20.7 (13.5 to 30.5)	24.1 (15.7 to 36.2)	-17.6 (-36.3 to 5.8)	-24.7 (-45.7 to 2)	-13.9 (-35.2 to 13.6)
		YLLs	25.7 (15.7 to 39.9)	25.6 (15.1 to 40.4)	26 (15.5 to 43.5)	19.5 (12.3 to 29.8)	17.7 (10.7 to 27.4)	20.7 (12.9 to 32.5)	-24.1 (-42.7 to -1.5)	-30.9 (-50.9 to -3.7)	-20.5 (-42 to 7.8)
		YLDs	2 (1.2 to 3.1)	1.9 (1.1 to 3.1)	2 (1.2 to 3.2)	3.3 (1.9 to 5.3)	3 (1.7 to 5.2)	3.4 (2 to 5.7)	67.3 (42.9 to 93.4)	60.2 (31.9 to 92.2)	70.1 (43.6 to 99.1)

Cause	Location	Measure	Age-standardized rate (per 100,000)						% Change (1990 to 2019)		
			1990			2019					
			Both	Female	Male	Both	Female	Male	Both	Female	Male
	Egypt	Incidence	1.9 (1.1 to 3.3)	1.5 (0.7 to 3)	2.2 (1.3 to 3.8)	3 (1.7 to 5.3)	2.4 (1.1 to 4.7)	3.5 (2 to 6.3)	59.5 (44.3 to 75)	60 (43.4 to 78.8)	56.6 (37.8 to 75.3)
		Prevalence	59.3 (41.5 to 84.6)	58.2 (37.8 to 88.3)	60.5 (41 to 88.9)	82.8 (56.5 to 123.1)	87.7 (55.1 to 134.9)	77.5 (50.8 to 120.7)	39.7 (22.3 to 58)	50.7 (32.1 to 71.5)	28.2 (8.5 to 50.4)
		Deaths	1.1 (0.6 to 1.7)	1.1 (0.5 to 1.8)	1.1 (0.6 to 1.7)	1.2 (0.6 to 2.1)	1.2 (0.5 to 2.3)	1.2 (0.6 to 2.3)	12.7 (-22.7 to 51.7)	12.7 (-27.4 to 55.5)	14.3 (-27.1 to 62.1)
		DALYs	38.4 (23.6 to 57.6)	39.9 (20.3 to 61.9)	37 (22.4 to 58.3)	42.8 (23 to 72.6)	42.5 (18.7 to 73.7)	43.1 (23.3 to 77.5)	11.4 (-20.2 to 48.2)	6.6 (-27.5 to 46)	16.6 (-21.6 to 60.3)
		YLLs	35.4 (20.9 to 54)	37.2 (17.8 to 59.3)	33.7 (19.3 to 54.2)	38.2 (18.9 to 67.5)	38.3 (14.7 to 69.6)	38.2 (19 to 72.4)	7.8 (-26.1 to 47)	2.9 (-34.8 to 45)	13.6 (-29.2 to 60.5)
		YLDs	3 (1.8 to 4.7)	2.7 (1.6 to 4.4)	3.3 (1.9 to 5.1)	4.6 (2.7 to 7.4)	4.3 (2.4 to 7.1)	4.9 (2.8 to 7.9)	53.2 (33.6 to 74.5)	56.8 (32.7 to 84.1)	48.2 (27.4 to 70)
	Iran (Islamic Republic of)	Incidence	2.1 (1.7 to 2.6)	1.7 (1.3 to 2.2)	2.5 (2.1 to 3.1)	3.2 (2.6 to 3.8)	2.5 (2 to 3.2)	3.9 (3.1 to 4.7)	49.7 (39.9 to 61.3)	47.7 (37.1 to 60)	52.1 (41.4 to 64.1)
		Prevalence	61.3 (53.2 to 71.4)	59.7 (51.8 to 69.9)	62.6 (53.9 to 73.4)	94.2 (80.9 to 110.8)	96.1 (81 to 114.7)	92.4 (78.6 to 109.8)	53.7 (42.3 to 65.9)	60.9 (48.4 to 76.5)	47.5 (36.7 to 58.2)
		Deaths	0.7 (0.5 to 1.1)	0.6 (0.4 to 0.9)	0.8 (0.5 to 1.2)	0.5 (0.4 to 0.8)	0.4 (0.3 to 0.6)	0.6 (0.4 to 0.9)	-26.6 (-34.2 to -18.4)	-27.1 (-38.7 to -16.4)	-25.4 (-35.1 to -12.9)
		DALYs	26.7 (18.2 to 37.4)	23 (15.9 to 32.2)	30 (20 to 42.9)	20.8 (14.7 to 27.8)	17.4 (12.5 to 23.1)	24.1 (16.6 to 32.9)	-22.2 (-29.5 to -13.2)	-24.4 (-33.3 to -13.9)	-19.7 (-29.2 to -7.5)
		YLLs	24.3 (16 to 34.7)	20.9 (13.8 to 29.6)	27.4 (17.5 to 39.8)	17.4 (11.7 to 24.2)	14.4 (9.7 to 19.9)	20.3 (13.3 to 28.8)	-28.4 (-35.4 to -20.2)	-30.9 (-39.6 to -20.3)	-25.7 (-34.9 to -13.5)
		YLDs	2.4 (1.6 to 3.3)	2.2 (1.5 to 3)	2.7 (1.8 to 3.7)	3.4 (2.2 to 4.8)	3 (2 to 4.3)	3.8 (2.5 to 5.3)	40.2 (27 to 54)	39.3 (23.5 to 57.4)	42.3 (29.2 to 55.2)

Cause	Location	Measure	Age-standardized rate (per 100,000)						% Change (1990 to 2019)		
			1990			2019					
			Both	Female	Male	Both	Female	Male	Both	Female	Male
	Iraq	Incidence	1.5 (0.7 to 3.3)	1.3 (0.5 to 3.3)	1.7 (0.8 to 3.5)	2.8 (1.5 to 5.6)	2.2 (0.9 to 5.2)	3.4 (1.7 to 6.3)	83.1 (61.2 to 122.1)	66.7 (47.6 to 100.8)	95.2 (69.3 to 138.1)
		Prevalence	38.4 (24.3 to 63.2)	37.3 (21.9 to 64.1)	39.6 (23.9 to 68.1)	70 (45.3 to 112.4)	67.6 (41.1 to 116)	72.5 (44.6 to 119.5)	82 (65 to 102.5)	81 (61.9 to 105)	83.4 (64.5 to 106.2)
		Deaths	1.7 (1.1 to 2.6)	1.6 (0.9 to 2.7)	1.8 (1.1 to 2.9)	1.3 (0.8 to 2.2)	1.1 (0.6 to 1.8)	1.6 (0.9 to 2.6)	-21 (-43.3 to 7.2)	-30.9 (-54.6 to 4.4)	-12.1 (-39 to 22.2)
		DALYs	61.5 (38.3 to 94.4)	59.8 (34.3 to 100.9)	63.4 (37.6 to 99.2)	48.3 (28.6 to 76.8)	41.5 (23.6 to 67.1)	55.2 (32 to 91.2)	-21.4 (-43.2 to 9.1)	-30.5 (-53.8 to 4.5)	-12.9 (-39.5 to 23.2)
		YLLs	59.3 (36.3 to 92.5)	57.8 (32.7 to 98.9)	61.1 (35.7 to 97)	44.4 (25.3 to 73.1)	38.2 (20.6 to 63.8)	50.8 (28.3 to 85.3)	-25.1 (-48.1 to 5.7)	-34 (-58.2 to 2.5)	-16.8 (-43.6 to 19.2)
		YLDs	2.2 (1.3 to 3.6)	1.9 (1.1 to 3.4)	2.4 (1.4 to 3.9)	3.9 (2.3 to 6.5)	3.4 (1.9 to 6)	4.5 (2.6 to 7.4)	81.9 (57.2 to 108.4)	73.5 (42 to 106.1)	89 (59.6 to 117.4)
	Jordan	Incidence	1.3 (0.5 to 3)	1.2 (0.4 to 3)	1.4 (0.5 to 3)	2.6 (1.3 to 5.2)	2.3 (0.9 to 5.1)	2.8 (1.3 to 5.6)	98.1 (71.6 to 155.8)	89.3 (61.8 to 143.8)	104.4 (74.4 to 170)
		Prevalence	36.8 (22.6 to 62.8)	39.7 (23.5 to 68.1)	34.3 (19.3 to 61.3)	67.5 (44.5 to 109.7)	76.4 (46.9 to 128.5)	60.5 (36.9 to 103.4)	83.4 (63.9 to 108.7)	92.5 (66.8 to 126.3)	76.3 (55.8 to 103.5)
		Deaths	1.3 (0.8 to 1.9)	1.3 (0.8 to 1.9)	1.3 (0.8 to 1.9)	1 (0.6 to 1.5)	0.9 (0.5 to 1.4)	1.1 (0.7 to 1.7)	-20.1 (-38.1 to 3.3)	-30.1 (-52.9 to -1.1)	-11.7 (-36.9 to 22.8)
		DALYs	43.8 (28.1 to 64)	43.8 (27.5 to 65.4)	43.8 (27.3 to 65)	35.5 (23.1 to 51.3)	30.9 (19.9 to 46)	39.4 (24.5 to 58.4)	-18.8 (-36.4 to 4.5)	-29.4 (-51.1 to -0.4)	-10 (-35.6 to 24.3)
		YLLs	41.8 (26.6 to 61.9)	41.8 (25.6 to 63.4)	41.9 (25.9 to 62.9)	32 (20.3 to 47.7)	27.2 (16.7 to 43)	36.1 (21.9 to 54.5)	-23.4 (-41.1 to 0.3)	-34.8 (-55.9 to -6.4)	-13.9 (-39.3 to 21)
		YLDs	1.9 (1.2 to 3.3)	2 (1.1 to 3.5)	1.9 (1.1 to 3.2)	3.5 (2 to 6)	3.6 (1.9 to 6.4)	3.4 (1.9 to 5.8)	80 (53.2 to 111.7)	85.4 (48.3 to 128.2)	76 (46.8 to 111.1)

Cause	Location	Measure	Age-standardized rate (per 100,000)						% Change (1990 to 2019)		
			1990			2019					
			Both	Female	Male	Both	Female	Male	Both	Female	Male
	Kuwait	Incidence	1.7 (0.9 to 3.4)	1.5 (0.7 to 3.4)	1.9 (1 to 3.5)	2.8 (1.6 to 5.1)	2.3 (1.1 to 5)	3.1 (1.8 to 5.6)	57.6 (39 to 90.5)	48.6 (31.8 to 77.4)	67 (43.1 to 105.5)
		Prevalence	49.1 (32.7 to 74.6)	52.9 (32.4 to 85.8)	46.3 (29.9 to 70.8)	72.5 (50.5 to 109.1)	76.9 (48.8 to 118.8)	67.8 (45.1 to 104.6)	47.8 (29.9 to 70.7)	45.4 (27.1 to 68.9)	46.5 (25.8 to 71.8)
		Deaths	0.8 (0.5 to 1.2)	0.9 (0.5 to 1.3)	0.8 (0.5 to 1.2)	0.4 (0.2 to 0.6)	0.3 (0.2 to 0.6)	0.4 (0.2 to 0.6)	-54 (-62.9 to -42.9)	-59.2 (-69.4 to -43.8)	-50.6 (-61.9 to -36.5)
		DALYs	28.7 (18.7 to 40.8)	30.7 (20 to 44.6)	27.5 (17.5 to 39.7)	14.7 (10 to 20.8)	13.9 (9.1 to 20.7)	15.1 (10 to 21.6)	-48.9 (-58.2 to -37.2)	-54.6 (-65.4 to -39.9)	-45.1 (-56.6 to -30.4)
		YLLs	26.4 (16.8 to 38.8)	28.5 (17.9 to 42.6)	25.2 (15.8 to 37.6)	11.2 (6.8 to 16.9)	10.7 (6.3 to 17)	11.5 (7 to 17.7)	-57.7 (-65.9 to -47.5)	-62.5 (-72.1 to -48.7)	-54.5 (-65.1 to -41.1)
		YLDs	2.2 (1.3 to 3.4)	2.2 (1.2 to 3.6)	2.2 (1.3 to 3.6)	3.5 (2 to 5.3)	3.2 (1.8 to 5.6)	3.6 (2 to 5.8)	55.4 (33.7 to 84.3)	49.1 (24.3 to 78.5)	60.4 (36.3 to 92.4)
	Lebanon	Incidence	1.4 (0.7 to 2.7)	1.2 (0.5 to 2.8)	1.6 (0.7 to 3.1)	2.7 (1.4 to 4.9)	2.2 (0.9 to 4.8)	3.2 (1.6 to 5.7)	93.3 (72.4 to 126.8)	84 (64 to 119.2)	104.2 (77 to 143.9)
		Prevalence	42 (28.1 to 64.9)	43.7 (27.4 to 71.2)	40.2 (24.8 to 63.9)	73.6 (48.9 to 114.2)	77.6 (48.1 to 124.8)	70 (44.4 to 111.9)	75.4 (60.1 to 91.4)	77.8 (60.2 to 98.2)	74.3 (54.7 to 95.2)
		Deaths	1 (0.6 to 1.5)	0.9 (0.6 to 1.4)	1 (0.6 to 1.6)	0.6 (0.3 to 1)	0.5 (0.3 to 0.8)	0.7 (0.4 to 1.2)	-38.9 (-57.4 to -16.7)	-40.4 (-61 to -16.4)	-36.3 (-60.4 to -2.9)
		DALYs	34.7 (22 to 52.7)	33.8 (20.8 to 52.1)	35.5 (22.1 to 56.2)	22.8 (14.2 to 35)	21.5 (13.3 to 32.1)	24.5 (14.2 to 41.9)	-34.4 (-52.6 to -12.4)	-36.3 (-57.5 to -10)	-31.2 (-53.2 to 0.7)
		YLLs	32.8 (20.4 to 50.6)	31.9 (19.2 to 50.2)	33.5 (20.4 to 54.1)	19.4 (11.4 to 31.8)	18.3 (10.2 to 28.6)	20.8 (11.4 to 38.3)	-40.9 (-59.4 to -18.5)	-42.7 (-63.7 to -16)	-38 (-60.3 to -4.9)
		YLDs	1.9 (1.1 to 3.3)	1.8 (1 to 3.3)	2 (1.2 to 3.4)	3.4 (2 to 6.2)	3.2 (1.7 to 6.1)	3.6 (2 to 6.4)	78 (53.4 to 103.2)	74.8 (41.9 to 106.6)	84 (58.2 to 113.6)

Cause	Location	Measure	Age-standardized rate (per 100,000)						% Change (1990 to 2019)		
			1990			2019					
			Both	Female	Male	Both	Female	Male	Both	Female	Male
	Libya	Incidence	1.4 (0.7 to 2.9)	1.3 (0.5 to 2.9)	1.5 (0.7 to 3.2)	2.7 (1.5 to 5.1)	2.2 (1 to 4.9)	3.1 (1.6 to 5.9)	90.2 (67.7 to 125.4)	79.4 (58.4 to 109.3)	100.8 (71.6 to 148)
		Prevalence	40.7 (26.4 to 65.9)	43.6 (27.5 to 74.2)	38.3 (23.3 to 65.4)	73.5 (49.8 to 118.2)	78 (49.6 to 131.4)	69.5 (43.9 to 119.3)	80.8 (62.6 to 100.6)	78.8 (58.8 to 99.9)	81.3 (60.5 to 108)
		Deaths	0.9 (0.6 to 1.5)	1 (0.6 to 1.5)	0.9 (0.5 to 1.5)	0.9 (0.5 to 1.6)	1 (0.5 to 1.6)	0.9 (0.5 to 1.7)	0.3 (-29.6 to 41.6)	1.3 (-32.3 to 42.7)	-1.6 (-35.2 to 48)
		DALYs	34 (21.1 to 51.7)	36.2 (21.7 to 55.6)	32.4 (19 to 51.7)	35.5 (20.6 to 56.7)	37.5 (20.7 to 59.5)	33.8 (19.3 to 58.1)	4.4 (-26 to 45.9)	3.4 (-29.4 to 44.8)	4.1 (-29.1 to 52.3)
		YLLs	32.1 (19.4 to 49.7)	34.3 (19.7 to 53.9)	30.5 (17.4 to 49.5)	31.9 (17.9 to 52.8)	34 (17.7 to 56.1)	30 (15.8 to 54.7)	-0.6 (-31.6 to 42.1)	-0.8 (-34.5 to 42.9)	-1.7 (-35.7 to 49.6)
		YLDs	1.9 (1.2 to 3.3)	1.9 (1.1 to 3.2)	2 (1.1 to 3.3)	3.6 (2.1 to 6.2)	3.4 (1.8 to 6.1)	3.8 (2.2 to 6.6)	86.1 (61.5 to 111)	78.3 (45 to 108.5)	94 (66.4 to 125.8)
	Morocco	Incidence	1.2 (0.6 to 2.5)	1.1 (0.4 to 2.3)	1.4 (0.7 to 2.7)	2.6 (1.3 to 5.1)	2.1 (0.9 to 4.6)	3.2 (1.6 to 6)	115.6 (98 to 141.1)	100.2 (83.1 to 125.1)	126.6 (103.6 to 159)
		Prevalence	40.6 (27.1 to 60.3)	42.3 (26.7 to 65)	38.7 (24.9 to 62.7)	71.8 (45.9 to 109.5)	72.5 (43.6 to 115.1)	71 (44.8 to 117.5)	76.9 (54.8 to 99.3)	71.1 (48.6 to 97.2)	83.3 (55.8 to 110.7)
		Deaths	0.9 (0.5 to 1.3)	0.8 (0.4 to 1.2)	0.9 (0.6 to 1.5)	0.9 (0.5 to 1.5)	0.9 (0.5 to 1.6)	0.9 (0.5 to 1.6)	10.7 (-21.9 to 47.4)	22.8 (-20.9 to 77.4)	0.6 (-30.8 to 37.9)
		DALYs	30.8 (19.4 to 45.8)	29.1 (17.2 to 44.3)	32.5 (19.5 to 49.9)	34.3 (20.4 to 53.9)	35.9 (20.8 to 57.1)	32.7 (18.8 to 52.8)	11.5 (-20.4 to 48)	23.5 (-19.8 to 78.7)	0.5 (-28.4 to 38.5)
		YLLs	29 (17.8 to 44)	27.4 (15.8 to 42.6)	30.7 (17.8 to 47.7)	30.7 (17.2 to 50.5)	32.6 (18.1 to 54.3)	28.8 (15 to 48.9)	5.7 (-27.3 to 44.2)	18.9 (-25.4 to 77.4)	-6.4 (-35.4 to 32.6)
		YLDs	1.7 (1 to 2.8)	1.7 (0.9 to 3)	1.8 (1.1 to 2.9)	3.6 (2 to 6.2)	3.3 (1.6 to 6.3)	3.9 (2.3 to 6.7)	107.9 (80.8 to 136.9)	99.4 (66.9 to 134.8)	115.2 (82.2 to 150.7)

Cause	Location	Measure	Age-standardized rate (per 100,000)						% Change (1990 to 2019)		
			1990			2019					
			Both	Female	Male	Both	Female	Male	Both	Female	Male
	Oman	Incidence	1.1 (0.6 to 2.2)	0.9 (0.4 to 2)	1.3 (0.6 to 2.4)	2.6 (1.5 to 4.6)	2 (0.9 to 4.1)	3 (1.7 to 5.2)	131.4 (100.2 to 185.1)	119 (88.2 to 171)	136.5 (99.8 to 192.1)
		Prevalence	37.9 (25.2 to 57.8)	38.9 (24 to 61.1)	37.3 (23.5 to 59.9)	73.5 (50.3 to 110.1)	80.5 (50.5 to 123.6)	71.5 (45.9 to 110.9)	94 (75.7 to 117.2)	106.7 (85.6 to 133.2)	91.5 (67.6 to 120.1)
		Deaths	0.4 (0.2 to 0.7)	0.4 (0.2 to 0.7)	0.5 (0.3 to 0.8)	0.4 (0.2 to 0.6)	0.4 (0.3 to 0.6)	0.4 (0.3 to 0.7)	-8.1 (-35.6 to 29.5)	-3.9 (-34.2 to 37.4)	-11.2 (-43.1 to 32.9)
		DALYs	15.2 (9.2 to 24.1)	15.5 (9.4 to 24.2)	15.6 (9.2 to 25.8)	14.8 (10 to 21.3)	15.6 (10.7 to 22.1)	14.9 (9.6 to 22.5)	-2.5 (-28.9 to 31.4)	0.9 (-29.1 to 40.8)	-4.4 (-34.9 to 36.4)
		YLLs	13.2 (7.5 to 21.9)	13.6 (7.7 to 22.3)	13.4 (7.4 to 23.7)	10.8 (6.6 to 16.9)	11.9 (7.6 to 17.6)	10.6 (6.3 to 18)	-18.3 (-43.4 to 16.7)	-12.8 (-40.4 to 27.4)	-21.3 (-49.6 to 19.2)
		YLDs	2.1 (1.3 to 3.2)	1.9 (1.1 to 3)	2.2 (1.3 to 3.4)	4.1 (2.4 to 6.6)	3.7 (2.1 to 6.2)	4.4 (2.5 to 7.2)	98 (65.3 to 133.8)	99.8 (60.5 to 143.9)	99 (62 to 139.8)
	Palestine	Incidence	1.8 (0.8 to 3.8)	1.5 (0.5 to 3.6)	2.1 (1 to 4.4)	2.8 (1.4 to 5.4)	2.2 (0.9 to 5.1)	3.4 (1.7 to 6.4)	55.7 (38.4 to 85)	49.9 (35.2 to 78.4)	57 (37.7 to 87.1)
		Prevalence	49.1 (31.9 to 81)	49 (29.4 to 83.7)	49.3 (29.5 to 82.5)	78.3 (50.3 to 125)	81 (49.7 to 135.1)	75.8 (45.9 to 125.1)	59.6 (45.9 to 77.9)	65.4 (47.6 to 88.8)	53.7 (38.6 to 72.1)
		Deaths	1.4 (0.8 to 2.2)	1.2 (0.7 to 2)	1.6 (0.9 to 2.7)	1 (0.6 to 1.5)	0.8 (0.5 to 1.2)	1.2 (0.7 to 1.7)	-31.1 (-49 to -6.8)	-35.1 (-52.7 to -9.4)	-29.6 (-48.3 to -2.5)
		DALYs	49.9 (30.3 to 77)	45.3 (27.4 to 70.6)	56.1 (32.5 to 89.1)	35.8 (24.2 to 51.4)	30.4 (20.4 to 43.5)	41.2 (27 to 60.2)	-28.3 (-46.8 to -2.5)	-32.9 (-50.8 to -6.8)	-26.6 (-45.8 to 1.7)
		YLLs	47.5 (28.2 to 74.8)	43 (25.4 to 68)	53.5 (30.3 to 86.5)	31.9 (20.4 to 46.9)	26.8 (17.1 to 39.5)	37 (23.7 to 55.4)	-32.8 (-51.2 to -7.6)	-37.8 (-55.5 to -11.7)	-30.7 (-49.6 to -1.8)
		YLDs	2.4 (1.4 to 4.1)	2.3 (1.3 to 4)	2.6 (1.5 to 4.4)	3.9 (2.2 to 6.5)	3.6 (2 to 6.5)	4.2 (2.3 to 7)	59.7 (38.5 to 82)	58.2 (34 to 83.4)	58.4 (35.8 to 84.2)

Cause	Location	Measure	Age-standardized rate (per 100,000)						% Change (1990 to 2019)		
			1990			2019					
			Both	Female	Male	Both	Female	Male	Both	Female	Male
	Qatar	Incidence	1.3 (0.6 to 2.8)	1.1 (0.4 to 2.7)	1.4 (0.6 to 3)	2.4 (1.2 to 4.8)	1.9 (0.7 to 4.7)	2.6 (1.2 to 5.3)	87.4 (68 to 120.4)	72.1 (56 to 92)	90.8 (68.4 to 124.7)
		Prevalence	36.1 (23 to 60.5)	38 (22.2 to 65.2)	35.4 (21.4 to 59.6)	60 (38 to 100.9)	64.1 (37.4 to 113.5)	59.9 (36.9 to 101.7)	66.3 (51.4 to 83)	68.7 (51.3 to 86.6)	69 (52 to 87.9)
		Deaths	1 (0.6 to 1.7)	1.2 (0.6 to 2.1)	1 (0.5 to 1.7)	0.7 (0.4 to 1.1)	1 (0.6 to 1.5)	0.6 (0.4 to 1)	-29.4 (-52.8 to 1.5)	-16.7 (-51.6 to 26.5)	-34.9 (-55.1 to -1.1)
		DALYs	28.6 (17.1 to 46.4)	34.3 (20 to 59.6)	26.7 (15.5 to 43.8)	19.3 (12.4 to 28.1)	23.5 (15.1 to 35.3)	17.9 (11.5 to 26.5)	-32.6 (-52 to -6.5)	-31.5 (-57 to -1.4)	-32.9 (-52.6 to -3.3)
		YLLs	26.6 (15.3 to 44)	32.4 (18 to 57.7)	24.6 (13.7 to 41.4)	16 (9.5 to 24.6)	20.4 (12.4 to 32)	14.5 (8.6 to 22.4)	-39.8 (-58.3 to -13.7)	-36.8 (-61.5 to -5.6)	-41.1 (-59.8 to -10.9)
		YLDs	2.1 (1.2 to 3.3)	1.9 (1.1 to 3.2)	2.1 (1.2 to 3.5)	3.3 (1.8 to 5.7)	3 (1.6 to 5.6)	3.4 (1.9 to 5.9)	60.4 (36.2 to 85.7)	59.2 (30.6 to 90.4)	61.9 (37 to 88)
	Saudi Arabia	Incidence	1.7 (0.9 to 3.2)	1.5 (0.7 to 3.1)	1.8 (0.9 to 3.4)	4.2 (2.5 to 7.1)	3.5 (1.7 to 6.7)	4.6 (2.6 to 7.7)	145.2 (112.3 to 199.9)	127.6 (100.4 to 170.4)	154.5 (114.8 to 223.1)
		Prevalence	47.7 (33.8 to 71)	52.6 (34.8 to 79.7)	44.5 (29.3 to 69.2)	97.3 (70.4 to 142.3)	102.6 (67.9 to 154.2)	93.6 (65 to 140.2)	104.1 (83.5 to 127.7)	94.9 (71.3 to 122)	110.5 (85.6 to 143)
		Deaths	1.6 (0.9 to 2.6)	1.4 (0.8 to 2.4)	1.7 (0.9 to 2.9)	1.6 (0.9 to 2.5)	1.6 (0.9 to 2.4)	1.6 (0.9 to 2.5)	-0.4 (-33.1 to 47.8)	8.7 (-33.7 to 63.3)	-6.5 (-40.1 to 48.6)
		DALYs	55.3 (32.4 to 87.8)	54.6 (31.1 to 88.1)	56.1 (31.4 to 93.6)	57.3 (35.5 to 89.5)	60.2 (35.7 to 93.3)	55.5 (33.7 to 87.7)	3.7 (-29.6 to 53.3)	10.3 (-30.9 to 67.2)	-1.1 (-36.5 to 53.3)
		YLLs	52.4 (29.8 to 84.8)	51.8 (28.3 to 84.9)	53.2 (28.8 to 90.1)	51.6 (30.2 to 82.8)	54.7 (31.2 to 87.1)	49.7 (28.5 to 80.5)	-1.5 (-35.4 to 49.6)	5.7 (-36.4 to 64.1)	-6.7 (-41.5 to 49.8)
		YLDs	2.8 (1.8 to 4.3)	2.8 (1.7 to 4.4)	2.9 (1.7 to 4.4)	5.7 (3.4 to 9)	5.5 (2.9 to 9)	5.8 (3.3 to 9.2)	99 (64.6 to 140.2)	94.2 (52.4 to 139.4)	102 (58.3 to 151.1)

Cause	Location	Measure	Age-standardized rate (per 100,000)						% Change (1990 to 2019)		
			1990			2019					
			Both	Female	Male	Both	Female	Male	Both	Female	Male
	Sudan	Incidence	1.1 (0.5 to 2.2)	0.9 (0.3 to 2)	1.3 (0.6 to 2.6)	2 (1.1 to 4.1)	1.6 (0.6 to 3.5)	2.5 (1.3 to 4.8)	89.7 (73.4 to 119.7)	78.2 (61.6 to 102)	95.9 (75.7 to 130.4)
		Prevalence	34.6 (22.7 to 53.1)	34.8 (20.8 to 56)	34.3 (21.1 to 54.6)	59.5 (38.4 to 93.6)	59.5 (35.2 to 98.3)	58.8 (36.2 to 93.8)	71.8 (53.9 to 89.9)	71.1 (50.1 to 93.2)	71.2 (50 to 93.4)
		Deaths	0.8 (0.5 to 1.4)	0.8 (0.4 to 1.3)	0.9 (0.5 to 1.5)	0.8 (0.4 to 1.4)	0.7 (0.4 to 1.3)	0.8 (0.4 to 1.6)	-7.4 (-43 to 44.9)	-7.3 (-50 to 44.1)	-8.1 (-43.5 to 65.6)
		DALYs	31 (18.2 to 47.9)	29.5 (16.5 to 48.5)	32.1 (19 to 50.7)	29 (16.4 to 49)	27.3 (14.7 to 47.1)	30.3 (16.3 to 56)	-6.5 (-41.3 to 44.5)	-7.6 (-48.3 to 45.6)	-5.7 (-41.7 to 68.1)
		YLLs	29.6 (17.2 to 46.6)	28.3 (15.2 to 47.4)	30.6 (18 to 48.7)	26.3 (13.8 to 46.2)	24.9 (12.5 to 44.8)	27.3 (13.2 to 53.6)	-11.2 (-47.2 to 41.7)	-11.9 (-53.3 to 42.7)	-10.8 (-47.4 to 66.1)
		YLDs	1.4 (0.8 to 2.2)	1.3 (0.7 to 2.2)	1.5 (0.9 to 2.4)	2.7 (1.5 to 4.5)	2.4 (1.2 to 4.3)	3 (1.7 to 4.8)	96.1 (74.2 to 121)	90.1 (60.4 to 122.8)	99.1 (72.9 to 132.2)
	Syrian Arab Republic	Incidence	1.7 (0.8 to 3.8)	1.4 (0.5 to 3.3)	2 (0.9 to 4.4)	2.8 (1.5 to 5.2)	2.2 (1 to 4.6)	3.4 (1.8 to 6.4)	61.2 (35.8 to 114.8)	51.8 (28.9 to 96.6)	69.2 (39.2 to 130.1)
		Prevalence	44.5 (28.7 to 73.8)	43.7 (27.5 to 72.9)	45.2 (27.5 to 79.6)	77.4 (52.9 to 117.9)	78.1 (49.6 to 123.2)	74.7 (48.8 to 120.7)	74 (51.7 to 101)	78.9 (54.2 to 107.9)	65.1 (45.8 to 92.4)
		Deaths	1.3 (0.8 to 2)	1.2 (0.7 to 1.8)	1.4 (0.8 to 2.1)	0.7 (0.4 to 1.2)	0.7 (0.4 to 1.1)	0.8 (0.5 to 1.3)	-41.9 (-60 to -16.5)	-41.9 (-61.5 to -12.4)	-41.7 (-61.3 to -13.4)
		DALYs	49.7 (31.2 to 75.3)	47.4 (29.1 to 72.9)	51.7 (31.4 to 79.2)	28.9 (17.8 to 45.3)	26.7 (16.2 to 41.3)	30.7 (19.1 to 49)	-41.8 (-58.7 to -17.8)	-43.7 (-62.4 to -15.5)	-40.5 (-58.4 to -14.2)
		YLLs	47.4 (29.4 to 71.6)	45.3 (26.9 to 70.6)	49.3 (29.3 to 77.3)	25.1 (14.8 to 40.8)	23.3 (13.3 to 37.1)	26.7 (15.7 to 44.3)	-47 (-64.1 to -22.9)	-48.6 (-66.7 to -20.9)	-45.8 (-63.2 to -18.6)
		YLDs	2.3 (1.4 to 3.7)	2.1 (1.2 to 3.7)	2.4 (1.4 to 3.9)	3.8 (2.2 to 6.2)	3.4 (1.8 to 6)	4 (2.4 to 6.7)	65.3 (43.3 to 91.6)	60.6 (33.4 to 91.5)	67.5 (42.5 to 97.6)

Cause	Location	Measure	Age-standardized rate (per 100,000)						% Change (1990 to 2019)		
			1990			2019					
			Both	Female	Male	Both	Female	Male	Both	Female	Male
	Tunisia	Incidence	1.2 (0.6 to 2.4)	1 (0.4 to 2.3)	1.3 (0.6 to 2.5)	2.4 (1.3 to 4.5)	1.9 (0.9 to 4)	2.8 (1.5 to 5.1)	103.4 (77.3 to 149.3)	86.9 (63.6 to 127.4)	117.3 (86.3 to 171.9)
		Prevalence	37.6 (24.3 to 60.3)	39.8 (24.2 to 64.3)	35.3 (21.8 to 57.3)	68 (45.3 to 103.3)	71 (43.5 to 111.1)	64.6 (40.7 to 102.6)	80.9 (62.9 to 103.1)	78.2 (58.1 to 103.3)	83 (60.2 to 111.6)
		Deaths	0.6 (0.4 to 0.9)	0.5 (0.3 to 0.8)	0.6 (0.4 to 1)	0.5 (0.3 to 0.9)	0.5 (0.3 to 0.8)	0.6 (0.3 to 1)	-6.5 (-36.7 to 32.9)	-8.9 (-41.7 to 33.6)	-3.6 (-38.6 to 42.9)
		DALYs	20.8 (13.2 to 31.8)	20.1 (12.8 to 30)	21.5 (12.9 to 33.1)	20.8 (13.1 to 32.4)	19.2 (11.9 to 29.8)	22.4 (13.4 to 35.3)	-0.1 (-28.2 to 37.4)	-4.4 (-33.6 to 34)	4.5 (-27.7 to 44.9)
		YLLs	18.6 (11.4 to 28.7)	18 (11 to 27.7)	19.2 (11.1 to 30.3)	17.1 (9.9 to 28.2)	15.8 (8.8 to 25.9)	18.5 (10.3 to 31)	-8 (-38.8 to 32.9)	-12.2 (-44.8 to 31)	-3.6 (-38.1 to 41.6)
		YLDs	2.2 (1.4 to 3.5)	2.1 (1.3 to 3.4)	2.3 (1.3 to 3.7)	3.7 (2.2 to 6.1)	3.4 (1.9 to 6)	3.9 (2.3 to 6.4)	67.2 (43.9 to 94.4)	62 (34.2 to 91.9)	72.1 (44 to 104.5)
	Turkey	Incidence	1.3 (0.6 to 2.7)	1 (0.4 to 2.2)	1.6 (0.8 to 3.3)	2.9 (1.6 to 5)	2.1 (1 to 4.3)	3.7 (2.1 to 6.4)	120.8 (80.5 to 190.2)	115.1 (75.4 to 191.3)	125.3 (81.9 to 195.8)
		Prevalence	45.2 (30.5 to 69.3)	44.3 (28.5 to 70.4)	46.1 (28.8 to 73.1)	88.1 (61.9 to 128.5)	90.1 (58.7 to 136.1)	87 (56.7 to 132.6)	95 (76.9 to 118.3)	103.2 (81.7 to 131.6)	88.6 (67.3 to 117.3)
		Deaths	1.2 (0.7 to 1.8)	0.9 (0.5 to 1.4)	1.4 (0.9 to 2.3)	0.6 (0.4 to 0.9)	0.5 (0.3 to 0.7)	0.7 (0.5 to 1.1)	-48.1 (-63.7 to -29.5)	-46.2 (-65.1 to -25.8)	-49.5 (-65.7 to -27.3)
		DALYs	42.2 (26.8 to 62)	33.6 (21 to 51.3)	51.2 (32 to 77.5)	23.4 (15.5 to 33.4)	18.8 (12.4 to 27.1)	28.1 (18.3 to 40.5)	-44.6 (-59.1 to -27.2)	-44 (-61.4 to -23.7)	-45 (-60.4 to -23.2)
		YLLs	39.7 (24.6 to 59.2)	31.3 (19.2 to 48.5)	48.3 (29.7 to 74.1)	18.8 (11.6 to 28.2)	14.8 (9.2 to 22.4)	23 (14 to 35.1)	-52.5 (-66.4 to -35.9)	-52.8 (-68.6 to -34)	-52.4 (-67.2 to -31.9)
		YLDs	2.6 (1.6 to 3.9)	2.3 (1.4 to 3.5)	2.9 (1.8 to 4.3)	4.6 (2.8 to 7)	4 (2.4 to 6.5)	5.2 (3.1 to 7.9)	77.5 (51.6 to 106)	76.4 (44.7 to 113.4)	79.4 (50.8 to 111.5)

Cause	Location	Measure	Age-standardized rate (per 100,000)						% Change (1990 to 2019)		
			1990			2019					
			Both	Female	Male	Both	Female	Male	Both	Female	Male
	United Arab Emirates	Incidence	1.7 (0.8 to 3.6)	1.3 (0.5 to 3)	1.9 (0.9 to 4.1)	3.6 (1.9 to 6.6)	2.5 (1.1 to 5.2)	4 (2.1 to 7.6)	109.7 (77.4 to 170.2)	97.2 (70.8 to 150.9)	108.3 (76.1 to 168)
		Prevalence	44.8 (28.6 to 71.2)	43 (26.2 to 72.8)	46.1 (28.4 to 75.6)	86 (57.7 to 133.1)	85 (54.7 to 135.6)	86.9 (56.2 to 139)	91.9 (72.7 to 115.9)	97.6 (77.6 to 123.5)	88.8 (69 to 115.5)
		Deaths	2 (1.2 to 3)	1.7 (0.9 to 2.9)	2.1 (1.2 to 3.3)	1.7 (0.9 to 3.1)	1.5 (0.8 to 2.5)	1.7 (0.9 to 3.4)	-13.5 (-44 to 33.3)	-13.1 (-53.2 to 36)	-15.7 (-47.1 to 40.5)
		DALYs	66.8 (40.8 to 102.3)	59.5 (33.6 to 97.9)	70 (41.6 to 110.8)	63.3 (35.3 to 113.6)	56.1 (30.3 to 88.6)	65.3 (35.6 to 127)	-5.3 (-38 to 44.3)	-5.7 (-48.2 to 46)	-6.8 (-41.3 to 54.2)
		YLLs	64.4 (38.2 to 99.8)	57.4 (31.8 to 95.9)	67.4 (39.2 to 108.2)	58.5 (31 to 109.4)	52 (27 to 85)	60.2 (31 to 120.9)	-9.2 (-43.4 to 41.6)	-9.3 (-52.6 to 44.7)	-10.8 (-46 to 52.5)
		YLDs	2.4 (1.5 to 4)	2.1 (1.2 to 3.5)	2.6 (1.5 to 4.4)	4.8 (2.9 to 8)	4 (2.3 to 7.1)	5.1 (2.9 to 8.6)	97.7 (70.4 to 131.9)	92.4 (57.8 to 131.8)	98 (67.6 to 135.5)
	Yemen	Incidence	1.3 (0.7 to 2.5)	1 (0.4 to 2.3)	1.6 (0.8 to 3)	2.4 (1.3 to 4.4)	1.8 (0.8 to 4)	2.9 (1.6 to 5.2)	83.6 (65 to 110.7)	80.9 (57.3 to 120.5)	86.7 (64.5 to 119.1)
		Prevalence	44.6 (30.6 to 67.4)	45.7 (29 to 71.3)	43.4 (27.6 to 67.1)	75.5 (51.5 to 116)	78.5 (49.6 to 126.5)	72.4 (46.2 to 111.4)	69.2 (53.7 to 88.3)	72 (52.1 to 97.9)	66.9 (49.1 to 86.8)
		Deaths	0.8 (0.5 to 1.4)	0.7 (0.4 to 1.3)	1 (0.5 to 1.6)	0.7 (0.4 to 1.2)	0.6 (0.3 to 1)	0.8 (0.5 to 1.3)	-13.7 (-39.6 to 25)	-11.1 (-42.8 to 34.5)	-16.3 (-42.6 to 24.6)
		DALYs	30.4 (17 to 50.8)	26.6 (13.9 to 47.5)	34.1 (19.2 to 56.1)	27.7 (16.4 to 43.4)	25.1 (14.6 to 41.8)	30.3 (17.9 to 47.8)	-8.8 (-36.4 to 32)	-5.6 (-39.2 to 44)	-10.9 (-39.2 to 33.8)
		YLLs	28.3 (15.2 to 48.3)	24.7 (12.3 to 46)	31.9 (17.3 to 54)	24 (13.2 to 40.4)	21.5 (11.4 to 37.8)	26.6 (14.6 to 44.3)	-15.3 (-42.8 to 27.9)	-12.8 (-46.3 to 39.2)	-16.8 (-44.8 to 30)
		YLDs	2 (1.2 to 3.1)	1.9 (1.1 to 3.1)	2.1 (1.2 to 3.3)	3.7 (2.2 to 5.7)	3.6 (1.9 to 6.1)	3.8 (2.2 to 6.1)	81.3 (56.8 to 107.1)	87.9 (52.2 to 129.6)	76.7 (51.3 to 104.6)

Cause	Location	Measure	Age-standardized rate (per 100,000)						% Change (1990 to 2019)		
			1990			2019					
			Both	Female	Male	Both	Female	Male	Both	Female	Male
Chronic kidney disease due to diabetes mellitus type 2	North Africa and Middle East	Incidence	35.1 (31.7 to 38.8)	37 (33.2 to 41.4)	33.2 (30.1 to 36.7)	61.3 (56 to 67.4)	58.8 (53.7 to 64.7)	63.8 (58 to 70.4)	74.9 (70.3 to 80.1)	58.9 (53.7 to 64.7)	92.2 (86.3 to 98.2)
		Prevalence	1413.4 (1306.5 to 1526)	1489.8 (1372.8 to 1610.8)	1340.6 (1237.9 to 1445.6)	1762.5 (1636.5 to 1893.4)	1772.3 (1641.7 to 1904.1)	1757.8 (1630.1 to 1884.7)	24.7 (21.2 to 28.2)	19 (15.2 to 22.7)	31.1 (27.1 to 35.3)
		Deaths	10.7 (8.3 to 14)	10.2 (7.8 to 14.4)	11.3 (8.7 to 15.4)	9.6 (7.5 to 12)	9.6 (7.3 to 12.2)	9.6 (7.4 to 12.4)	-10.2 (-28.9 to 8.2)	-5.5 (-29.8 to 12)	-15.2 (-35.1 to 10.7)
		DALYs	222.2 (175.5 to 274.4)	218.8 (172 to 285.7)	226.5 (177.2 to 287.6)	205.9 (162.4 to 253.6)	207.4 (160.2 to 257)	204.8 (159.8 to 263.7)	-7.3 (-24.6 to 9.3)	-5.2 (-27.8 to 11.7)	-9.6 (-29.6 to 14.3)
		YLLs	203.5 (159.4 to 254.8)	198.6 (154.1 to 262.2)	209.3 (161.9 to 270.7)	176.6 (133.7 to 223.8)	179.2 (133.8 to 227.9)	174.1 (133 to 230.8)	-13.2 (-31.2 to 4.6)	-9.8 (-33.2 to 8.3)	-16.8 (-36.1 to 8.8)
		YLDs	18.6 (13.5 to 25.6)	20.1 (14.5 to 27.3)	17.2 (12.1 to 24)	29.4 (20.7 to 41)	28.1 (20 to 38.5)	30.7 (20.9 to 44.1)	57.6 (47.9 to 67.8)	39.7 (30.8 to 49.5)	78.6 (66.2 to 91.2)
	Afghanistan	Incidence	32 (28.1 to 36.5)	33.8 (29.2 to 39)	30.4 (26.8 to 34.4)	51.7 (45.5 to 58.9)	49.7 (43 to 56.8)	54 (47.5 to 61.2)	61.4 (53.9 to 69.6)	46.9 (38.5 to 58.4)	77.9 (68 to 88.4)
		Prevalence	1366 (1244 to 1506.6)	1454.4 (1310 to 1620.8)	1266.5 (1146.4 to 1400.4)	1608.3 (1474.1 to 1743.6)	1650.4 (1492.6 to 1812.8)	1571.7 (1431.5 to 1719.3)	17.7 (13.2 to 22.6)	13.5 (8.7 to 19)	24.1 (18.6 to 30)
		Deaths	17.6 (12.1 to 27.9)	16.7 (11.2 to 29.3)	18.3 (12.2 to 28.7)	16.1 (11.2 to 25.5)	16.1 (10.5 to 27.8)	16 (10.3 to 25.9)	-8.5 (-30.5 to 18.3)	-3.9 (-36.9 to 34.5)	-12.3 (-34.4 to 12.4)
		DALYs	388.7 (269.2 to 566.2)	394.4 (270.4 to 613.4)	379 (253.5 to 568.4)	351.7 (249.8 to 512.9)	368.4 (250.4 to 573.6)	333.8 (222.6 to 504.6)	-9.5 (-32.6 to 18.2)	-6.6 (-38.2 to 30.5)	-11.9 (-35.3 to 15.6)
		YLLs	370.7 (252.6 to 548.2)	374 (249.5 to 592.3)	363.6 (237.3 to 551.5)	324.4 (223.2 to 486.3)	340.4 (221.5 to 540.5)	307.1 (198.5 to 477.7)	-12.5 (-35.6 to 16.9)	-9 (-41.4 to 29.8)	-15.5 (-38.7 to 13.8)
		YLDs	18 (12.5 to 24.8)	20.4 (14.2 to 27.9)	15.5 (10.4 to 22.5)	27.3 (18.8 to 38.4)	28.1 (19.3 to 39.1)	26.7 (17.9 to 38.9)	51.7 (35.8 to 68.7)	37.7 (19.3 to 58)	72.3 (46.8 to 102.2)

Cause	Location	Measure	Age-standardized rate (per 100,000)						% Change (1990 to 2019)		
			1990			2019					
			Both	Female	Male	Both	Female	Male	Both	Female	Male
	Algeria	Incidence	36.5 (32.3 to 41.2)	39.4 (34.4 to 45.4)	33.3 (29 to 37.9)	62.5 (55.2 to 70.4)	60.8 (53.5 to 68.3)	64.2 (55.8 to 73.4)	71.6 (63.7 to 79.4)	54.3 (45.1 to 64.3)	93 (79.1 to 106.4)
		Prevalence	1436.1 (1308.1 to 1575.9)	1533.7 (1377.2 to 1705.7)	1338.1 (1209.1 to 1468.7)	1778.4 (1627.1 to 1940.8)	1813.2 (1646.4 to 1984.1)	1741.2 (1580.7 to 1909)	23.8 (18.6 to 28.6)	18.2 (12.5 to 23.6)	30.1 (23.9 to 36.6)
		Deaths	11.5 (8.3 to 16.9)	12 (7.8 to 20.9)	11.1 (8.2 to 15.3)	9.6 (6.8 to 13.2)	11.3 (7.7 to 17.1)	8.4 (5.8 to 11.8)	-16.6 (-36.5 to 10.6)	-6 (-31.3 to 28.8)	-24.9 (-45.1 to 5.2)
		DALYs	218.1 (158.1 to 310.7)	231 (158.2 to 379.8)	205.9 (150.6 to 282)	187.5 (138.8 to 254.1)	211.6 (151.4 to 307.1)	166.9 (120.4 to 232.7)	-14 (-34.7 to 12.5)	-8.4 (-33.5 to 24.4)	-19 (-40 to 9.8)
		YLLs	199.6 (139.1 to 293.2)	210.1 (137.2 to 358.1)	189.7 (135.9 to 264.8)	157.8 (111 to 222.4)	182.2 (121.9 to 276)	136.9 (92.4 to 199.2)	-20.9 (-41.9 to 7.2)	-13.3 (-38.3 to 21.7)	-27.8 (-48.3 to 2.7)
		YLDs	18.5 (12.9 to 26)	20.8 (14.6 to 29.2)	16.2 (10.7 to 23.8)	29.7 (20.2 to 42.5)	29.4 (20.5 to 41.4)	30 (19.8 to 44.5)	60.7 (42.1 to 81.1)	41.1 (20.4 to 63.8)	85.3 (56 to 118.7)
	Bahrain	Incidence	43.6 (39 to 49.1)	46.4 (41 to 52.2)	41.7 (36.5 to 47.4)	74.2 (66.4 to 82.6)	67.9 (60.8 to 75.5)	78.9 (69.5 to 88.5)	70.2 (60.9 to 80)	46.5 (37.3 to 56.3)	89.1 (75.5 to 102.1)
		Prevalence	1538.8 (1406.7 to 1686.8)	1650.5 (1498.1 to 1829.9)	1466.6 (1330.9 to 1610.6)	1951.2 (1784.2 to 2133.3)	1943.6 (1770.7 to 2121.9)	1986.9 (1803.9 to 2179.5)	26.8 (21.5 to 32.2)	17.8 (12.4 to 23.4)	35.5 (28.5 to 42.3)
		Deaths	13.1 (10 to 16.9)	13.2 (9.6 to 17.1)	13.3 (9.8 to 18.6)	11.9 (8.9 to 15.4)	11.8 (8.6 to 15.3)	12.1 (8.8 to 16.5)	-8.8 (-29.2 to 15.2)	-10 (-34.3 to 17.4)	-8.7 (-30.6 to 19.2)
		DALYs	246.9 (193.6 to 311.2)	260 (195.8 to 331.1)	240 (181.7 to 324.3)	219.5 (171.2 to 276.7)	219.3 (167 to 277.4)	222.5 (168.3 to 286.4)	-11.1 (-30 to 10.7)	-15.7 (-36.9 to 9.3)	-7.3 (-28.7 to 18.6)
		YLLs	224.6 (170.1 to 287.6)	235.5 (171.5 to 308.3)	219.3 (162.7 to 302.6)	183.2 (134.9 to 236.2)	186.3 (134.7 to 241.9)	183.3 (131.3 to 247.4)	-18.4 (-37.3 to 4.5)	-20.9 (-42.7 to 6.7)	-16.4 (-37.3 to 10)
		YLDs	22.3 (15.3 to 31.2)	24.5 (17 to 34.6)	20.7 (13.8 to 29.8)	36.3 (24.2 to 53.2)	33 (22.5 to 47.5)	39.1 (25.2 to 58.2)	62.8 (43.9 to 84.8)	34.7 (16.4 to 57.9)	88.7 (61 to 122.2)

Cause	Location	Measure	Age-standardized rate (per 100,000)						% Change (1990 to 2019)		
			1990			2019					
			Both	Female	Male	Both	Female	Male	Both	Female	Male
	Egypt	Incidence	36.5 (32.2 to 41.6)	42 (36.7 to 48.4)	31 (27.2 to 35.3)	65.6 (58.4 to 73.7)	65.1 (57.8 to 73.7)	65.6 (57.6 to 75.1)	79.9 (72.8 to 87.8)	55.2 (47.1 to 64.1)	111.6 (98.9 to 124)
		Prevalence	1379.8 (1254.5 to 1509.1)	1511 (1366.3 to 1674.6)	1249.2 (1128 to 1375.5)	1829.9 (1671 to 1986.6)	1876.1 (1703.8 to 2052.5)	1783.1 (1629.2 to 1946.6)	32.6 (27.9 to 37.3)	24.2 (19.2 to 29.4)	42.7 (35.6 to 49.9)
		Deaths	11.9 (7.8 to 16)	13.3 (7 to 19.3)	10.5 (7.4 to 14.8)	14.4 (8.5 to 21.5)	18.4 (8.1 to 27.5)	11.9 (7.3 to 20)	20.8 (-12.7 to 59)	37.8 (-11.2 to 85.9)	13.5 (-26 to 60.2)
		DALYs	246.4 (167.6 to 322.2)	278.3 (157.8 to 388.2)	214.3 (154.1 to 295.5)	302.8 (184.5 to 442.2)	362.6 (179.5 to 526.2)	259.3 (166.9 to 419.6)	22.8 (-10.1 to 60.6)	30.3 (-12.4 to 73.7)	21 (-17.7 to 64.7)
		YLLs	226.8 (146.8 to 302.3)	255.3 (133.1 to 363)	197.9 (137.9 to 279.7)	268.6 (149.9 to 406.8)	328.3 (144.5 to 492.5)	225.4 (133 to 383.2)	18.4 (-16.9 to 59.8)	28.6 (-17 to 76.8)	13.9 (-28.1 to 62.9)
		YLDs	19.7 (14 to 27.6)	23 (16.3 to 32.4)	16.4 (11.2 to 23.8)	34.2 (23.8 to 48.8)	34.3 (23.7 to 48.4)	33.9 (22.7 to 49.8)	73.5 (54.9 to 94.4)	49.1 (30 to 70.9)	107.1 (79.6 to 140.4)
	Iran (Islamic Republic of)	Incidence	41.7 (37.3 to 46.6)	42.9 (38.2 to 48.5)	40.9 (36.4 to 45.9)	55.6 (50.1 to 61.3)	55.6 (50.2 to 61.3)	55.3 (49.7 to 61)	33.1 (28.6 to 37.9)	29.5 (23.7 to 35.7)	35.4 (30.9 to 40.2)
		Prevalence	1601.5 (1480.1 to 1724.5)	1645.1 (1519.2 to 1776.1)	1561.5 (1442.9 to 1681.1)	1763.5 (1638.3 to 1890.2)	1779.1 (1650.5 to 1907.4)	1748.8 (1624.2 to 1877)	10.1 (7.5 to 13.1)	8.1 (5.1 to 11.5)	12 (9.4 to 14.9)
		Deaths	7.3 (5.9 to 9.2)	6.8 (5.2 to 9.5)	7.9 (6.3 to 9.6)	6.2 (5 to 7.4)	6 (4.7 to 7.2)	6.4 (5.2 to 7.8)	-15.4 (-32.7 to -5.7)	-11.8 (-41.3 to 3)	-18.8 (-31 to -5)
		DALYs	152.7 (124.8 to 180.4)	142.5 (112.7 to 179.7)	162.5 (132 to 193.5)	126.9 (105.5 to 147.6)	120.3 (98.9 to 141.2)	133.4 (110.8 to 157)	-16.9 (-27.6 to -8.9)	-15.6 (-35.6 to -4.4)	-17.9 (-28.3 to -6)
		YLLs	133 (106.8 to 159.6)	122.4 (92.8 to 158.5)	143.2 (114.1 to 172.6)	104.7 (85.2 to 124.8)	99.3 (79.3 to 118.2)	110 (88.5 to 132.5)	-21.3 (-33 to -12.5)	-18.8 (-40.7 to -6.1)	-23.2 (-34.1 to -9.8)
		YLDs	19.7 (14 to 27.1)	20.1 (14.2 to 27.3)	19.3 (13.4 to 26.9)	22.2 (15.4 to 30.8)	21 (14.8 to 28.5)	23.4 (16.1 to 32.6)	12.4 (5.4 to 19.8)	4.3 (-3.9 to 12.9)	21.4 (12.8 to 30.8)

Cause	Location	Measure	Age-standardized rate (per 100,000)						% Change (1990 to 2019)		
			1990			2019					
			Both	Female	Male	Both	Female	Male	Both	Female	Male
	Iraq	Incidence	40.6 (35.8 to 45.9)	42.6 (37.4 to 48.3)	38.6 (33.6 to 43.6)	66.4 (59.4 to 74.2)	63 (56 to 70.5)	70.1 (61.5 to 79.4)	63.4 (56.1 to 70.9)	47.7 (39.2 to 56.6)	81.6 (70.7 to 93.9)
		Prevalence	1587.6 (1445.8 to 1742.3)	1684.7 (1522.6 to 1862.4)	1492.5 (1351.5 to 1645.1)	1933.8 (1778.4 to 2100.7)	1942.9 (1768.4 to 2125.9)	1933.9 (1764.3 to 2134.7)	21.8 (17.1 to 26.4)	15.3 (10.3 to 20.7)	29.6 (23.3 to 36)
		Deaths	15.4 (11.4 to 21.6)	14.6 (10.3 to 21.9)	16.3 (11.7 to 23.7)	14.2 (10.3 to 19.1)	11.8 (8.5 to 16)	17.2 (11.9 to 24.7)	-7.4 (-29.6 to 16)	-19.2 (-43.5 to 12.7)	5.2 (-22.9 to 37.2)
		DALYs	327.4 (249.4 to 430.4)	324.4 (231.5 to 469.3)	332.5 (240.4 to 450.2)	297.5 (225.5 to 383.9)	259.9 (188.8 to 344.4)	340.8 (248.4 to 457.4)	-9.1 (-30.6 to 14.8)	-19.9 (-43 to 10.7)	2.5 (-24.8 to 35.5)
		YLLs	303.6 (227.8 to 405.4)	298.6 (208.2 to 443.6)	310.7 (218.2 to 428.4)	261.1 (188.9 to 348.1)	225.3 (154.7 to 310.5)	302.3 (209.5 to 420.8)	-14 (-36.6 to 11)	-24.5 (-47.8 to 7.6)	-2.7 (-30.6 to 32)
		YLDs	23.8 (17 to 33.3)	25.9 (18.2 to 35.6)	21.8 (14.7 to 31.1)	36.4 (25.1 to 51.4)	34.6 (24.1 to 48.3)	38.6 (25.2 to 56.8)	52.9 (36.7 to 70.7)	33.8 (15.2 to 54.4)	76.8 (51.6 to 106.8)
	Jordan	Incidence	40.5 (35.8 to 45.3)	46.9 (41.5 to 52.8)	34.3 (29.7 to 38.8)	69.3 (62.3 to 76.9)	67.5 (60.3 to 75.4)	70.9 (62.7 to 79.9)	71.2 (60.8 to 81.3)	43.8 (34.1 to 54)	106.6 (89.5 to 124.7)
		Prevalence	1534.8 (1402.5 to 1682.6)	1672.1 (1524.3 to 1844.6)	1402.7 (1270.5 to 1555.7)	1913.5 (1753.7 to 2076.2)	1932.5 (1765.7 to 2115.4)	1900.8 (1739.5 to 2086.4)	24.7 (19.4 to 30.3)	15.6 (9.9 to 21.5)	35.5 (28.5 to 44)
		Deaths	14.3 (11.2 to 17.9)	15.9 (11.8 to 20.8)	12.6 (9.7 to 16.4)	12.6 (9.7 to 15.8)	13.4 (9.9 to 17.5)	11.9 (8.6 to 16.1)	-11.3 (-31.3 to 11.9)	-15.7 (-41.9 to 12.8)	-5.1 (-31.3 to 30.4)
		DALYs	284.3 (225.2 to 351.5)	320.8 (241.8 to 414.9)	248.6 (192.4 to 316.4)	255.5 (200.3 to 316.7)	263.6 (198.2 to 336.1)	247.9 (185.5 to 326.6)	-10.1 (-28.1 to 12.5)	-17.8 (-40.8 to 8.2)	-0.3 (-26.4 to 36.1)
		YLLs	261.8 (203.2 to 326.9)	295 (215.5 to 387.5)	229.5 (174.3 to 296.3)	220.8 (168.7 to 278.9)	229.6 (166.6 to 301.8)	212.3 (153.2 to 291.4)	-15.7 (-34.8 to 9)	-22.2 (-46.7 to 6.1)	-7.5 (-33.5 to 30.8)
		YLDs	22.4 (15.8 to 31.2)	25.8 (18.1 to 35.9)	19 (12.8 to 27.3)	34.8 (23.5 to 49.1)	34 (23.5 to 47.8)	35.6 (23.2 to 52.2)	55.1 (37.4 to 74.4)	31.7 (13.7 to 52.5)	86.9 (59 to 119.1)

Cause	Location	Measure	Age-standardized rate (per 100,000)						% Change (1990 to 2019)		
			1990			2019					
			Both	Female	Male	Both	Female	Male	Both	Female	Male
	Kuwait	Incidence	41.6 (36.9 to 46.9)	48 (42.3 to 54.8)	38.2 (33.1 to 43.5)	63.3 (56 to 71.3)	62.2 (54.7 to 69.7)	63.9 (56.2 to 73.1)	52.4 (37.9 to 67.4)	29.5 (17 to 44.1)	67.3 (48.7 to 88.6)
		Prevalence	1534.5 (1402 to 1679.3)	1693.8 (1537.2 to 1874.3)	1441.3 (1307.9 to 1590)	1797 (1635.8 to 1959)	1843.6 (1681.3 to 2022.9)	1759.6 (1602.1 to 1927.3)	17.1 (10.8 to 23.9)	8.8 (2.7 to 15.9)	22.1 (14.4 to 30.2)
		Deaths	8.9 (7 to 10.8)	10.2 (7.4 to 12.9)	8.1 (6.3 to 10)	5.2 (3.9 to 6.9)	5.3 (3.7 to 7.2)	5.2 (3.8 to 7)	-41.2 (-51.5 to -27.5)	-47.9 (-60 to -26.9)	-36.1 (-48.7 to -20.5)
		DALYs	186.9 (149.1 to 223.3)	220.5 (163 to 274.5)	167.4 (133.2 to 200.5)	116.1 (90.5 to 144)	119.6 (90 to 154.3)	113.4 (86.9 to 144.4)	-37.9 (-47.5 to -24.4)	-45.8 (-57.1 to -25)	-32.2 (-44.8 to -17.5)
		YLLs	166.6 (128.6 to 202.2)	197.4 (140.6 to 249.9)	148.8 (117 to 180.7)	87.4 (65.3 to 112.7)	92 (64.6 to 125.7)	84.3 (60.8 to 112.8)	-47.5 (-57.1 to -34.3)	-53.4 (-64.8 to -31.4)	-43.3 (-55.6 to -28)
		YLDs	20.3 (14.2 to 28.6)	23.1 (15.9 to 32.9)	18.6 (12 to 27.2)	28.6 (19.4 to 41.6)	27.6 (19 to 38.9)	29.1 (18.9 to 44.1)	41.2 (22 to 63.1)	19.3 (1 to 41.3)	56.5 (30 to 90.3)
	Lebanon	Incidence	36.5 (32.2 to 41.1)	39.4 (34.4 to 44.8)	33.7 (29.4 to 38.4)	67.1 (60.1 to 75.1)	63.6 (56.7 to 71.2)	71.3 (63.3 to 80.7)	84 (73.8 to 96)	61.5 (51.3 to 73.9)	111.9 (97.3 to 129.4)
		Prevalence	1460.4 (1333.9 to 1601.6)	1551.4 (1397.8 to 1710.5)	1363.7 (1235.3 to 1502.2)	1861.6 (1712.8 to 2023.9)	1855.9 (1695.5 to 2023.3)	1869.7 (1703.3 to 2055)	27.5 (22.3 to 33.3)	19.6 (14 to 25.4)	37.1 (30.1 to 44.7)
		Deaths	10.1 (7.7 to 13)	9.9 (7.2 to 13.7)	10.4 (7.8 to 13.6)	7 (4.8 to 9.7)	6.8 (4.4 to 9.1)	7.2 (4.2 to 12.2)	-30.6 (-52 to -8.2)	-31 (-53.5 to -9.8)	-30.3 (-59.3 to 11.4)
		DALYs	207.2 (159.4 to 263.1)	208.7 (156.1 to 279.3)	206.2 (155.6 to 268.6)	154 (113.2 to 206.1)	149.8 (106.5 to 195.1)	159 (104.7 to 247.2)	-25.7 (-45.5 to -6.2)	-28.2 (-49.3 to -7)	-22.9 (-49.2 to 13.4)
		YLLs	188.4 (142.5 to 242.9)	188 (136 to 258.2)	189.5 (140.1 to 249.7)	124.1 (84.4 to 173.1)	121.7 (79 to 165)	127 (74.1 to 214.4)	-34.2 (-54.7 to -13.2)	-35.3 (-57.1 to -12.8)	-33 (-59.5 to 6.1)
		YLDs	18.7 (13.1 to 26.1)	20.7 (14.4 to 28.8)	16.7 (11.2 to 24.1)	29.9 (20.3 to 42.2)	28.2 (19.4 to 39.4)	32 (21.2 to 47.1)	59.5 (42 to 80.7)	36.1 (16.6 to 57.8)	92.2 (61.7 to 127)

Cause	Location	Measure	Age-standardized rate (per 100,000)						% Change (1990 to 2019)		
			1990			2019					
			Both	Female	Male	Both	Female	Male	Both	Female	Male
	Libya	Incidence	37.2 (33.1 to 42.1)	40 (35.2 to 45.3)	34.8 (30.6 to 39.8)	62 (55.2 to 69.6)	60.4 (53.4 to 67.7)	63.5 (55.9 to 71.8)	66.8 (59.3 to 74.4)	51.1 (42.6 to 61.2)	82.4 (70.7 to 94.9)
		Prevalence	1477.5 (1345.2 to 1624.7)	1577.5 (1434.2 to 1747.4)	1398.1 (1267.5 to 1551.3)	1805 (1655.4 to 1960.4)	1843.7 (1681.9 to 2014.7)	1770.3 (1611.9 to 1945.3)	22.2 (17.9 to 26.9)	16.9 (11.9 to 22.9)	26.6 (20.9 to 33)
		Deaths	9.3 (6.4 to 12.7)	9.7 (6.4 to 14)	8.9 (6 to 12.8)	9.6 (6.1 to 13.8)	10.4 (5.9 to 15.2)	8.8 (5.5 to 14.3)	3.9 (-25.3 to 38.6)	7.9 (-27.8 to 47.1)	-1.3 (-32.4 to 46.5)
		DALYs	197.8 (141.5 to 260.4)	216.1 (148.4 to 301.2)	182.7 (128 to 255.1)	212.4 (143.3 to 287.5)	234 (148.2 to 326.3)	191.6 (128.5 to 296.2)	7.4 (-19.9 to 41)	8.3 (-24.2 to 44.2)	4.9 (-25.2 to 50.5)
		YLLs	178.5 (121.6 to 242.5)	194.4 (126.1 to 277)	165.3 (111.3 to 234.1)	182.4 (114.1 to 258)	204 (118.9 to 295.4)	161.5 (98.6 to 263.6)	2.2 (-27.6 to 39.7)	4.9 (-31 to 45.3)	-2.3 (-34.7 to 46.4)
		YLDs	19.3 (13.5 to 26.7)	21.7 (15.1 to 29.6)	17.3 (11.5 to 25.3)	30 (20.8 to 42.2)	30 (20.7 to 41.4)	30.1 (20 to 44.1)	55.4 (37.5 to 74.4)	38.4 (18.7 to 59)	73.6 (46.4 to 109.7)
	Morocco	Incidence	28.2 (24.8 to 32.2)	30 (25.9 to 34.7)	26.4 (23.3 to 30.2)	59.8 (53.3 to 67.4)	56.3 (49.6 to 64.2)	63.5 (55.7 to 72.4)	112.2 (103.1 to 123.4)	87.9 (76.2 to 100.5)	140.5 (127 to 158.5)
		Prevalence	1303.9 (1183.3 to 1438)	1381.4 (1248.7 to 1538.1)	1223.1 (1095.3 to 1352.2)	1745.3 (1604.3 to 1898.4)	1755.1 (1600.6 to 1923.9)	1738.3 (1577.6 to 1899.6)	33.8 (28.7 to 39.9)	27 (21.1 to 33.8)	42.1 (34.9 to 50)
		Deaths	8.5 (6.1 to 12.2)	7.8 (5.3 to 12.2)	9.3 (6.4 to 14.6)	10.8 (7.6 to 14.7)	10.6 (7.3 to 14.8)	10.9 (7.3 to 16.4)	27.1 (-6.2 to 60.6)	35.6 (-7.8 to 83.2)	17.7 (-15.1 to 56.6)
		DALYs	179.3 (133.2 to 243.1)	174.9 (125.2 to 249.8)	185 (131.8 to 264.3)	228.5 (166.7 to 298.9)	233.3 (168.5 to 316.4)	224.4 (154 to 315.4)	27.4 (-5 to 59.7)	33.4 (-7.6 to 78.9)	21.4 (-12 to 58)
		YLLs	164 (118.1 to 226.4)	158 (109.8 to 230.5)	171.4 (116.9 to 250.7)	198 (138.1 to 266.1)	204.1 (139.7 to 284.6)	192.6 (123.7 to 283.9)	20.7 (-12.7 to 54.9)	29.2 (-14.6 to 80.4)	12.4 (-20.8 to 50.6)
		YLDs	15.3 (10.6 to 21.7)	16.9 (11.7 to 23.5)	13.6 (9 to 19.9)	30.4 (21 to 43.3)	29.2 (20 to 41.2)	31.8 (21.1 to 46.8)	99.2 (77.3 to 125.2)	72.6 (50.1 to 100)	134 (97.4 to 179.3)

Cause	Location	Measure	Age-standardized rate (per 100,000)						% Change (1990 to 2019)		
			1990			2019					
			Both	Female	Male	Both	Female	Male	Both	Female	Male
	Oman	Incidence	31.3 (27.6 to 35.3)	33.1 (28.9 to 37.8)	30.2 (26.2 to 34.5)	63.3 (56.6 to 70.9)	59.6 (52.8 to 67.2)	66.7 (58.8 to 75.2)	102.1 (90 to 114)	80.2 (67.8 to 94.6)	121.1 (104.1 to 137.4)
		Prevalence	1352 (1231.5 to 1474.9)	1448.4 (1301.3 to 1602.3)	1299.3 (1180.7 to 1428.3)	1790.8 (1645.5 to 1934.3)	1814.8 (1652.7 to 1984.1)	1805.3 (1655.1 to 1962.6)	32.4 (27.3 to 38.1)	25.3 (18.6 to 32.4)	38.9 (32.2 to 46.6)
		Deaths	5.5 (4 to 7.6)	5.3 (3.7 to 7.3)	6.3 (4.4 to 9.2)	6.8 (5.2 to 8.5)	6.5 (4.7 to 8.5)	7.4 (5.5 to 9.7)	22.1 (-14.1 to 68.4)	23.9 (-16.3 to 74.5)	17.3 (-25.1 to 75.3)
		DALYs	120.2 (90 to 160)	121.3 (89.8 to 161.1)	124 (89.3 to 177.7)	142.4 (115.4 to 171.5)	145.4 (115.2 to 179.3)	144.3 (113.6 to 177)	18.5 (-9.7 to 53.9)	19.9 (-10.9 to 58.8)	16.4 (-19.2 to 63.6)
		YLLs	100.9 (71.4 to 140.5)	99.9 (69.7 to 138.3)	106.6 (72.4 to 157.5)	106.9 (82.3 to 132.8)	109.9 (80.8 to 142.1)	108.3 (81.3 to 139.9)	5.9 (-23.7 to 45.9)	10.1 (-23.5 to 57.2)	1.6 (-32.9 to 54.4)
		YLDs	19.3 (13.7 to 26.4)	21.5 (15 to 29.7)	17.4 (11.9 to 24.6)	35.5 (24.5 to 49.7)	35.5 (24.7 to 49.3)	36 (23.6 to 52.1)	84.6 (64.3 to 107.6)	65.5 (44.3 to 90.7)	106.9 (76.3 to 144.1)
	Palestine	Incidence	41.1 (36 to 45.9)	41.8 (36.3 to 47.4)	40.2 (35.1 to 45.6)	65.9 (58.6 to 73.7)	61.2 (54.2 to 69.2)	71.2 (62.5 to 80.5)	60.5 (52.7 to 69.6)	46.6 (36.6 to 57.7)	77.4 (63 to 91.6)
		Prevalence	1514.6 (1379.5 to 1661.5)	1574.8 (1426.9 to 1736.9)	1444.2 (1312.2 to 1583.4)	1827.4 (1671.1 to 1987.6)	1807.9 (1647.6 to 1979.8)	1863.4 (1695.1 to 2032)	20.6 (16 to 25.7)	14.8 (9.3 to 20.5)	29 (22.2 to 36.1)
		Deaths	14.5 (10.6 to 19.5)	13.4 (9.6 to 18)	16.1 (11.6 to 22)	10.9 (8.6 to 13.7)	10 (7.7 to 12.6)	12.6 (9.6 to 15.9)	-24.7 (-42.5 to -2.8)	-25.4 (-43.7 to 0.8)	-21.5 (-42.3 to 4.3)
		DALYs	303.1 (224.6 to 402.2)	289.7 (208.2 to 384.2)	321.1 (233.1 to 429.8)	233.2 (186.4 to 286.1)	217.7 (172.9 to 270)	255.3 (200.8 to 313.8)	-23.1 (-40.4 to -1.2)	-24.9 (-42.7 to -1.2)	-20.5 (-40.4 to 4.4)
		YLLs	281.2 (204.3 to 377.3)	266.4 (187.4 to 362)	300.7 (213.9 to 410.1)	200.8 (156.1 to 251.5)	187.2 (144.2 to 238.5)	220.5 (168.2 to 280.5)	-28.6 (-46 to -5.7)	-29.7 (-47.8 to -4.1)	-26.7 (-46 to - 1.3)
		YLDs	21.9 (15.3 to 30.5)	23.3 (16.2 to 32.4)	20.4 (13.9 to 29)	32.4 (22.6 to 45.7)	30.4 (20.8 to 42.7)	34.8 (23.6 to 51.6)	47.6 (31.5 to 65.7)	30.9 (12.3 to 51.4)	70.2 (44.1 to 101.7)

Cause	Location	Measure	Age-standardized rate (per 100,000)						% Change (1990 to 2019)		
			1990			2019					
			Both	Female	Male	Both	Female	Male	Both	Female	Male
	Qatar	Incidence	46.6 (41.4 to 52.7)	54.1 (47.2 to 61.4)	44.1 (38.6 to 50.8)	77.2 (69 to 86.2)	71.7 (63.7 to 80)	79.4 (70.3 to 89.3)	65.5 (56.6 to 75.2)	32.5 (23.8 to 41.6)	80.1 (68.6 to 92.1)
		Prevalence	1604.7 (1463.9 to 1758.8)	1794.5 (1625.8 to 1978.6)	1517.5 (1370.9 to 1674.1)	1995.4 (1831.9 to 2172.6)	2026.4 (1843.7 to 2208.1)	1989.7 (1816.2 to 2176.6)	24.3 (19.1 to 29.8)	12.9 (7.4 to 18)	31.1 (24.5 to 37.6)
		Deaths	16.9 (12.4 to 29)	19.4 (13.2 to 37.8)	15.6 (10.9 to 24.8)	15.8 (12 to 20.9)	24.2 (17.6 to 32)	13.1 (9.5 to 17.7)	-6.5 (-41.4 to 27.2)	25 (-34.9 to 80.1)	-16.5 (-44.1 to 18.6)
		DALYs	303.4 (228.4 to 485.6)	367.7 (261.1 to 674.3)	270 (194.5 to 396.7)	256.9 (199.9 to 333.8)	380.9 (289.4 to 495.7)	215 (162.2 to 281.5)	-15.3 (-43.4 to 12.8)	3.6 (-41 to 46.3)	-20.4 (-43.7 to 10.4)
		YLLs	278.5 (202.7 to 456)	338.5 (233.2 to 645.4)	247.4 (173.9 to 375.9)	218.4 (164.8 to 290.8)	344.5 (252.3 to 457.5)	175.6 (127.1 to 238.3)	-21.6 (-48 to 7.3)	1.8 (-44.1 to 47.7)	-29 (-51.5 to 1.7)
		YLDs	24.9 (17.3 to 35.4)	29.2 (20.2 to 40.7)	22.6 (15.3 to 32.9)	38.5 (25.8 to 55.6)	36.4 (25.3 to 50.9)	39.5 (25.4 to 58.6)	54.9 (33.8 to 76.4)	24.6 (7 to 44.5)	74.4 (47.1 to 104.5)
	Saudi Arabia	Incidence	42.2 (37.4 to 47.8)	45.5 (40 to 51.8)	40.2 (35.3 to 45.6)	78.4 (70.9 to 86.3)	71.3 (63.7 to 80.1)	83 (73.8 to 92.3)	85.5 (73.7 to 97.4)	56.7 (44.3 to 69.5)	106.4 (91.4 to 123.2)
		Prevalence	1514 (1385 to 1660.7)	1633.7 (1479.5 to 1806.5)	1436.9 (1303.2 to 1575.5)	2068.4 (1912.4 to 2240.2)	2064.1 (1887.9 to 2261.1)	2075.1 (1907.8 to 2259.6)	36.6 (30.6 to 43.5)	26.3 (19.2 to 34.4)	44.4 (36.4 to 52.4)
		Deaths	15.1 (11 to 20.4)	13.5 (9.9 to 18.4)	16.8 (11.7 to 23)	16.6 (12.1 to 21.7)	16.6 (11.6 to 22.9)	16.7 (12 to 22.8)	9.3 (-23.4 to 51)	22.6 (-18.9 to 71.5)	-0.6 (-35.4 to 48.4)
		DALYs	324.3 (236.1 to 439.5)	306.8 (224.1 to 413.6)	341.5 (240.6 to 469.7)	349.6 (261.7 to 456.8)	360.6 (264 to 487.9)	343.3 (251.9 to 451.6)	7.8 (-23.2 to 45.8)	17.5 (-20 to 63.2)	0.5 (-32.8 to 46.1)
		YLLs	301.2 (212.2 to 414)	280.9 (198.4 to 389.4)	320.3 (218.5 to 449.3)	310 (225 to 415.3)	322.8 (225.6 to 446.9)	302.4 (214 to 414.5)	2.9 (-29.5 to 43.4)	14.9 (-26.2 to 64)	-5.6 (-38.7 to 42.4)
		YLDs	23.1 (16.5 to 32.1)	25.9 (18.3 to 35.8)	21.2 (14.2 to 30.5)	39.7 (27.1 to 56.4)	37.8 (25.9 to 52.8)	40.9 (27 to 60.3)	71.6 (51.9 to 95.5)	46.1 (24.9 to 72.4)	92.7 (64.7 to 123.4)

Cause	Location	Measure	Age-standardized rate (per 100,000)						% Change (1990 to 2019)		
			1990			2019					
			Both	Female	Male	Both	Female	Male	Both	Female	Male
	Sudan	Incidence	28.2 (24.9 to 31.9)	30.2 (26.2 to 34.8)	26.5 (23.2 to 30.3)	52 (45.9 to 58.8)	49.9 (43.5 to 56.3)	53.8 (46.7 to 62)	84.5 (75.7 to 93)	65.3 (55.8 to 74.5)	103.1 (89.7 to 116.6)
		Prevalence	1307 (1182.5 to 1437.7)	1382.2 (1236.4 to 1539.9)	1232.2 (1102.4 to 1365.5)	1618.8 (1482.4 to 1761.4)	1634.2 (1480.8 to 1791.2)	1596.6 (1446.6 to 1754.2)	23.9 (18.9 to 29)	18.2 (12.6 to 23.6)	29.6 (23.4 to 36.5)
		Deaths	7.5 (5 to 11.2)	7 (4.4 to 11.1)	8.1 (5.4 to 13.2)	8 (5.1 to 12.6)	7.7 (4.8 to 11.5)	8.4 (4.8 to 15.3)	6.8 (-29.4 to 60.7)	10.4 (-35.3 to 58.1)	2.8 (-32.8 to 76.2)
		DALYs	167.3 (115.9 to 236.8)	163.2 (106.6 to 244.5)	171.4 (116.7 to 262.4)	178 (120.4 to 262.2)	175.5 (116.9 to 250)	180 (110.1 to 299.1)	6.4 (-27 to 52.3)	7.5 (-33 to 51.1)	5 (-29.2 to 71.4)
		YLLs	152.7 (101.4 to 221.7)	146.9 (92 to 226.5)	158.5 (104.2 to 247.5)	152.8 (95.3 to 237.4)	150.6 (92.1 to 221.2)	154.5 (87.4 to 273.2)	0 (-34.7 to 50.4)	2.6 (-40.8 to 49.7)	-2.5 (-38.8 to 69)
		YLDs	14.6 (10 to 20.6)	16.4 (11.4 to 22.8)	13 (8.5 to 18.6)	25.3 (17.3 to 35.5)	24.8 (17.4 to 34.8)	25.5 (16.6 to 37.9)	72.8 (53 to 94.7)	51.8 (30.3 to 76.2)	96.2 (63.7 to 131.8)
	Syrian Arab Republic	Incidence	36.8 (32.4 to 42)	41.1 (35.8 to 46.9)	33 (28.7 to 38)	60.5 (54.5 to 67.8)	59.8 (53.1 to 67.1)	60.9 (54 to 69.5)	64.3 (52.6 to 75.5)	45.6 (35.5 to 57.1)	84.5 (65.8 to 103.5)
		Prevalence	1455.2 (1334.5 to 1597.1)	1560.5 (1415.6 to 1741.4)	1356.7 (1236.7 to 1503.8)	1736.3 (1605.8 to 1896.4)	1763.7 (1614.6 to 1940.1)	1703.3 (1562.5 to 1871.5)	19.3 (13.9 to 24.7)	13 (7.4 to 18.8)	25.5 (18.5 to 33.1)
		Deaths	10.3 (7.6 to 13.7)	10.2 (7.4 to 13.7)	10.5 (7.4 to 14.6)	8 (5.7 to 10.7)	8.8 (6.2 to 11.8)	7.5 (5.2 to 10.4)	-22.7 (-45.7 to 6.3)	-13.6 (-40.1 to 17.3)	-27.9 (-54.2 to 6.4)
		DALYs	223.4 (167 to 292)	221.2 (164.2 to 290.1)	225.2 (161.7 to 303.3)	172 (126.9 to 227.5)	179.1 (129.9 to 237.8)	167.7 (120.7 to 226)	-23 (-44 to 4)	-19 (-40.9 to 9.1)	-25.5 (-49 to 7.6)
		YLLs	203.5 (147.6 to 270)	198.6 (143.6 to 265.7)	207.7 (143.7 to 284)	143.7 (100 to 198.3)	151 (104.8 to 208.5)	139.5 (96.1 to 194.2)	-29.4 (-51.1 to -0.3)	-24 (-48 to 7.3)	-32.9 (-56.8 to 1.1)
		YLDs	20 (14.1 to 27.7)	22.6 (15.9 to 31.4)	17.5 (12 to 25.2)	28.2 (19.5 to 40.2)	28.1 (19.3 to 39.6)	28.3 (18.7 to 41.4)	41.5 (24.6 to 61.3)	24.2 (5.6 to 43.1)	61.4 (34.8 to 94.5)

Cause	Location	Measure	Age-standardized rate (per 100,000)						% Change (1990 to 2019)		
			1990			2019					
			Both	Female	Male	Both	Female	Male	Both	Female	Male
	Tunisia	Incidence	35.6 (31.4 to 40.4)	37.9 (32.8 to 43.5)	33.5 (29.3 to 38.2)	63.2 (56.1 to 70.8)	60.3 (52.9 to 68.1)	66.4 (58.8 to 74.9)	77.7 (68.6 to 86.9)	59.3 (48.8 to 70.5)	98.1 (84.5 to 112.7)
		Prevalence	1408.1 (1277.1 to 1538.3)	1486.7 (1335.6 to 1642.7)	1330 (1194.2 to 1468.7)	1762.1 (1605.5 to 1918.4)	1767 (1604.4 to 1931.4)	1760.2 (1594.8 to 1921)	25.1 (20.1 to 30.2)	18.9 (12.8 to 25)	32.3 (26.1 to 39.2)
		Deaths	6.4 (4.7 to 8.5)	6.1 (4.5 to 8.7)	6.7 (4.7 to 9.2)	6.4 (4.3 to 9.2)	6.2 (4 to 9.1)	6.7 (4.3 to 10)	0.5 (-31.5 to 37.8)	1.5 (-32.7 to 40)	-0.8 (-36.7 to 43.9)
		DALYs	133.8 (101.8 to 172.1)	133 (100.7 to 179.5)	135.1 (98.9 to 177.5)	140.8 (101.8 to 189.1)	136.9 (96.2 to 186)	145.3 (102.3 to 205.3)	5.2 (-23.3 to 40.1)	2.9 (-26.1 to 37)	7.6 (-25.8 to 50.3)
		YLLs	115.9 (85 to 153.1)	114.4 (83 to 163.2)	118 (82.7 to 160.8)	112.2 (74.5 to 157.6)	110.5 (69.9 to 159.2)	114.5 (73.1 to 171)	-3.2 (-35.4 to 36.6)	-3.4 (-37.2 to 36.1)	-3 (-38.6 to 44)
		YLDs	17.9 (12.4 to 25)	18.6 (12.7 to 25.6)	17.1 (11.6 to 24.7)	28.5 (19.3 to 40.5)	26.4 (18 to 36.2)	30.9 (20.1 to 45.5)	59.6 (41 to 82)	41.9 (22.7 to 65.9)	80 (53.1 to 114.7)
	Turkey	Incidence	30.8 (27.8 to 34.4)	30 (26.8 to 33.9)	31.7 (28.5 to 35.4)	59.8 (53.4 to 66.8)	55.4 (49.2 to 62.6)	64.9 (57 to 73.4)	94.1 (78.5 to 110.5)	84.6 (68.8 to 102.8)	104.3 (85.5 to 126.9)
		Prevalence	1330.9 (1221.6 to 1460.6)	1369.4 (1242.2 to 1521.8)	1297.4 (1179.2 to 1426.8)	1670.5 (1539.1 to 1817.4)	1644.7 (1501.9 to 1804.6)	1710.5 (1560.4 to 1873)	25.5 (18.8 to 32.9)	20.1 (12.9 to 28)	31.8 (23.4 to 40.7)
		Deaths	11.4 (8.4 to 16.8)	9.7 (7.1 to 15.5)	13.4 (9.2 to 20.8)	7.6 (5.7 to 9.8)	7.2 (5.3 to 9.5)	8 (5.9 to 10.5)	-33.5 (-57.4 to -9.5)	-26 (-55.3 to 2.5)	-40.3 (-63.6 to -10.9)
		DALYs	234.7 (176.4 to 324.6)	205 (155.2 to 306.7)	269 (189.8 to 389.1)	159.3 (124.4 to 201.1)	148.8 (115.6 to 188.7)	171.1 (130.7 to 218.5)	-32.1 (-53.2 to -11.1)	-27.4 (-52.4 to -2.8)	-36.4 (-58.5 to -10.3)
		YLLs	217 (160.5 to 307.1)	187.3 (137.2 to 286.1)	251.2 (173.7 to 374.9)	130.8 (98.6 to 170.8)	122.7 (89.9 to 161.3)	139.7 (101.5 to 184.7)	-39.7 (-60.2 to -18.4)	-34.5 (-58.8 to -9.2)	-44.4 (-65.3 to -17.6)
		YLDs	17.7 (12.6 to 23.9)	17.7 (12.5 to 24.2)	17.8 (12.4 to 24.6)	28.5 (19.3 to 40.7)	26.1 (17.9 to 36.7)	31.4 (20.6 to 45.2)	61.1 (41 to 85.2)	47.5 (25.4 to 75.9)	76.7 (47.2 to 110.8)

Cause	Location	Measure	Age-standardized rate (per 100,000)						% Change (1990 to 2019)		
			1990			2019					
			Both	Female	Male	Both	Female	Male	Both	Female	Male
	United Arab Emirates	Incidence	49 (43.3 to 54.9)	52.7 (46.2 to 59.8)	47 (41.1 to 53.1)	75.4 (67.5 to 83.4)	72.6 (64.9 to 80.8)	77.3 (68.6 to 86.5)	53.9 (46.5 to 61.2)	37.7 (30.1 to 45.4)	64.6 (54.6 to 74.8)
		Prevalence	1632.2 (1483.8 to 1784.2)	1778.1 (1606.5 to 1964.1)	1567.7 (1429.3 to 1721.4)	1966 (1805.7 to 2145.5)	2012.6 (1849.4 to 2196.8)	1962.4 (1782.5 to 2156.4)	20.5 (15.8 to 25.3)	13.2 (8.4 to 17.9)	25.2 (19.3 to 31.4)
		Deaths	20.4 (12.3 to 27)	20.9 (11.6 to 29.9)	20.2 (11.9 to 28.1)	15.9 (9.3 to 25.5)	17.1 (8.6 to 24.5)	15.4 (8.9 to 28)	-22.1 (-47.4 to 10.7)	-17.9 (-52 to 18.6)	-24 (-49.3 to 20.1)
		DALYs	412 (272.4 to 528.9)	427.7 (259.4 to 603.8)	405.2 (253.1 to 559.8)	337.7 (215.6 to 525)	360.1 (204.1 to 505.4)	329.3 (207.2 to 570.5)	-18 (-43.2 to 15.5)	-15.8 (-49.4 to 21.1)	-18.7 (-44.5 to 24.3)
		YLLs	385.7 (243.9 to 507)	398.6 (230.2 to 574.8)	380.4 (229.9 to 537.5)	298.9 (178.3 to 488.2)	322.7 (157.2 to 472)	289.7 (167.3 to 527.7)	-22.5 (-49 to 12.8)	-19 (-54.3 to 20.3)	-23.8 (-50.8 to 21.2)
		YLDs	26.3 (18 to 37.7)	29.1 (20.2 to 40.8)	24.8 (16.1 to 36.5)	38.8 (25.5 to 55.8)	37.4 (25.7 to 53)	39.6 (24.9 to 58)	47.3 (30.2 to 65.8)	28.4 (11.4 to 48.4)	59.8 (36.8 to 86.8)
	Yemen	Incidence	27.3 (24 to 31.1)	28.3 (24.6 to 32.9)	26.3 (22.7 to 30.2)	47 (41.1 to 54.2)	45.4 (38.5 to 54.1)	48.6 (41.8 to 56.2)	72.2 (61.6 to 86.7)	60.3 (44.7 to 86.4)	84.9 (72.5 to 96.3)
		Prevalence	1242.7 (1127.8 to 1364.5)	1304.8 (1173.5 to 1453)	1182.9 (1073.1 to 1301.5)	1483.5 (1351.3 to 1623.3)	1505.4 (1356.4 to 1671.5)	1462.5 (1327.7 to 1606.7)	19.4 (14.3 to 25.4)	15.4 (8.6 to 24.7)	23.6 (17.5 to 29.7)
		Deaths	7.2 (4.9 to 10.7)	6.3 (4.1 to 10.7)	8.7 (5.7 to 13.4)	6.9 (4.8 to 9.4)	6.2 (4.2 to 9)	7.7 (5.2 to 11.1)	-4.5 (-28.1 to 27.5)	-3 (-31.6 to 33.1)	-11.5 (-34.7 to 22.8)
		DALYs	163.6 (113.2 to 239.4)	150.2 (102.1 to 240.5)	183.2 (124.5 to 268.2)	157.2 (113.5 to 211.4)	147.4 (106.2 to 206.2)	167.8 (116.7 to 231.3)	-3.9 (-26.4 to 27.7)	-1.9 (-29.4 to 32.7)	-8.4 (-32.4 to 26)
		YLLs	148.6 (99.8 to 222.8)	134 (86.1 to 225.4)	169.7 (111.5 to 254.2)	134 (91.3 to 188)	123.8 (83 to 180.3)	144.9 (96.3 to 207.8)	-9.9 (-33.5 to 23.7)	-7.6 (-35.2 to 30.9)	-14.7 (-39.2 to 20.6)
		YLDs	14.9 (10.6 to 20.7)	16.3 (11.5 to 22.4)	13.5 (9.2 to 19.1)	23.2 (16.1 to 33)	23.6 (16.3 to 33.8)	23 (15.1 to 33.7)	55.7 (36.5 to 76.7)	44.9 (20.8 to 75.8)	70.3 (45.6 to 98.5)

Cause	Location	Measure	Age-standardized rate (per 100,000)						% Change (1990 to 2019)		
			1990			2019					
			Both	Female	Male	Both	Female	Male	Both	Female	Male
Chronic kidney disease due to hypertension	North Africa and Middle East	Incidence	21.1 (19.2 to 23.1)	21.8 (19.8 to 24)	20.5 (18.7 to 22.4)	36.6 (33.6 to 39.6)	34.4 (31.7 to 37.1)	38.7 (35.6 to 42.1)	73 (68.4 to 77.7)	57.2 (52.2 to 62.8)	89.1 (84 to 94.5)
		Prevalence	406.7 (374.5 to 443.7)	424.1 (388.1 to 465.1)	389.2 (358.3 to 422.6)	698.8 (643.8 to 756.9)	670.4 (617.2 to 726)	727.6 (667.4 to 791)	71.8 (67.9 to 76)	58.1 (53.5 to 62.7)	87 (82.1 to 92.2)
		Deaths	11.9 (9.6 to 15.9)	10.6 (8.4 to 15.3)	13.5 (10.6 to 18.3)	10.6 (8.5 to 12.8)	10 (7.9 to 12.4)	11.2 (9 to 14.1)	-11.3 (-29.5 to 6.3)	-5.7 (-32.3 to 13)	-17 (-36.6 to 6.8)
		DALYs	222.4 (183.4 to 282.6)	203.6 (163.5 to 271.3)	242.6 (197.8 to 311.6)	203.3 (165.8 to 243)	192.3 (154.2 to 238.2)	214.1 (173.9 to 265.3)	-8.6 (-24.8 to 7.6)	-5.6 (-27.9 to 12.4)	-11.7 (-30.7 to 11)
		YLLs	204.6 (166.6 to 264.8)	185.7 (146.3 to 252.8)	224.9 (179.6 to 296.2)	174.6 (139 to 215.2)	166.4 (130.4 to 210.1)	182.6 (144.3 to 233.2)	-14.7 (-31 to 2.5)	-10.4 (-33.2 to 8.6)	-18.8 (-37.4 to 4.8)
		YLDs	17.8 (12.9 to 23.7)	17.9 (13.1 to 23.9)	17.7 (12.3 to 23.9)	28.7 (20.6 to 38.7)	25.9 (18.6 to 34.2)	31.6 (21.8 to 43.6)	61.4 (53.2 to 70)	44.6 (36.4 to 53.3)	78.1 (66.2 to 89.6)
	Afghanistan	Incidence	19.4 (17.1 to 21.9)	20.1 (17.4 to 23.1)	18.8 (16.4 to 21.3)	31.4 (27.7 to 35.4)	29.6 (25.6 to 34)	33.3 (29.1 to 37.7)	61.3 (54.8 to 68.7)	47.4 (39.9 to 57.2)	77 (68.2 to 86.9)
		Prevalence	359.2 (320.9 to 403)	386.8 (342.6 to 436.6)	331.7 (292.6 to 372)	580.1 (519.5 to 650.4)	572.1 (507.2 to 650.1)	590.2 (520.7 to 661.8)	61.5 (55.2 to 68.6)	47.9 (40.9 to 56.2)	77.9 (68 to 88.1)
		Deaths	18.3 (12.6 to 28.8)	16 (10.7 to 28.3)	20.3 (13.5 to 32.6)	16.4 (11.5 to 26.1)	15.2 (10 to 25.8)	17.6 (11.3 to 28.7)	-10.3 (-32.6 to 15.1)	-5 (-38.9 to 34.3)	-13.6 (-34.2 to 9)
		DALYs	365.5 (256.1 to 525.7)	343.7 (237 to 536)	381.6 (259.4 to 569.1)	322.5 (231.9 to 469.8)	315.3 (216.5 to 503)	330.3 (222.2 to 506.6)	-11.8 (-34.8 to 15.5)	-8.3 (-39.4 to 29.2)	-13.4 (-35.2 to 11.2)
		YLLs	348.2 (239.8 to 510.4)	325.1 (219.9 to 519.8)	365.9 (244.2 to 556.6)	295.7 (207.1 to 442.9)	288.7 (188.7 to 479.1)	302.9 (197.6 to 478.3)	-15.1 (-37.7 to 12.8)	-11.2 (-43.3 to 28.6)	-17.2 (-38.9 to 8.1)
		YLDs	17.3 (12.2 to 23.8)	18.6 (12.9 to 26)	15.7 (10.6 to 22.1)	26.8 (19 to 36.9)	26.6 (18.7 to 36.7)	27.4 (18.4 to 39.6)	54.5 (37.1 to 73.5)	42.7 (21.9 to 66.7)	74.4 (47.6 to 106.6)

Cause	Location	Measure	Age-standardized rate (per 100,000)						% Change (1990 to 2019)		
			1990			2019					
			Both	Female	Male	Both	Female	Male	Both	Female	Male
	Algeria	Incidence	21.9 (19.5 to 24.6)	23.1 (20.2 to 26.4)	20.5 (18 to 23.2)	37.1 (33.3 to 41.2)	35.2 (31.3 to 39.4)	38.8 (34.1 to 43.5)	69.4 (62.5 to 76.8)	52.2 (44.5 to 60.8)	89.7 (79 to 101.8)
		Prevalence	416.5 (372.9 to 466.9)	449.6 (395.2 to 513.5)	385.3 (340.4 to 436.5)	711.5 (639.5 to 789.5)	695.3 (618.1 to 782.1)	722.9 (639.5 to 807.3)	70.8 (63.6 to 78)	54.6 (47.1 to 63.1)	87.6 (76.3 to 99.4)
		Deaths	14.1 (10.2 to 19.8)	13.8 (9.1 to 23.2)	14.3 (10.4 to 19)	11.6 (8.5 to 15.4)	13.2 (9.1 to 18.9)	10.6 (7.8 to 14.6)	-17.3 (-36.2 to 8.3)	-4.9 (-29.7 to 28.9)	-25.3 (-45.2 to 5.4)
		DALYs	231.7 (169.3 to 325.6)	230.7 (156.5 to 373.2)	232.8 (172.3 to 313.1)	195.5 (148.1 to 254.4)	210.5 (153.4 to 291.8)	184.7 (139.2 to 246.7)	-15.6 (-35.1 to 8.7)	-8.8 (-32.1 to 22.9)	-20.7 (-40.8 to 8.5)
		YLLs	214.1 (152.1 to 308.5)	212.2 (138.2 to 354.8)	216 (157.4 to 296.3)	166.3 (121.1 to 226.2)	183.3 (126.4 to 264.5)	153.7 (109.3 to 215.4)	-22.3 (-41.4 to 3.2)	-13.6 (-37.9 to 19.7)	-28.9 (-49.2 to 0.8)
		YLDs	17.7 (12.2 to 24.8)	18.5 (12.7 to 26.3)	16.8 (11.4 to 23.5)	29.2 (19.9 to 41.6)	27.2 (18.3 to 38.4)	31 (20.5 to 45.4)	65.5 (46.9 to 84.6)	46.8 (25.2 to 70.6)	84.4 (54.6 to 114.9)
	Bahrain	Incidence	25.5 (22.9 to 28.4)	26.4 (23.4 to 29.7)	25.1 (22.2 to 28.2)	42.4 (38.3 to 46.8)	38.1 (34.3 to 42.3)	45.8 (40.8 to 51.4)	66.4 (58 to 74.9)	44.4 (36.7 to 52.9)	82.3 (70.9 to 94.3)
		Prevalence	472.5 (426.7 to 527.1)	500.2 (445 to 560.3)	449.3 (396.7 to 504)	806.5 (726.9 to 889.9)	750.4 (672.4 to 831.9)	859.6 (764.3 to 959.3)	70.7 (62.5 to 78.8)	50 (41.6 to 58.8)	91.3 (79.3 to 102.8)
		Deaths	13.5 (10.4 to 17.2)	12.7 (9.3 to 16.5)	14.7 (10.6 to 20.4)	12.6 (9.6 to 16)	11.7 (8.6 to 15.3)	13.5 (10 to 17.9)	-6.5 (-27.2 to 18.6)	-7.7 (-32.3 to 23.9)	-7.8 (-30.5 to 21.5)
		DALYs	227.7 (180.4 to 287.4)	222.7 (166.5 to 287.1)	237.6 (181 to 322.4)	208.8 (164.2 to 258.8)	193.5 (147.4 to 246.2)	223.5 (171.7 to 285.8)	-8.3 (-26.8 to 13.2)	-13.1 (-35 to 14.1)	-5.9 (-27.5 to 22)
		YLLs	207.5 (160.2 to 266.2)	202.3 (147.3 to 268.6)	217.3 (159.3 to 303.7)	174.5 (132.2 to 224.7)	164.4 (118.9 to 216.3)	184.9 (136.3 to 246.2)	-15.9 (-34.5 to 8)	-18.7 (-41.6 to 10.7)	-14.9 (-36.7 to 14.2)
		YLDs	20.2 (14.3 to 28)	20.4 (14 to 28)	20.3 (14 to 28.8)	34.3 (23.6 to 48.3)	29.1 (20 to 40.8)	38.6 (25.8 to 55.6)	70.2 (51 to 92.1)	42.5 (21.8 to 64.2)	90.1 (63.2 to 122.2)

Cause	Location	Measure	Age-standardized rate (per 100,000)						% Change (1990 to 2019)		
			1990			2019					
			Both	Female	Male	Both	Female	Male	Both	Female	Male
	Egypt	Incidence	22.1 (19.5 to 24.6)	24.7 (21.5 to 27.9)	19.6 (17.2 to 22.1)	38.8 (34.5 to 43.1)	37.2 (32.9 to 41.5)	39.7 (34.9 to 44.7)	75.6 (68.7 to 82.4)	51.1 (43.9 to 58.8)	102.9 (91.8 to 112.8)
		Prevalence	416.4 (372.2 to 466.8)	465.8 (411.6 to 532.9)	366.3 (324.9 to 413.1)	746.9 (670.9 to 827.8)	735.6 (655.2 to 823.3)	748.8 (666.8 to 843.6)	79.3 (72.1 to 86.2)	57.9 (50.4 to 65.6)	104.4 (93.2 to 116.5)
		Deaths	13.9 (9.2 to 18.3)	14.6 (8 to 20.8)	13.1 (9.5 to 17.9)	15.5 (9.4 to 22.1)	19.3 (8.8 to 28.7)	13.3 (8.4 to 22)	11.5 (-17.5 to 46)	32.6 (-13.2 to 79.9)	1.4 (-32.3 to 44)
		DALYs	253 (175.1 to 325.7)	268.4 (155.4 to 382)	237.3 (174.8 to 313.1)	289.4 (181.5 to 407.2)	334.5 (164.2 to 494.1)	260.4 (169.7 to 418.4)	14.4 (-15.2 to 48.6)	24.6 (-15.8 to 66.8)	9.7 (-23.6 to 53)
		YLLs	234.6 (155.8 to 308.3)	248.5 (137.1 to 362.2)	220.4 (158.2 to 296.9)	257 (149.6 to 375.1)	304.5 (136.2 to 465.6)	226.3 (135.2 to 384.8)	9.5 (-22.2 to 46.4)	22.6 (-20.4 to 69.1)	2.7 (-33.8 to 48.2)
		YLDs	18.4 (13 to 24.9)	19.9 (13.8 to 27.7)	16.9 (11.5 to 23.5)	32.4 (22.9 to 44.8)	29.9 (20.8 to 42.1)	34.1 (22.8 to 49.4)	76.2 (57.1 to 95.8)	50.4 (31 to 73)	101.2 (73.5 to 132.4)
	Iran (Islamic Republic of)	Incidence	24.9 (22.6 to 27.4)	25 (22.6 to 27.7)	24.9 (22.5 to 27.5)	33.7 (30.9 to 36.6)	32.8 (30.1 to 35.5)	34.4 (31.4 to 37.5)	35.5 (31.3 to 39.8)	31 (25.5 to 36.5)	38.3 (34.3 to 42.8)
		Prevalence	507.8 (462.8 to 556.2)	513.4 (465.1 to 567)	503 (458.1 to 551.4)	672.4 (618.4 to 729.1)	660.8 (608.9 to 716.3)	683.2 (627.7 to 741.8)	32.4 (28.8 to 36.4)	28.7 (24.4 to 33.3)	35.8 (32.3 to 39.6)
		Deaths	8.2 (6.8 to 10.3)	7.3 (5.6 to 10.5)	9.2 (7.5 to 11)	7.1 (5.9 to 8.3)	6.6 (5.4 to 7.9)	7.5 (6.1 to 8.8)	-14 (-31.7 to -1.2)	-9.4 (-40.2 to 11.4)	-18.5 (-32.7 to -3)
		DALYs	157.2 (132.2 to 187.4)	139.1 (111.1 to 176.5)	174.9 (146.2 to 208.3)	133.1 (113.7 to 153.3)	121.6 (102.7 to 142.6)	144.5 (121.8 to 168.6)	-15.3 (-27.8 to -4.9)	-12.6 (-33.6 to 4.1)	-17.4 (-29.9 to -3.6)
		YLLs	138.1 (114.1 to 167.8)	120.9 (94.1 to 158.5)	155.1 (126.7 to 187)	110.2 (92.3 to 129.3)	101.1 (82.5 to 120.6)	119.3 (98.7 to 140.5)	-20.2 (-33.8 to -8.8)	-16.4 (-38.8 to 2.3)	-23.1 (-36.3 to -8.6)
		YLDs	19.1 (13.8 to 25.5)	18.2 (13.2 to 24.3)	19.8 (14 to 26.7)	22.8 (16.5 to 31)	20.5 (14.9 to 27.5)	25.2 (17.7 to 34.4)	19.9 (13.5 to 26.6)	12.6 (5.2 to 19.9)	27 (18.8 to 34.9)

Cause	Location	Measure	Age-standardized rate (per 100,000)						% Change (1990 to 2019)		
			1990			2019					
			Both	Female	Male	Both	Female	Male	Both	Female	Male
	Iraq	Incidence	23.8 (21.2 to 26.7)	24.5 (21.7 to 27.7)	23.2 (20.3 to 26.3)	38.7 (35.1 to 42.9)	36 (32.5 to 40.2)	41.7 (37.1 to 47)	62.5 (55.9 to 69.3)	47.3 (40.1 to 55.5)	79.6 (70.1 to 90.4)
		Prevalence	472.6 (423.1 to 526.1)	495.6 (442.9 to 554.1)	448.2 (395.8 to 506.7)	770.1 (696.1 to 853.3)	733.3 (658.8 to 819.3)	814.3 (723.3 to 914.6)	63 (56.5 to 69)	48 (40.9 to 55.1)	81.7 (71.7 to 92.8)
		Deaths	15.2 (11.3 to 21.5)	13.5 (9.3 to 21)	17.3 (12.4 to 26)	14.4 (10.6 to 19.4)	11.1 (8.1 to 15.1)	18.4 (12.4 to 28.6)	-5.6 (-26.3 to 16.6)	-17.7 (-42.9 to 13.9)	6.7 (-20.8 to 40)
		DALYs	291.4 (221.8 to 393.4)	267.4 (191.9 to 394.4)	318.7 (234.3 to 438.6)	270.7 (204.2 to 346.3)	219.4 (160.7 to 290.5)	329.9 (239.2 to 449.1)	-7.1 (-27.9 to 15.5)	-18 (-40.9 to 13.1)	3.5 (-22.4 to 34.4)
		YLLs	269.3 (199.2 to 368.6)	245.2 (168.5 to 372.2)	296.7 (213.8 to 416.7)	235.9 (173 to 313.5)	188.3 (131.6 to 260.9)	290.9 (201.3 to 412.7)	-12.4 (-33.6 to 11.8)	-23.2 (-46 to 9.6)	-2 (-29 to 30.7)
		YLDs	22.1 (15.7 to 30.5)	22.2 (15.5 to 30)	22 (15 to 31.1)	34.8 (24.1 to 48.4)	31.1 (21.5 to 43)	39.1 (26.5 to 56.6)	58 (40.6 to 76.7)	40.1 (17.6 to 64.6)	77.6 (52.5 to 106.9)
	Jordan	Incidence	23.6 (21.1 to 26.2)	26.7 (23.6 to 30)	20.6 (18 to 23.5)	40.1 (36.1 to 44.4)	38.2 (34.1 to 42.7)	41.9 (37 to 46.8)	70 (61.2 to 78.7)	43.2 (34.6 to 51.5)	102.8 (88.1 to 117.8)
		Prevalence	460.3 (413.9 to 509.5)	516.2 (462.8 to 577.5)	403.5 (356.2 to 450.1)	785 (716.8 to 860.8)	761 (688.8 to 845)	806.5 (718.5 to 903.2)	70.5 (61 to 80)	47.4 (38 to 56.4)	99.9 (85.4 to 116.3)
		Deaths	13.9 (10.7 to 17.4)	14.6 (10.7 to 19.5)	13.2 (9.9 to 16.8)	13 (10.2 to 16.1)	13.3 (9.9 to 17.3)	12.7 (9.6 to 16.6)	-6.4 (-26.4 to 16.7)	-8.7 (-37.6 to 25.9)	-3.5 (-29.7 to 30.9)
		DALYs	246.3 (192.7 to 305.9)	259.3 (191.7 to 342.6)	233.4 (180 to 295.8)	233.6 (188.4 to 280.8)	230.4 (176.1 to 292.5)	236.2 (181.5 to 305.6)	-5.2 (-24.9 to 17.5)	-11.1 (-36.7 to 19.8)	1.2 (-24 to 35.3)
		YLLs	226.1 (173.2 to 283.8)	237.7 (172.2 to 320.3)	214.5 (161.1 to 277.9)	200.9 (156.8 to 248.1)	200.6 (147.2 to 262.1)	200.8 (147.3 to 267.3)	-11.1 (-30.8 to 12.2)	-15.6 (-42.2 to 17.8)	-6.4 (-32.6 to 29.3)
		YLDs	20.2 (14.2 to 27.8)	21.7 (15.2 to 29.7)	18.9 (12.7 to 26.4)	32.7 (22.7 to 44.7)	29.9 (21.1 to 41.1)	35.4 (23.5 to 50.3)	61.6 (43.5 to 82.5)	37.9 (17.3 to 60.3)	87.3 (58.6 to 120.2)

Cause	Location	Measure	Age-standardized rate (per 100,000)						% Change (1990 to 2019)		
			1990			2019					
			Both	Female	Male	Both	Female	Male	Both	Female	Male
	Kuwait	Incidence	24.5 (21.8 to 27.7)	27.5 (24.1 to 31.1)	23.1 (20.1 to 26.7)	37.7 (33.5 to 41.9)	35.9 (31.7 to 40)	38.7 (34 to 43.9)	53.6 (40.9 to 66.6)	30.6 (20.5 to 42.4)	67.6 (51.4 to 85.8)
		Prevalence	495 (446.6 to 552.1)	553.2 (492 to 621.6)	454.3 (401.7 to 513.5)	727 (653.1 to 808.8)	716.4 (640.2 to 796.2)	732.2 (650.5 to 824.6)	46.9 (35.5 to 59.5)	29.5 (18.9 to 41.3)	61.2 (45.6 to 78)
		Deaths	9.7 (7.6 to 11.7)	10.1 (7.1 to 12.9)	9.5 (7.6 to 11.7)	6 (4.6 to 7.6)	5.5 (3.8 to 7.6)	6.3 (4.8 to 8.1)	-37.9 (-48.2 to -24.6)	-45.3 (-58.8 to -25)	-33.3 (-45.9 to -17.7)
		DALYs	182.1 (149.5 to 219.6)	196.2 (146.7 to 249)	175 (141.8 to 210.5)	117.6 (94.2 to 143.2)	110.7 (82.5 to 146.2)	121.9 (96.2 to 151.1)	-35.5 (-45 to -23)	-43.6 (-55.6 to -24.3)	-30.3 (-42.3 to -15.1)
		YLLs	162.6 (128.5 to 199.5)	175.8 (123.5 to 227.5)	155.9 (123.9 to 191.6)	89.1 (68.7 to 112.9)	85.2 (58.6 to 118.1)	91.6 (68 to 119.2)	-45.2 (-54.6 to -32.2)	-51.5 (-63.8 to -30.3)	-41.2 (-53.1 to -25.8)
		YLDs	19.5 (13.5 to 27.2)	20.4 (14 to 27.7)	19.1 (12.8 to 27.3)	28.5 (19.5 to 40)	25.5 (17.4 to 36.5)	30.3 (19.8 to 43.5)	45.9 (27.2 to 68.7)	25 (6.3 to 47.8)	58.4 (31.5 to 90.4)
	Lebanon	Incidence	21.8 (19.3 to 24.6)	23.1 (20.3 to 26.2)	20.7 (18.1 to 23.8)	39.2 (35.1 to 43.5)	36.5 (32.6 to 40.8)	42.5 (37.6 to 47.7)	79.5 (70.2 to 90.3)	58.4 (49.4 to 69.4)	105.2 (91 to 120.9)
		Prevalence	427.8 (383 to 478.1)	455.9 (405.7 to 512.9)	397.9 (351.9 to 450.1)	764.6 (690.7 to 843.7)	729.2 (655.2 to 804.4)	810.4 (723.9 to 916.3)	78.7 (70.7 to 88)	60 (51.4 to 69.6)	103.7 (90.2 to 118.3)
		Deaths	11.4 (8.8 to 14.6)	10.5 (7.7 to 14.9)	12.5 (9.5 to 16.1)	8.5 (6 to 11.4)	7.9 (5.3 to 10.2)	9.3 (5.5 to 15)	-25.5 (-48 to -1.4)	-25.1 (-49.7 to -1)	-25.9 (-56.3 to 18.1)
		DALYs	207.6 (163.8 to 265.2)	195 (147.4 to 261.2)	221.8 (170 to 289.4)	161.7 (118.8 to 209.5)	149.2 (108.4 to 188)	177.2 (116.5 to 266.1)	-22.1 (-42 to 0.4)	-23.5 (-46.1 to -0.8)	-20.1 (-46.8 to 19.3)
		YLLs	189.7 (146.8 to 246.8)	176.6 (128.1 to 244.3)	204.4 (153.7 to 268.8)	132.6 (92.6 to 178.6)	123 (82.7 to 161.6)	144.4 (84.8 to 234.2)	-30.1 (-51.4 to -5.9)	-30.3 (-53 to -6.1)	-29.3 (-57.2 to 12.6)
		YLDs	17.9 (12.3 to 25.2)	18.5 (12.7 to 25.4)	17.3 (11.5 to 25.1)	29 (19.8 to 39.9)	26.1 (17.7 to 36.6)	32.8 (21.2 to 47.1)	61.9 (44.3 to 81.6)	41.3 (21 to 64.2)	88.9 (60.7 to 122.1)

Cause	Location	Measure	Age-standardized rate (per 100,000)						% Change (1990 to 2019)		
			1990			2019					
			Both	Female	Male	Both	Female	Male	Both	Female	Male
	Libya	Incidence	22 (19.5 to 24.6)	23.2 (20.3 to 26.4)	21.1 (18.5 to 23.8)	36.7 (32.7 to 40.8)	35 (30.9 to 39.2)	38.4 (33.7 to 43.4)	66.8 (59.8 to 73.6)	50.9 (43.4 to 59.7)	82.2 (71.4 to 93.1)
		Prevalence	432.6 (388 to 481.3)	462.7 (410.1 to 519.7)	405.6 (360.2 to 454.4)	709.4 (636.7 to 787.7)	698 (621.1 to 773.7)	721.8 (639.9 to 813.8)	64 (58 to 70.1)	50.9 (43.7 to 59.1)	77.9 (68.4 to 88.8)
		Deaths	10.4 (7.4 to 14.4)	10 (6.8 to 15)	10.7 (7.4 to 15.2)	10.6 (6.9 to 14.9)	10.8 (6.5 to 15.9)	10.3 (6.7 to 16.4)	2 (-25.3 to 36)	8.2 (-26.9 to 47.3)	-4.1 (-34.6 to 41)
		DALYs	196.4 (146.3 to 261.5)	198 (141.6 to 274.6)	196 (140.1 to 275.4)	207.5 (146.4 to 280.5)	215.5 (139 to 303.3)	199.9 (137.6 to 306.3)	5.7 (-22.1 to 39.2)	8.8 (-23.9 to 46.6)	2 (-27.6 to 46.6)
		YLLs	178.2 (129.5 to 245.9)	179.1 (122.2 to 254.4)	178.2 (122.8 to 254.9)	178.5 (116 to 251.5)	188.3 (113.1 to 276.1)	169.1 (109 to 274.3)	0.2 (-28.6 to 37)	5.1 (-29.9 to 47.2)	-5.1 (-36.3 to 42.8)
		YLDs	18.2 (12.8 to 24.9)	18.9 (13.1 to 26.4)	17.7 (11.7 to 24.9)	29 (20 to 40.5)	27.2 (18.4 to 38.4)	30.9 (21 to 44.2)	59.1 (42.3 to 77.6)	43.6 (22.9 to 65.8)	73.9 (48.8 to 101.4)
	Morocco	Incidence	17.1 (15 to 19.4)	17.9 (15.4 to 20.5)	16.5 (14.2 to 18.7)	35.5 (31.4 to 39.6)	32.8 (28.9 to 37.1)	38.4 (33.5 to 43.5)	107.1 (98.4 to 117)	83.7 (73.5 to 95.1)	133 (120.2 to 147.7)
		Prevalence	331.4 (295.9 to 370.6)	351.8 (308.7 to 397.3)	309.4 (273 to 348.6)	684.4 (613.2 to 756.2)	652.8 (580.4 to 733.8)	718.7 (636.4 to 803.2)	106.5 (97.7 to 116.3)	85.5 (75.9 to 96)	132.3 (118.6 to 148)
		Deaths	9.9 (7.4 to 14.7)	8.5 (6 to 13.8)	11.6 (8.2 to 17.8)	12.3 (9.2 to 16.5)	11.5 (8.3 to 16.6)	13.1 (8.9 to 19.1)	24.4 (-6.7 to 56.8)	34.5 (-11.3 to 84.9)	13 (-17.4 to 51.7)
		DALYs	183.9 (143.9 to 252)	167.7 (123.4 to 247.2)	203.1 (148.6 to 290.1)	227.8 (174.8 to 294)	220.2 (161.8 to 296.3)	236.4 (168.8 to 319.4)	23.8 (-6.3 to 55.3)	31.3 (-10.4 to 79)	16.4 (-13.6 to 53)
		YLLs	169.1 (129.4 to 238.4)	152.3 (108.3 to 230.5)	188.9 (133.2 to 277.3)	197.8 (144.5 to 261.6)	193.1 (139.1 to 267)	203.4 (138.6 to 287)	16.9 (-13.9 to 50.2)	26.8 (-17.3 to 79.3)	7.7 (-23 to 45.9)
		YLDs	14.8 (10.2 to 20.1)	15.4 (10.5 to 21.1)	14.2 (9.5 to 20)	30 (20.5 to 41.2)	27.1 (18.4 to 38.2)	33 (21.9 to 46.9)	102.6 (79.6 to 125.1)	76.4 (51.1 to 103.1)	132.8 (99.3 to 167.9)

Cause	Location	Measure	Age-standardized rate (per 100,000)						% Change (1990 to 2019)		
			1990			2019					
			Both	Female	Male	Both	Female	Male	Both	Female	Male
	Oman	Incidence	18.7 (16.6 to 21.1)	19.4 (16.9 to 22.2)	18.5 (16.1 to 20.9)	37.3 (33.4 to 41.3)	34.4 (30.6 to 38.5)	40 (35.2 to 44.9)	98.9 (88.2 to 109.2)	77.6 (66.6 to 90)	116.4 (101.5 to 131.4)
		Prevalence	368.3 (331.9 to 411.7)	384.3 (341.5 to 432.7)	354.9 (315.4 to 399.7)	707.8 (643.9 to 779)	676 (603.9 to 749.3)	746.7 (666.9 to 830.2)	92.2 (82.4 to 102.4)	75.9 (65.5 to 88.1)	110.4 (96 to 125.4)
		Deaths	5.9 (4.3 to 8)	5.3 (3.8 to 7.4)	7.4 (5.2 to 10.6)	7.2 (5.6 to 8.8)	6.4 (4.7 to 8.3)	8.4 (6.4 to 10.8)	21 (-15.9 to 66.5)	21.9 (-18.7 to 69.1)	14.2 (-30.8 to 69.6)
		DALYs	115.3 (87.4 to 152.9)	108.5 (80.4 to 147.4)	128.7 (93 to 179)	135.6 (111.6 to 162.4)	128.4 (99.7 to 159.3)	147.3 (117.9 to 181.5)	17.6 (-11.8 to 52.1)	18.4 (-14.6 to 57.2)	14.5 (-21.8 to 60.3)
		YLLs	98 (71 to 135)	90.4 (63.5 to 129.7)	111.7 (77.6 to 162.8)	103 (80.3 to 128.7)	98.1 (71.6 to 128.6)	112.1 (85.6 to 144)	5.1 (-25.1 to 45.4)	8.4 (-26.7 to 52.5)	0.3 (-37.3 to 52.2)
		YLDs	17.3 (12.3 to 23.7)	18.1 (12.6 to 24.7)	16.9 (11.8 to 23.8)	32.7 (23.4 to 44.4)	30.3 (21.6 to 41.7)	35.2 (23.6 to 49.8)	88.6 (68.4 to 111.5)	68 (47 to 90.3)	107.8 (79.1 to 141)
	Palestine	Incidence	24.1 (21.6 to 26.9)	24 (21.1 to 27.6)	24.2 (21.3 to 27.2)	38.3 (34.4 to 42.6)	35.1 (31.1 to 39.5)	42.1 (37.3 to 47.4)	59.1 (52.2 to 66.9)	46 (37.5 to 55.5)	74.4 (62.6 to 86.6)
		Prevalence	477.3 (430.2 to 531.2)	486.3 (431.8 to 545.1)	467 (415.7 to 522.3)	741.6 (671 to 821.6)	695.9 (623.7 to 775.9)	803.7 (714.6 to 901.2)	55.4 (49.2 to 61.9)	43.1 (34.8 to 52)	72.1 (59.6 to 84.7)
		Deaths	16.4 (12.7 to 20.8)	14 (10.3 to 18.3)	19.9 (15.2 to 25.6)	11.7 (9.2 to 14.5)	10 (7.6 to 12.7)	14.9 (11.4 to 18.5)	-28.4 (-45.5 to -8.3)	-28.6 (-46.9 to -2.9)	-24.9 (-44.9 to -2.4)
		DALYs	306.1 (234.3 to 390.6)	270.5 (196.7 to 351.3)	352.8 (266.6 to 462.8)	225 (180.8 to 274.3)	195 (150.4 to 243.5)	267.1 (213.9 to 324.1)	-26.5 (-42.8 to -5.5)	-27.9 (-46.5 to -2.3)	-24.3 (-42.8 to -1.6)
		YLLs	285.3 (213.2 to 370.3)	250 (176 to 333.3)	331.4 (246.9 to 441.4)	194 (150.7 to 242.8)	167.7 (125.1 to 215.7)	231.5 (178.8 to 290.2)	-32 (-48.8 to -10.6)	-32.9 (-51.6 to -6)	-30.1 (-48.3 to -7.5)
		YLDs	20.8 (14.3 to 28.6)	20.5 (14.4 to 28.5)	21.3 (13.9 to 29.7)	31 (21.1 to 42.5)	27.3 (18.9 to 37.7)	35.6 (23.7 to 51)	49.1 (31.7 to 68.7)	33.5 (13.6 to 57.2)	66.7 (41.7 to 95.4)

Cause	Location	Measure	Age-standardized rate (per 100,000)						% Change (1990 to 2019)		
			1990			2019					
			Both	Female	Male	Both	Female	Male	Both	Female	Male
	Qatar	Incidence	26.8 (23.8 to 30.2)	30 (26.3 to 34.3)	26.3 (23.1 to 29.9)	44.2 (39.8 to 49.2)	39.7 (35.7 to 44.7)	46 (40.9 to 51.6)	64.7 (57 to 72.8)	32.2 (24.7 to 40.3)	75.1 (65.6 to 85.4)
		Prevalence	511.1 (456.3 to 573.3)	573.4 (507.4 to 650.3)	470.7 (417.5 to 531.2)	852.2 (766.6 to 946.1)	803.9 (719.9 to 893.5)	867.6 (777.2 to 974.2)	66.7 (58.1 to 75)	40.2 (31.8 to 48.5)	84.3 (73.4 to 95.3)
		Deaths	17.1 (12.3 to 30.1)	18.2 (12.1 to 36.6)	16.8 (11.6 to 26)	17.5 (13.2 to 22.4)	24.7 (18.2 to 32.2)	15.2 (11.4 to 20.1)	2.6 (-35.9 to 41)	35.4 (-29.5 to 98.4)	-9.9 (-39.5 to 32)
		DALYs	277.2 (205.4 to 446.4)	309 (214.6 to 573.3)	263.6 (189.1 to 385.4)	258.1 (203.8 to 324.8)	353.7 (265.3 to 458.7)	225.9 (174.6 to 288.2)	-6.9 (-38.2 to 25)	14.5 (-34.5 to 62.9)	-14.3 (-39.7 to 20.8)
		YLLs	254.9 (184.5 to 423.7)	285.2 (191.7 to 548.5)	241.7 (165.5 to 360.9)	221.1 (166.7 to 288.8)	321.9 (234.3 to 426.7)	187 (137 to 249.2)	-13.3 (-43.9 to 20.7)	12.9 (-37.7 to 66.9)	-22.6 (-48.4 to 14)
		YLDs	22.3 (15.8 to 31.1)	23.8 (16.8 to 33.1)	21.9 (14.8 to 31.1)	37.1 (25.5 to 52.2)	31.8 (22.2 to 43.9)	38.9 (26 to 56)	66.2 (45.9 to 88)	33.8 (15.4 to 55.7)	77.4 (53.3 to 107.3)
	Saudi Arabia	Incidence	25 (22.2 to 28)	26.4 (23.1 to 29.9)	24.2 (21.3 to 27.4)	45.7 (41.4 to 50.2)	41 (36.8 to 45.8)	48.7 (43.7 to 53.9)	82.8 (72.7 to 94)	55.6 (45.2 to 66.8)	101.6 (88.2 to 115.5)
		Prevalence	489.7 (439.4 to 539.9)	528.8 (465.6 to 591.5)	460.2 (410.9 to 515.6)	926.3 (841.3 to 1008.6)	879 (792.2 to 975.7)	961.8 (864 to 1063.4)	89.1 (79.6 to 99.4)	66.2 (54.8 to 79.3)	109 (96.1 to 123.5)
		Deaths	16.9 (12.7 to 21.9)	13.9 (10.4 to 18.6)	19.8 (14.2 to 26.9)	19.3 (14.7 to 24.1)	17.6 (12.3 to 23.5)	20.7 (15.5 to 26.3)	14.3 (-18.9 to 56.4)	27 (-15.3 to 81.2)	4.3 (-30.4 to 54.6)
		DALYs	324.2 (244 to 427.4)	282.3 (210 to 379.6)	361.2 (258.9 to 492.7)	360.9 (283 to 452.8)	340.2 (245 to 456.3)	376.6 (291.5 to 469.4)	11.3 (-20.5 to 51.7)	20.5 (-17.7 to 68.2)	4.3 (-30.2 to 54.5)
		YLLs	302.5 (221.6 to 406.1)	259.9 (187.4 to 357.4)	339.9 (239.9 to 470.4)	322 (242.2 to 411.3)	305.2 (212.5 to 420)	335.1 (252.5 to 430.9)	6.4 (-25.2 to 49.3)	17.4 (-22.5 to 69.8)	-1.4 (-35 to 51.7)
		YLDs	21.7 (15.1 to 29.7)	22.4 (15.7 to 30.8)	21.3 (14.5 to 30.2)	38.9 (26.7 to 54.4)	35.1 (23.7 to 49.2)	41.5 (27.9 to 59.3)	79.3 (58.3 to 102)	56.5 (33.5 to 83)	95 (66.1 to 126.9)

Cause	Location	Measure	Age-standardized rate (per 100,000)						% Change (1990 to 2019)		
			1990			2019					
			Both	Female	Male	Both	Female	Male	Both	Female	Male
	Sudan	Incidence	17.2 (15.1 to 19.3)	17.9 (15.6 to 20.5)	16.5 (14.4 to 18.8)	31.5 (27.9 to 35.4)	29.5 (25.8 to 33.4)	33.2 (29 to 37.9)	83.8 (76 to 91.6)	64.5 (55.9 to 72.8)	101.2 (89.9 to 113.5)
		Prevalence	328.2 (292.9 to 370)	345.7 (303.9 to 394.9)	311.1 (274.7 to 350.2)	587.9 (522.2 to 653.8)	562.8 (496.1 to 632.4)	608.4 (537 to 686)	79.1 (71.4 to 87)	62.8 (54.4 to 71.3)	95.6 (83.7 to 108)
		Deaths	10.5 (7.6 to 15.9)	8.9 (6.1 to 14.7)	12.1 (8.6 to 18.9)	10.6 (7.1 to 17.3)	9.4 (6 to 14)	11.7 (7.3 to 21.3)	1.3 (-32.1 to 51.1)	5.3 (-39.2 to 55.1)	-3.4 (-36 to 61.3)
		DALYs	204.8 (152.7 to 296.6)	184.6 (128.9 to 283.3)	224.6 (164.8 to 337.4)	204.5 (144.3 to 315)	187.9 (127.6 to 273.2)	218.8 (143 to 369.5)	-0.1 (-31.5 to 44.3)	1.8 (-38.8 to 47.3)	-2.6 (-35.5 to 54.8)
		YLLs	190.7 (139.3 to 281.7)	170.1 (114.7 to 269.2)	211.1 (151.5 to 320.9)	179.8 (118.9 to 285.8)	165.3 (104.1 to 250.8)	192.4 (118.1 to 344.6)	-5.7 (-38.4 to 41.3)	-2.8 (-44.6 to 45.5)	-8.8 (-41.7 to 51.6)
		YLDs	14.1 (9.8 to 19.4)	14.5 (10.2 to 20.2)	13.6 (9.4 to 19.1)	24.7 (16.9 to 34.8)	22.6 (15.5 to 30.9)	26.4 (17.8 to 38.2)	75.7 (56.6 to 95.9)	55.2 (35.6 to 77.7)	94.5 (66.8 to 126.8)
	Syrian Arab Republic	Incidence	22.1 (19.7 to 24.8)	24.2 (21.2 to 27.3)	20.2 (17.7 to 23.2)	36.3 (32.9 to 40.1)	35 (31.1 to 38.7)	37.2 (32.8 to 42.1)	64.2 (54.5 to 74.1)	44.9 (36.4 to 54.5)	83.7 (67.9 to 100.1)
		Prevalence	438.1 (392.5 to 487.6)	476.6 (422.1 to 536)	403.5 (357.4 to 457.5)	697 (635.5 to 769.7)	681.5 (607.8 to 760)	705.7 (630.8 to 787.2)	59.1 (50 to 68.4)	43 (34.2 to 51.6)	74.9 (60.8 to 90.7)
		Deaths	12.8 (9.8 to 17.2)	12.1 (9.2 to 16.3)	13.5 (9.9 to 19)	10.3 (7.6 to 13.3)	11.1 (8.2 to 14.5)	10 (7.3 to 13.2)	-20 (-43.8 to 7.6)	-8 (-38 to 25.3)	-26.3 (-52.7 to 7.8)
		DALYs	244.8 (190 to 318.5)	227.9 (174.3 to 301.2)	260.1 (195.6 to 352.5)	190.2 (145 to 246.9)	191.5 (143.3 to 254.2)	192.8 (145.6 to 252.8)	-22.3 (-43.1 to 4.2)	-16 (-39.7 to 13.7)	-25.9 (-49.8 to 7.4)
		YLLs	225 (171.2 to 299.2)	207.2 (153.1 to 280.3)	241.1 (174.2 to 332.4)	162.2 (118.4 to 218.1)	165.7 (119.5 to 228.8)	163.1 (117.4 to 222.9)	-27.9 (-48.9 to 0.2)	-20 (-44.7 to 12.6)	-32.4 (-56.3 to 2.6)
		YLDs	19.8 (13.8 to 27.8)	20.7 (14.3 to 29)	19 (12.7 to 27.2)	28 (19.3 to 38.4)	25.8 (17.4 to 35.6)	29.7 (19.8 to 41.9)	41 (24 to 58.8)	24.3 (6.8 to 45.5)	56.6 (30.8 to 86.4)

Cause	Location	Measure	Age-standardized rate (per 100,000)						% Change (1990 to 2019)		
			1990			2019					
			Both	Female	Male	Both	Female	Male	Both	Female	Male
	Tunisia	Incidence	21.2 (18.8 to 23.8)	22.1 (19.2 to 25)	20.5 (18 to 23.4)	37.3 (33.3 to 41.2)	34.9 (30.8 to 38.9)	39.9 (35.6 to 44.8)	75.6 (66.9 to 83.9)	57.8 (48.2 to 67.6)	94.9 (82.7 to 108.7)
		Prevalence	405.9 (366.8 to 450.4)	427.7 (380.4 to 478.1)	384.8 (343.6 to 435.4)	705.4 (638.3 to 776.8)	676.1 (606.4 to 750.1)	740.2 (664.3 to 827.2)	73.8 (66.1 to 81.4)	58.1 (49.6 to 67.7)	92.4 (80.8 to 105.2)
		Deaths	8.5 (6.5 to 10.8)	7.5 (5.7 to 10.8)	9.7 (7.3 to 12.4)	8.4 (5.9 to 11.6)	7.6 (5 to 10.6)	9.4 (6.3 to 13.6)	-1.3 (-32.3 to 33.7)	0.5 (-31.8 to 42)	-2.8 (-36.8 to 39.1)
		DALYs	154.3 (122.5 to 193.4)	141.6 (110.7 to 195.9)	167.9 (128.2 to 213.2)	157.4 (116.5 to 209.1)	142.6 (102.6 to 193.3)	174.4 (125.6 to 246.1)	2.1 (-25.7 to 35.5)	0.7 (-28.1 to 38.6)	3.9 (-27 to 43.3)
		YLLs	136.9 (105.9 to 176.1)	124.7 (93.8 to 178.7)	150.2 (110.3 to 194.9)	129.5 (90.1 to 182.9)	118.2 (77.3 to 170.1)	142.5 (94.7 to 210.2)	-5.4 (-35.6 to 30.2)	-5.2 (-37.3 to 37.4)	-5.1 (-37.9 to 38.1)
		YLDs	17.3 (12.2 to 23.6)	16.9 (11.8 to 23.4)	17.7 (12.1 to 24.4)	27.9 (19.2 to 38.5)	24.4 (16.5 to 33.1)	31.9 (21.4 to 44.9)	61.2 (44.3 to 81.5)	43.8 (24 to 66.2)	80.5 (53.8 to 109.8)
	Turkey	Incidence	19 (17.1 to 21)	18.2 (16.1 to 20.6)	19.8 (17.7 to 22)	35.9 (32.2 to 39.9)	32.8 (29.2 to 37)	39.5 (35 to 44.8)	89.2 (76.2 to 103.4)	79.8 (66.5 to 95.5)	99.2 (83.4 to 118.4)
		Prevalence	358 (327.1 to 394.1)	349.8 (313.5 to 391.1)	368.1 (332.8 to 406.7)	669.5 (607.5 to 737.3)	619.7 (553.3 to 688.1)	731.1 (654.2 to 824.6)	87 (75 to 100)	77.2 (64.2 to 92.5)	98.7 (83 to 117.7)
		Deaths	11.7 (8.5 to 17.9)	9.3 (6.5 to 15)	14.8 (10.2 to 23.6)	8.2 (6.1 to 10.6)	7.4 (5.3 to 9.8)	9.4 (6.9 to 12.1)	-29.6 (-56.2 to -2.1)	-20.7 (-52.7 to 13.1)	-36.8 (-61.5 to -4.4)
		DALYs	217.6 (163.8 to 309.1)	174.9 (127.3 to 262)	267.5 (194.9 to 401.4)	154.2 (119.4 to 192.4)	135 (104.1 to 172.4)	176.5 (136.2 to 222.9)	-29.2 (-52 to -5.6)	-22.8 (-48.8 to 6.2)	-34 (-57.2 to -4.8)
		YLLs	200.7 (145.8 to 290.6)	159 (111.6 to 247.2)	249.4 (178.3 to 383.2)	126.9 (94.9 to 164.5)	111.5 (82 to 148.5)	144.7 (106.2 to 190.5)	-36.8 (-59 to -11.8)	-29.9 (-56 to 1.4)	-42 (-63.6 to -12.9)
		YLDs	16.9 (12.1 to 23.4)	15.9 (11.4 to 22.1)	18.1 (12.5 to 25.3)	27.3 (19 to 38.1)	23.6 (16.4 to 32.7)	31.7 (20.9 to 45.5)	61.5 (43.4 to 83.4)	48.7 (29.5 to 73.8)	74.9 (48.1 to 103.7)

Cause	Location	Measure	Age-standardized rate (per 100,000)						% Change (1990 to 2019)		
			1990			2019					
			Both	Female	Male	Both	Female	Male	Both	Female	Male
	United Arab Emirates	Incidence	28.5 (25.4 to 31.7)	29.6 (26.1 to 33.4)	28 (24.7 to 31.5)	43.8 (39.6 to 48.4)	40.6 (36.5 to 45.1)	45.6 (40.9 to 50.8)	53.9 (47.6 to 60.4)	37.2 (30.9 to 43.8)	63.1 (54.9 to 71.1)
		Prevalence	540 (485.4 to 600.9)	573.4 (506.4 to 650.3)	516.8 (458.2 to 580.5)	856.3 (772.9 to 945.8)	815.3 (737 to 899.2)	874.4 (782.3 to 977.6)	58.6 (51.4 to 65.1)	42.2 (34.8 to 48.9)	69.2 (59.5 to 78.8)
		Deaths	21.1 (12.1 to 27.4)	20.3 (11.3 to 29.9)	21.8 (12.3 to 29.6)	17 (9.7 to 26.6)	16.7 (8.2 to 24)	17.1 (9.8 to 30.1)	-19.6 (-44.9 to 15.5)	-17.5 (-52.8 to 22.4)	-21.7 (-46.8 to 23.6)
		DALYs	385.5 (237.8 to 495.7)	368 (224.3 to 527.5)	397.6 (242.5 to 541.3)	325.6 (206.5 to 490.1)	312.9 (176.7 to 438.8)	330.5 (211.3 to 547.5)	-15.6 (-40.5 to 18.7)	-15 (-49.1 to 24.3)	-16.9 (-43.2 to 27)
		YLLs	361.1 (214.9 to 470)	343.9 (196.8 to 504.1)	372.8 (216.6 to 518)	287.9 (170.9 to 453.2)	280.7 (144.9 to 405.8)	290.4 (171.9 to 510.1)	-20.3 (-46.1 to 16)	-18.4 (-53.7 to 23.3)	-22.1 (-49.5 to 24.2)
		YLDs	24.4 (17 to 33.5)	24 (16.6 to 33.4)	24.8 (17 to 35.4)	37.7 (25.8 to 52.9)	32.3 (22.3 to 45.4)	40.1 (26.8 to 58.1)	54.6 (35.6 to 75.3)	34.1 (16.2 to 55.9)	61.6 (39.1 to 89.2)
	Yemen	Incidence	16.7 (14.8 to 18.9)	17.1 (15 to 19.6)	16.5 (14.4 to 18.9)	28.9 (25.5 to 32.5)	27.4 (23.7 to 32.2)	30.5 (26.5 to 34.9)	72.8 (63.1 to 85.9)	60.5 (46.4 to 83.5)	85 (74.3 to 95.7)
		Prevalence	323.3 (287.6 to 363.9)	332.1 (292.6 to 377.6)	311 (274.6 to 354.1)	536.4 (477.7 to 599.3)	519.8 (451.4 to 610.1)	555.1 (485.2 to 629.6)	65.9 (56.6 to 79.2)	56.5 (43.1 to 80.3)	78.5 (68.1 to 88.8)
		Deaths	8.7 (6.1 to 12.4)	7.1 (4.8 to 11.5)	11.6 (8 to 17.3)	8.4 (6.1 to 11.7)	6.9 (4.9 to 9.9)	10.1 (7.1 to 14.5)	-3.4 (-27.1 to 27.1)	-3.1 (-32.6 to 35.8)	-12.6 (-34.1 to 17.3)
		DALYs	176.1 (125.1 to 241)	149.2 (101.9 to 228.7)	215.6 (151.9 to 307.8)	168.6 (128 to 226.5)	145.8 (106.9 to 205.8)	193.4 (142.8 to 263.8)	-4.2 (-27.4 to 26.4)	-2.3 (-30.7 to 34)	-10.3 (-32.7 to 20.4)
		YLLs	161.7 (110.9 to 228.4)	134.5 (87.7 to 213.5)	201.5 (138.1 to 291.4)	145.6 (105.8 to 203.5)	123.8 (86.5 to 182)	169.2 (116.5 to 240.5)	-10 (-33.4 to 22.8)	-8 (-38.4 to 32.8)	-16 (-38.5 to 15.9)
		YLDs	14.4 (10 to 19.6)	14.7 (10.1 to 20.3)	14.1 (9.4 to 19.8)	23 (15.7 to 32)	22 (15 to 30.9)	24.1 (16.1 to 34.1)	60.2 (42.3 to 79.6)	49.9 (26.1 to 80.3)	71 (46.8 to 99.7)

Cause	Location	Measure	Age-standardized rate (per 100,000)						% Change (1990 to 2019)		
			1990			2019					
			Both	Female	Male	Both	Female	Male	Both	Female	Male
Chronic kidney disease due to glomerulonephritis	North Africa and Middle East	Incidence	8.2 (7.4 to 9.1)	6.3 (5.5 to 7.2)	9.9 (9 to 11)	13.4 (12 to 14.9)	9.6 (8.4 to 11)	17 (15.3 to 18.8)	64.2 (58.3 to 69.6)	52.4 (46.1 to 58.7)	70.5 (64.3 to 76.6)
		Prevalence	192.5 (175.4 to 210.5)	174.5 (156.6 to 193.6)	209.9 (191.8 to 229.7)	280.6 (255.5 to 307.4)	241.3 (216.9 to 267.6)	317.6 (289.3 to 350.3)	45.8 (41.6 to 50.2)	38.3 (33.6 to 43)	51.3 (46.5 to 56.4)
		Deaths	2 (1.5 to 2.7)	1.7 (1.3 to 2.3)	2.4 (1.7 to 3.3)	1.7 (1.2 to 2.3)	1.5 (1 to 2)	1.9 (1.4 to 2.7)	-16.9 (-31.1 to -1.3)	-15.4 (-33.7 to 0.6)	-18.9 (-35.4 to 2.5)
		DALYs	71.2 (57.6 to 87.3)	67.8 (54 to 84.4)	74.7 (60.1 to 93.5)	64 (50.2 to 79.8)	60 (46.3 to 74.8)	67.9 (53.5 to 86.6)	-10.1 (-21.7 to 2.9)	-11.5 (-25.6 to 2.6)	-9.2 (-21.5 to 7.3)
		YLLs	57.8 (44.7 to 73.1)	53.6 (40.5 to 69.5)	62.2 (48.1 to 80.7)	43.3 (32.4 to 56.6)	39.1 (28.1 to 52.6)	47.5 (34.7 to 64.9)	-25 (-37 to -10.7)	-27 (-42 to -11.5)	-23.7 (-36.3 to -5.8)
		YLDs	13.4 (9.1 to 18.3)	14.2 (9.6 to 19.5)	12.6 (8.6 to 17.2)	20.6 (14 to 28.2)	20.9 (14.3 to 28.7)	20.4 (13.8 to 28)	54.1 (46.5 to 63)	46.8 (37.2 to 57.6)	62.3 (52.1 to 73.6)
	Afghanistan	Incidence	7.9 (5.8 to 10.6)	6.8 (4.3 to 10.1)	9 (6.7 to 11.9)	11.8 (8.7 to 15.8)	9.4 (6.1 to 14.1)	14.3 (10.6 to 18.7)	49.6 (43.7 to 56)	38.2 (32.2 to 45.2)	58 (49.5 to 66.1)
		Prevalence	177 (144.6 to 219.2)	175.8 (135.6 to 225.1)	176.5 (142 to 218.9)	248 (201.2 to 306.9)	236.5 (182.2 to 308.3)	261.5 (210.5 to 328.1)	40.2 (33.9 to 46.7)	34.6 (27.6 to 42.5)	48.1 (38.8 to 57.3)
		Deaths	3.6 (2.5 to 5.5)	3.2 (2.2 to 5.2)	3.9 (2.5 to 6.2)	2.9 (2 to 4.5)	2.7 (1.7 to 4.3)	3.2 (2 to 5.2)	-19.3 (-40.2 to 4.6)	-18.1 (-46.8 to 12.4)	-17.6 (-38.1 to 7.7)
		DALYs	127.6 (94.3 to 179.5)	133 (91.1 to 205.5)	118.9 (86.3 to 167.1)	103.6 (76.7 to 145.5)	107 (75 to 161.5)	101.5 (71.8 to 144.4)	-18.8 (-37.8 to 3.2)	-19.6 (-43.5 to 6.9)	-14.6 (-34.6 to 12.9)
		YLLs	113.9 (80.7 to 165.8)	116.8 (75.5 to 189)	108.1 (76.2 to 156.5)	83.3 (57.5 to 126.1)	83.5 (53.3 to 137.7)	84.1 (56.1 to 128.4)	-26.8 (-46.1 to -3.3)	-28.5 (-52.9 to -0.2)	-22.2 (-42.9 to 7.9)
		YLDs	13.8 (8.9 to 20.4)	16.2 (10.2 to 24.4)	10.9 (6.8 to 16.2)	20.3 (12.9 to 29.9)	23.5 (14.3 to 35.8)	17.4 (10.8 to 25.4)	47.4 (25.9 to 70.8)	45 (17.7 to 77.2)	60.6 (29.9 to 100.4)

Cause	Location	Measure	Age-standardized rate (per 100,000)						% Change (1990 to 2019)		
			1990			2019					
			Both	Female	Male	Both	Female	Male	Both	Female	Male
	Algeria	Incidence	8.1 (5.9 to 10.7)	6.6 (4.4 to 9.6)	9.6 (7.1 to 12.6)	13.3 (9.9 to 17.2)	9.9 (6.7 to 14.3)	16.5 (12.5 to 21.2)	63.6 (56.4 to 72.3)	50.8 (42.9 to 58.8)	71.3 (61.1 to 82.4)
		Prevalence	192.2 (155.7 to 237.9)	179.1 (138.4 to 230.5)	205.2 (164.4 to 256)	283.8 (230.3 to 352.4)	250.4 (194.1 to 321.6)	316.1 (250.5 to 394.6)	47.7 (40.3 to 55.2)	39.8 (31.4 to 48.3)	54.1 (44.3 to 65.2)
		Deaths	2.2 (1.5 to 3.3)	2.1 (1.3 to 3.7)	2.4 (1.6 to 3.5)	1.7 (1.1 to 2.5)	1.7 (1.1 to 2.6)	1.7 (1.1 to 2.5)	-25.6 (-43.1 to -1.4)	-18.1 (-41 to 10.1)	-30.3 (-48.8 to -0.8)
		DALYs	72.8 (54.3 to 101.8)	74.5 (51.5 to 119.2)	71 (52.9 to 94)	60.6 (46 to 78.6)	63 (46.3 to 86.8)	58.4 (43 to 78.6)	-16.7 (-34.3 to 5.2)	-15.4 (-38.7 to 9.6)	-17.8 (-38 to 10.5)
		YLLs	59.2 (41.6 to 88.5)	59.6 (37.5 to 104)	58.8 (41.2 to 82.5)	39.3 (27.1 to 55.5)	40.7 (27.1 to 62.3)	38 (25.9 to 56.5)	-33.7 (-49.2 to -10.8)	-31.7 (-51.2 to -6.1)	-35.4 (-53.7 to -5.8)
		YLDs	13.5 (8.4 to 20)	14.9 (9 to 22.6)	12.3 (7.4 to 18.1)	21.3 (13.4 to 31.3)	22.3 (13.5 to 33.8)	20.4 (12.3 to 30.7)	57.6 (37.3 to 83.6)	49.8 (21.7 to 84.1)	66.5 (34.8 to 106.4)
	Bahrain	Incidence	8.2 (6.2 to 10.7)	6.3 (4.3 to 9)	9.7 (7.4 to 12.4)	14.8 (11.4 to 19)	9.6 (6.5 to 13.8)	18 (13.7 to 23.1)	80.4 (70 to 92.3)	52.9 (44.1 to 62.2)	86.4 (74.5 to 99.5)
		Prevalence	189.5 (153.6 to 233.7)	173 (133.5 to 222.1)	203.7 (163.5 to 256.3)	295.9 (240.3 to 366.3)	242.8 (188.5 to 314.8)	333.1 (267.9 to 418.5)	56.1 (46.9 to 66)	40.3 (30.7 to 50.7)	63.6 (51.6 to 75.8)
		Deaths	1.9 (1.3 to 2.8)	1.7 (1.1 to 2.5)	2.2 (1.5 to 3.5)	1.7 (1.1 to 2.4)	1.4 (0.9 to 2)	1.9 (1.3 to 2.9)	-14.1 (-32.5 to 9)	-17.4 (-39.8 to 9.3)	-14.5 (-34.7 to 10.5)
		DALYs	55 (42.1 to 72.1)	52.3 (38.7 to 70.3)	58.5 (43.8 to 81)	54.5 (41.7 to 70.5)	48.4 (35.7 to 64.9)	59.7 (44.3 to 80)	-0.8 (-18.6 to 19.3)	-7.6 (-28.1 to 15.2)	2 (-19.5 to 27.3)
		YLLs	41.8 (30.1 to 58)	37.9 (26.2 to 54.4)	46 (32.1 to 67.4)	32.4 (23.2 to 45.1)	27 (18.8 to 38.3)	37 (25.6 to 53.6)	-22.4 (-39.8 to -1.4)	-28.9 (-48.2 to -4.9)	-19.5 (-38.2 to 4.7)
		YLDs	13.2 (8.4 to 19.5)	14.4 (8.9 to 21.5)	12.6 (7.8 to 18.5)	22.1 (14 to 31.6)	21.4 (13.3 to 32.4)	22.7 (14 to 33.2)	67.3 (43 to 94.9)	48.5 (18.7 to 81.4)	80.4 (44.1 to 123.1)

Cause	Location	Measure	Age-standardized rate (per 100,000)						% Change (1990 to 2019)		
			1990			2019					
			Both	Female	Male	Both	Female	Male	Both	Female	Male
	Egypt	Incidence	7.8 (5.8 to 10.3)	6.6 (4.5 to 9.6)	9 (6.6 to 11.5)	13.8 (10.3 to 17.9)	10.2 (7 to 14.7)	17.1 (12.7 to 21.6)	77 (68.5 to 86.8)	54.3 (47 to 61.9)	90.3 (78.7 to 104.1)
		Prevalence	181.5 (146.3 to 223.9)	175.6 (134.5 to 224.4)	187.2 (147.1 to 231.5)	285.4 (231.2 to 351.4)	251 (192.9 to 321.6)	315.4 (249.9 to 387.3)	57.3 (48.9 to 65.6)	42.9 (35 to 51.9)	68.5 (56.5 to 81.6)
		Deaths	2.3 (1.5 to 3.2)	2.2 (1.1 to 3.4)	2.3 (1.5 to 3.4)	2.4 (1.3 to 3.8)	2.5 (1 to 4.2)	2.3 (1.4 to 3.9)	5.7 (-21.5 to 39.4)	12.3 (-24.5 to 48.9)	2.4 (-31.1 to 41.7)
		DALYs	74.3 (52.3 to 98.1)	79.7 (48 to 109.4)	69.4 (51 to 94.5)	78 (51.4 to 110.4)	79 (43.8 to 116.6)	77.6 (52.2 to 120.2)	4.9 (-15.5 to 30.4)	-0.8 (-23 to 25.7)	11.8 (-13.8 to 41.6)
		YLLs	61.3 (39.9 to 84.1)	65 (34 to 94.2)	57.9 (40.6 to 82.7)	56.1 (31.1 to 86.9)	56.6 (23.1 to 93.3)	56.2 (32.7 to 94.7)	-8.5 (-33 to 20.6)	-12.9 (-41.2 to 18.1)	-2.9 (-32.8 to 33)
		YLDs	13 (8.5 to 18.4)	14.7 (9.2 to 21.3)	11.5 (7.3 to 16.3)	21.9 (14.1 to 31.3)	22.3 (13.9 to 32.9)	21.3 (13.2 to 31.3)	67.9 (46.6 to 91.7)	52.4 (24.7 to 84.5)	86.1 (53 to 119.5)
	Iran (Islamic Republic of)	Incidence	10.5 (9.4 to 11.8)	7.7 (6.8 to 8.8)	13.2 (11.8 to 14.7)	13.8 (12.4 to 15.4)	9.9 (8.8 to 11.3)	17.6 (15.8 to 19.5)	30.9 (27.7 to 34.4)	28.4 (24.1 to 32.5)	33.4 (30.1 to 37.1)
		Prevalence	241.2 (219.2 to 267)	206.2 (184.6 to 228.6)	274.3 (249 to 304)	297.4 (269.7 to 327.7)	250 (223.6 to 277.3)	344 (313.6 to 380)	23.3 (21 to 26)	21.2 (18 to 24.5)	25.4 (22.6 to 28.4)
		Deaths	1.5 (1.1 to 2)	1.2 (0.9 to 1.6)	1.8 (1.4 to 2.5)	1.2 (0.9 to 1.5)	0.9 (0.7 to 1.3)	1.4 (1 to 1.8)	-24.2 (-33.7 to -15.9)	-23.5 (-42.3 to -11.5)	-25.3 (-34 to -12.6)
		DALYs	58.7 (47.4 to 71.5)	52 (41.3 to 64.3)	65 (51.7 to 80.1)	45.9 (37.4 to 55.1)	40.1 (32.3 to 48.8)	51.7 (41.4 to 62.9)	-21.7 (-30.9 to -12.6)	-22.9 (-33.4 to -12.4)	-20.4 (-29.8 to -9)
		YLLs	43.8 (33.8 to 55.4)	36.9 (27.3 to 47.8)	50.4 (37.9 to 65.3)	28.1 (22.1 to 35.5)	22.6 (17.4 to 29.1)	33.6 (26 to 42.7)	-35.8 (-44.2 to -26.9)	-38.8 (-48.7 to -27.8)	-33.3 (-42.2 to -20.8)
		YLDs	14.9 (10.1 to 19.9)	15.1 (10.3 to 20.3)	14.6 (9.8 to 19.9)	17.8 (11.9 to 24.2)	17.5 (11.7 to 24)	18.1 (12 to 24.7)	19.7 (12.5 to 26.5)	16.1 (6.6 to 25.1)	23.9 (15.2 to 33.3)

Cause	Location	Measure	Age-standardized rate (per 100,000)						% Change (1990 to 2019)		
			1990			2019					
			Both	Female	Male	Both	Female	Male	Both	Female	Male
	Iraq	Incidence	10 (7.2 to 13.2)	7.8 (5.2 to 11.1)	12.1 (8.9 to 15.8)	15.4 (11.5 to 20.2)	10.9 (7.3 to 15.3)	19.9 (14.7 to 25.9)	54.6 (48 to 62.2)	39.6 (33.1 to 46.6)	63.9 (55.3 to 73.8)
		Prevalence	223 (177.2 to 275.5)	205.1 (158 to 264.9)	240.9 (189.8 to 299.3)	319.9 (253.4 to 396.8)	272.9 (210.2 to 351.7)	366.5 (290.2 to 456.4)	43.5 (37.3 to 49.5)	33.1 (25.7 to 40.6)	52.2 (43.3 to 61.3)
		Deaths	2.7 (1.9 to 3.8)	2.3 (1.5 to 3.6)	3.2 (2.2 to 4.6)	2.3 (1.6 to 3.3)	1.6 (1.1 to 2.4)	3.1 (2 to 4.6)	-15.1 (-35.5 to 7.5)	-28.4 (-48.4 to -0.2)	-3.7 (-30.2 to 29.4)
		DALYs	95 (71 to 125.4)	90.8 (63.6 to 131.2)	100 (71.5 to 135.9)	82.5 (61.4 to 108.1)	69.9 (50.5 to 94)	96.2 (69.1 to 129.7)	-13.2 (-31.2 to 8.1)	-23 (-42.2 to 1.2)	-3.8 (-25.3 to 23.2)
		YLLs	77.8 (55.2 to 107.4)	72.2 (46.6 to 113)	84.1 (57.4 to 118.7)	56.7 (37.7 to 80.5)	44.2 (27.9 to 65.6)	70.2 (46.2 to 104.7)	-27.1 (-45.8 to -4.2)	-38.8 (-57 to -12.9)	-16.5 (-39.7 to 14)
		YLDs	17.2 (10.7 to 25)	18.5 (11.5 to 27.6)	15.9 (9.5 to 23.5)	25.8 (16.1 to 37.1)	25.7 (15.3 to 37.7)	25.9 (15.6 to 37.4)	50.3 (28.1 to 75.1)	38.8 (11.1 to 71.8)	63.5 (32.9 to 101)
	Jordan	Incidence	8.7 (6.6 to 11.4)	7.1 (4.9 to 10.1)	10.2 (7.6 to 13.3)	14.6 (11.3 to 18.6)	10.3 (7.1 to 14.6)	18.4 (14 to 23.6)	68.2 (58.4 to 79.2)	45.1 (36.3 to 53.6)	80.5 (67 to 95.6)
		Prevalence	202.2 (164.1 to 253.2)	187.9 (145.6 to 246.8)	215.5 (172.6 to 268.6)	298.6 (245.7 to 370.7)	253.9 (194.5 to 333.3)	338 (275.1 to 417.1)	47.7 (40.4 to 55.6)	35.1 (26.5 to 43.9)	56.9 (46.3 to 68)
		Deaths	2.7 (1.9 to 3.7)	2.6 (1.8 to 3.7)	2.8 (2 to 3.9)	2.4 (1.7 to 3.3)	2.1 (1.4 to 3.1)	2.6 (1.8 to 3.8)	-12.5 (-31.8 to 10.1)	-19.5 (-44.6 to 7.2)	-7.3 (-32.1 to 26)
		DALYs	82.7 (64.1 to 104.4)	83.4 (62.4 to 108.8)	82.4 (62.6 to 106.8)	76.9 (58.7 to 97.9)	70.6 (52.7 to 93)	82.5 (61.9 to 108.5)	-7 (-24.3 to 13.6)	-15.3 (-35.9 to 8.1)	0 (-22.9 to 29.2)
		YLLs	67.7 (49.8 to 90.1)	66.7 (46.8 to 91.9)	69 (50.3 to 93.2)	54.3 (40 to 74.6)	47.5 (33 to 67.2)	60.2 (42.4 to 84.9)	-19.8 (-37.9 to 1.9)	-28.9 (-50.7 to -2.4)	-12.7 (-36.1 to 17.2)
		YLDs	15 (9.8 to 22.4)	16.6 (10.6 to 24.9)	13.4 (8.3 to 20.8)	22.6 (14.3 to 34.2)	23.1 (14.5 to 34.8)	22.2 (13.5 to 34.2)	51.1 (29.9 to 76.2)	38.8 (12.5 to 71.1)	65.3 (33.7 to 101.9)

Cause	Location	Measure	Age-standardized rate (per 100,000)						% Change (1990 to 2019)		
			1990			2019					
			Both	Female	Male	Both	Female	Male	Both	Female	Male
	Kuwait	Incidence	9.6 (7.3 to 12.5)	7.7 (5.1 to 11.1)	10.7 (8.1 to 13.8)	13.4 (10.1 to 17.3)	9.8 (6.4 to 14.2)	16.2 (12.3 to 20.8)	39.6 (29.2 to 50.7)	27.3 (18.5 to 37.3)	50.6 (37.5 to 64.9)
		Prevalence	210.9 (169.5 to 260.9)	198.5 (148.8 to 257.7)	219.3 (176.4 to 270.5)	279.8 (222.6 to 348)	244.4 (181.1 to 320.2)	306.9 (242.8 to 380.5)	32.7 (24.8 to 41.8)	23.1 (15 to 32.4)	40 (29.8 to 51.7)
		Deaths	1.5 (1.1 to 2.1)	1.5 (1 to 2.1)	1.6 (1.2 to 2.2)	0.9 (0.6 to 1.2)	0.7 (0.5 to 1.1)	1 (0.7 to 1.4)	-44.6 (-54.5 to -32.9)	-51.8 (-63.1 to -33.5)	-41.4 (-53.7 to -27.1)
		DALYs	54 (43.1 to 67.2)	56 (43.3 to 71.1)	53 (41.4 to 67.4)	38.2 (29.1 to 49.2)	36.9 (27.3 to 49.8)	38.6 (28.8 to 51.2)	-29.2 (-40.7 to -15.6)	-34.1 (-47.5 to -18.1)	-27.1 (-41.3 to -10.9)
		YLLs	39.8 (30.6 to 52.2)	39.7 (28.8 to 52.7)	40.2 (30.3 to 53)	18.5 (13.6 to 25.2)	16.6 (11.4 to 23.8)	19.7 (14.1 to 27.9)	-53.5 (-61.4 to -43.3)	-58.1 (-67.6 to -43.2)	-51 (-61.4 to -37.8)
		YLDs	14.2 (9.2 to 20.6)	16.3 (10.2 to 24)	12.8 (7.9 to 18.7)	19.7 (12.4 to 29.4)	20.3 (12.3 to 31)	18.9 (11.5 to 28.8)	38.8 (17.4 to 62.9)	24.8 (2 to 54.8)	48.4 (17.4 to 86.1)
	Lebanon	Incidence	8.1 (6.1 to 10.8)	6.6 (4.4 to 9.5)	9.6 (7.3 to 12.7)	13.9 (10.8 to 18.3)	10.2 (6.9 to 14.6)	18.1 (14.2 to 23.3)	71.9 (63.2 to 81)	55.6 (46.6 to 65.5)	87.6 (75.5 to 101.5)
		Prevalence	195.9 (155.6 to 243.7)	182.7 (139.9 to 236.1)	208.9 (165.7 to 262.3)	295.9 (238.7 to 370)	258.3 (197.2 to 332.7)	338.1 (271.8 to 422.6)	51.1 (43.3 to 59.1)	41.4 (31.8 to 51.5)	61.8 (50.9 to 72.9)
		Deaths	1.8 (1.3 to 2.6)	1.6 (1.1 to 2.3)	2.2 (1.5 to 3.1)	1.3 (0.8 to 1.9)	1.1 (0.7 to 1.6)	1.5 (0.8 to 2.5)	-30.6 (-51.2 to -5.8)	-31.4 (-52.4 to -9.9)	-28.9 (-55.6 to 13.9)
		DALYs	60.7 (46.8 to 78)	57.5 (42.5 to 75.8)	64.3 (48.5 to 85)	51 (38.3 to 67.8)	47.5 (34.6 to 62.8)	55.7 (39.1 to 79.9)	-16 (-33.8 to 4.6)	-17.4 (-36.8 to 3.8)	-13.4 (-35.6 to 21.1)
		YLLs	47.4 (34.3 to 64.5)	43 (29.4 to 60.6)	52.2 (37.1 to 73.5)	30.6 (21 to 44.1)	27 (17.1 to 37.9)	35.1 (21.1 to 55.7)	-35.5 (-54.1 to -12.1)	-37.3 (-56.7 to -15.8)	-32.8 (-55.9 to 5.1)
		YLDs	13.3 (8.6 to 19.4)	14.5 (8.9 to 21.5)	12.1 (7.7 to 18.1)	20.4 (12.8 to 29.9)	20.5 (12.5 to 31.6)	20.6 (12.7 to 30.5)	53.5 (31.4 to 77.9)	41.9 (15.4 to 74)	70 (37 to 111.2)

Cause	Location	Measure	Age-standardized rate (per 100,000)						% Change (1990 to 2019)		
			1990			2019					
			Both	Female	Male	Both	Female	Male	Both	Female	Male
	Libya	Incidence	8.5 (6.4 to 11.3)	6.7 (4.6 to 9.8)	10.1 (7.5 to 13.1)	13.3 (10 to 17.4)	9.9 (6.8 to 14.2)	16.6 (12.4 to 21.5)	56.2 (49.5 to 63.3)	47.4 (40.2 to 55.2)	64.6 (55.5 to 75.2)
		Prevalence	195.1 (155.5 to 244.9)	179 (136.8 to 233)	209.4 (165.2 to 262.6)	278.5 (222.7 to 350.3)	243.9 (186.4 to 316.6)	311.5 (247.1 to 387.7)	42.8 (36.5 to 49.4)	36.3 (28.7 to 45.1)	48.7 (39.9 to 58.7)
		Deaths	1.8 (1.2 to 2.5)	1.6 (1 to 2.4)	1.9 (1.2 to 2.9)	1.7 (1.1 to 2.7)	1.6 (0.9 to 2.6)	1.8 (1.1 to 3.1)	-2.3 (-29.1 to 33.8)	1.2 (-30.8 to 36.4)	-5.3 (-34.3 to 39.2)
		DALYs	61 (44.8 to 79.9)	61.5 (43.7 to 83.7)	60.8 (43.5 to 84)	64 (45.3 to 87.6)	64.6 (42.4 to 90.2)	63.5 (44.5 to 94.9)	5 (-17.1 to 33.3)	5.1 (-18.9 to 32.4)	4.4 (-21.7 to 41)
		YLLs	47.7 (32.9 to 65.7)	46.9 (30.5 to 66.9)	48.5 (32.5 to 70.3)	43.8 (27.6 to 64.5)	43.7 (25 to 65.5)	44 (27.7 to 72.7)	-8 (-33 to 26.5)	-6.8 (-36.5 to 26.9)	-9.3 (-36.5 to 33.2)
		YLDs	13.3 (8.4 to 19.6)	14.6 (9 to 22)	12.3 (7.8 to 18.5)	20.2 (12.8 to 29.9)	20.9 (12.9 to 31.4)	19.5 (11.9 to 29.7)	51.5 (31.1 to 77.6)	43.5 (15.5 to 79.6)	58.5 (29.5 to 94.7)
	Morocco	Incidence	6.5 (4.9 to 8.9)	5.4 (3.5 to 7.8)	7.7 (5.8 to 10.3)	13.3 (10 to 17.7)	9.9 (6.5 to 14.4)	16.7 (12.7 to 21.9)	103 (94 to 113.3)	84.2 (74.4 to 94.4)	115.4 (102.7 to 128.3)
		Prevalence	163.2 (128.1 to 206.2)	155 (115 to 202.5)	171.5 (133.2 to 217.2)	286.6 (226.1 to 362.3)	252.4 (189.1 to 332.5)	320.8 (248.7 to 403)	75.6 (63.5 to 87.3)	62.9 (49.8 to 75.9)	87 (71.2 to 102.8)
		Deaths	1.4 (1 to 2.1)	1.2 (0.8 to 1.7)	1.7 (1.1 to 2.6)	1.6 (1 to 2.3)	1.4 (0.9 to 2)	1.8 (1.1 to 2.8)	11.4 (-15.7 to 39.2)	18.3 (-19.5 to 59.7)	4.6 (-23.3 to 37.8)
		DALYs	50.6 (39 to 64.4)	48.9 (36.1 to 63.9)	52.6 (39.2 to 70.7)	60.5 (44 to 78.2)	60.4 (43.7 to 80.6)	60.8 (42.7 to 82.6)	19.5 (-4.3 to 43.3)	23.4 (-7.3 to 57.2)	15.6 (-10.1 to 45.3)
		YLLs	39.1 (28.4 to 51.8)	36.1 (24.9 to 49.9)	42.3 (29.6 to 59.8)	38 (25.4 to 53.2)	37 (23.5 to 54)	39.3 (25.1 to 58.3)	-2.6 (-27 to 26.3)	2.4 (-32.5 to 42.8)	-7.2 (-33 to 22.3)
		YLDs	11.6 (7.2 to 16.9)	12.8 (7.8 to 19.6)	10.3 (6.3 to 15.3)	22.5 (14.1 to 33.1)	23.4 (14 to 35.2)	21.5 (13.2 to 32.4)	94.1 (66.7 to 123.3)	82.3 (45.5 to 123.4)	109.5 (74.1 to 149.1)

Cause	Location	Measure	Age-standardized rate (per 100,000)						% Change (1990 to 2019)		
			1990			2019					
			Both	Female	Male	Both	Female	Male	Both	Female	Male
	Oman	Incidence	6.5 (4.9 to 8.6)	5 (3.4 to 7.2)	7.5 (5.7 to 9.8)	12.6 (9.7 to 16.3)	8.7 (5.9 to 12.4)	15.2 (11.7 to 19.5)	94.7 (83 to 108.3)	73.4 (62.5 to 85.2)	101.4 (87.3 to 117.6)
		Prevalence	161.2 (129.3 to 199.8)	150.2 (114.4 to 196.3)	170.7 (136.6 to 213.3)	264 (212.8 to 323.4)	227 (174.5 to 295.1)	291.2 (234 to 357.7)	63.8 (53.4 to 75.7)	51.1 (39.6 to 62.4)	70.6 (56.9 to 85.1)
		Deaths	0.9 (0.6 to 1.3)	0.8 (0.5 to 1.1)	1.1 (0.7 to 1.8)	1 (0.7 to 1.4)	0.8 (0.5 to 1.2)	1.2 (0.8 to 1.8)	8 (-22.9 to 50.5)	7.6 (-24.5 to 51.7)	4 (-33.7 to 56.1)
		DALYs	31.6 (23.8 to 42.1)	31.3 (23.5 to 41.7)	33.5 (24.9 to 46.8)	38.4 (29.5 to 49.4)	37.8 (28.1 to 50)	40.5 (30.4 to 53.6)	21.5 (-3.1 to 48.6)	20.5 (-5.8 to 48.9)	20.7 (-10.5 to 54.3)
		YLLs	20.4 (13.5 to 29.8)	19.2 (12.8 to 28.4)	22.7 (14.5 to 34.8)	18.5 (13.4 to 25.3)	17.7 (12.6 to 24.2)	20.2 (13.9 to 29.1)	-9.3 (-33.5 to 24)	-7.5 (-33.6 to 30.2)	-11.3 (-40 to 30.4)
		YLDs	11.2 (7.3 to 16)	12.1 (7.6 to 18.3)	10.8 (7 to 15.7)	19.9 (12.7 to 28.9)	20 (12.1 to 29.7)	20.3 (12.9 to 30.9)	77.7 (53.8 to 104)	64.8 (34.6 to 102.5)	88.1 (55.7 to 122)
	Palestine	Incidence	10.1 (7.4 to 13.5)	7.5 (4.9 to 11.1)	12.9 (9.4 to 17.3)	14.4 (10.7 to 19)	9.8 (6.6 to 14.4)	19 (14.2 to 24.8)	43.3 (35.3 to 52.5)	30.7 (23.1 to 38.4)	47.3 (36.9 to 58.2)
		Prevalence	229.4 (182.5 to 289.4)	200 (152.4 to 263.3)	261 (202.5 to 332.2)	301.9 (242.6 to 379.6)	248.7 (189 to 324.7)	355.4 (279.1 to 447.5)	31.6 (25.7 to 38.1)	24.4 (17.8 to 30.8)	36.1 (27.6 to 45)
		Deaths	2.7 (1.9 to 3.8)	2.2 (1.5 to 3.2)	3.4 (2.3 to 4.9)	1.8 (1.3 to 2.5)	1.4 (1 to 1.9)	2.4 (1.7 to 3.4)	-33.2 (-48.6 to -13.2)	-35.9 (-51.3 to -14.7)	-29.6 (-47.1 to -8.2)
		DALYs	90.5 (67.2 to 120.2)	83.2 (60.8 to 112.2)	100.6 (73 to 138.9)	67.9 (53.6 to 85.7)	59 (45.4 to 75.1)	78.2 (60.1 to 100.3)	-25 (-39.2 to -8.4)	-29.1 (-43.8 to -10.2)	-22.2 (-38.3 to -2)
		YLLs	74.4 (53 to 103.9)	66.6 (45.9 to 95.5)	84.8 (58.3 to 121.9)	45.5 (33.9 to 60)	37.4 (27.3 to 49.3)	55.2 (39.9 to 76)	-38.7 (-52.2 to -20.6)	-43.9 (-57.4 to -25.6)	-35 (-49.9 to -13.2)
		YLDs	16.2 (10.5 to 23.6)	16.6 (10.6 to 24.2)	15.8 (10 to 23.3)	22.3 (14.7 to 32.3)	21.6 (13.4 to 33)	23.1 (14.9 to 34.2)	38.1 (18.3 to 59.8)	29.9 (5.1 to 58.8)	46.3 (16 to 82.1)

Cause	Location	Measure	Age-standardized rate (per 100,000)						% Change (1990 to 2019)		
			1990			2019					
			Both	Female	Male	Both	Female	Male	Both	Female	Male
	Qatar	Incidence	9 (6.8 to 11.4)	7 (4.9 to 9.8)	10 (7.5 to 12.9)	16.1 (12.2 to 20.4)	10.5 (7.2 to 14.9)	18.2 (13.7 to 23.3)	79.3 (69.1 to 90)	49.6 (40.2 to 59.5)	81.4 (69.3 to 92.9)
		Prevalence	201.1 (163.1 to 247.4)	187.2 (144.9 to 241.6)	211 (167 to 261)	314.5 (253.7 to 385.5)	260.8 (200 to 333.4)	336.3 (266.3 to 415.1)	56.4 (46.1 to 66.8)	39.3 (29.7 to 50)	59.4 (47.9 to 72.3)
		Deaths	2.3 (1.5 to 4)	2.3 (1.3 to 4.5)	2.5 (1.5 to 4.2)	2.1 (1.3 to 3.3)	2.6 (1.6 to 4.1)	2 (1.2 to 3)	-7.8 (-41.5 to 30.1)	13.3 (-38.4 to 67.2)	-21 (-45.4 to 12.1)
		DALYs	60.7 (44.8 to 88.4)	66 (46.5 to 104.8)	60.2 (43.3 to 87.7)	56.6 (41.8 to 74.2)	65.9 (47.1 to 89.2)	53.5 (39.2 to 70.6)	-6.7 (-29.7 to 19.2)	-0.1 (-32.4 to 31.9)	-11.1 (-33.1 to 15.4)
		YLLs	46.6 (31.9 to 71.8)	49.8 (31.6 to 88.6)	46.8 (31.6 to 72.9)	33.5 (22.3 to 49.2)	41.7 (26.9 to 62.1)	30.6 (20.2 to 46.2)	-28 (-50.7 to 1)	-16.1 (-48.5 to 22.8)	-34.5 (-53.8 to -6.6)
		YLDs	14.1 (8.9 to 20.7)	16.2 (10.1 to 24.9)	13.4 (8 to 19.9)	23.1 (14.2 to 33.8)	24.1 (14.5 to 36.9)	22.9 (13.9 to 34)	63.4 (38.2 to 94.5)	48.9 (19.5 to 84.5)	70.9 (38.2 to 115.1)
	Saudi Arabia	Incidence	9.8 (7.5 to 12.8)	7.6 (5.2 to 10.6)	11.4 (8.7 to 14.8)	18.8 (14.5 to 23.9)	13.5 (9.1 to 18.9)	22.3 (17.1 to 28.6)	90.7 (80.2 to 103.4)	77.3 (65 to 91.8)	96.1 (82.3 to 111)
		Prevalence	211.3 (171.1 to 256.6)	193.6 (148.2 to 243.8)	224.8 (179.8 to 274.7)	358.3 (290.2 to 432.2)	311.1 (236.1 to 394.9)	391.1 (311.5 to 478.4)	69.6 (59.5 to 80.7)	60.7 (49 to 73.5)	74 (61.8 to 87.8)
		Deaths	2.9 (2 to 4.1)	2.3 (1.5 to 3.3)	3.5 (2.2 to 5.1)	3 (2.1 to 4.3)	2.6 (1.7 to 3.8)	3.4 (2.2 to 4.9)	4.4 (-26.5 to 44.9)	14.7 (-23.7 to 60.6)	-3.3 (-35.1 to 42.8)
		DALYs	89.8 (65.6 to 120.8)	81.5 (59.1 to 111.3)	96.7 (68.5 to 135.5)	98.1 (74.7 to 129.3)	94.9 (68.7 to 127)	100.3 (73.8 to 135.8)	9.3 (-18.4 to 43.9)	16.3 (-16.6 to 54.8)	3.8 (-24.3 to 45)
		YLLs	74.8 (50.4 to 106.3)	65.3 (44 to 94.8)	82.5 (54 to 121.1)	71.5 (50.5 to 100.5)	67.1 (44.9 to 98.3)	74.5 (50.8 to 109.5)	-4.4 (-32.1 to 34.7)	2.7 (-32.5 to 48.4)	-9.7 (-38.5 to 33.2)
		YLDs	15 (9.7 to 21.9)	16.2 (10.2 to 23.7)	14.2 (8.8 to 21.1)	26.6 (17.4 to 38.9)	27.8 (17.4 to 42)	25.9 (16.1 to 38.5)	78.1 (52.6 to 106.5)	71.2 (37.2 to 110.3)	82.4 (48.8 to 125.3)

Cause	Location	Measure	Age-standardized rate (per 100,000)						% Change (1990 to 2019)		
			1990			2019					
			Both	Female	Male	Both	Female	Male	Both	Female	Male
	Sudan	Incidence	6.4 (4.7 to 8.5)	4.9 (3.3 to 7.1)	7.8 (5.7 to 10.2)	11 (8.2 to 14.5)	7.8 (5.3 to 11.3)	14 (10.4 to 18.3)	72.1 (64.3 to 80.8)	59 (49.8 to 68.6)	78.8 (67.9 to 89.7)
		Prevalence	156.4 (127 to 194.5)	144.1 (109.8 to 185.5)	168 (135.1 to 211.4)	239.7 (195.1 to 298.1)	207.5 (158.4 to 265.2)	268.7 (216.7 to 339.4)	53.3 (44.4 to 62.1)	44 (33.9 to 55.5)	59.9 (49.3 to 72.1)
		Deaths	2.3 (1.6 to 3.4)	1.9 (1.3 to 3)	2.7 (1.8 to 4.1)	2.1 (1.3 to 3.6)	1.7 (1.1 to 2.7)	2.4 (1.3 to 4.6)	-8.4 (-38.9 to 39)	-10.2 (-48.2 to 27)	-8.8 (-39.7 to 54.4)
		DALYs	80.8 (58.9 to 108.8)	78.2 (52.5 to 116.8)	83.1 (59.5 to 112.2)	74.3 (50.4 to 111.8)	68.3 (46.5 to 95.1)	79.3 (51.1 to 132.2)	-8 (-35.6 to 31.5)	-12.7 (-46.7 to 21)	-4.6 (-33.7 to 50.2)
		YLLs	70.2 (47.8 to 98.3)	66.8 (42.3 to 105.6)	73.2 (50.2 to 101.6)	56.6 (35.2 to 91.9)	50.1 (30.1 to 75.3)	62.1 (36.1 to 114.2)	-19.4 (-48.8 to 25.2)	-25 (-59.2 to 11.8)	-15.3 (-46.3 to 46.9)
		YLDs	10.7 (7 to 15.3)	11.4 (7 to 17.2)	9.9 (6.3 to 14)	17.8 (11.3 to 25.8)	18.2 (11.1 to 27.5)	17.2 (10.7 to 25.3)	66.9 (46.4 to 90.1)	59.7 (32.4 to 93.2)	74.8 (47 to 106.1)
	Syrian Arab Republic	Incidence	9.8 (7 to 13.4)	7.6 (5 to 11.2)	11.8 (8.4 to 15.8)	13 (9.8 to 17.1)	9.4 (6.4 to 13.6)	16.6 (12.4 to 21.6)	32.8 (23.7 to 44.8)	24.2 (15.9 to 34.3)	40.4 (28.7 to 54.2)
		Prevalence	227 (177.1 to 289.5)	203.8 (152.1 to 266.1)	248.8 (193.6 to 318.6)	275.7 (220.5 to 344.9)	237.3 (180.3 to 306.7)	314.7 (247.3 to 396.8)	21.5 (15.9 to 27.4)	16.5 (10.4 to 22.8)	26.5 (18.7 to 35.6)
		Deaths	2.5 (1.8 to 3.5)	2.2 (1.5 to 3)	2.8 (1.9 to 4)	1.7 (1.1 to 2.4)	1.5 (1 to 2.2)	1.8 (1.2 to 2.6)	-33.5 (-52.6 to -8.9)	-28.5 (-49.6 to -0.6)	-36 (-56.2 to -7.7)
		DALYs	96.8 (73 to 125.1)	90.4 (68.4 to 119)	102.5 (75.9 to 134.6)	64 (47.8 to 82.6)	60.7 (45.2 to 79)	67 (48.9 to 87.6)	-33.9 (-49.7 to -14.8)	-32.9 (-49.4 to -11)	-34.6 (-51.3 to -12.7)
		YLLs	80.5 (58 to 109)	72.8 (51.7 to 102.4)	87.6 (61.4 to 120.4)	44.1 (31 to 61.8)	40.6 (27.9 to 57.1)	47.8 (32.9 to 68.7)	-45.2 (-60.3 to -23.3)	-44.2 (-60.6 to -17.2)	-45.5 (-61.6 to -21.3)
		YLDs	16.3 (10.3 to 24.1)	17.7 (10.7 to 26.9)	14.9 (9.4 to 22.6)	19.8 (12.6 to 29.8)	20.1 (12.5 to 30.8)	19.3 (12.3 to 29.1)	21.8 (3.1 to 41.2)	13.8 (-8.4 to 39.4)	28.8 (1.6 to 61.7)

Cause	Location	Measure	Age-standardized rate (per 100,000)						% Change (1990 to 2019)		
			1990			2019					
			Both	Female	Male	Both	Female	Male	Both	Female	Male
	Tunisia	Incidence	7.2 (5.3 to 9.4)	5.7 (3.8 to 8.2)	8.7 (6.4 to 11.3)	12.4 (9.2 to 15.8)	8.8 (5.9 to 12.6)	16 (12 to 20.3)	71.5 (63.4 to 81)	54.7 (45.3 to 65.6)	83.9 (72.8 to 96.5)
		Prevalence	177.3 (141.9 to 218.1)	163 (123.6 to 212.6)	190.8 (150.9 to 235.5)	265.1 (214 to 327.8)	224.5 (169.6 to 290.2)	306.2 (243 to 377.3)	49.5 (41.7 to 57.6)	37.7 (28.8 to 47.4)	60.5 (49.9 to 71.7)
		Deaths	1.6 (1.2 to 2.3)	1.4 (1 to 2)	1.9 (1.3 to 2.8)	1.5 (0.9 to 2.3)	1.2 (0.7 to 2)	1.8 (1.1 to 2.8)	-8.5 (-36.6 to 25.1)	-9.9 (-38.7 to 25.5)	-6.3 (-36.9 to 33.8)
		DALYs	55.9 (43.9 to 69.9)	53.6 (41.1 to 68.6)	58.2 (44.3 to 75.1)	54.3 (39.9 to 73.2)	48.9 (34.6 to 66.7)	60.2 (42.7 to 82.6)	-2.8 (-22.4 to 21.5)	-8.8 (-29.7 to 15.5)	3.5 (-21 to 35.4)
		YLLs	43.3 (32.4 to 58.2)	40.3 (29.4 to 54)	46.3 (33.1 to 64.6)	35 (22.8 to 51.4)	30 (18.5 to 44.4)	40.4 (25.4 to 61.1)	-19.2 (-43 to 10.9)	-25.6 (-51 to 5.2)	-12.6 (-39.3 to 25)
		YLDs	12.6 (8.3 to 18.1)	13.3 (8.2 to 19.4)	11.9 (7.7 to 17.4)	19.3 (12.5 to 28)	18.9 (11.5 to 28.1)	19.7 (12.5 to 29.2)	53.1 (33.9 to 76.6)	41.9 (15.7 to 72.8)	65.9 (37.8 to 101)
	Turkey	Incidence	6.9 (5.2 to 9.3)	4.9 (3.3 to 7.3)	9 (6.7 to 11.7)	11.6 (9 to 15.1)	7.8 (5.4 to 11.3)	15.6 (12 to 19.8)	68.4 (55.8 to 83)	59.8 (46.6 to 75.1)	73.6 (59 to 91.5)
		Prevalence	173.1 (140.2 to 215.9)	150.1 (113.5 to 195.6)	196.5 (156.5 to 245.8)	248.7 (203.5 to 307.5)	205.3 (158.8 to 264.9)	293.5 (237.2 to 364.6)	43.7 (35.8 to 53.2)	36.8 (27.1 to 47.8)	49.4 (38.4 to 61.6)
		Deaths	1.9 (1.3 to 2.8)	1.4 (0.9 to 2.2)	2.4 (1.5 to 3.8)	1.1 (0.8 to 1.6)	0.9 (0.6 to 1.3)	1.4 (0.9 to 2)	-39.6 (-58.6 to -19.8)	-34.8 (-58.4 to -12.6)	-42.9 (-62.4 to -17.3)
		DALYs	66.7 (50.7 to 86)	57.2 (42.3 to 76.5)	77.2 (57 to 103.1)	44 (33.2 to 55.7)	38.7 (29.5 to 50.4)	49.9 (37 to 64.5)	-34 (-49.7 to -18.6)	-32.3 (-50.3 to -13.4)	-35.4 (-52.1 to -16.1)
		YLLs	54.4 (39.3 to 72.8)	44.9 (30.6 to 64.5)	64.9 (45 to 90.5)	26.2 (18.4 to 35.5)	21.6 (14.8 to 29.4)	31.2 (21.6 to 43)	-51.9 (-64.9 to -36.9)	-52 (-67.1 to -35.4)	-51.9 (-65.6 to -33)
		YLDs	12.3 (7.9 to 18.1)	12.3 (7.7 to 19)	12.4 (7.8 to 18.1)	17.9 (11.2 to 26.1)	17.2 (10.5 to 25.8)	18.7 (11.5 to 28.1)	45 (24.8 to 66.7)	39.5 (13.7 to 71.3)	50.9 (25 to 85.2)

Cause	Location	Measure	Age-standardized rate (per 100,000)						% Change (1990 to 2019)		
			1990			2019					
			Both	Female	Male	Both	Female	Male	Both	Female	Male
	United Arab Emirates	Incidence	11.1 (8.4 to 14.2)	7.7 (5.2 to 10.9)	13 (9.6 to 16.7)	17.7 (13.4 to 22.5)	11.1 (7.4 to 15.7)	20.6 (15.3 to 26.5)	59.3 (51.2 to 67.5)	43.8 (37.2 to 51.1)	58.7 (49.7 to 67.9)
		Prevalence	234.6 (189.6 to 288.7)	199 (153.2 to 255.7)	255.7 (204.7 to 316)	337.2 (273 to 413.9)	265.5 (205.8 to 341.5)	372 (296.8 to 459.4)	43.7 (37 to 50.8)	33.4 (25.4 to 41.3)	45.5 (37.2 to 54.2)
		Deaths	3.5 (2.3 to 4.9)	2.9 (1.8 to 4.5)	3.9 (2.3 to 5.7)	2.9 (1.7 to 4.8)	2.4 (1.2 to 3.6)	3 (1.8 to 5.7)	-18.3 (-44.4 to 19.8)	-17.7 (-53 to 19.8)	-22 (-48.2 to 24.7)
		DALYs	106 (77.6 to 140.1)	92.7 (63.7 to 128.8)	114.4 (79.5 to 156)	98 (69 to 145.7)	85 (55.3 to 117.7)	103.2 (68.4 to 166.3)	-7.6 (-33.1 to 28.4)	-8.3 (-41.7 to 26.2)	-9.8 (-37.2 to 32.9)
		YLLs	89.4 (61.7 to 122.8)	75.3 (47.1 to 111.7)	98.1 (64.1 to 138.9)	73.5 (46.5 to 120.2)	61.6 (33.7 to 92.5)	78.2 (46.8 to 139.7)	-17.8 (-45 to 24.3)	-18.2 (-55.2 to 23.2)	-20.3 (-49.4 to 27.9)
		YLDs	16.6 (10.3 to 23.9)	17.4 (10.9 to 25.6)	16.4 (9.8 to 24)	24.5 (15.2 to 35.3)	23.4 (13.9 to 34.4)	25 (15.4 to 36.9)	47.7 (24.8 to 75.8)	34.6 (8.2 to 65.1)	52.6 (23.9 to 90.6)
	Yemen	Incidence	6.3 (4.7 to 8.4)	4.7 (3.1 to 7)	7.9 (5.8 to 10.4)	10 (7.5 to 13.1)	7.3 (4.7 to 11)	12.8 (9.6 to 16.5)	58.6 (49.8 to 70.8)	54.4 (38.9 to 82.4)	61.5 (51.4 to 71.7)
		Prevalence	154.6 (124.2 to 195.6)	139.5 (105.3 to 182.5)	170 (134.6 to 214.8)	222.5 (177.6 to 279.3)	195.4 (146.7 to 263.5)	249.6 (197.9 to 312.5)	43.9 (35.1 to 55.3)	40 (26.8 to 61.8)	46.8 (37.4 to 56.7)
		Deaths	1.6 (1 to 2.4)	1.2 (0.8 to 2.1)	2.1 (1.3 to 3.2)	1.4 (0.9 to 2)	1.1 (0.7 to 1.7)	1.7 (1.1 to 2.6)	-10.1 (-33.5 to 21.9)	-11.2 (-38.3 to 23.6)	-15.6 (-37.7 to 17.3)
		DALYs	55.8 (39.5 to 78.3)	50.1 (33.5 to 79.3)	63.3 (44.3 to 91.6)	54.1 (39.7 to 71.8)	49.4 (35.5 to 69.2)	59.1 (42.5 to 81.3)	-3.1 (-27.6 to 27.4)	-1.4 (-30.3 to 32)	-6.5 (-30.7 to 27.3)
		YLLs	45 (29.1 to 68.4)	38.6 (23.1 to 66.9)	53.1 (34.3 to 80.1)	37.1 (24.5 to 53.8)	31.2 (19.4 to 48.4)	43.2 (28.2 to 62.7)	-17.7 (-42.1 to 17.3)	-19.3 (-46.7 to 20)	-18.6 (-42.4 to 19.3)
		YLDs	10.8 (7.1 to 16)	11.5 (7.4 to 17.6)	10.2 (6.7 to 15.1)	17.1 (10.9 to 25.8)	18.2 (11.3 to 27.6)	15.9 (10 to 24.3)	57.7 (37 to 81.4)	58.9 (30.5 to 99.2)	56.7 (29.5 to 84.5)

Cause	Location	Measure	Age-standardized rate (per 100,000)						% Change (1990 to 2019)		
			1990			2019					
			Both	Female	Male	Both	Female	Male	Both	Female	Male
Chronic kidney disease due to other and unspecified causes	North Africa and Middle East	Incidence	196 (180.7 to 214.5)	240.4 (220.9 to 264.2)	152.7 (140.7 to 165.9)	333.3 (308.8 to 359.2)	380.5 (352.6 to 409.2)	288.4 (265.9 to 312.6)	70.1 (66.1 to 74.3)	58.3 (53.8 to 63)	88.9 (84 to 94)
		Prevalence	5636.5 (5253 to 5996.7)	6645 (6198.5 to 7080.1)	4635.7 (4324.9 to 4925.9)	7772 (7296.1 to 8235.7)	8935.4 (8376.5 to 9450.8)	6670.7 (6241.7 to 7086.7)	37.9 (35.2 to 40.7)	34.5 (31.7 to 37.3)	43.9 (40.4 to 47.7)
		Deaths	9 (6.9 to 11.9)	10.1 (7.7 to 13.7)	7.8 (5.7 to 11)	7.6 (5.6 to 9.8)	8.9 (6.7 to 11.4)	6.3 (4.5 to 8.9)	-15.7 (-30.9 to -1.3)	-11.5 (-31.9 to 3.9)	-20.2 (-35.7 to 1.5)
		DALYs	279.9 (236.7 to 333)	326.3 (273.8 to 388)	233.9 (193.7 to 286.5)	237.2 (191.1 to 288.4)	278.4 (223.6 to 336.4)	197.6 (156.3 to 252.1)	-15.3 (-24.7 to -5)	-14.7 (-26.7 to -2.7)	-15.5 (-26.4 to -0.4)
		YLLs	228.6 (188.5 to 278.2)	257.6 (210.4 to 315.2)	199.8 (160 to 250.6)	165.8 (128.6 to 212.1)	189.6 (142.6 to 241.2)	142.7 (106.5 to 192.7)	-27.4 (-38.1 to -15.2)	-26.4 (-40.6 to -12.2)	-28.6 (-39.7 to -11.9)
		YLDs	51.3 (37.9 to 66.8)	68.7 (50.7 to 90.7)	34.1 (25.1 to 44.5)	71.4 (52.9 to 92.2)	88.7 (66.1 to 114.9)	54.9 (40.1 to 72.7)	39 (31.3 to 47.1)	29.1 (22.1 to 36.9)	61.3 (49.9 to 73.2)
	Afghanistan	Incidence	181.6 (165.3 to 200.7)	225.5 (204.3 to 250.7)	140 (127.1 to 154)	288.3 (262.3 to 319.3)	329.2 (299 to 366.9)	244.5 (221.5 to 269)	58.8 (52.6 to 66.1)	46 (39.1 to 55.6)	74.7 (66.2 to 83.6)
		Prevalence	5265.3 (4894.1 to 5650.6)	6286.9 (5821.2 to 6789.9)	4228.4 (3926.7 to 4535.6)	6911.6 (6413.7 to 7426)	8026.9 (7423.3 to 8668.7)	5777.8 (5349.7 to 6199.8)	31.3 (27.6 to 35.4)	27.7 (23.6 to 32.7)	36.6 (31.6 to 42.3)
		Deaths	14.2 (9.7 to 21)	16.3 (11.1 to 26.3)	12 (7.7 to 18.3)	12.1 (8.1 to 18.8)	14.2 (9.2 to 23.1)	9.9 (6.1 to 16.2)	-14.7 (-36.6 to 8.2)	-13.1 (-41.7 to 18.4)	-17.6 (-37.4 to 5.8)
		DALYs	468.2 (359.3 to 615.8)	571 (427.9 to 805.4)	360.7 (265.8 to 480.8)	381.7 (287.4 to 515)	463.6 (342.3 to 665.2)	297.8 (206.6 to 421)	-18.5 (-36.6 to 3.1)	-18.8 (-41.4 to 5.9)	-17.4 (-36.1 to 13)
		YLLs	415.9 (309.7 to 563.1)	496.5 (355.4 to 722.6)	331.4 (238.1 to 451)	306.4 (215.9 to 433.4)	362.3 (247.4 to 567.6)	248.7 (161.2 to 372.5)	-26.3 (-45.6 to -2.7)	-27 (-51.3 to 0.8)	-25 (-44.1 to 7.3)
		YLDs	52.4 (37.9 to 68.6)	74.5 (54 to 97.5)	29.3 (21 to 39.2)	75.3 (55.1 to 98)	101.3 (74.1 to 132)	49.1 (35.1 to 66.8)	43.8 (31.2 to 57.1)	36 (22 to 51.4)	67.7 (44.3 to 94.5)

Cause	Location	Measure	Age-standardized rate (per 100,000)						% Change (1990 to 2019)		
			1990			2019					
			Both	Female	Male	Both	Female	Male	Both	Female	Male
	Algeria	Incidence	202.9 (184.9 to 224.3)	253.7 (229.7 to 281.6)	150.8 (136.4 to 166.3)	337.6 (310.7 to 367.7)	390.4 (358.9 to 423)	286 (259.1 to 316.2)	66.4 (59.8 to 72.9)	53.9 (46.6 to 61.9)	89.6 (78.8 to 100.5)
		Prevalence	5673.1 (5278.5 to 6069.1)	6829.6 (6324.9 to 7344.3)	4558.4 (4241.8 to 4882.4)	7825.7 (7292.1 to 8357.6)	9121.1 (8465 to 9772.1)	6582.6 (6115.1 to 7060.4)	37.9 (33.7 to 42.1)	33.6 (28.6 to 38.3)	44.4 (38.5 to 50.7)
		Deaths	9.8 (6.8 to 14.3)	12.6 (8.4 to 21.2)	7.7 (5.2 to 11)	7.9 (5.4 to 11)	11.3 (7.7 to 16.9)	5.4 (3.5 to 8)	-19.7 (-37.3 to 3.9)	-10.1 (-33.8 to 21.3)	-29.8 (-47.6 to -1.4)
		DALYs	277.7 (209.2 to 381)	349.5 (255.7 to 547.8)	212.2 (157.8 to 276.8)	227.9 (177.1 to 292.3)	296.7 (228 to 399.6)	167.6 (126.9 to 223.1)	-17.9 (-33.3 to 1.6)	-15.1 (-35.8 to 7)	-21 (-39.7 to 8.2)
		YLLs	226 (160.3 to 329.5)	277 (187.5 to 475.2)	180.4 (127.7 to 244.5)	154.3 (112.3 to 212.1)	202.2 (140.6 to 299.3)	113.9 (81 to 164.8)	-31.7 (-46.7 to -11)	-27 (-46.7 to -1.4)	-36.9 (-54.4 to -6.8)
		YLDs	51.7 (37.5 to 68.4)	72.5 (52.3 to 96.3)	31.9 (22.5 to 42.8)	73.7 (54 to 96.7)	94.5 (68.5 to 123.7)	53.7 (38.2 to 72.9)	42.5 (28.1 to 57.9)	30.3 (15.3 to 47.4)	68.4 (44.5 to 98.5)
	Bahrain	Incidence	227.5 (208.6 to 248.2)	285.3 (259.7 to 312.6)	181.3 (164.5 to 198.5)	369.2 (339.1 to 400.7)	420.2 (386.1 to 455.1)	339.1 (310.5 to 371.5)	62.3 (54.8 to 70.1)	47.3 (39.4 to 55.4)	87.1 (75.6 to 98.7)
		Prevalence	6005.5 (5588 to 6397.5)	7292.6 (6747.1 to 7802.9)	4966 (4615.9 to 5302.5)	8261.7 (7698.1 to 8830.7)	9596.3 (8936.3 to 10267.5)	7394.7 (6849.1 to 7942.9)	37.6 (33.1 to 42.2)	31.6 (26.3 to 36.9)	48.9 (42.4 to 55.5)
		Deaths	9.1 (6.5 to 12)	10.6 (7.3 to 14.3)	7.4 (4.8 to 10.9)	8 (5.5 to 10.8)	9.5 (6.5 to 12.7)	6.5 (4.2 to 9.3)	-12.5 (-32.2 to 9.3)	-10.5 (-34.6 to 17.3)	-12.5 (-33 to 11.7)
		DALYs	224.4 (176.1 to 284)	275.7 (212.8 to 351.8)	179.5 (134.5 to 237.3)	206.2 (160.8 to 253.6)	243.7 (187.9 to 297)	177.3 (135 to 226.7)	-8.1 (-23.2 to 8.9)	-11.6 (-29.4 to 6.5)	-1.2 (-19.5 to 19.4)
		YLLs	170 (124.8 to 225.8)	199.4 (140.7 to 267.1)	143.2 (101 to 203)	131.1 (93 to 173.1)	151.5 (106.3 to 199.7)	114.7 (79.4 to 162)	-22.9 (-39.9 to -3.5)	-24 (-44.1 to 0.3)	-19.9 (-38.2 to 3.1)
		YLDs	54.4 (39.3 to 71.6)	76.3 (54.5 to 101.1)	36.4 (25.9 to 49.1)	75.1 (54.6 to 99.1)	92.2 (67.1 to 123.3)	62.6 (43.8 to 85.1)	38 (22.1 to 55.8)	20.7 (4 to 39.3)	72 (45.9 to 100.1)

Cause	Location	Measure	Age-standardized rate (per 100,000)						% Change (1990 to 2019)		
			1990			2019					
			Both	Female	Male	Both	Female	Male	Both	Female	Male
	Egypt	Incidence	207.4 (188.9 to 229.6)	270.1 (244.6 to 299.9)	145 (132.1 to 159.8)	352.2 (323 to 383.1)	416 (380.4 to 451.5)	295.7 (268.1 to 326)	69.9 (64.3 to 76.2)	54 (47.2 to 61.1)	104 (93.8 to 114.3)
		Prevalence	5690.2 (5285.6 to 6089.3)	6942.1 (6425 to 7491)	4440.2 (4124.6 to 4746)	7990 (7430.9 to 8558.7)	9486.5 (8770 to 10188.2)	6755.5 (6244.6 to 7271.3)	40.4 (36.7 to 44.5)	36.7 (32.4 to 41.2)	52.1 (46.2 to 59)
		Deaths	10.2 (6.2 to 14.1)	13.2 (7 to 19)	7.2 (4.9 to 10.5)	10.4 (5.8 to 15.8)	16.1 (7 to 24.4)	7.2 (4.2 to 12.3)	2 (-24.6 to 29.9)	21.4 (-18.2 to 57.3)	-0.2 (-31.5 to 34.8)
		DALYs	304.1 (211.2 to 387.9)	396.6 (249 to 524.5)	212.5 (156.5 to 288.2)	296.6 (193 to 405.6)	399.4 (227.8 to 569.2)	222 (150.4 to 329.8)	-2.5 (-20.6 to 18.9)	0.7 (-20.1 to 24.8)	4.5 (-17.3 to 31.6)
		YLLs	249.8 (159.5 to 332.5)	320 (174.5 to 443.4)	180.3 (123.3 to 254.9)	217.2 (121.8 to 325.1)	296.2 (130.9 to 461.1)	162.4 (91 to 271.5)	-13.1 (-34.6 to 12.1)	-7.5 (-36.3 to 21.7)	-9.9 (-34.9 to 20.4)
		YLDs	54.3 (39.8 to 72.1)	76.6 (55.9 to 102.5)	32.2 (23.2 to 43.6)	79.4 (58.7 to 105.1)	103.2 (74.2 to 137)	59.5 (42.8 to 81.4)	46.1 (31.6 to 61.9)	34.8 (20.3 to 51.9)	84.8 (59.1 to 115.1)
	Iran (Islamic Republic of)	Incidence	233 (212 to 255.2)	281 (255.7 to 310.3)	189.3 (172 to 207.7)	314.3 (288.9 to 341.2)	366.8 (338.1 to 396.5)	260.6 (239.9 to 283.5)	34.9 (31.1 to 39.2)	30.5 (25.7 to 35.5)	37.6 (33.9 to 41.8)
		Prevalence	6596.5 (6123.1 to 7033.7)	7643.2 (7096.4 to 8161.8)	5568.4 (5187.2 to 5925.2)	7948.3 (7456.9 to 8420.6)	9160.3 (8583.5 to 9703.7)	6749.7 (6330.4 to 7154.2)	20.5 (18.6 to 22.6)	19.8 (17.5 to 22.4)	21.2 (19.2 to 23.4)
		Deaths	6.3 (4.9 to 7.9)	6.7 (5.1 to 8.9)	5.8 (4.3 to 7.5)	4.8 (3.6 to 6.1)	5.4 (4 to 6.6)	4.3 (3.2 to 5.6)	-23.7 (-38.6 to -15.3)	-19.5 (-43.8 to -7.9)	-26.6 (-35.9 to -13.4)
		DALYs	225.1 (188.6 to 264.1)	245.4 (205.2 to 290.3)	203.5 (161 to 244)	160 (134.3 to 188.8)	176.1 (145.8 to 208.6)	144.3 (119.6 to 174.4)	-28.9 (-37 to -20.4)	-28.2 (-37.1 to -19.5)	-29.1 (-39 to -14.8)
		YLLs	168.3 (136.2 to 203.6)	173 (137.6 to 211.5)	162.7 (122.7 to 201.9)	100.1 (80.5 to 122.5)	104.2 (82.6 to 125.7)	96 (77 to 121)	-40.5 (-49.4 to -31.3)	-39.8 (-49.8 to -29.5)	-41 (-51.6 to -25.8)
		YLDs	56.8 (41.9 to 74.6)	72.4 (52.9 to 95.2)	40.8 (30.1 to 53.6)	60 (43.8 to 78.3)	72 (52.6 to 95)	48.3 (34.9 to 64.1)	5.6 (-2.8 to 14.2)	-0.7 (-10.3 to 9.1)	18.4 (6.7 to 29.7)

Cause	Location	Measure	Age-standardized rate (per 100,000)						% Change (1990 to 2019)		
			1990			2019					
			Both	Female	Male	Both	Female	Male	Both	Female	Male
	Iraq	Incidence	226.9 (207.5 to 250)	276.3 (250.9 to 305.4)	176.4 (160.2 to 194.8)	361 (332.8 to 392.8)	406.2 (373.6 to 442.7)	316 (286.5 to 345.4)	59.1 (53 to 64.9)	47 (39.9 to 54.1)	79.2 (70.2 to 89.9)
		Prevalence	6263.2 (5848.9 to 6694.1)	7425.7 (6915.9 to 7942.8)	5067 (4714.6 to 5443.5)	8473 (7906 to 9063.4)	9647.1 (8986.1 to 10350.7)	7273.9 (6752.5 to 7822.1)	35.3 (31.4 to 39)	29.9 (25.6 to 34.8)	43.6 (37.6 to 49.5)
		Deaths	11.3 (8 to 16.4)	12.7 (8.6 to 19.7)	9.8 (6.6 to 14.5)	9.7 (6.6 to 13.6)	9.7 (6.7 to 13.6)	9.7 (6 to 15.2)	-14.6 (-33.8 to 7.2)	-23.5 (-45.9 to 3.6)	-1.8 (-28.1 to 31.3)
		DALYs	351.6 (274.7 to 461.7)	411.8 (311.5 to 586.5)	290.3 (211.5 to 382.1)	292.1 (226.4 to 374.2)	314.6 (242.9 to 407.7)	269.8 (195.1 to 361.3)	-16.9 (-32.4 to 0.5)	-23.6 (-41.1 to -2.8)	-7.1 (-26.9 to 20.8)
		YLLs	286.9 (211.7 to 393.7)	324.6 (232.8 to 496.3)	248.7 (172.9 to 341)	203.9 (143.7 to 279.8)	206.3 (142.2 to 292.1)	201.9 (133 to 294)	-28.9 (-45.6 to -8.9)	-36.4 (-54 to -12.9)	-18.8 (-40.1 to 12.6)
		YLDs	64.6 (47.1 to 85.4)	87.2 (62.9 to 114.6)	41.6 (29.7 to 54.6)	88.2 (64.8 to 114.3)	108.3 (78 to 141.5)	67.9 (48.7 to 92)	36.4 (23.7 to 49.7)	24.1 (10.6 to 39.5)	63.1 (39.6 to 88.1)
	Jordan	Incidence	222.9 (205 to 242.2)	293.8 (269.6 to 318.7)	154.8 (139.8 to 171.7)	367.5 (342.5 to 396.4)	426.2 (394.5 to 459.1)	314.1 (288.2 to 346.3)	64.9 (56.9 to 72.8)	45.1 (36.4 to 53.6)	102.9 (88.1 to 118.8)
		Prevalence	6071.1 (5656.2 to 6463.5)	7454.3 (6926.4 to 7965)	4723 (4387.4 to 5070.4)	8332.2 (7815.7 to 8872.2)	9713.6 (9085.2 to 10344.4)	7113.9 (6637.7 to 7638.8)	37.2 (32.7 to 42)	30.3 (25 to 35.8)	50.6 (43.7 to 58.7)
		Deaths	8.8 (6.2 to 12)	11.5 (7.9 to 16.2)	6 (4.2 to 8.6)	7.6 (5.3 to 10.3)	9.9 (6.7 to 13.6)	5.6 (3.7 to 8.1)	-13.4 (-31.8 to 8.8)	-13.7 (-40.1 to 15.4)	-6.8 (-31.8 to 26.3)
		DALYs	242.4 (192.2 to 305)	314.6 (244 to 410.8)	172 (134.1 to 223.7)	224.3 (178.5 to 279.1)	277.9 (216.1 to 355)	176.9 (135.5 to 223.4)	-7.5 (-22.3 to 9.6)	-11.7 (-30.5 to 8.9)	2.8 (-17.1 to 29.9)
		YLLs	183.5 (136.2 to 243.1)	230.2 (164.9 to 319.8)	137.7 (102.2 to 187.5)	145.1 (105.9 to 194)	174.7 (124.5 to 241.8)	118.5 (83.6 to 165.8)	-20.9 (-38 to -0.1)	-24.1 (-46.9 to 2.2)	-14 (-35.5 to 16.3)
		YLDs	58.9 (42.8 to 78.1)	84.5 (60.9 to 112.7)	34.3 (24.5 to 46.1)	79.2 (57.4 to 103.7)	103.2 (74.2 to 136.5)	58.4 (40.7 to 79.3)	34.5 (20.8 to 49.7)	22.2 (7.2 to 37.9)	70.3 (46.1 to 99.8)

Cause	Location	Measure	Age-standardized rate (per 100,000)						% Change (1990 to 2019)		
			1990			2019					
			Both	Female	Male	Both	Female	Male	Both	Female	Male
	Kuwait	Incidence	222.5 (203.8 to 244.4)	306.2 (277.3 to 339.9)	172.9 (155.4 to 193.1)	332.9 (303.9 to 365.1)	398.2 (364.9 to 433.1)	285.9 (258.6 to 317.9)	49.6 (38 to 61.8)	30 (19.4 to 41.3)	65.3 (49 to 82.7)
		Prevalence	6141.5 (5751.4 to 6542.6)	7816.8 (7287.5 to 8368.5)	5016.5 (4654.1 to 5390.2)	7780.9 (7241 to 8341.7)	9322.9 (8637.3 to 9995.9)	6654 (6160.3 to 7162.3)	26.7 (20.5 to 33.5)	19.3 (12.5 to 26.2)	32.6 (24.6 to 41.4)
		Deaths	6.8 (5 to 8.5)	8.8 (6.3 to 11.4)	5.1 (3.6 to 6.8)	3.7 (2.6 to 5)	4.6 (3.2 to 6.5)	3.1 (2.1 to 4.3)	-45 (-54.8 to -32.2)	-47.5 (-59.9 to -28)	-38.6 (-50.5 to -24.6)
		DALYs	195.2 (159.1 to 233.5)	260.9 (208.2 to 316.9)	150.2 (120.6 to 184.7)	131.6 (105.1 to 161.3)	163 (127.6 to 203.9)	108.1 (83.2 to 135.6)	-32.6 (-41.5 to -23)	-37.5 (-47.7 to -23.8)	-28 (-39.5 to -14.5)
		YLLs	146.6 (114.7 to 183.8)	190.4 (141.9 to 245)	116.9 (90.2 to 149)	69.5 (50.9 to 91.9)	85.7 (61 to 117.3)	58.3 (40.5 to 80.1)	-52.6 (-60.9 to -41.9)	-55 (-65.3 to -38.3)	-50.2 (-60.7 to -37)
		YLDs	48.6 (35.3 to 64.2)	70.5 (50.2 to 91.9)	33.3 (23.7 to 45.2)	62.1 (44.3 to 81.3)	77.3 (54.9 to 101.7)	49.9 (34.9 to 67.6)	27.7 (13.1 to 44.4)	9.5 (-4.5 to 27.2)	49.9 (25.5 to 77.1)
	Lebanon	Incidence	203.2 (185 to 223.9)	253.4 (231.3 to 280)	152.9 (137.4 to 168.8)	364.8 (335.6 to 397.5)	406.4 (373.3 to 439.2)	316.6 (289.7 to 349.1)	79.5 (71.1 to 89.3)	60.3 (51.9 to 70.4)	107.1 (94.3 to 121.9)
		Prevalence	5831.2 (5441.3 to 6231.8)	6933.6 (6466.2 to 7431.3)	4657.5 (4313.1 to 5007)	8345.8 (7817 to 8891)	9425.7 (8835.2 to 10038.5)	7097.6 (6589.2 to 7641.7)	43.1 (38.4 to 48.5)	35.9 (30.7 to 41.3)	52.4 (45.6 to 59.9)
		Deaths	8.1 (5.8 to 10.9)	9.3 (6.5 to 13)	6.7 (4.7 to 9.5)	5.9 (4 to 8.1)	6.7 (4.4 to 8.9)	4.8 (2.7 to 8.2)	-26.8 (-48.1 to -6.3)	-27.4 (-49.2 to -6)	-27.9 (-56.3 to 14.7)
		DALYs	226.1 (178.7 to 283)	268.9 (208.4 to 344.2)	180.7 (138.5 to 233.2)	185.2 (145 to 233.4)	211.7 (159 to 259.1)	154.4 (109.7 to 219.4)	-18.1 (-34.4 to 0.5)	-21.3 (-38.4 to -3.9)	-14.5 (-35.5 to 17.8)
		YLLs	175.2 (131.4 to 228.9)	200.2 (143 to 272.3)	148.8 (109.7 to 202.7)	116.4 (80.4 to 156.1)	129.7 (86.7 to 171.8)	100.3 (61.1 to 164.3)	-33.5 (-51.9 to -13)	-35.2 (-54.6 to -15.3)	-32.6 (-55.6 to 5.5)
		YLDs	50.9 (36.9 to 66.8)	68.7 (49.2 to 90.4)	31.9 (22.5 to 43.3)	68.8 (49.4 to 89.4)	82 (58.8 to 107.4)	54.2 (37.8 to 73.4)	35.2 (20.7 to 51.5)	19.4 (4.1 to 37.1)	69.8 (43.6 to 97)

Cause	Location	Measure	Age-standardized rate (per 100,000)						% Change (1990 to 2019)		
			1990			2019					
			Both	Female	Male	Both	Female	Male	Both	Female	Male
	Libya	Incidence	204.3 (185.9 to 225.7)	257.7 (232.9 to 285.5)	157.7 (141.9 to 175.4)	336.5 (307.6 to 369.3)	390.3 (356.9 to 424.6)	285.2 (258.4 to 315.2)	64.7 (58.4 to 71.1)	51.5 (44.5 to 59.6)	80.8 (70.7 to 91.5)
		Prevalence	5814.7 (5418.1 to 6220.5)	7039.3 (6545.9 to 7564.9)	4754.2 (4422.8 to 5101.1)	7935.8 (7368.8 to 8470.3)	9264.6 (8617.8 to 9903)	6673.7 (6172.5 to 7165)	36.5 (32.8 to 40.3)	31.6 (27.4 to 36.3)	40.4 (35.2 to 46.2)
		Deaths	7.5 (5 to 10.5)	9.1 (6 to 13.2)	6 (3.9 to 8.8)	7.5 (4.6 to 11.1)	9.5 (5.5 to 14.4)	5.6 (3.3 to 9.4)	1.2 (-26 to 35)	4.3 (-28.2 to 39.2)	-5.8 (-34.4 to 36)
		DALYs	228.7 (178 to 288.4)	289.1 (220.1 to 370.1)	175.5 (134.1 to 231.8)	236.2 (170.2 to 314.3)	295.9 (205.2 to 391.4)	178.6 (128.8 to 260.1)	3.3 (-17.4 to 29)	2.4 (-20.6 to 27.3)	1.8 (-22.5 to 36.1)
		YLLs	176.7 (127.8 to 232.6)	215.5 (147.7 to 294.4)	142.6 (103.5 to 198.2)	163.3 (102.3 to 235.5)	203.2 (119.7 to 297.2)	124.5 (79 to 203)	-7.6 (-33.2 to 25.5)	-5.7 (-34.8 to 27.8)	-12.6 (-39.1 to 28.1)
		YLDs	52 (37.9 to 69)	73.6 (52.6 to 99.6)	33 (23.4 to 43.4)	72.9 (53.6 to 94.6)	92.7 (66.8 to 122.1)	54.1 (39 to 72.1)	40.3 (26.7 to 55.7)	25.9 (10.5 to 42.8)	64.1 (41.8 to 89.6)
	Morocco	Incidence	159.4 (144.4 to 176.2)	197 (177.1 to 219.5)	121.2 (109.4 to 134.4)	325.1 (297.9 to 356.1)	366.4 (334.8 to 402.9)	283.9 (257.6 to 311.9)	103.9 (96.3 to 112.7)	86 (76.4 to 96.1)	134.1 (122.4 to 148.8)
		Prevalence	5069 (4698.8 to 5415.7)	5985.4 (5520.3 to 6434.6)	4104.5 (3803.9 to 4412.3)	7694.4 (7193.1 to 8231.1)	8804 (8213.2 to 9422.6)	6562.3 (6095.1 to 7032.5)	51.8 (47 to 56.9)	47.1 (41.7 to 52.9)	59.9 (53.3 to 67.4)
		Deaths	8.8 (6.7 to 12.4)	9.5 (7 to 14.3)	8.2 (5.6 to 12.8)	10.3 (7.3 to 14.1)	11.9 (8.3 to 16.5)	8.7 (5.6 to 13.4)	16.6 (-11.7 to 44.7)	24.8 (-14 to 64.2)	6.3 (-21.4 to 39.5)
		DALYs	261.3 (215.5 to 321.4)	301.3 (244.4 to 377.6)	220.5 (169.7 to 281.8)	293.5 (228.3 to 374.5)	350 (263.1 to 448)	236.2 (174.3 to 318.9)	12.3 (-9.9 to 35.6)	16.2 (-12.5 to 45.2)	7.1 (-15 to 34.8)
		YLLs	216.3 (171.8 to 271)	239.6 (185.1 to 313.2)	192.9 (144 to 257.3)	214.9 (153.9 to 287.6)	251.8 (174.8 to 340.9)	177.5 (119.1 to 257.1)	-0.7 (-24.7 to 25.5)	5.1 (-28.1 to 42)	-8 (-31.3 to 22.1)
		YLDs	45.1 (32.7 to 59.2)	61.7 (44.5 to 81)	27.6 (19.4 to 37.3)	78.6 (57.4 to 103)	98.2 (71 to 129.4)	58.7 (41.2 to 79.4)	74.6 (56.9 to 94)	59.3 (40.7 to 80.1)	112.4 (81.3 to 147.4)

Cause	Location	Measure	Age-standardized rate (per 100,000)						% Change (1990 to 2019)		
			1990			2019					
			Both	Female	Male	Both	Female	Male	Both	Female	Male
	Oman	Incidence	168.7 (153.2 to 186.7)	209.8 (189.1 to 234.1)	134.1 (120.7 to 149)	327.1 (298.4 to 355.3)	376.1 (342.5 to 409.7)	291.9 (265 to 319.6)	93.9 (85 to 103.6)	79.2 (68.6 to 91.3)	117.6 (102.8 to 132.4)
		Prevalence	5191.8 (4816.7 to 5544.4)	6230.8 (5757.8 to 6694.7)	4360.8 (4031.7 to 4687.4)	7635.5 (7143.4 to 8164.7)	8976.7 (8370.8 to 9601.6)	6745 (6281.7 to 7233.8)	47.1 (42.2 to 52)	44.1 (38.2 to 50.3)	54.7 (48 to 62.1)
		Deaths	4 (2.7 to 5.9)	4.5 (3.1 to 6.6)	3.7 (2.2 to 5.7)	4.6 (3.2 to 6.1)	5.3 (3.7 to 7)	4 (2.7 to 5.7)	13.2 (-18.1 to 49.8)	16.9 (-17.9 to 59.4)	9.2 (-29.8 to 61.9)
		DALYs	131.1 (101.4 to 168.1)	167.2 (129.5 to 212)	100.2 (73.2 to 138.6)	160 (127.9 to 194.6)	206.1 (165.5 to 254.1)	125 (96.9 to 157)	22 (1.5 to 43.9)	23.3 (2 to 45.7)	24.7 (-5.3 to 58.5)
		YLLs	78.9 (53.9 to 112.9)	94.1 (65 to 132.9)	67 (42.4 to 102.6)	75.5 (56.4 to 99.3)	93.2 (67.9 to 123.3)	62.3 (42.5 to 86.1)	-4.2 (-28.6 to 26.6)	-1 (-29.9 to 36.4)	-7 (-37.7 to 35.4)
		YLDs	52.2 (37.6 to 68.1)	73 (52.9 to 95.9)	33.2 (23.8 to 44.8)	84.4 (62.4 to 110.9)	112.9 (81.8 to 149.5)	62.7 (45.2 to 84.8)	61.7 (47.2 to 78)	54.6 (39.4 to 72.4)	88.6 (62.4 to 118.1)
	Palestine	Incidence	231 (211.4 to 253.3)	270.6 (245.3 to 300)	184 (166.2 to 201.4)	353.5 (324.8 to 384.9)	390.6 (358.6 to 426.5)	316.8 (288 to 346.9)	53 (47 to 59.8)	44.3 (36.2 to 53.3)	72.2 (60 to 83.4)
		Prevalence	6210 (5767.7 to 6637.3)	7178.6 (6660.4 to 7677.3)	5074.9 (4713.6 to 5440.6)	8135.3 (7610.3 to 8702.2)	9116.2 (8527.8 to 9787.2)	7087.3 (6563.7 to 7602.4)	31 (27.3 to 35)	27 (21.9 to 32.2)	39.7 (33 to 45.9)
		Deaths	11.7 (8.4 to 15.7)	12.7 (9.2 to 17)	10.6 (7.3 to 14.9)	8.1 (5.9 to 10.6)	8.6 (6.3 to 11.2)	7.6 (5.3 to 10.4)	-31 (-46.6 to -11.9)	-32.6 (-47.6 to -11.8)	-28.3 (-46 to - 6.9)
		DALYs	343.8 (266.6 to 433.6)	393.7 (303.9 to 499.1)	289.2 (213.3 to 386.6)	248.1 (200.7 to 301.9)	273.5 (222.5 to 331.1)	220.9 (174.4 to 276.5)	-27.8 (-40.4 to -12.1)	-30.5 (-43.4 to -13.9)	-23.6 (-38.1 to -4.4)
		YLLs	282.2 (206.3 to 369.4)	313.2 (227.3 to 414.7)	248.9 (174.8 to 346)	169.2 (131.2 to 218.2)	178.6 (135.5 to 230.8)	159.3 (116.3 to 210.3)	-40 (-52.7 to -22.8)	-43 (-56.3 to -26.1)	-36 (-49.9 to - 17)
		YLDs	61.6 (45.3 to 80.4)	80.5 (58.6 to 106)	40.3 (28.6 to 53.1)	78.8 (58.9 to 103.6)	94.9 (70.5 to 125.2)	61.5 (43.9 to 82.3)	28 (15.5 to 41.2)	17.9 (3.9 to 33.5)	52.8 (32 to 77.3)

Cause	Location	Measure	Age-standardized rate (per 100,000)						% Change (1990 to 2019)		
			1990			2019					
			Both	Female	Male	Both	Female	Male	Both	Female	Male
	Qatar	Incidence	234.5 (211.7 to 258.2)	328 (295.4 to 361.9)	189.7 (170.1 to 210.3)	366.5 (335.5 to 399.3)	444.6 (407.6 to 481.1)	340.5 (309.2 to 372.5)	56.3 (49.5 to 63.3)	35.5 (28 to 43.4)	79.5 (69.8 to 89.7)
		Prevalence	6184.8 (5742.2 to 6641.3)	7951.1 (7345.1 to 8588.9)	5111.3 (4730.3 to 5490)	8053.7 (7465 to 8646.4)	10037.7 (9301.3 to 10818.4)	7406.1 (6848.6 to 7964.9)	30.2 (25.5 to 34.6)	26.2 (21 to 31.2)	44.9 (38.4 to 51)
		Deaths	11.6 (7.6 to 21.3)	15 (9.4 to 29.9)	8.3 (5 to 13.9)	9.7 (6.5 to 13.5)	19.4 (13.3 to 26.4)	6.9 (4.3 to 10.1)	-16.4 (-50.8 to 15.7)	29.7 (-31.5 to 86.9)	-17.1 (-43.6 to 17.3)
		DALYs	257.9 (193.6 to 387.3)	356.5 (259.5 to 588.2)	189.3 (135.8 to 268.5)	212.6 (161.2 to 272.1)	366.8 (279.1 to 469.3)	163.5 (120.5 to 212.3)	-17.6 (-41.5 to 3.2)	2.9 (-33.7 to 34.6)	-13.6 (-33.5 to 10.5)
		YLLs	200.5 (140 to 334.9)	271.1 (177.8 to 500.4)	149.8 (100.6 to 227.5)	140.7 (96.1 to 192.7)	268.1 (182.4 to 362.2)	100.4 (65.4 to 146.3)	-29.8 (-54.4 to -4.6)	-1.1 (-42.3 to 40.4)	-33 (-51.6 to -7.4)
		YLDs	57.4 (42.7 to 76.6)	85.4 (62.2 to 112.8)	39.5 (28.6 to 53.6)	71.9 (52.1 to 96.2)	98.8 (72.2 to 128.1)	63 (44 to 85.9)	25.2 (8.7 to 42.9)	15.7 (1.6 to 31.9)	59.7 (35.8 to 84.7)
	Saudi Arabia	Incidence	228.6 (209.7 to 249.7)	295.7 (267.2 to 325.4)	182 (164.4 to 200.5)	414.4 (386.3 to 442.4)	477.4 (444.3 to 512.9)	372.8 (344.5 to 401)	81.3 (72.6 to 90.7)	61.4 (50.9 to 73.2)	104.9 (91.8 to 117.9)
		Prevalence	6035.8 (5607.2 to 6425.4)	7497.2 (6957.2 to 8025.3)	4975.6 (4610.6 to 5317.6)	9103.1 (8536.7 to 9675.9)	10787.3 (10048.3 to 11532.4)	7975.1 (7438.6 to 8519.4)	50.8 (45.4 to 57.1)	43.9 (36.5 to 52.5)	60.3 (53.2 to 68)
		Deaths	11.4 (7.9 to 15.5)	12.4 (8.7 to 17)	10.7 (6.8 to 15.5)	12.4 (8.5 to 16.7)	15.1 (10.2 to 20.8)	10.4 (7 to 14.7)	9 (-21.5 to 45.7)	21.6 (-18 to 67.3)	-2.3 (-34.9 to 43.5)
		DALYs	313.2 (236.4 to 410.3)	370.6 (283.4 to 486.6)	273.3 (194.2 to 375.2)	331.8 (249.9 to 423)	416.2 (310.8 to 542.3)	273.7 (201.4 to 363.9)	5.9 (-19.1 to 35.5)	12.3 (-17.2 to 45.3)	0.1 (-26.1 to 38.3)
		YLLs	257.9 (182.1 to 353.8)	292.1 (206.5 to 399.3)	235.2 (156.5 to 334.6)	248.4 (173.9 to 342.5)	309.9 (210.3 to 432.3)	206.1 (141.2 to 291.9)	-3.7 (-31.5 to 30.9)	6.1 (-28.1 to 48.6)	-12.4 (-39.8 to 27.5)
		YLDs	55.3 (40.6 to 72.3)	78.5 (57.1 to 103)	38.1 (27.7 to 50.8)	83.4 (60.7 to 109.2)	106.3 (78 to 140.6)	67.6 (47.3 to 90.6)	50.7 (34.2 to 69.8)	35.4 (17.6 to 54.9)	77.2 (50.9 to 105.7)

Cause	Location	Measure	Age-standardized rate (per 100,000)						% Change (1990 to 2019)		
			1990			2019					
			Both	Female	Male	Both	Female	Male	Both	Female	Male
	Sudan	Incidence	156.5 (142.1 to 172.7)	194.9 (176.3 to 217.3)	121.7 (110.3 to 134.3)	278.4 (251.9 to 308.3)	320.5 (290.5 to 353.1)	242.7 (218.1 to 270)	77.9 (71.3 to 84.7)	64.5 (56.1 to 72.4)	99.4 (88.7 to 110.7)
		Prevalence	4988.7 (4631 to 5360.5)	5890 (5451.3 to 6361.8)	4111.6 (3807.2 to 4427.8)	6894.9 (6405.7 to 7399.6)	7946.7 (7382.1 to 8522.3)	5930.5 (5478.1 to 6373.4)	38.2 (34.1 to 42.6)	34.9 (30.3 to 40.2)	44.2 (38.4 to 50.3)
		Deaths	7.4 (5.2 to 10.7)	8.2 (5.5 to 12.6)	6.5 (4.4 to 10)	6.8 (4.3 to 10.7)	7.8 (4.9 to 11.7)	6 (3.4 to 10.9)	-7.1 (-38.2 to 33.4)	-5 (-44.5 to 31.1)	-8.3 (-39.4 to 53.9)
		DALYs	254 (195.6 to 340)	304.7 (223.5 to 439.5)	205.9 (150.9 to 271.5)	231.4 (168.1 to 320.1)	270.8 (195.9 to 359)	196.1 (135.1 to 307.8)	-8.9 (-34.1 to 24.2)	-11.1 (-42.7 to 18.6)	-4.7 (-33.1 to 52.1)
		YLLs	210.6 (152.5 to 294)	244.9 (164.2 to 373.4)	178.4 (124.4 to 241.7)	164.3 (104.8 to 246.5)	184.3 (114.3 to 268.6)	146.9 (90.5 to 251.2)	-22 (-49.7 to 17.4)	-24.7 (-58.1 to 10.8)	-17.7 (-47.9 to 47.4)
		YLDs	43.4 (31.5 to 58.5)	59.8 (43 to 81.3)	27.4 (19.8 to 36.4)	67.1 (48.7 to 87.9)	86.5 (62.7 to 114.5)	49.2 (35.5 to 66.7)	54.6 (40.9 to 70.3)	44.7 (28.6 to 61.7)	79.4 (54.5 to 107.6)
	Syrian Arab Republic	Incidence	208.3 (190.4 to 229.3)	267.7 (242.6 to 295.9)	154.6 (139.8 to 170.5)	329.9 (305.1 to 358.4)	383.8 (353.2 to 416.9)	276.2 (252.4 to 305.8)	58.3 (49.4 to 66.7)	43.4 (34.5 to 52.4)	78.7 (63.3 to 94.3)
		Prevalence	5838.3 (5430.1 to 6239.8)	7081.2 (6557.2 to 7589.1)	4693.3 (4352.5 to 5054.7)	7687.5 (7200.4 to 8172.7)	8918.7 (8297.2 to 9539)	6477.5 (6020.6 to 6947.3)	31.7 (27 to 36.3)	25.9 (20.4 to 31.1)	38 (31 to 45.8)
		Deaths	10.1 (7.5 to 13.6)	11.8 (8.7 to 15.8)	8.5 (6.1 to 11.7)	7.1 (5.1 to 9.7)	9.7 (6.9 to 12.9)	5.6 (3.8 to 7.8)	-29.1 (-48.3 to -5.4)	-17.6 (-41.2 to 9.8)	-34.4 (-55 to -7.4)
		DALYs	340.4 (275.8 to 417.6)	392.6 (316.2 to 480.2)	292.2 (227.1 to 366.6)	229.8 (179.6 to 289.8)	279.3 (219.6 to 354.7)	187.8 (143.1 to 243.2)	-32.5 (-46.6 to -15.2)	-28.9 (-43.9 to -9.9)	-35.7 (-50.6 to -16.2)
		YLLs	281.1 (218.1 to 353.1)	309.3 (237.1 to 392.4)	255.1 (191.7 to 330.8)	160.1 (118 to 213)	191.2 (138.9 to 259.5)	136.6 (97.1 to 191.9)	-43 (-57.9 to -22.2)	-38.2 (-55.7 to -14.1)	-46.5 (-61.3 to -24.9)
		YLDs	59.3 (43.2 to 78.6)	83.3 (60.4 to 110.3)	37 (26.5 to 50.3)	69.7 (50.6 to 91.8)	88 (63.9 to 116.3)	51.2 (35.9 to 69)	17.5 (5.9 to 31.6)	5.6 (-7 to 19.1)	38.3 (17.5 to 63.9)

Cause	Location	Measure	Age-standardized rate (per 100,000)						% Change (1990 to 2019)		
			1990			2019					
			Both	Female	Male	Both	Female	Male	Both	Female	Male
	Tunisia	Incidence	192.6 (175 to 211.6)	239.3 (216.4 to 262.5)	149.3 (134.6 to 165.8)	337.3 (309.8 to 365.2)	381.1 (348.3 to 413.6)	292.9 (266.8 to 318.5)	75.1 (67.3 to 83.1)	59.3 (50.4 to 69.1)	96.2 (84.4 to 108.9)
		Prevalence	5514.2 (5141.4 to 5895)	6553.4 (6105 to 7000.8)	4494.6 (4156.8 to 4840.5)	7764.5 (7251.9 to 8256)	8838.4 (8217.2 to 9430.7)	6629.3 (6162 to 7097.1)	40.8 (36.6 to 45.2)	34.9 (29.8 to 40.5)	47.5 (41.5 to 54.4)
		Deaths	7.2 (5.5 to 9.5)	7.9 (6 to 10.8)	6.5 (4.6 to 9)	6.8 (4.5 to 9.4)	7.4 (4.8 to 10.6)	6 (3.8 to 9.1)	-5.6 (-32.9 to 25.6)	-6 (-35.8 to 28.5)	-7 (-36.7 to 31.5)
		DALYs	212.4 (175.6 to 255.5)	246 (202.7 to 300.2)	179.9 (142.8 to 226.4)	195.3 (148.1 to 252.6)	214 (160.9 to 279.8)	174.8 (129.5 to 236.7)	-8.1 (-27.6 to 13.5)	-13 (-32.9 to 9.4)	-2.8 (-25 to 26.8)
		YLLs	168.3 (136.2 to 209.2)	189.8 (150 to 238.4)	147.6 (111.5 to 194.8)	132.3 (90.4 to 183.3)	142.1 (92.4 to 201.3)	121.4 (80.1 to 177.8)	-21.4 (-44.3 to 5.4)	-25.2 (-49.5 to 3.6)	-17.8 (-42.2 to 17.5)
		YLDs	44.1 (32.4 to 58.1)	56.1 (41.4 to 75.1)	32.2 (22.8 to 43.1)	62.9 (46.3 to 82.4)	72 (52.4 to 95.1)	53.5 (38.4 to 71.4)	42.7 (27.9 to 58.7)	28.2 (12.2 to 47.6)	66 (44.6 to 89.7)
	Turkey	Incidence	170.9 (158.3 to 185.3)	194.8 (180.2 to 213.4)	144.7 (133.1 to 156.4)	321.9 (296.1 to 350.6)	351.7 (323 to 381.3)	289.5 (263.4 to 320.6)	88.4 (75.5 to 101.9)	80.5 (66.9 to 95.9)	100.1 (84.2 to 119.3)
		Prevalence	5208.3 (4859.9 to 5582.7)	5919.1 (5520.4 to 6383.4)	4452.7 (4155.8 to 4762.4)	7421.8 (6934.1 to 7928.8)	8254.1 (7696.6 to 8801.5)	6533.9 (6058 to 7036.9)	42.5 (36.4 to 49.7)	39.4 (32.7 to 47.4)	46.7 (39 to 56.1)
		Deaths	9.1 (6.6 to 13.4)	9.3 (6.7 to 14)	8.9 (6 to 14.1)	6 (4.3 to 7.9)	6.6 (4.7 to 8.8)	5.1 (3.5 to 7.1)	-34.5 (-56 to -13)	-28.6 (-54.2 to -4.2)	-42.7 (-62.6 to -16.9)
		DALYs	292.7 (238.5 to 368.1)	307.5 (246.9 to 398.3)	276.6 (213.6 to 357.7)	181.1 (147.9 to 221.8)	198.8 (159.3 to 244.9)	160.4 (125.6 to 201.2)	-38.1 (-50.2 to -24.8)	-35.4 (-50.7 to -20.2)	-42 (-54.5 to -25.1)
		YLLs	247 (194.8 to 320.8)	250.5 (189.2 to 341.9)	243 (182.6 to 322.2)	117.5 (89.3 to 153.8)	124.4 (93.6 to 162.1)	108.2 (80.1 to 145.3)	-52.4 (-63.7 to -38.9)	-50.3 (-65.2 to -33.4)	-55.5 (-67.1 to -39)
		YLDs	45.7 (34 to 60.5)	57 (42.2 to 75.8)	33.6 (24.5 to 44.1)	63.6 (46.9 to 83.7)	74.4 (54.9 to 98.4)	52.1 (37.2 to 69.9)	39.1 (24.9 to 56.5)	30.4 (14.9 to 51.1)	55.2 (32.1 to 81.2)

Cause	Location	Measure	Age-standardized rate (per 100,000)						% Change (1990 to 2019)		
			1990			2019					
			Both	Female	Male	Both	Female	Male	Both	Female	Male
	United Arab Emirates	Incidence	253.9 (231.5 to 276)	328.4 (299.1 to 361.9)	209.6 (190 to 229.8)	375.9 (346.7 to 406.9)	459.2 (426.6 to 493.6)	345.4 (315.6 to 377.9)	48 (42.2 to 54)	39.8 (33.3 to 46.3)	64.8 (56.2 to 73)
		Prevalence	6375.7 (5923 to 6810.7)	8004.4 (7410.1 to 8642.1)	5446.9 (5055.3 to 5831.9)	8277.6 (7710.8 to 8877)	10178.7 (9493.1 to 10891.5)	7522 (6978.1 to 8095.2)	29.8 (26.2 to 33.7)	27.2 (22.8 to 31.5)	38.1 (32.5 to 43.9)
		Deaths	14.3 (8.6 to 19.7)	17.5 (10.5 to 25.4)	12 (7 to 17.5)	10.7 (6.1 to 16.8)	14.4 (7.3 to 21)	9.2 (5 to 16.7)	-25.1 (-49.7 to 5.2)	-17.9 (-51 to 17.7)	-23.3 (-48.3 to 19.3)
		DALYs	372.7 (279.7 to 480.6)	452.1 (325 to 621.2)	324.9 (232.2 to 440.8)	312.4 (212.9 to 450.6)	393.8 (262.7 to 521.9)	279.8 (184.2 to 453.4)	-16.2 (-39.2 to 12.9)	-12.9 (-43.1 to 17.7)	-13.9 (-37.6 to 25.6)
		YLLs	311.8 (217.1 to 415.6)	363 (236.7 to 529.6)	280.5 (188.9 to 395)	234 (144.5 to 368.5)	289.8 (163 to 422.6)	212.5 (124.2 to 384.4)	-24.9 (-50.1 to 9.6)	-20.2 (-54.8 to 16.8)	-24.2 (-50 to 22.1)
		YLDs	60.9 (44.2 to 81)	89.1 (64.6 to 116.4)	44.5 (31.7 to 60.4)	78.3 (57.2 to 104.6)	104 (76.1 to 136.9)	67.3 (47.9 to 91.8)	28.6 (15.1 to 42.1)	16.7 (3.9 to 31.4)	51.4 (30.5 to 74.8)
	Yemen	Incidence	155.6 (141.1 to 173.2)	186.2 (167.4 to 208.8)	121.9 (109.8 to 135.5)	259.8 (232.3 to 291.1)	297.3 (260.1 to 345.5)	222 (198.5 to 249.2)	66.9 (57.3 to 81.6)	59.7 (45.5 to 83.5)	82.1 (71.4 to 92.2)
		Prevalence	4923.7 (4561.6 to 5264.9)	5681 (5243.5 to 6130.6)	4043.6 (3751.2 to 4341.5)	6497.1 (6009.9 to 7016.7)	7447.3 (6797.5 to 8223.9)	5520.7 (5091.9 to 5955.3)	32 (26.6 to 39.4)	31.1 (23.8 to 43.5)	36.5 (30.7 to 42.6)
		Deaths	6.5 (4.5 to 9.9)	6.8 (4.4 to 11.2)	6.4 (4.1 to 9.6)	5.8 (4 to 8.3)	6.2 (4.3 to 9.1)	5.4 (3.5 to 8.1)	-11 (-32.9 to 16.4)	-8.2 (-34.7 to 25)	-15.2 (-37 to 14.8)
		DALYs	215.4 (159.1 to 298.9)	242.9 (173.2 to 363.8)	186.5 (131 to 258)	203.9 (156.1 to 263.8)	236.6 (181.9 to 308.1)	170.6 (124.7 to 228.1)	-5.3 (-25.9 to 20.7)	-2.6 (-28.2 to 27)	-8.5 (-30.8 to 25.1)
		YLLs	169.7 (117.2 to 247.6)	181.6 (116.8 to 303.5)	158.9 (105.5 to 226.9)	135.9 (95.1 to 191.9)	145.8 (101 to 213.1)	125.8 (83.6 to 180.7)	-19.9 (-41.6 to 10.2)	-19.7 (-45 to 14.7)	-20.8 (-43.5 to 16.3)
		YLDs	45.7 (33 to 61.3)	61.3 (43.7 to 82)	27.6 (19.4 to 37.3)	68 (48.6 to 89.3)	90.8 (63.8 to 122.9)	44.8 (32.1 to 61.1)	48.8 (32.4 to 68.8)	48.2 (28.5 to 76.5)	62.1 (39.9 to 86.5)

Data in parentheses are 95% Uncertainty Intervals (95% UIs)
