# Supplementary material for: Regional burden of chronic kidney disease in North Africa and Middle East during 1990–2019; Results from Global Burden of Disease study 2019
Source: Front Public Health. 2022 Oct 11;10:1015902. doi: 10.3389/fpubh.2022.1015902 (PMC9592811; doi:10.3389/fpubh.2022.1015902)
Supplement: Supplementary file 7 [file Data_Sheet_7.PDF]

Country	Measure	Metric	Age	Year						% Change (1990 to 2019)		
				1990			2019					
				Both	Female	Male	Both	Female	Male	Both	Female	Male
Afghanistan	Deaths	Number	0 to 9	241 (79 to 379)	132 (38 to 228)	109 (30 to 202)	368 (240 to 538)	170 (100 to 291)	198 (125 to 301)	52.5 (-9.4 to 339.1)	28.7 (-32.6 to 294.5)	81.3 (-9.1 to 534.1)
Afghanistan	Deaths	Number	10 to 24	120 (81 to 175)	83 (45 to 133)	37 (26 to 53)	247 (177 to 373)	153 (101 to 252)	94 (59 to 157)	105 (36.1 to 230.2)	83.9 (7.8 to 221.5)	152.3 (50.9 to 372.9)
Afghanistan	Deaths	Number	25 to 49	357 (234 to 501)	239 (133 to 376)	119 (80 to 170)	1059 (689 to 1591)	628 (320 to 1051)	432 (289 to 649)	196.5 (94.9 to 357.2)	163.1 (56.4 to 322.1)	263.7 (131.8 to 506.9)
Afghanistan	Deaths	Number	50 to 74	2046 (1475 to 2929)	989 (696 to 1527)	1057 (721 to 1579)	2474 (1805 to 3499)	1411 (964 to 2292)	1063 (724 to 1562)	20.9 (-17.5 to 68)	42.6 (-12.7 to 116.1)	0.6 (-32.4 to 42.7)
Afghanistan	Deaths	Number	75 plus	945 (674 to 1643)	375 (250 to 793)	569 (378 to 962)	1490 (1075 to 2507)	704 (458 to 1286)	785 (516 to 1316)	57.7 (16.7 to 105.3)	87.7 (16.1 to 176.6)	38 (1.3 to 87.7)
Afghanistan	Deaths	Rate	0 to 9	6.7 (2.2 to 10.5)	7.5 (2.2 to 13)	5.9 (1.6 to 10.9)	3 (1.9 to 4.3)	2.8 (1.7 to 4.8)	3.1 (2 to 4.7)	-55.6 (-73.6 to 27.8)	-62.3 (-80.3 to 15.5)	-47.5 (-73.7 to 83.6)
Afghanistan	Deaths	Rate	10 to 24	3.2 (2.1 to 4.6)	4.4 (2.4 to 7)	2 (1.4 to 2.8)	1.9 (1.4 to 2.9)	2.4 (1.6 to 4)	1.4 (0.9 to 2.3)	-40.2 (-60.3 to -3.6)	-45 (-67.7 to -3.8)	-28.1 (-57 to 34.7)
Afghanistan	Deaths	Rate	25 to 49	15.1 (9.9 to 21.2)	18.1 (10.1 to 28.5)	11.4 (7.7 to 16.4)	10.5 (6.8 to 15.8)	12.9 (6.6 to 21.6)	8.2 (5.5 to 12.4)	-30.7 (-54.4 to 6.9)	-28.5 (-57.5 to 14.7)	-27.8 (-54 to 20.5)
Afghanistan	Deaths	Rate	50 to 74	136.7 (98.5 to 195.7)	136.7 (96.2 to 211)	136.6 (93.2 to 204.1)	97.1 (70.9 to 137.4)	107.3 (73.3 to 174.3)	86.3 (58.8 to 126.8)	-28.9 (-51.5 to -1.2)	-21.5 (-52 to 18.9)	-36.8 (-57.5 to -10.4)
Afghanistan	Deaths	Rate	75 plus	562.1 (401.3 to 977.6)	489.5 (325.6 to 1034)	623.1 (414 to 1052.8)	544.8 (393.3 to 916.9)	508.2 (330.6 to 928)	582.4 (382.4 to 976.4)	-3.1 (-28.3 to 26.1)	3.8 (-35.8 to 53)	-6.5 (-31.4 to 27.2)
Afghanistan	DALYs	Number	0 to 9	21676 (7596 to 33781)	11905 (3849 to 20209)	9771 (2870 to 17680)	35947 (24756 to 50907)	17111 (10739 to 27840)	18835 (12270 to 27857)	65.8 (1.6 to 346.9)	43.7 (-21.1 to 304.9)	92.8 (-0.5 to 531.1)
Afghanistan	DALYs	Number	10 to 24	9917 (7183 to 14023)	6863 (4280 to 10560)	3053 (2214 to 4199)	24514 (18536 to 33576)	15595 (11220 to 22549)	8919 (6126 to 13593)	147.2 (78.7 to 259.8)	127.2 (49.6 to 260.6)	192.1 (91.2 to 384)

Country	Measure	Metric	Age	Year						% Change (1990 to 2019)		
				1990			2019					
				Both	Female	Male	Both	Female	Male	Both	Female	Male
			25 to 49	19389 (13128 to 26295)	13290 (7932 to 19780)	6099 (4251 to 8441)	63410 (44962 to 91643)	38684 (23659 to 60723)	24726 (17718 to 35246)	227 (129.9 to 370.4)	191.1 (92.4 to 322.4)	305.4 (171.8 to 532.2)
			50 to 74	57345 (41970 to 79224)	28596 (20735 to 41972)	28749 (19984 to 41918)	75039 (55796 to 102695)	42963 (29973 to 67674)	32075 (22502 to 45146)	30.9 (-9.2 to 79.6)	50.2 (-4.9 to 122.9)	11.6 (-23.5 to 56.7)
			75 plus	12792 (9293 to 21383)	5198 (3530 to 10374)	7593 (5115 to 12560)	20613 (15379 to 32934)	10030 (6828 to 16996)	10583 (7308 to 17099)	61.1 (21 to 109.1)	92.9 (25.3 to 179.6)	39.4 (1.8 to 90.3)
		Rate	0 to 9	267.2 (93.6 to 416.4)	301.2 (97.4 to 511.4)	234.9 (69 to 425)	132.8 (91.4 to 188)	130.3 (81.8 to 212)	135.1 (88 to 199.8)	-50.3 (-69.6 to 33.9)	-56.7 (-76.3 to 21.9)	-42.5 (-70.3 to 88.3)
			10 to 24	261.5 (189.4 to 369.7)	362.8 (226.3 to 558.3)	160.6 (116.5 to 220.9)	188.6 (142.6 to 258.3)	246.7 (177.5 to 356.7)	133.6 (91.8 to 203.6)	-27.9 (-47.9 to 5)	-32 (-55.2 to 7.9)	-16.8 (-45.5 to 37.9)
			25 to 49	821.7 (556.3 to 1114.3)	1006.5 (600.7 to 1498.1)	586.8 (409 to 812.1)	628.3 (445.5 to 908)	796 (486.8 to 1249.5)	472.5 (338.6 to 673.5)	-23.5 (-46.2 to 10)	-20.9 (-47.7 to 14.8)	-19.5 (-46 to 25.6)
			50 to 74	2023.2 (1480.7 to 2795.1)	2081.4 (1509.2 to 3054.9)	1968.5 (1368.4 to 2870.3)	1541.2 (1146 to 2109.2)	1713.6 (1195.5 to 2699.2)	1358.1 (952.7 to 1911.5)	-23.8 (-47.1 to 4.5)	-17.7 (-47.9 to 22.1)	-31 (-52.7 to -3.1)
			75 plus	5320.3 (3865 to 8893.6)	4726.2 (3209.7 to 9432.2)	5821.2 (3921.4 to 9628.3)	5181.3 (3865.7 to 8278.2)	5037.9 (3429.5 to 8536.9)	5324.9 (3676.9 to 8603.3)	-2.6 (-26.9 to 26.4)	6.6 (-30.8 to 54.4)	-8.5 (-33.2 to 24.9)
Algeria	Deaths	Number	0 to 9	187 (105 to 294)	102 (51 to 211)	86 (37 to 142)	94 (66 to 137)	48 (30 to 77)	46 (30 to 69)	-50.1 (-69.7 to -2.1)	-53.2 (-75.5 to -2.7)	-46.4 (-71.9 to 50)
			10 to 24	162 (111 to 240)	98 (61 to 173)	64 (43 to 92)	100 (73 to 142)	59 (39 to 90)	42 (27 to 66)	-38.1 (-56.9 to -3)	-40.3 (-61.9 to -0.6)	-34.6 (-61.2 to 28.4)
			25 to 49	383 (277 to 563)	230 (150 to 397)	153 (108 to 209)	698 (506 to 973)	426 (284 to 651)	272 (192 to 394)	82.5 (25.3 to 160.1)	85.4 (18.8 to 183)	78.1 (9.9 to 187.8)
			50 to 74	1567 (1123 to 2270)	825 (555 to 1382)	742 (526 to 1080)	3103 (2294 to 4329)	1705 (1215 to 2500)	1398 (978 to 2079)	98 (39.4 to 181.5)	106.7 (41.1 to 202.3)	88.4 (25.3 to 182)

Country	Measure	Metric	Age	Year						% Change (1990 to 2019)		
				1990			2019					
				Both	Female	Male	Both	Female	Male	Both	Female	Male
			75 plus	1260 (950 to 1734)	617 (414 to 1019)	643 (471 to 854)	4206 (3195 to 5496)	2317 (1665 to 3367)	1889 (1375 to 2550)	233.8 (153.1 to 346.7)	275.4 (166.2 to 441.9)	193.9 (104.7 to 336.9)
		Rate	0 to 9	2.5 (1.4 to 3.9)	2.8 (1.4 to 5.8)	2.3 (1 to 3.7)	1.1 (0.8 to 1.7)	1.2 (0.7 to 1.9)	1.1 (0.7 to 1.6)	-54.9 (-72.6 to -11.5)	-57.3 (-77.7 to -11.3)	-51.9 (-74.7 to 34.7)
			10 to 24	1.9 (1.3 to 2.8)	2.3 (1.5 to 4.1)	1.5 (1 to 2.1)	1 (0.7 to 1.5)	1.2 (0.8 to 1.9)	0.8 (0.5 to 1.3)	-45.9 (-62.3 to -15.3)	-47.7 (-66.6 to -12.8)	-43.1 (-66.3 to 11.7)
			25 to 49	5.9 (4.3 to 8.6)	7.2 (4.7 to 12.4)	4.6 (3.3 to 6.3)	4.3 (3.1 to 6)	5.3 (3.5 to 8.1)	3.4 (2.4 to 4.9)	-26.4 (-49.5 to 4.9)	-26.2 (-52.7 to 12.7)	-27.3 (-55.1 to 17.5)
			50 to 74	62.8 (45 to 91)	63.9 (43 to 107)	61.6 (43.6 to 89.6)	46.1 (34.1 to 64.3)	51.4 (36.6 to 75.3)	40.9 (28.6 to 60.9)	-26.6 (-48.3 to 4.4)	-19.6 (-45.1 to 17.6)	-33.5 (-55.8 to -0.5)
			75 plus	442.5 (333.7 to 608.9)	443 (297.5 to 731.3)	442 (323.7 to 587.5)	441.9 (335.7 to 577.4)	499.4 (358.8 to 725.6)	387.3 (281.9 to 522.9)	-0.1 (-24.3 to 33.6)	12.7 (-20.1 to 62.7)	-12.4 (-39 to 30.3)
	DALYs	Number	0 to 9	18082 (10835 to 27220)	9844 (5444 to 19175)	8238 (3948 to 13056)	11137 (8198 to 15414)	5736 (3873 to 8421)	5401 (3769 to 7673)	-38.4 (-60.8 to 8.6)	-41.7 (-68.6 to 9.8)	-34.4 (-63.1 to 54.1)
			10 to 24	14847 (10870 to 20640)	9076 (6301 to 14424)	5771 (4049 to 7903)	12608 (9230 to 16282)	7485 (5448 to 10083)	5123 (3590 to 7205)	-15.1 (-38.6 to 18.9)	-17.5 (-44.8 to 20.4)	-11.2 (-42.2 to 43)
			25 to 49	24267 (18456 to 33859)	14854 (10596 to 23522)	9413 (7047 to 12280)	53452 (41763 to 68282)	33014 (24606 to 44189)	20439 (15311 to 26869)	120.3 (66.1 to 189.1)	122.3 (57.4 to 204.5)	117.1 (52.7 to 211.4)
			50 to 74	45539 (33868 to 63875)	24755 (17691 to 39336)	20783 (15510 to 28997)	101033 (78166 to 133604)	55148 (41095 to 76782)	45885 (33897 to 65039)	121.9 (62.4 to 196.5)	122.8 (55.6 to 211.7)	120.8 (53 to 211.1)
			75 plus	17878 (13620 to 24230)	8945 (6297 to 14233)	8933 (6592 to 11915)	56494 (44625 to 72121)	30751 (23037 to 43032)	25743 (19407 to 34434)	216 (142.2 to 314.1)	243.8 (152.3 to 378.3)	188.2 (104.8 to 310.7)
		Rate	0 to 9	115.4 (69.1 to 173.7)	128.1 (70.8 to 249.5)	103.2 (49.5 to 163.6)	63 (46.4 to 87.2)	66.7 (45 to 97.9)	59.6 (41.6 to 84.6)	-45.4 (-65.2 to -3.7)	-48 (-72 to -2)	-42.3 (-67.5 to 35.6)

Country	Measure	Metric	Age	Year						% Change (1990 to 2019)		
				1990			2019					
				Both	Female	Male	Both	Female	Male	Both	Female	Male
			10 to 24	174 (127.4 to 241.8)	216.1 (150.1 to 343.5)	133.1 (93.4 to 182.3)	129 (94.4 to 166.6)	156.3 (113.8 to 210.5)	102.8 (72 to 144.6)	-25.8 (-46.4 to 3.9)	-27.7 (-51.6 to 5.5)	-22.8 (-49.7 to 24.4)
			25 to 49	372.9 (283.6 to 520.2)	463 (330.3 to 733.2)	285.2 (213.5 to 372.1)	331 (258.6 to 422.9)	409.6 (305.3 to 548.3)	252.7 (189.3 to 332.3)	-11.2 (-33.1 to 16.5)	-11.5 (-37.3 to 21.2)	-11.4 (-37.7 to 27.1)
			50 to 74	975.4 (725.4 to 1368.1)	1024.7 (732.2 to 1628.2)	922.5 (688.5 to 1287.1)	788 (609.7 to 1042.1)	873.2 (650.7 to 1215.7)	705.4 (521.1 to 999.8)	-19.2 (-40.8 to 8)	-14.8 (-40.5 to 19.2)	-23.5 (-47 to 7.8)
			75 plus	4838.9 (3686.5 to 6558.3)	5038.6 (3546.9 to 8017)	4654.2 (3434.8 to 6208.3)	3972.4 (3137.8 to 5071.2)	4461.7 (3342.4 to 6243.5)	3512.2 (2647.8 to 4698)	-17.9 (-37.1 to 7.6)	-11.5 (-35 to 23.2)	-24.5 (-46.4 to 7.6)
Bahrain	Deaths	Number	0 to 9	1 (1 to 2)	1 (0 to 1)	1 (0 to 1)	1 (1 to 1)	0 (0 to 1)	1 (0 to 1)	-33.5 (-54.3 to -1.2)	-38 (-65.9 to 8.6)	-29.8 (-56.2 to 20.3)
			10 to 24	1 (1 to 1)	1 (0 to 1)	0 (0 to 1)	2 (1 to 2)	1 (0 to 1)	1 (1 to 1)	61 (11.5 to 123.1)	18.5 (-24.8 to 84.2)	110.1 (30.3 to 215.8)
			25 to 49	4 (3 to 5)	1 (1 to 2)	3 (2 to 4)	17 (13 to 22)	5 (4 to 7)	12 (9 to 16)	319.8 (201.6 to 471.4)	263.6 (135 to 434.7)	351.4 (213.3 to 534.1)
			50 to 74	25 (21 to 31)	13 (10 to 16)	12 (10 to 17)	98 (76 to 125)	39 (29 to 49)	59 (44 to 81)	291.3 (190.5 to 424.6)	206.2 (114.4 to 329)	377.7 (244.4 to 578.4)
			75 plus	13 (11 to 17)	7 (5 to 9)	6 (5 to 9)	59 (46 to 72)	31 (23 to 38)	28 (22 to 37)	341.8 (231.6 to 471.3)	351.4 (224.3 to 530.9)	331.8 (203.8 to 513.5)
		Rate	0 to 9	1.1 (0.8 to 1.5)	1 (0.7 to 1.6)	1.2 (0.8 to 1.8)	0.6 (0.4 to 0.8)	0.5 (0.3 to 0.7)	0.7 (0.5 to 1)	-48.1 (-64.3 to -22.9)	-52 (-73.6 to -15.9)	-44.8 (-65.6 to -5.4)
			10 to 24	0.8 (0.6 to 1)	0.9 (0.6 to 1.2)	0.7 (0.5 to 1)	0.7 (0.5 to 0.8)	0.5 (0.4 to 0.7)	0.8 (0.6 to 1.1)	-16 (-41.8 to 16.4)	-38.6 (-61 to -4.5)	10.2 (-31.6 to 65.7)
			25 to 49	1.8 (1.4 to 2.5)	1.9 (1.4 to 2.7)	1.8 (1.3 to 2.6)	2.2 (1.7 to 2.9)	2.1 (1.5 to 2.8)	2.3 (1.7 to 3.1)	20.7 (-13.3 to 64.3)	9.8 (-29 to 61.5)	26.6 (-12.1 to 77.8)

Country	Measure	Metric	Age	Year						% Change (1990 to 2019)		
				1990			2019					
				Both	Female	Male	Both	Female	Male	Both	Female	Male
			50 to 74	64.2 (52.8 to 78.8)	74.3 (56.6 to 94.2)	56.5 (43.4 to 75.3)	36.1 (27.9 to 46)	40.6 (30.3 to 51.6)	33.6 (24.8 to 46.1)	-43.9 (-58.3 to -24.7)	-45.3 (-61.7 to -23.3)	-40.5 (-57.1 to -15.5)
			75 plus	484.9 (390.5 to 628.1)	466.1 (343.7 to 611.2)	506.2 (368.9 to 731.1)	504.3 (397.6 to 621.8)	502.1 (380.9 to 633.3)	506.7 (390.2 to 673.4)	4 (-21.9 to 34.5)	7.7 (-22.6 to 50.6)	0.1 (-29.6 to 42.2)
	DALYs	Number	0 to 9	142 (110 to 186)	66 (46 to 96)	75 (54 to 107)	126 (93 to 167)	58 (40 to 80)	68 (48 to 96)	-11.3 (-36.5 to 21)	-13.3 (-46.4 to 31.1)	-9.6 (-39.8 to 39.3)
			10 to 24	121 (94 to 152)	69 (52 to 87)	52 (39 to 71)	253 (185 to 338)	123 (86 to 170)	130 (92 to 176)	109 (63.4 to 159.7)	79.7 (29.2 to 137.6)	147.2 (80.3 to 226.3)
			25 to 49	346 (275 to 436)	143 (112 to 185)	203 (156 to 269)	1655 (1256 to 2136)	601 (457 to 772)	1055 (777 to 1393)	378.4 (278.5 to 482.5)	318.6 (230.7 to 421.9)	420.8 (291.8 to 562.6)
			50 to 74	730 (602 to 877)	371 (291 to 457)	358 (284 to 467)	3411 (2717 to 4250)	1288 (1011 to 1595)	2123 (1641 to 2735)	367.4 (264 to 506.3)	246.8 (157.1 to 365.5)	492.3 (340.5 to 695.1)
			75 plus	190 (154 to 244)	97 (74 to 125)	93 (69 to 132)	841 (674 to 1032)	434 (339 to 540)	406 (314 to 532)	341.5 (237.3 to 462.6)	346.8 (227.5 to 504.7)	335.9 (208.1 to 509.4)
		Rate	0 to 9	55 (42.8 to 72.2)	52.9 (36.6 to 76.8)	56.9 (41.1 to 80.7)	40.9 (30.3 to 54.4)	38.4 (26.8 to 53.4)	43.3 (30.9 to 61.3)	-25.5 (-46.7 to 1.6)	-27.4 (-55.1 to 9.8)	-23.8 (-49.3 to 17.3)
			10 to 24	93.5 (72.5 to 117.3)	110.1 (83 to 140.3)	78 (58.2 to 105.7)	101.9 (74.4 to 136.4)	102.6 (71.4 to 141.4)	101.2 (72.1 to 137.5)	9 (-14.8 to 35.5)	-6.9 (-33 to 23.1)	29.7 (-5.4 to 71.2)
			25 to 49	158.5 (126 to 199.8)	190 (148.1 to 244.7)	141.9 (109.5 to 188.4)	218.1 (165.5 to 281.5)	240.3 (182.7 to 308.8)	207.2 (152.7 to 273.7)	37.6 (8.9 to 67.5)	26.5 (-0.1 to 57.7)	46 (9.9 to 85.8)
			50 to 74	973.9 (802.7 to 1170.3)	1139.9 (892.4 to 1402.9)	846.1 (670.2 to 1103.5)	640.7 (510.3 to 798.2)	695.6 (545.8 to 861.2)	611.4 (472.7 to 787.7)	-34.2 (-48.8 to -14.7)	-39 (-54.8 to -18.1)	-27.7 (-46.3 to -3)
			75 plus	5046.2 (4075.7 to 6470.5)	4738.4 (3617.7 to 6067.4)	5413.2 (3993.2 to 7678.5)	4990.2 (3999.4 to 6129.4)	4885.3 (3816.5 to 6070.6)	5107.4 (3952.6 to 6691.7)	-1.1 (-24.4 to 26)	3.1 (-24.4 to 39.5)	-5.6 (-33.3 to 31.9)

Country	Measure	Metric	Age	Year						% Change (1990 to 2019)		
				1990			2019					
				Both	Female	Male	Both	Female	Male	Both	Female	Male
Egypt	Deaths	Number	0 to 9	608 (203 to 923)	377 (102 to 603)	231 (70 to 398)	254 (134 to 401)	120 (41 to 229)	133 (69 to 231)	-58.3 (-75.5 to 4.4)	-68.1 (-84.7 to -6.9)	-42.4 (-70.7 to 78.2)
			10 to 24	242 (157 to 309)	160 (85 to 229)	82 (56 to 118)	249 (144 to 383)	131 (53 to 209)	119 (68 to 200)	2.9 (-30.3 to 53)	-18.4 (-51.7 to 33.8)	44.6 (-10 to 125.1)
			25 to 49	783 (541 to 977)	439 (248 to 599)	345 (257 to 462)	1498 (855 to 2224)	748 (307 to 1169)	750 (432 to 1236)	91.3 (30.3 to 168.5)	70.5 (6.3 to 151.6)	117.6 (38.7 to 222.9)
			50 to 74	5006 (3446 to 6269)	2811 (1584 to 3813)	2195 (1642 to 2978)	12600 (6932 to 18760)	6881 (3112 to 10254)	5718 (3180 to 9771)	151.7 (62.4 to 251.4)	144.8 (44.3 to 245.9)	160.5 (54.2 to 287.3)
			75 plus	3081 (2046 to 4234)	1803 (976 to 2694)	1278 (947 to 1774)	7380 (4512 to 10417)	3755 (1681 to 5462)	3625 (2410 to 5782)	139.6 (72.5 to 214.9)	108.3 (31.9 to 190.4)	183.6 (89.6 to 299)
		Rate	0 to 9	3.9 (1.3 to 5.8)	4.9 (1.3 to 7.9)	2.9 (0.9 to 4.9)	1.1 (0.6 to 1.8)	1.1 (0.4 to 2.1)	1.2 (0.6 to 2)	-70.5 (-82.6 to -26)	-77.3 (-89.1 to -33.7)	-59.3 (-79.3 to 25.8)
			10 to 24	1.4 (0.9 to 1.8)	1.9 (1 to 2.8)	0.9 (0.6 to 1.3)	0.9 (0.5 to 1.3)	1 (0.4 to 1.5)	0.8 (0.5 to 1.4)	-37.8 (-57.9 to -7.6)	-50.8 (-70.9 to -19.3)	-12.5 (-45.6 to 36.2)
			25 to 49	4.8 (3.3 to 6)	5.5 (3.1 to 7.5)	4.2 (3.1 to 5.6)	4.5 (2.6 to 6.7)	4.6 (1.9 to 7.2)	4.4 (2.5 to 7.2)	-7.5 (-37 to 29.8)	-16.5 (-48 to 23.1)	4 (-33.7 to 54.4)
			50 to 74	84.3 (58.1 to 105.6)	95.5 (53.8 to 129.6)	73.4 (54.9 to 99.5)	92.3 (50.8 to 137.4)	107.1 (48.4 to 159.5)	79.1 (44 to 135.2)	9.4 (-29.4 to 52.8)	12.1 (-33.9 to 58.4)	7.9 (-36.2 to 60.4)
			75 plus	462.9 (307.4 to 636.2)	522.9 (283.2 to 781.5)	398.5 (295.3 to 553.1)	554.5 (339 to 782.8)	706.9 (316.5 to 1028.3)	453.3 (301.3 to 723)	19.8 (-13.7 to 57.5)	35.2 (-14.4 to 88.5)	13.7 (-24 to 60)
	DALYs	Number	0 to 9	56313 (20828 to 84266)	34742 (11208 to 55099)	21571 (7601 to 36246)	30559 (18851 to 45062)	15171 (7658 to 24868)	15387 (9171 to 24341)	-45.7 (-67.1 to 29.1)	-56.3 (-75.1 to 14.6)	-28.7 (-61.3 to 92.3)
			10 to 24	23782 (17017 to 29496)	15667 (10005 to 21138)	8115 (6045 to 10885)	33648 (23642 to 45740)	18894 (11742 to 26175)	14754 (9663 to 21815)	41.5 (8.6 to 86.5)	20.6 (-11.2 to 70.9)	81.8 (31.5 to 143.8)

Country	Measure	Metric	Age	Year						% Change (1990 to 2019)		
				1990			2019					
				Both	Female	Male	Both	Female	Male	Both	Female	Male
			25 to 49	50282 (38034 to 60803)	29315 (19413 to 37879)	20967 (16395 to 26912)	110495 (75541 to 149535)	58971 (35657 to 82882)	51524 (35630 to 76410)	119.7 (73.3 to 174.7)	101.2 (54.4 to 161.8)	145.7 (80.3 to 228.9)
			50 to 74	142133 (102257 to 174607)	80098 (48538 to 105464)	62035 (47467 to 82364)	377282 (230282 to 538702)	202015 (104868 to 290204)	175266 (105982 to 284088)	165.4 (85.7 to 251.8)	152.2 (62.3 to 244.7)	182.5 (87.5 to 301.5)
			75 plus	42303 (29378 to 56557)	25144 (14855 to 37321)	17159 (12970 to 23304)	104291 (68373 to 140875)	53175 (26942 to 74684)	51116 (35425 to 76665)	146.5 (82.1 to 215.6)	111.5 (39.5 to 191.5)	197.9 (107.6 to 309.6)
		Rate	0 to 9	162.4 (60.1 to 243)	205.6 (66.3 to 326)	121.3 (42.8 to 203.9)	66.4 (41 to 97.9)	67.9 (34.3 to 111.3)	65 (38.7 to 102.8)	-59.1 (-75.2 to -2.7)	-67 (-81.1 to -13.3)	-46.4 (-71 to 44.5)
			10 to 24	138.6 (99.2 to 171.9)	189.3 (120.9 to 255.3)	91.4 (68.1 to 122.5)	118.5 (83.2 to 161)	137.7 (85.6 to 190.8)	100.5 (65.8 to 148.6)	-14.5 (-34.4 to 12.7)	-27.2 (-46.4 to 3.1)	10 (-20.5 to 47.5)
			25 to 49	311.2 (235.4 to 376.3)	368.8 (244.2 to 476.6)	255.3 (199.7 to 327.7)	330.6 (226 to 447.5)	363.1 (219.6 to 510.4)	299.9 (207.4 to 444.8)	6.3 (-16.2 to 32.8)	-1.5 (-24.4 to 28.1)	17.5 (-13.8 to 57.2)
			50 to 74	1261.7 (907.7 to 1550)	1435.1 (869.7 to 1889.6)	1091.4 (835.1 to 1449.1)	1447 (883.2 to 2066.2)	1641.3 (852 to 2357.8)	1273.3 (770 to 2064)	14.7 (-19.7 to 52)	14.4 (-26.4 to 56.3)	16.7 (-22.6 to 65.8)
			75 plus	4423.2 (3071.8 to 5913.7)	5036.1 (2975.4 to 7475.2)	3753.8 (2837.4 to 5098.1)	5460 (3579.6 to 7375.4)	7195.4 (3645.6 to 10105.9)	4364.9 (3025.1 to 6546.6)	23.4 (-8.8 to 58)	42.9 (-5.8 to 96.9)	16.3 (-19 to 59.9)
Iran (Islamic Republic of)	Deaths	Number	0 to 9	602 (361 to 816)	294 (161 to 408)	309 (165 to 442)	96 (75 to 119)	39 (28 to 50)	57 (42 to 77)	-84.1 (-90.3 to -68.8)	-86.8 (-92.6 to -70.9)	-81.6 (-90 to -56.2)
			10 to 24	199 (176 to 221)	97 (81 to 110)	102 (86 to 116)	140 (129 to 151)	65 (58 to 72)	74 (67 to 82)	-29.8 (-37.6 to -19.6)	-33.1 (-42.2 to -21.9)	-26.7 (-38.2 to -10.7)
			25 to 49	488 (438 to 534)	229 (197 to 257)	259 (226 to 288)	921 (863 to 979)	385 (354 to 419)	536 (491 to 581)	88.8 (71 to 109.2)	68.4 (48.2 to 95.2)	106.8 (81.2 to 136.7)
			50 to 74	2742 (2503 to 3037)	1133 (999 to 1378)	1609 (1440 to 1798)	4818 (4474 to 5110)	2306 (2120 to 2465)	2511 (2311 to 2732)	75.7 (56.8 to 93.2)	103.5 (62.3 to 130.8)	56.1 (35.4 to 80.1)

Country	Measure	Metric	Age	Year						% Change (1990 to 2019)		
				1990			2019					
				Both	Female	Male	Both	Female	Male	Both	Female	Male
			75 plus	1237 (1080 to 1638)	662 (542 to 1043)	575 (491 to 659)	6572 (5714 to 7151)	3201 (2779 to 3499)	3371 (2890 to 3745)	431.3 (275.6 to 485)	383.6 (174.7 to 469)	486.1 (385.9 to 582.9)
		Rate	0 to 9	3.3 (2 to 4.4)	3.3 (1.8 to 4.5)	3.3 (1.8 to 4.7)	0.7 (0.5 to 0.8)	0.6 (0.4 to 0.7)	0.8 (0.6 to 1.1)	-79.2 (-87.3 to -59.2)	-82.6 (-90.3 to -61.8)	-76.1 (-87 to -43.1)
			10 to 24	1 (0.9 to 1.1)	1 (0.9 to 1.2)	1 (0.9 to 1.2)	0.8 (0.7 to 0.9)	0.8 (0.7 to 0.8)	0.8 (0.7 to 0.9)	-23.5 (-31.9 to -12.3)	-26.7 (-36.8 to -14.5)	-20.3 (-32.8 to -2.9)
			25 to 49	3.3 (3 to 3.6)	3.1 (2.7 to 3.5)	3.5 (3 to 3.8)	2.6 (2.4 to 2.7)	2.2 (2 to 2.4)	2.9 (2.7 to 3.2)	-22.5 (-29.8 to -14.1)	-30.4 (-38.7 to -19.3)	-15.5 (-26 to -3.3)
			50 to 74	47.2 (43.1 to 52.2)	42.1 (37.1 to 51.2)	51.6 (46.1 to 57.6)	33.2 (30.8 to 35.2)	31.5 (29 to 33.7)	34.8 (32.1 to 37.9)	-29.7 (-37.2 to -22.7)	-25.1 (-40.3 to -15.1)	-32.4 (-41.4 to -22)
			75 plus	327.9 (286.3 to 434.1)	316.2 (258.9 to 498.5)	342.6 (292.2 to 392.5)	312.5 (271.7 to 340)	311.1 (270.1 to 340)	313.8 (269.1 to 348.7)	-4.7 (-32.6 to 4.9)	-1.6 (-44.1 to 15.7)	-8.4 (-24 to 6.7)
	DALYs	Number	0 to 9	57823 (36418 to 76643)	28385 (16988 to 38318)	29438 (16646 to 41358)	13266 (10067 to 17327)	5969 (4272 to 8282)	7297 (5490 to 9454)	-77.1 (-85.5 to -59.8)	-79 (-87.5 to -60.6)	-75.2 (-85.5 to -49.1)
			10 to 24	22320 (18628 to 26598)	11918 (9835 to 14443)	10402 (8481 to 12471)	18867 (14870 to 23800)	9802 (7574 to 12549)	9065 (7262 to 11376)	-15.5 (-25.2 to -5.8)	-17.8 (-29.1 to -6.9)	-12.9 (-24.9 to 0.7)
			25 to 49	36395 (31789 to 41773)	18938 (16053 to 22248)	17458 (15139 to 19976)	78792 (66240 to 94991)	37846 (30856 to 46011)	40946 (35004 to 48723)	116.5 (97.2 to 136.1)	99.8 (79.1 to 122.6)	134.5 (110.5 to 161.6)
			50 to 74	84424 (77282 to 93003)	35643 (31708 to 41435)	48780 (44154 to 54428)	157794 (144175 to 173536)	74788 (67891 to 82495)	83005 (75119 to 92121)	86.9 (69.3 to 103.1)	109.8 (78.5 to 133.9)	70.2 (50.3 to 91.9)
			75 plus	16463 (14626 to 21041)	8989 (7570 to 13125)	7474 (6501 to 8447)	88302 (77869 to 98101)	43895 (38472 to 48770)	44407 (38741 to 50353)	436.4 (307.3 to 485.4)	388.3 (212.1 to 461.1)	494.1 (403.5 to 582.2)
		Rate	0 to 9	150.6 (94.9 to 199.7)	151 (90.4 to 203.9)	150.3 (85 to 211.1)	44.9 (34.1 to 58.7)	41.5 (29.7 to 57.6)	48.1 (36.2 to 62.4)	-70.2 (-81.2 to -47.7)	-72.5 (-83.7 to -48.5)	-68 (-81.2 to -34.2)

Country	Measure	Metric	Age	Year						% Change (1990 to 2019)		
				1990			2019					
				Both	Female	Male	Both	Female	Male	Both	Female	Male
			10 to 24	116.3 (97 to 138.5)	126.6 (104.5 to 153.4)	106.3 (86.7 to 127.4)	107.2 (84.5 to 135.2)	114 (88.1 to 145.9)	100.7 (80.7 to 126.4)	-7.8 (-18.5 to 2.7)	-10 (-22.4 to 1.9)	-5.3 (-18.3 to 9.5)
			25 to 49	246.1 (214.9 to 282.4)	259 (219.5 to 304.2)	233.4 (202.4 to 267.1)	218.8 (183.9 to 263.8)	213.9 (174.4 to 260)	223.5 (191.1 to 266)	-11.1 (-19 to -3)	-17.4 (-26 to -8)	-4.2 (-14 to 6.8)
			50 to 74	764.6 (699.9 to 842.3)	696.5 (619.6 to 809.7)	823.4 (745.3 to 918.7)	570.1 (520.9 to 627)	537.6 (488 to 593)	603 (545.7 to 669.2)	-25.4 (-32.5 to -19)	-22.8 (-34.3 to -14)	-26.8 (-35.3 to -17.4)
			75 plus	3007.6 (2672 to 3843.9)	2915.3 (2455 to 4256.5)	3126.6 (2719.3 to 3533.5)	2676.6 (2360.4 to 2973.7)	2741 (2402.3 to 3045.4)	2615.9 (2282.2 to 2966.2)	-11 (-32.4 to -2.9)	-6 (-39.9 to 8)	-16.3 (-29.1 to -3.9)
Iraq	Deaths	Number	0 to 9	198 (103 to 271)	104 (53 to 183)	94 (41 to 141)	102 (68 to 150)	45 (29 to 67)	57 (35 to 94)	-48.4 (-68.4 to 12.9)	-56.7 (-76.7 to -1.1)	-39.1 (-68.4 to 79.4)
			10 to 24	124 (90 to 172)	74 (48 to 118)	51 (33 to 71)	165 (113 to 227)	83 (52 to 122)	82 (52 to 119)	32.8 (-7.9 to 92.2)	13.2 (-29.9 to 77.9)	61.4 (-1.5 to 168.5)
			25 to 49	354 (248 to 481)	182 (119 to 289)	173 (106 to 236)	826 (493 to 1220)	386 (206 to 599)	440 (256 to 658)	133.4 (56 to 257.7)	112.9 (27.6 to 251.6)	154.9 (62.8 to 303.4)
			50 to 74	1556 (1215 to 2041)	794 (578 to 1182)	762 (557 to 1029)	3825 (2756 to 5083)	1709 (1168 to 2365)	2117 (1419 to 2909)	145.9 (70 to 236.7)	115.2 (37.3 to 214.7)	177.9 (81 to 303.9)
			75 plus	1231 (923 to 1792)	611 (435 to 984)	620 (443 to 978)	2968 (2278 to 4106)	1338 (1002 to 1780)	1630 (1110 to 2629)	141.2 (85.4 to 202.7)	119.1 (53.8 to 201.9)	162.9 (89.2 to 259.1)
		Rate	0 to 9	3.4 (1.8 to 4.7)	3.7 (1.9 to 6.4)	3.1 (1.4 to 4.7)	1.1 (0.7 to 1.6)	1 (0.6 to 1.5)	1.2 (0.7 to 2)	-67.8 (-80.3 to -29.6)	-72.9 (-85.4 to -38.1)	-62.1 (-80.3 to 11.6)
			10 to 24	2.2 (1.6 to 3)	2.6 (1.7 to 4.2)	1.7 (1.1 to 2.4)	1.2 (0.9 to 1.7)	1.3 (0.8 to 1.9)	1.2 (0.8 to 1.8)	-42.8 (-60.3 to -17.1)	-51 (-69.6 to -22.9)	-30.7 (-57.7 to 15.3)
			25 to 49	8.1 (5.6 to 10.9)	8.5 (5.6 to 13.5)	7.7 (4.7 to 10.5)	5.8 (3.4 to 8.5)	5.5 (3 to 8.6)	5.9 (3.5 to 8.9)	-28.6 (-52.3 to 9.4)	-34.6 (-60.8 to 8)	-22.4 (-50.4 to 22.9)

Country	Measure	Metric	Age	Year						% Change (1990 to 2019)		
				1990			2019					
				Both	Female	Male	Both	Female	Male	Both	Female	Male
			50 to 74	105.6 (82.4 to 138.4)	106.8 (77.7 to 158.9)	104.3 (76.3 to 141)	81.4 (58.7 to 108.2)	73 (49.9 to 101)	89.8 (60.2 to 123.4)	-22.9 (-46.7 to 5.6)	-31.6 (-56.4 to 0)	-13.9 (-44 to 25.1)
			75 plus	558 (418.5 to 812.4)	528.4 (376.3 to 851)	590.7 (421.9 to 931.7)	561.9 (431.3 to 777.4)	463 (346.6 to 615.9)	681.6 (464 to 1099.6)	0.7 (-22.6 to 26.4)	-12.4 (-38.5 to 20.7)	15.4 (-17 to 57.6)
	DALYs	Number	0 to 9	18708 (10861 to 25012)	9896 (5475 to 16371)	8812 (4231 to 13029)	12642 (8926 to 17502)	5901 (3982 to 8388)	6741 (4630 to 10060)	-32.4 (-56.1 to 30.6)	-40.4 (-65.8 to 19.4)	-23.5 (-56.5 to 86.4)
			10 to 24	11468 (8820 to 14987)	6885 (5000 to 10077)	4583 (3224 to 6149)	20441 (14877 to 26883)	11129 (7874 to 14922)	9313 (6362 to 12651)	78.3 (31.8 to 135.4)	61.6 (9.7 to 126.1)	103.2 (36.1 to 193.2)
			25 to 49	21726 (16418 to 27489)	11719 (8499 to 16786)	10007 (6784 to 13207)	60247 (43401 to 81463)	30574 (21201 to 41508)	29673 (20424 to 41446)	177.3 (105.7 to 276.6)	160.9 (85.1 to 270.9)	196.5 (111.6 to 321.6)
			50 to 74	44583 (35582 to 56957)	23096 (17390 to 32843)	21488 (15992 to 28195)	118079 (89511 to 150300)	53880 (38885 to 71713)	64199 (45132 to 84849)	164.8 (94.6 to 253.1)	133.3 (60.4 to 225.9)	198.8 (108.9 to 321.2)
			75 plus	15633 (12129 to 22016)	7774 (5746 to 11880)	7859 (5699 to 12040)	39862 (31896 to 52324)	18350 (14373 to 23543)	21513 (15249 to 32719)	155 (99.6 to 218.3)	136 (69.7 to 215.4)	173.7 (98.1 to 271.4)
		Rate	0 to 9	146.2 (84.9 to 195.5)	158.4 (87.7 to 262.1)	134.6 (64.6 to 198.9)	64.6 (45.6 to 89.4)	61.9 (41.8 to 88)	67.1 (46.1 to 100.1)	-55.8 (-71.3 to -14.6)	-60.9 (-77.6 to -21.7)	-50.1 (-71.6 to 21.5)
			10 to 24	201.5 (155 to 263.3)	247.6 (179.8 to 362.4)	157.4 (110.7 to 211.2)	154.8 (112.7 to 203.6)	173.4 (122.7 to 232.4)	137.3 (93.8 to 186.5)	-23.2 (-43.2 to 1.5)	-30 (-52.5 to -2.1)	-12.8 (-41.6 to 25.8)
			25 to 49	494.3 (373.5 to 625.4)	547.6 (397.1 to 784.4)	443.7 (300.8 to 585.6)	419.3 (302 to 566.9)	439 (304.4 to 596)	400.7 (275.8 to 559.6)	-15.2 (-37.1 to 15.2)	-19.8 (-43.1 to 14)	-9.7 (-35.6 to 28.4)
			50 to 74	1600.1 (1277 to 2044.1)	1645.8 (1239.2 to 2340.5)	1553.6 (1156.2 to 2038.6)	1318.5 (999.5 to 1678.3)	1209.8 (873.1 to 1610.2)	1426.1 (1002.5 to 1884.8)	-17.6 (-39.4 to 9.9)	-26.5 (-49.5 to 2.7)	-8.2 (-35.8 to 29.4)
			75 plus	4707.3 (3652.3 to 6629.2)	4394.3 (3248.2 to 6715.3)	5064 (3672.2 to 7758.3)	5085.6 (4069.2 to 6675.5)	4225.9 (3310 to 5421.9)	6153.4 (4361.7 to 9358.7)	8 (-15.4 to 34.9)	-3.8 (-30.8 to 28.5)	21.5 (-12.1 to 64.9)

Country	Measure	Metric	Age	Year						% Change (1990 to 2019)		
				1990			2019					
				Both	Female	Male	Both	Female	Male	Both	Female	Male
Jordan	Deaths	Number	0 to 9	14 (11 to 19)	7 (5 to 11)	7 (4 to 10)	17 (13 to 24)	9 (6 to 13)	8 (6 to 12)	21.7 (-21.4 to 86.7)	25.7 (-28.2 to 107.6)	17.6 (-26.6 to 105.5)
			10 to 24	18 (14 to 22)	10 (7 to 13)	8 (6 to 10)	28 (22 to 34)	14 (10 to 19)	13 (10 to 18)	57.7 (14.2 to 120.1)	42.9 (-8.4 to 118.9)	77.2 (12.5 to 176.8)
			25 to 49	44 (35 to 53)	21 (16 to 28)	23 (17 to 29)	161 (132 to 197)	61 (45 to 82)	100 (76 to 131)	267.4 (179.3 to 402.3)	185.9 (81.5 to 333)	344.1 (209.8 to 560.4)
			50 to 74	218 (181 to 263)	120 (93 to 154)	98 (76 to 124)	892 (736 to 1078)	440 (339 to 563)	452 (342 to 592)	308.2 (209.6 to 437.8)	265.4 (149 to 417.5)	360.8 (219.7 to 584.3)
			75 plus	146 (119 to 176)	85 (65 to 110)	61 (48 to 75)	715 (587 to 856)	374 (290 to 466)	341 (261 to 433)	391.3 (279.1 to 528.7)	341.4 (203.5 to 521)	460.9 (294.5 to 679.4)
		Rate	0 to 9	1.2 (0.9 to 1.6)	1.3 (0.9 to 1.9)	1.2 (0.7 to 1.7)	0.7 (0.5 to 1)	0.8 (0.5 to 1.1)	0.7 (0.5 to 0.9)	-42.2 (-62.7 to -11.4)	-40.4 (-66 to -1.6)	-44.2 (-65.1 to -2.4)
			10 to 24	1.3 (1 to 1.6)	1.6 (1.1 to 2.1)	1.1 (0.8 to 1.4)	0.8 (0.6 to 1)	0.9 (0.6 to 1.1)	0.7 (0.5 to 1)	-39.1 (-55.9 to -15)	-45.1 (-64.8 to -15.9)	-31.3 (-56.4 to 7.2)
			25 to 49	4.8 (3.9 to 5.8)	4.9 (3.7 to 6.4)	4.6 (3.6 to 6)	4 (3.2 to 4.9)	3.4 (2.5 to 4.5)	4.5 (3.4 to 5.8)	-17 (-36.9 to 13.5)	-31.7 (-56.6 to 3.4)	-4.2 (-33.2 to 42.4)
			50 to 74	74.2 (61.5 to 89.2)	86.4 (66.6 to 110.8)	63.2 (49.1 to 80.1)	62.4 (51.5 to 75.4)	65.9 (50.7 to 84.3)	59.3 (44.9 to 77.7)	-15.9 (-36.2 to 10.8)	-23.7 (-48 to 8)	-6.2 (-34.9 to 39.3)
			75 plus	554.9 (452.9 to 670.1)	612 (467.9 to 795.1)	491 (389.1 to 606.9)	485.9 (398.9 to 580.9)	528 (408.5 to 656.9)	446.7 (341.3 to 567)	-12.4 (-32.4 to 12)	-13.7 (-40.7 to 21.4)	-9 (-36 to 26.4)
	DALYs	Number	0 to 9	1509 (1160 to 1922)	782 (557 to 1109)	727 (478 to 995)	2284 (1686 to 3030)	1213 (857 to 1650)	1071 (771 to 1436)	51.3 (7.7 to 109.5)	55.1 (1 to 127.7)	47.2 (-0.9 to 125)
			10 to 24	1802 (1472 to 2163)	1062 (832 to 1343)	740 (573 to 930)	4001 (3078 to 5131)	2262 (1670 to 2973)	1738 (1277 to 2332)	122 (75 to 178)	113.1 (58.3 to 179)	134.8 (68.7 to 223.5)

Country	Measure	Metric	Age	Year						% Change (1990 to 2019)		
				1990			2019					
				Both	Female	Male	Both	Female	Male	Both	Female	Male
			25 to 49	2936 (2465 to 3458)	1545 (1244 to 1912)	1392 (1131 to 1725)	12708 (10469 to 15687)	5744 (4467 to 7312)	6964 (5505 to 8826)	332.8 (255.7 to 432.4)	271.8 (177.9 to 380.5)	400.5 (279.7 to 570.7)
			50 to 74	6460 (5438 to 7627)	3559 (2836 to 4413)	2901 (2288 to 3609)	27992 (23514 to 33375)	13628 (10928 to 17019)	14364 (11162 to 18133)	333.3 (242.6 to 449.8)	282.9 (174.5 to 416.6)	395.1 (253.9 to 595)
			75 plus	1879 (1549 to 2269)	1117 (877 to 1421)	762 (610 to 939)	10014 (8334 to 11940)	5224 (4170 to 6426)	4790 (3693 to 6011)	432.9 (317.4 to 573.3)	367.7 (230.9 to 533.7)	528.5 (354.6 to 768.5)
		Rate	0 to 9	59.3 (45.6 to 75.6)	63.1 (44.9 to 89.5)	55.7 (36.6 to 76.2)	44.7 (33 to 59.4)	48.7 (34.4 to 66.3)	41 (29.5 to 54.9)	-24.6 (-46.3 to 4.4)	-22.8 (-49.7 to 13.4)	-26.5 (-50.5 to 12.3)
			10 to 24	131.8 (107.7 to 158.2)	165.1 (129.4 to 208.8)	102.2 (79.2 to 128.4)	112.9 (86.9 to 144.8)	135.1 (99.7 to 177.6)	93 (68.3 to 124.8)	-14.3 (-32.5 to 7.3)	-18.1 (-39.2 to 7.2)	-9 (-34.6 to 25.3)
			25 to 49	320.6 (269.1 to 377.5)	357.7 (288 to 442.7)	287.5 (233.7 to 356.3)	313.5 (258.3 to 387)	317.6 (247 to 404.4)	310.2 (245.2 to 393.1)	-2.2 (-19.6 to 20.3)	-11.2 (-33.6 to 14.8)	7.9 (-18.1 to 44.6)
			50 to 74	1140.7 (960.3 to 1346.7)	1335.2 (1063.9 to 1655.5)	967.8 (763.4 to 1204)	1024.9 (860.9 to 1221.9)	1072.2 (859.8 to 1339)	983.6 (764.4 to 1241.8)	-10.2 (-29 to 14)	-19.7 (-42.4 to 8.3)	1.6 (-27.4 to 42.7)
			75 plus	4771.5 (3933.5 to 5761.3)	5435.5 (4270.2 to 6913.5)	4047 (3240.6 to 4987.3)	4753.4 (3956.1 to 5667.6)	5162 (4120.9 to 6350.5)	4375.7 (3373.5 to 5490.4)	-0.4 (-22 to 25.9)	-5 (-32.8 to 28.7)	8.1 (-21.8 to 49.4)
Kuwait	Deaths	Number	0 to 9	4 (3 to 5)	2 (2 to 3)	2 (1 to 3)	2 (2 to 3)	1 (1 to 2)	1 (1 to 1)	-49.8 (-64.3 to -30.5)	-42.9 (-60.2 to -18.4)	-57.6 (-71.5 to -37.4)
			10 to 24	5 (4 to 6)	2 (2 to 3)	3 (2 to 3)	3 (2 to 4)	2 (1 to 2)	2 (1 to 2)	-35.7 (-51.1 to -14.6)	-31.9 (-50.5 to -7.3)	-39.1 (-55.9 to -17.8)
			25 to 49	22 (19 to 25)	9 (8 to 11)	13 (11 to 15)	33 (26 to 41)	15 (11 to 21)	17 (14 to 22)	46.5 (12 to 89.2)	60.3 (14.3 to 120.8)	36.4 (2.2 to 82.1)
			50 to 74	66 (56 to 74)	29 (21 to 33)	37 (33 to 43)	131 (106 to 162)	58 (44 to 76)	73 (55 to 95)	98.4 (58.2 to 157.8)	102 (46.7 to 211.6)	95.7 (46.5 to 154.9)

Country	Measure	Metric	Age	Year						% Change (1990 to 2019)		
				1990			2019					
				Both	Female	Male	Both	Female	Male	Both	Female	Male
			75 plus	42 (35 to 48)	24 (18 to 28)	18 (15 to 21)	154 (119 to 188)	60 (43 to 78)	94 (73 to 117)	265.5 (199.6 to 337.7)	152.1 (96 to 233.8)	411.3 (313.5 to 533.7)
		Rate	0 to 9	1.1 (0.8 to 1.3)	1.1 (0.9 to 1.4)	1 (0.7 to 1.3)	0.4 (0.3 to 0.5)	0.4 (0.3 to 0.6)	0.3 (0.2 to 0.4)	-66.1 (-75.8 to -53)	-61 (-72.8 to -44.2)	-71.6 (-80.9 to -58)
			10 to 24	1 (0.8 to 1.3)	1 (0.8 to 1.2)	1.1 (0.9 to 1.4)	0.4 (0.3 to 0.5)	0.4 (0.3 to 0.5)	0.4 (0.3 to 0.5)	-62 (-71.1 to -49.6)	-59.6 (-70.6 to -45)	-64.2 (-74.1 to -51.7)
			25 to 49	3 (2.6 to 3.4)	3.5 (2.9 to 4)	2.7 (2.3 to 3.1)	1.4 (1.1 to 1.7)	1.4 (1 to 1.9)	1.4 (1.1 to 1.7)	-54.1 (-64.9 to -40.8)	-60.4 (-71.8 to -45.5)	-49.4 (-62.1 to -32.5)
			50 to 74	50.9 (43.5 to 57.2)	64.5 (47.9 to 75.7)	43.9 (38.6 to 50)	21.8 (17.6 to 27)	23.3 (17.8 to 30.8)	20.7 (15.7 to 26.8)	-57.3 (-66 to -44.5)	-63.8 (-73.7 to -44.2)	-52.9 (-64.8 to -38.7)
			75 plus	370.9 (307.1 to 425.1)	415.5 (314.5 to 494.9)	326 (269.7 to 380.4)	286.8 (221.1 to 351.5)	282.6 (201.8 to 370.4)	289.5 (223.4 to 358.9)	-22.7 (-36.6 to -7.4)	-32 (-47.1 to -9.9)	-11.2 (-28.2 to 10.1)
	DALYs	Number	0 to 9	448 (356 to 553)	241 (187 to 298)	207 (159 to 260)	347 (249 to 488)	198 (140 to 277)	149 (102 to 210)	-22.5 (-43.4 to 1.9)	-17.6 (-40.6 to 8.9)	-28.1 (-51.3 to 0.1)
			10 to 24	539 (438 to 660)	290 (232 to 361)	249 (198 to 318)	638 (449 to 872)	361 (246 to 508)	277 (190 to 384)	18.3 (-8.7 to 47.7)	24.4 (-5.4 to 59.1)	11.2 (-19.9 to 45.7)
			25 to 49	1672 (1420 to 1963)	774 (646 to 921)	898 (747 to 1073)	4081 (3037 to 5330)	2152 (1566 to 2855)	1929 (1418 to 2542)	144.1 (98.9 to 194.7)	177.9 (120 to 240.7)	114.9 (69.4 to 168.9)
			50 to 74	2023 (1753 to 2252)	867 (678 to 1009)	1156 (1016 to 1307)	4942 (4052 to 6024)	2175 (1721 to 2744)	2767 (2181 to 3500)	144.2 (102.9 to 200.8)	150.7 (95.2 to 256.7)	139.4 (90.9 to 196.6)
			75 plus	543 (459 to 620)	299 (236 to 355)	244 (207 to 286)	2043 (1657 to 2447)	779 (599 to 978)	1263 (1022 to 1537)	275.9 (214.9 to 349.4)	160.5 (109.8 to 234.6)	417.3 (325.2 to 525.9)
		Rate	0 to 9	52 (41.3 to 64.3)	57.2 (44.4 to 70.7)	47 (36.1 to 59.2)	27.8 (19.9 to 39.1)	32.7 (23.1 to 45.7)	23.1 (15.9 to 32.5)	-46.6 (-61 to -29.8)	-42.7 (-58.7 to -24.3)	-51 (-66.8 to -31.7)

Country	Measure	Metric	Age	Year						% Change (1990 to 2019)		
				1990			2019					
				Both	Female	Male	Both	Female	Male	Both	Female	Male
			10 to 24	113.7 (92.4 to 139.3)	125.4 (100.3 to 156.1)	102.6 (81.6 to 131.1)	79.5 (56 to 108.7)	92.6 (63.2 to 130.5)	67.1 (46.1 to 92.9)	-30.1 (-46.1 to -12.7)	-26.1 (-43.8 to -5.6)	-34.6 (-52.9 to -14.3)
			25 to 49	224.7 (190.9 to 263.9)	282.5 (235.5 to 335.8)	191 (158.9 to 228.3)	171.6 (127.7 to 224.1)	193.8 (141 to 257)	152.2 (111.9 to 200.6)	-23.6 (-37.8 to -7.8)	-31.4 (-45.7 to -15.9)	-20.3 (-37.2 to -0.3)
			50 to 74	810.7 (702.5 to 902.4)	1021 (798 to 1187.7)	702.2 (617.2 to 794.2)	425.8 (349.1 to 519)	453.3 (358.8 to 571.9)	406.4 (320.2 to 513.9)	-47.5 (-56.4 to -35.3)	-55.6 (-65.4 to -36.8)	-42.1 (-53.8 to -28.3)
			75 plus	3161.7 (2673.3 to 3604.9)	3360.9 (2653.3 to 3986.6)	2947.8 (2497.4 to 3451.3)	2379.7 (1930.6 to 2851)	2315.1 (1779.3 to 2904.2)	2421.5 (1958.4 to 2946.2)	-24.7 (-37 to -10)	-31.1 (-44.5 to -11.5)	-17.9 (-32.5 to -0.6)
Lebanon	Deaths	Number	0 to 9	10 (6 to 14)	5 (3 to 8)	5 (3 to 7)	5 (3 to 8)	2 (1 to 4)	3 (1 to 5)	-49.8 (-71.9 to -11.7)	-54.5 (-79.9 to -10.1)	-44.6 (-72.2 to 13.9)
			10 to 24	11 (8 to 14)	6 (4 to 8)	5 (3 to 7)	8 (6 to 11)	4 (3 to 6)	4 (3 to 6)	-24.3 (-49.3 to 15.2)	-28.8 (-58 to 8.4)	-19.1 (-51.8 to 42)
			25 to 49	46 (36 to 58)	26 (19 to 34)	20 (14 to 27)	58 (39 to 80)	32 (20 to 44)	27 (16 to 44)	26.4 (-17 to 84.5)	22.5 (-25 to 93)	31.4 (-17.5 to 114)
			50 to 74	311 (250 to 394)	154 (118 to 206)	158 (121 to 203)	430 (299 to 581)	231 (154 to 300)	199 (117 to 335)	38.3 (-8.4 to 88.7)	50.6 (-6.4 to 114.6)	26.3 (-25.1 to 108.4)
			75 plus	243 (199 to 306)	131 (100 to 184)	113 (90 to 143)	678 (481 to 888)	378 (252 to 476)	301 (178 to 477)	178.8 (90.8 to 272.3)	188.6 (90.2 to 287)	167.3 (51.1 to 336.4)
		Rate	0 to 9	1.1 (0.7 to 1.5)	1.2 (0.7 to 1.9)	1 (0.6 to 1.5)	0.5 (0.3 to 0.8)	0.5 (0.3 to 0.8)	0.5 (0.3 to 0.9)	-54.2 (-74.3 to -19.5)	-58.6 (-81.7 to -18)	-49.3 (-74.6 to 4.1)
			10 to 24	1.1 (0.8 to 1.5)	1.3 (0.9 to 1.8)	1 (0.7 to 1.4)	0.7 (0.5 to 1)	0.8 (0.5 to 1.1)	0.7 (0.4 to 1.1)	-35.6 (-56.9 to -2.1)	-39 (-64 to -7.1)	-31.8 (-59.3 to 19.8)
			25 to 49	5.1 (3.9 to 6.3)	5.4 (3.9 to 7.2)	4.7 (3.3 to 6.3)	3 (2 to 4)	3.1 (1.9 to 4.3)	2.8 (1.7 to 4.6)	-41.5 (-61.6 to -14.7)	-42.4 (-64.7 to -9.3)	-40.3 (-62.5 to -2.8)

Country	Measure	Metric	Age	Year						% Change (1990 to 2019)		
				1990			2019					
				Both	Female	Male	Both	Female	Male	Both	Female	Male
			50 to 74	65.6 (52.8 to 83.1)	65.7 (50.3 to 88.2)	65.5 (50.5 to 84.3)	46.7 (32.4 to 63)	45.8 (30.6 to 59.4)	47.7 (28 to 80.4)	-28.9 (-52.9 to -3)	-30.3 (-56.7 to -0.7)	-27.2 (-56.8 to 20.2)
			75 plus	432.1 (354.2 to 543.6)	428.5 (328 to 601.2)	436.4 (349.1 to 553.1)	345.1 (244.4 to 451.7)	345.2 (230.1 to 435.5)	344.9 (204.2 to 547.2)	-20.2 (-45.4 to 6.7)	-19.5 (-46.9 to 8)	-21 (-55.3 to 29)
	DALYs	Number	0 to 9	1057 (741 to 1430)	564 (359 to 840)	493 (337 to 687)	741 (485 to 1084)	373 (231 to 561)	368 (226 to 578)	-29.9 (-55 to 7.9)	-33.9 (-61.6 to 9.6)	-25.4 (-54.9 to 26.3)
			10 to 24	1114 (863 to 1409)	628 (470 to 822)	487 (363 to 642)	1147 (823 to 1513)	620 (429 to 842)	527 (367 to 761)	2.9 (-23.4 to 32.7)	-1.3 (-29.6 to 29.6)	8.3 (-25.6 to 55.5)
			25 to 49	2934 (2333 to 3583)	1719 (1319 to 2150)	1215 (915 to 1560)	4977 (3792 to 6399)	2829 (2089 to 3627)	2147 (1522 to 3067)	69.6 (29 to 121.3)	64.6 (19.2 to 124.7)	76.8 (26.8 to 143.4)
			50 to 74	8992 (7447 to 11112)	4519 (3572 to 5883)	4472 (3523 to 5655)	13663 (10313 to 17590)	7354 (5459 to 9230)	6309 (4221 to 9506)	51.9 (8.4 to 97.4)	62.7 (10.8 to 119.8)	41.1 (-6.8 to 111.8)
			75 plus	3200 (2665 to 3914)	1744 (1370 to 2340)	1455 (1155 to 1822)	9394 (7200 to 12002)	5165 (3755 to 6344)	4229 (2835 to 6331)	193.6 (115.5 to 279.1)	196.1 (108.5 to 282.3)	190.5 (85.8 to 343.4)
		Rate	0 to 9	53.5 (37.5 to 72.4)	59 (37.6 to 88)	48.3 (33 to 67.3)	35.4 (23.1 to 51.7)	36.8 (22.8 to 55.4)	34 (20.9 to 53.4)	-33.9 (-57.6 to 1.6)	-37.7 (-63.8 to 3.2)	-29.6 (-57.5 to 19.1)
			10 to 24	118.8 (91.9 to 150.2)	140.8 (105.5 to 184.3)	98.9 (73.8 to 130.4)	103.9 (74.6 to 137.1)	119.1 (82.4 to 161.9)	90.4 (63 to 130.4)	-12.5 (-34.9 to 12.8)	-15.4 (-39.7 to 11.1)	-8.6 (-37.2 to 31.2)
			25 to 49	320.9 (255.2 to 392)	357.3 (274.1 to 447)	280.5 (211.3 to 360.2)	251.7 (191.8 to 323.7)	276.4 (204 to 354.4)	225.2 (159.7 to 321.7)	-21.6 (-40.4 to 2.4)	-22.6 (-44 to 5.6)	-19.7 (-42.4 to 10.6)
			50 to 74	995.2 (824.2 to 1229.9)	1017.5 (804.2 to 1324.6)	973.7 (767 to 1231.2)	794.2 (599.5 to 1022.5)	780.3 (579.3 to 979.5)	811.1 (542.6 to 1222)	-20.2 (-43 to 3.7)	-23.3 (-47.8 to 3.6)	-16.7 (-44.9 to 25.1)
			75 plus	3798.5 (3163.3 to 4646.6)	3795 (2980.2 to 5091.2)	3802.8 (3018.2 to 4761.2)	3107.9 (2382.2 to 3970.8)	3053.9 (2220 to 3751)	3176.4 (2129.4 to 4755.8)	-18.2 (-39.9 to 5.7)	-19.5 (-43.3 to 3.9)	-16.5 (-46.6 to 27.5)

Country	Measure	Metric	Age	Year						% Change (1990 to 2019)		
				1990			2019					
				Both	Female	Male	Both	Female	Male	Both	Female	Male
Libya	Deaths	Number	0 to 9	22 (14 to 32)	12 (7 to 19)	10 (5 to 16)	7 (5 to 11)	4 (2 to 6)	4 (2 to 6)	-67.2 (-80.1 to -39.4)	-68.4 (-83.5 to -37.6)	-65.7 (-82.1 to -13.8)
			10 to 24	16 (12 to 20)	9 (6 to 13)	7 (5 to 10)	16 (11 to 23)	9 (5 to 14)	7 (4 to 11)	-0.7 (-31 to 49.1)	-0.9 (-39.6 to 59.3)	-0.5 (-41 to 82.6)
			25 to 49	50 (37 to 64)	25 (17 to 33)	25 (18 to 34)	156 (103 to 218)	85 (49 to 125)	71 (47 to 118)	212.3 (111.8 to 351.3)	243.7 (118.2 to 422.6)	181.5 (77 to 342.8)
			50 to 74	231 (168 to 296)	122 (86 to 166)	110 (76 to 153)	599 (399 to 827)	341 (203 to 473)	258 (169 to 410)	159.1 (79.2 to 260.4)	180.6 (73.7 to 300.3)	135.2 (53.9 to 260.7)
			75 plus	203 (146 to 289)	101 (68 to 165)	102 (73 to 144)	596 (392 to 847)	315 (189 to 496)	281 (187 to 444)	194.1 (115.4 to 288.6)	212.9 (115.2 to 321.6)	175.6 (88.4 to 309.7)
		Rate	0 to 9	1.7 (1 to 2.4)	1.8 (1 to 2.8)	1.6 (0.8 to 2.4)	0.8 (0.5 to 1.2)	0.9 (0.5 to 1.3)	0.7 (0.5 to 1.3)	-52.5 (-71.3 to -12.5)	-52.5 (-75.2 to -6.3)	-52.3 (-75.1 to 19.9)
			10 to 24	1.1 (0.8 to 1.4)	1.3 (0.9 to 1.8)	0.9 (0.7 to 1.3)	0.9 (0.6 to 1.3)	1.1 (0.6 to 1.7)	0.8 (0.5 to 1.3)	-18 (-43 to 23.2)	-17.9 (-50 to 31.9)	-18 (-51.3 to 50.6)
			25 to 49	4.7 (3.5 to 6)	5.3 (3.7 to 7.1)	4.2 (3 to 5.7)	5.3 (3.5 to 7.4)	6.1 (3.5 to 8.9)	4.6 (3 to 7.7)	14 (-22.7 to 64.7)	14 (-27.6 to 73.3)	11.3 (-30 to 75.1)
			50 to 74	62.8 (45.7 to 80.5)	72.5 (51.1 to 99)	54.6 (38 to 76.2)	58.9 (39.2 to 81.3)	68.2 (40.6 to 94.6)	49.8 (32.7 to 79.3)	-6.2 (-35.1 to 30.5)	-5.9 (-41.8 to 34.2)	-8.8 (-40.3 to 39.9)
			75 plus	399.4 (288 to 570.1)	393.9 (265.7 to 647.3)	405.1 (288 to 570.6)	420.6 (276.7 to 598)	454 (272.3 to 715.2)	388.6 (257.9 to 614.3)	5.3 (-22.9 to 39.1)	15.3 (-20.7 to 55.3)	-4.1 (-34.4 to 42.6)
	DALYs	Number	0 to 9	2228 (1476 to 3069)	1211 (743 to 1788)	1017 (554 to 1524)	924 (636 to 1270)	483 (299 to 691)	442 (293 to 663)	-58.5 (-72.6 to -32.3)	-60.1 (-76.4 to -31.3)	-56.5 (-73.8 to -9.2)
			10 to 24	1664 (1312 to 2052)	989 (737 to 1257)	675 (497 to 897)	1978 (1405 to 2601)	1141 (768 to 1544)	837 (573 to 1213)	18.8 (-8.4 to 56.2)	15.3 (-15.2 to 55.5)	23.9 (-13.5 to 84.2)

Country	Measure	Metric	Age	Year						% Change (1990 to 2019)		
				1990			2019					
				Both	Female	Male	Both	Female	Male	Both	Female	Male
			25 to 49	3295 (2608 to 4077)	1707 (1307 to 2160)	1589 (1200 to 2045)	10831 (7899 to 14305)	6045 (4163 to 8181)	4785 (3395 to 7167)	228.7 (150.9 to 332.1)	254.2 (157.8 to 375.8)	201.2 (111.2 to 327.4)
			50 to 74	6787 (5241 to 8509)	3586 (2666 to 4680)	3201 (2354 to 4328)	18902 (13359 to 24873)	10667 (6955 to 14193)	8235 (5745 to 12291)	178.5 (104.9 to 268.9)	197.5 (101.1 to 310.2)	157.2 (78.5 to 276.4)
			75 plus	2567 (1936 to 3524)	1311 (938 to 2023)	1256 (915 to 1745)	7943 (5689 to 10828)	4108 (2688 to 5910)	3835 (2729 to 5824)	209.4 (135.8 to 297.6)	213.4 (124.2 to 313.8)	205.3 (113.1 to 333.2)
		Rate	0 to 9	77.8 (51.6 to 107.2)	84.2 (51.6 to 124.3)	71.4 (38.9 to 107.1)	50.2 (34.6 to 69)	53.9 (33.4 to 77.2)	46.7 (30.9 to 70.2)	-35.5 (-57.4 to 5.3)	-35.9 (-62.1 to 10.4)	-34.6 (-60.6 to 36.8)
			10 to 24	116.4 (91.8 to 143.5)	141.6 (105.4 to 180)	92.3 (68 to 122.6)	114.3 (81.2 to 150.3)	135.3 (91 to 183)	94.3 (64.6 to 136.8)	-1.8 (-24.3 to 29.1)	-4.5 (-29.8 to 28.7)	2.2 (-28.7 to 51.9)
			25 to 49	307.7 (243.5 to 380.7)	367.6 (281.5 to 465.1)	261.9 (197.8 to 337.2)	369.1 (269.2 to 487.5)	431.6 (297.2 to 584.1)	312 (221.4 to 467.3)	20 (-8.4 to 57.7)	17.4 (-14.5 to 57.7)	19.1 (-16.5 to 69)
			50 to 74	967.3 (746.9 to 1212.7)	1127.2 (838.1 to 1471.1)	834.7 (613.8 to 1128.4)	972.4 (687.3 to 1279.6)	1116.6 (728 to 1485.8)	833.1 (581.2 to 1243.4)	0.5 (-26 to 33.1)	-0.9 (-33 to 36.6)	-0.2 (-30.8 to 46.1)
			75 plus	3274.4 (2469.4 to 4495.6)	3324.6 (2378.2 to 5129.4)	3223.6 (2348.9 to 4477.4)	3612.8 (2587.7 to 4924.9)	3779.7 (2472.9 to 5437.3)	3449.6 (2455 to 5239.4)	10.3 (-15.9 to 41.8)	13.7 (-18.7 to 50.1)	7 (-25.3 to 51.9)
Morocco	Deaths	Number	0 to 9	163 (71 to 241)	87 (34 to 135)	76 (27 to 125)	64 (35 to 109)	30 (14 to 53)	34 (17 to 62)	-60.5 (-79 to -11.6)	-65.2 (-85.9 to -11.5)	-55.2 (-80.4 to 36.5)
			10 to 24	99 (71 to 131)	60 (38 to 84)	39 (28 to 55)	91 (64 to 131)	54 (33 to 83)	38 (23 to 60)	-7.8 (-38.7 to 39.3)	-10.4 (-49.7 to 47.2)	-3.9 (-41.8 to 65.2)
			25 to 49	333 (268 to 410)	178 (136 to 236)	155 (117 to 202)	624 (417 to 924)	387 (243 to 573)	237 (150 to 385)	87.2 (22.8 to 182.1)	116.9 (27.2 to 246)	53 (-1.9 to 152.6)
			50 to 74	1766 (1456 to 2255)	881 (678 to 1248)	884 (672 to 1197)	4634 (3346 to 5973)	2466 (1737 to 3266)	2168 (1450 to 3036)	162.5 (83.3 to 245.3)	179.7 (79.3 to 300.7)	145.3 (68.3 to 247.4)

Country	Measure	Metric	Age	Year						% Change (1990 to 2019)		
				1990			2019					
				Both	Female	Male	Both	Female	Male	Both	Female	Male
			75 plus	1190 (942 to 1820)	564 (421 to 1038)	626 (451 to 1004)	3906 (3072 to 5392)	1979 (1430 to 2955)	1927 (1360 to 2906)	228.3 (144 to 322.8)	251.1 (128 to 396.8)	207.8 (117.5 to 334.8)
		Rate	0 to 9	2.4 (1 to 3.6)	2.6 (1 to 4)	2.2 (0.8 to 3.6)	1 (0.6 to 1.8)	1 (0.5 to 1.7)	1.1 (0.5 to 1.9)	-56.9 (-77.1 to -3.6)	-61.6 (-84.5 to -2.3)	-51.6 (-78.8 to 47.3)
			10 to 24	1.2 (0.9 to 1.6)	1.5 (0.9 to 2.1)	1 (0.7 to 1.3)	1 (0.7 to 1.4)	1.2 (0.7 to 1.8)	0.8 (0.5 to 1.3)	-19.7 (-46.6 to 21.4)	-20.3 (-55.3 to 31.1)	-17.9 (-50.3 to 41.1)
			25 to 49	4.5 (3.6 to 5.6)	4.8 (3.7 to 6.3)	4.3 (3.2 to 5.6)	4.8 (3.2 to 7.1)	5.9 (3.7 to 8.7)	3.6 (2.3 to 5.9)	5.1 (-31 to 58.4)	22.8 (-27.9 to 96)	-14.8 (-45.3 to 40.7)
			50 to 74	64.9 (53.5 to 82.9)	64.3 (49.5 to 91.1)	65.4 (49.7 to 88.6)	70.8 (51.1 to 91.2)	75.7 (53.3 to 100.3)	65.9 (44.1 to 92.2)	9.1 (-23.8 to 43.5)	17.6 (-24.6 to 68.5)	0.7 (-30.9 to 42.6)
			75 plus	361.1 (285.9 to 552.3)	326.4 (243.9 to 600.8)	399.3 (287.9 to 640.7)	488.7 (384.3 to 674.6)	461 (333.1 to 688.2)	520.8 (367.5 to 785.4)	35.4 (0.6 to 74.3)	41.3 (-8.3 to 99.9)	30.4 (-7.8 to 84.2)
	DALYs	Number	0 to 9	15700 (7638 to 22518)	8461 (3867 to 12549)	7239 (2988 to 11560)	8176 (5148 to 12571)	4031 (2323 to 6286)	4144 (2535 to 6637)	-47.9 (-68.6 to 1.4)	-52.4 (-75.8 to 0.1)	-42.7 (-70.3 to 42.6)
			10 to 24	9745 (7648 to 12156)	6061 (4444 to 7934)	3684 (2787 to 4843)	12167 (8841 to 16111)	7276 (5185 to 9842)	4891 (3257 to 6920)	24.9 (-8.4 to 65.7)	20 (-18 to 65.1)	32.8 (-8.5 to 95.7)
			25 to 49	21361 (17683 to 25440)	12117 (9621 to 14972)	9244 (7290 to 11628)	45731 (34002 to 62284)	28464 (20860 to 38840)	17266 (12301 to 25436)	114.1 (58.8 to 189)	134.9 (64.3 to 225)	86.8 (34.9 to 167.2)
			50 to 74	50095 (42207 to 62515)	25687 (20567 to 35524)	24408 (19060 to 31973)	141532 (107022 to 176848)	75634 (55923 to 98295)	65897 (46157 to 88552)	182.5 (103.7 to 255.7)	194.4 (98 to 304.6)	170 (91.2 to 267.5)
			75 plus	16090 (12902 to 23462)	7759 (5913 to 13036)	8331 (6018 to 12837)	52723 (42415 to 68973)	27245 (20809 to 37775)	25478 (18435 to 36152)	227.7 (147.3 to 317.3)	251.1 (138.4 to 384.8)	205.8 (121.3 to 325.8)
		Rate	0 to 9	107.1 (52.1 to 153.6)	117.1 (53.5 to 173.7)	97.3 (40.2 to 155.4)	63.3 (39.9 to 97.3)	64 (36.9 to 99.7)	62.7 (38.3 to 100.4)	-40.9 (-64.3 to 15.1)	-45.4 (-72.3 to 14.7)	-35.6 (-66.6 to 60.4)

Country	Measure	Metric	Age	Year						% Change (1990 to 2019)		
				1990			2019					
				Both	Female	Male	Both	Female	Male	Both	Female	Male
			10 to 24	120.2 (94.3 to 149.9)	148.9 (109.2 to 195)	91.2 (69 to 119.9)	130.8 (95.1 to 173.2)	159.1 (113.4 to 215.3)	103.4 (68.9 to 146.4)	8.8 (-20.1 to 44.4)	6.9 (-27 to 47)	13.4 (-21.9 to 67.1)
			25 to 49	290.8 (240.7 to 346.3)	325.1 (258.1 to 401.7)	255.4 (201.4 to 321.3)	349.7 (260 to 476.2)	432.6 (317 to 590.2)	265.7 (189.3 to 391.4)	20.3 (-10.8 to 62.3)	33.1 (-6.9 to 84.1)	4 (-24.9 to 48.8)
			50 to 74	972.9 (819.7 to 1214.1)	988.4 (791.4 to 1366.9)	957 (747.4 to 1253.7)	1131.2 (855.4 to 1413.5)	1216 (899.1 to 1580.3)	1047.5 (733.7 to 1407.6)	16.3 (-16.2 to 46.4)	23 (-17.3 to 69.1)	9.4 (-22.5 to 49)
			75 plus	3359.7 (2693.9 to 4898.8)	3026 (2305.9 to 5083.7)	3744.2 (2704.6 to 5769.2)	4382.4 (3525.6 to 5733.1)	4222 (3224.7 to 5853.8)	4567.9 (3305.2 to 6481.6)	30.4 (-1.6 to 66.1)	39.5 (-5.3 to 92.6)	22 (-11.7 to 69.9)
Oman	Deaths	Number	0 to 9	2 (1 to 3)	1 (1 to 2)	1 (0 to 1)	1 (1 to 2)	1 (1 to 1)	0 (0 to 1)	-33.3 (-57.4 to 20.1)	-38.3 (-65.3 to 23.7)	-21.1 (-56.2 to 76.7)
			10 to 24	2 (1 to 2)	1 (1 to 2)	1 (0 to 1)	3 (3 to 5)	2 (2 to 3)	1 (1 to 2)	97.8 (34.9 to 193.1)	94.9 (23.1 to 210.5)	102.4 (16.6 to 255.4)
			25 to 49	8 (6 to 12)	3 (2 to 5)	5 (3 to 7)	22 (16 to 32)	9 (7 to 12)	13 (9 to 22)	157.8 (72.8 to 286.2)	152.8 (59.4 to 296.3)	161.3 (56.9 to 345.5)
			50 to 74	46 (34 to 63)	21 (16 to 30)	24 (17 to 37)	97 (79 to 121)	49 (37 to 61)	49 (35 to 68)	111.6 (54 to 194.2)	126.2 (54.5 to 236.5)	98.7 (27.1 to 214.4)
			75 plus	24 (18 to 32)	13 (10 to 18)	11 (8 to 16)	75 (60 to 90)	35 (27 to 44)	40 (28 to 51)	210.6 (101.6 to 334.9)	166.6 (70.3 to 289.8)	264.3 (95.2 to 477.4)
		Rate	0 to 9	0.3 (0.2 to 0.5)	0.5 (0.3 to 0.8)	0.2 (0.1 to 0.3)	0.2 (0.1 to 0.2)	0.2 (0.2 to 0.3)	0.1 (0.1 to 0.2)	-46.5 (-65.8 to -3.6)	-50.3 (-72 to -0.3)	-36.9 (-65 to 41.2)
			10 to 24	0.3 (0.2 to 0.4)	0.4 (0.3 to 0.6)	0.2 (0.2 to 0.3)	0.4 (0.3 to 0.5)	0.5 (0.4 to 0.7)	0.3 (0.2 to 0.4)	14.5 (-22 to 69.6)	17.7 (-25.7 to 87.5)	13.3 (-34.7 to 99)
			25 to 49	1.3 (0.9 to 1.8)	1.7 (1.2 to 2.4)	1.1 (0.8 to 1.7)	0.9 (0.7 to 1.3)	1.3 (0.9 to 1.7)	0.7 (0.5 to 1.3)	-32.5 (-54.7 to 1.1)	-25.3 (-52.9 to 17.1)	-35 (-61 to 10.8)

Country	Measure	Metric	Age	Year						% Change (1990 to 2019)		
				1990			2019					
				Both	Female	Male	Both	Female	Male	Both	Female	Male
			50 to 74	33 (24.6 to 45.1)	36.5 (26.6 to 50.3)	30.5 (21.1 to 45.5)	24.1 (19.5 to 30)	31.7 (24.1 to 39.7)	19.4 (14.2 to 27.1)	-27 (-46.9 to 1.5)	-13.3 (-40.8 to 29)	-36.2 (-59.2 to 1)
			75 plus	215.2 (163.2 to 286.6)	200 (148.9 to 278.7)	237.1 (170.4 to 338.6)	302.2 (243.7 to 361.4)	289.4 (218.3 to 358.3)	314.6 (221.9 to 402.9)	40.4 (-8.9 to 96.6)	44.7 (-7.6 to 111.6)	32.7 (-28.9 to 110.3)
	DALYs	Number	0 to 9	308 (214 to 419)	198 (133 to 288)	110 (73 to 155)	360 (251 to 530)	206 (137 to 307)	154 (103 to 232)	17.2 (-18.5 to 63.8)	4.4 (-33.9 to 62)	40.2 (-3.8 to 103)
			10 to 24	287 (211 to 380)	169 (123 to 223)	118 (84 to 159)	720 (501 to 999)	388 (275 to 517)	332 (209 to 511)	150.6 (94 to 209)	128.7 (71.4 to 197.3)	182 (103.3 to 272.9)
			25 to 49	775 (598 to 988)	336 (253 to 434)	439 (328 to 572)	3406 (2438 to 4586)	1314 (977 to 1749)	2092 (1423 to 2935)	339.6 (242.2 to 446.7)	291.4 (192.6 to 400.1)	376.4 (255.8 to 522.1)
			50 to 74	1461 (1138 to 1905)	697 (543 to 895)	765 (559 to 1068)	3833 (3212 to 4645)	1836 (1492 to 2197)	1998 (1567 to 2610)	162.3 (104.9 to 237.4)	163.5 (99.8 to 248.1)	161.3 (82 to 266.7)
			75 plus	378 (298 to 478)	217 (170 to 285)	161 (117 to 227)	1225 (1010 to 1444)	602 (481 to 734)	624 (463 to 788)	223.8 (129.1 to 325.9)	176.6 (93.5 to 271.5)	287.7 (130.1 to 478.7)
		Rate	0 to 9	23.8 (16.6 to 32.4)	31.1 (20.9 to 45.4)	16.8 (11.1 to 23.7)	22.4 (15.6 to 33)	26.2 (17.4 to 38.9)	18.8 (12.5 to 28.4)	-5.8 (-34.5 to 31.7)	-15.8 (-46.7 to 30.7)	12.3 (-22.9 to 62.6)
			10 to 24	52.3 (38.5 to 69.2)	69.5 (50.2 to 91.4)	38.6 (27.7 to 52.3)	75.9 (52.8 to 105.3)	95.9 (68 to 128)	61 (38.4 to 93.7)	45 (12.2 to 78.8)	38.1 (3.5 to 79.5)	57.9 (13.8 to 108.8)
			25 to 49	120.3 (92.8 to 153.3)	164 (123.6 to 212.1)	99.9 (74.6 to 130.1)	138.5 (99.1 to 186.5)	189.6 (140.9 to 252.4)	118.4 (80.6 to 166.2)	15.1 (-10.4 to 43.2)	15.6 (-13.6 to 47.7)	18.5 (-11.5 to 54.8)
			50 to 74	548 (426.6 to 714.4)	623.8 (486.2 to 800.8)	493.4 (360.6 to 689.4)	492.5 (412.6 to 596.7)	626.3 (508.9 to 749.6)	411.6 (322.8 to 537.8)	-10.1 (-29.8 to 15.6)	0.4 (-23.9 to 32.7)	-16.6 (-41.9 to 17.1)
			75 plus	2371 (1866.6 to 2997.5)	2249.1 (1760.2 to 2945.9)	2558.5 (1866.1 to 3604.4)	3552.9 (2929.1 to 4189)	3457.8 (2765.9 to 4219.2)	3649.8 (2709.1 to 4614.8)	49.9 (6 to 97.1)	53.7 (7.6 to 106.5)	42.7 (-15.3 to 112.9)

Country	Measure	Metric	Age	Year						% Change (1990 to 2019)		
				1990			2019					
				Both	Female	Male	Both	Female	Male	Both	Female	Male
Palestine	Deaths	Number	0 to 9	24 (14 to 33)	14 (7 to 21)	10 (5 to 15)	11 (8 to 16)	5 (4 to 7)	6 (4 to 9)	-52 (-70.8 to -13.1)	-63.3 (-79.2 to -29.9)	-36.2 (-63.8 to 45)
			10 to 24	12 (9 to 17)	7 (5 to 10)	5 (3 to 8)	17 (14 to 21)	9 (7 to 12)	8 (6 to 11)	40.5 (1.5 to 98.1)	30.9 (-12.8 to 94.7)	53.8 (-0.4 to 156.6)
			25 to 49	29 (21 to 40)	15 (10 to 21)	14 (10 to 20)	63 (52 to 75)	28 (22 to 34)	35 (28 to 44)	114 (51.2 to 202.8)	81.6 (22 to 172.7)	148.6 (74.7 to 261.7)
			50 to 74	182 (137 to 231)	96 (73 to 124)	85 (62 to 113)	322 (265 to 380)	155 (127 to 185)	167 (134 to 203)	77.2 (30.6 to 140.5)	60.9 (15.1 to 125.5)	95.7 (35.4 to 182.8)
			75 plus	141 (115 to 173)	70 (54 to 89)	70 (56 to 90)	235 (199 to 272)	129 (104 to 153)	106 (86 to 126)	66.7 (27.3 to 113)	82.5 (36.2 to 147.8)	51 (8.8 to 98.8)
		Rate	0 to 9	3.2 (1.9 to 4.5)	3.9 (2.1 to 5.9)	2.6 (1.3 to 4)	0.9 (0.7 to 1.2)	0.8 (0.6 to 1.2)	1 (0.7 to 1.4)	-72.1 (-83.1 to -49.6)	-78.7 (-88 to -59.4)	-62.9 (-79 to -15.7)
			10 to 24	1.8 (1.3 to 2.5)	2.2 (1.6 to 3)	1.5 (0.9 to 2.2)	1.1 (0.9 to 1.3)	1.2 (0.9 to 1.5)	1 (0.7 to 1.3)	-40.9 (-57.4 to -16.7)	-45.3 (-63.6 to -18.7)	-35 (-57.9 to 8.5)
			25 to 49	6.3 (4.5 to 8.5)	6.4 (4.4 to 9)	6.1 (4.2 to 8.4)	4.2 (3.4 to 5)	3.7 (2.9 to 4.6)	4.6 (3.6 to 5.7)	-33.9 (-53.3 to -6.5)	-42.5 (-61.4 to -13.7)	-25.1 (-47.4 to 9)
			50 to 74	108.2 (81.6 to 137.8)	103.3 (78.1 to 133.2)	114.4 (83.5 to 151.8)	62.9 (51.8 to 74.3)	61.6 (50.4 to 73.7)	64.1 (51.5 to 77.8)	-41.9 (-57.2 to -21.2)	-40.3 (-57.3 to -16.4)	-44 (-61.3 to -19.1)
			75 plus	596.7 (486 to 733.7)	539.8 (416.3 to 679)	667.1 (532 to 856.5)	449.2 (381.2 to 519)	410.5 (330.9 to 488.3)	507.1 (408.3 to 599.9)	-24.7 (-42.5 to -3.8)	-24 (-43.3 to 3.3)	-24 (-45.2 to 0.1)
	DALYs	Number	0 to 9	2275 (1423 to 3115)	1316 (759 to 1963)	958 (527 to 1412)	1468 (1083 to 1960)	686 (493 to 944)	782 (559 to 1070)	-35.4 (-58.2 to 10.4)	-47.9 (-69.1 to -6.6)	-18.4 (-50.2 to 63.9)
			10 to 24	1202 (924 to 1533)	703 (536 to 914)	499 (346 to 676)	2205 (1739 to 2734)	1226 (943 to 1545)	979 (733 to 1253)	83.5 (40.6 to 132)	74.5 (29.3 to 130.3)	96.2 (37.8 to 182.2)

Country	Measure	Metric	Age	Year						% Change (1990 to 2019)		
				1990			2019					
				Both	Female	Male	Both	Female	Male	Both	Female	Male
			25 to 49	1899 (1463 to 2432)	1033 (767 to 1358)	865 (634 to 1131)	4867 (4032 to 5762)	2388 (1951 to 2896)	2480 (2018 to 2984)	156.3 (97.5 to 229.1)	131.1 (73.5 to 210.6)	186.5 (115.5 to 282.5)
			50 to 74	5042 (3902 to 6377)	2717 (2101 to 3421)	2325 (1726 to 3055)	10020 (8449 to 11689)	4784 (3994 to 5608)	5236 (4310 to 6298)	98.7 (51.7 to 162.1)	76.1 (31.7 to 134.5)	125.2 (63.5 to 218.9)
			75 plus	1807 (1485 to 2205)	911 (718 to 1144)	896 (707 to 1142)	3291 (2814 to 3851)	1811 (1504 to 2149)	1480 (1212 to 1765)	82.1 (43.3 to 128.6)	98.9 (52.9 to 164.7)	65.1 (21 to 116.5)
		Rate	0 to 9	137.9 (86.3 to 188.8)	164.2 (94.8 to 245)	112.9 (62.1 to 166.4)	55.8 (41.1 to 74.4)	53.6 (38.5 to 73.7)	57.8 (41.3 to 79.1)	-59.6 (-73.8 to -30.8)	-67.4 (-80.7 to -41.6)	-48.8 (-68.8 to 2.8)
			10 to 24	177.6 (136.6 to 226.6)	214.1 (163.3 to 278.4)	143.2 (99.4 to 194)	137 (108 to 169.8)	156.1 (120 to 196.6)	118.7 (88.9 to 152)	-22.9 (-40.9 to -2.5)	-27.1 (-46 to -3.8)	-17.1 (-41.8 to 19.3)
			25 to 49	404.8 (312 to 518.4)	436.3 (324 to 573.4)	372.7 (273.1 to 487.2)	320.4 (265.4 to 379.3)	319.1 (260.7 to 387)	321.7 (261.9 to 387.1)	-20.9 (-39 to 1.6)	-26.9 (-45.1 to -1.7)	-13.7 (-35.1 to 15.2)
			50 to 74	1589.2 (1229.9 to 2010.1)	1536.2 (1188 to 1934.4)	1655.9 (1229.5 to 2175.6)	1021.8 (861.6 to 1192)	996.7 (832.2 to 1168.3)	1045.9 (860.9 to 1258.1)	-35.7 (-50.9 to -15.2)	-35.1 (-51.5 to -13.6)	-36.8 (-54.1 to -10.6)
			75 plus	5078.3 (4172.2 to 6197.6)	4550.3 (3587.8 to 5717.3)	5757.1 (4543.8 to 7336.3)	4235.9 (3621.1 to 4956)	3827.4 (3177.5 to 4541.9)	4872.3 (3989.6 to 5811)	-16.6 (-34.4 to 4.7)	-15.9 (-35.3 to 12)	-15.4 (-38 to 11)
Qatar	Deaths	Number	0 to 9	1 (1 to 2)	1 (0 to 1)	1 (0 to 1)	1 (1 to 2)	1 (0 to 1)	1 (0 to 1)	1.4 (-35.7 to 66.2)	-6.7 (-48.4 to 74.3)	9.4 (-39.9 to 130.9)
			10 to 24	1 (0 to 1)	0 (0 to 0)	0 (0 to 0)	2 (1 to 2)	1 (0 to 1)	1 (1 to 2)	171.9 (83 to 301.6)	94.8 (24.6 to 193.6)	259.3 (118.8 to 494.9)
			25 to 49	3 (3 to 5)	1 (1 to 2)	2 (1 to 3)	16 (11 to 21)	4 (3 to 6)	12 (8 to 16)	357.5 (209.3 to 556.9)	214.9 (94.7 to 384.7)	440.8 (243.4 to 730.7)
			50 to 74	15 (12 to 23)	7 (5 to 12)	9 (6 to 12)	80 (58 to 106)	31 (23 to 41)	49 (33 to 68)	415.9 (239.7 to 652)	362.2 (164.8 to 599)	456.8 (266.5 to 757.9)

Country	Measure	Metric	Age	Year						% Change (1990 to 2019)		
				1990			2019					
				Both	Female	Male	Both	Female	Male	Both	Female	Male
			75 plus	8 (6 to 15)	5 (4 to 11)	3 (2 to 5)	34 (27 to 42)	15 (11 to 19)	19 (15 to 25)	305.6 (140.7 to 464.4)	176.8 (43.8 to 315.9)	528.1 (302.1 to 820.8)
		Rate	0 to 9	1.3 (0.9 to 1.8)	1.3 (0.8 to 2)	1.3 (0.8 to 1.9)	0.4 (0.3 to 0.6)	0.4 (0.2 to 0.6)	0.4 (0.3 to 0.7)	-68.5 (-80 to -48.5)	-71 (-84 to -45.8)	-66.1 (-81.4 to -28.5)
			10 to 24	0.6 (0.5 to 0.9)	0.8 (0.6 to 1.2)	0.5 (0.3 to 0.7)	0.3 (0.2 to 0.4)	0.4 (0.3 to 0.6)	0.3 (0.2 to 0.4)	-47.2 (-64.5 to -22)	-47.4 (-66.3 to -20.7)	-41.2 (-64.2 to -2.6)
			25 to 49	1.5 (1.1 to 2.1)	2.3 (1.7 to 3.2)	1.3 (0.9 to 2)	0.9 (0.6 to 1.2)	1.1 (0.8 to 1.5)	0.9 (0.6 to 1.2)	-41.3 (-60.3 to -15.7)	-53 (-71 to -27.7)	-33.6 (-57.8 to 2)
			50 to 74	56.2 (43.1 to 84.1)	88.7 (62.7 to 158)	43.9 (32.3 to 61.7)	27.6 (20.1 to 36.5)	49.4 (36.5 to 65.3)	21.5 (14.8 to 30)	-50.9 (-67.7 to -28.5)	-44.2 (-68.1 to -15.7)	-50.9 (-67.7 to -24.4)
			75 plus	740.4 (564.9 to 1314.8)	827.7 (588.6 to 1666.2)	626.3 (440.6 to 977.6)	657.1 (520.7 to 807.7)	927.6 (691.5 to 1202.6)	537.7 (404.6 to 704)	-11.2 (-47.3 to 23.5)	12.1 (-41.8 to 68.4)	-14.1 (-45 to 25.9)
	DALYs	Number	0 to 9	123 (88 to 164)	62 (42 to 90)	61 (39 to 88)	196 (136 to 279)	96 (63 to 145)	100 (66 to 143)	59.6 (6.2 to 141)	55.1 (-7.6 to 155.4)	64.2 (0.2 to 185.5)
			10 to 24	82 (63 to 109)	43 (32 to 59)	39 (27 to 54)	410 (270 to 586)	147 (99 to 203)	263 (158 to 402)	399.1 (268.2 to 543.7)	240 (146.7 to 336.6)	575.3 (356 to 842.2)
			25 to 49	324 (253 to 415)	120 (93 to 153)	205 (152 to 276)	2685 (1911 to 3647)	781 (564 to 1055)	1905 (1337 to 2626)	728.6 (559.5 to 930.5)	553.3 (391.8 to 718.7)	830.9 (594.2 to 1104.1)
			50 to 74	467 (374 to 663)	196 (145 to 326)	271 (206 to 363)	2936 (2280 to 3751)	980 (765 to 1244)	1956 (1466 to 2591)	528.5 (343.1 to 756.6)	399.8 (205.6 to 613.4)	621.6 (402.8 to 926.1)
			75 plus	110 (85 to 188)	68 (50 to 129)	42 (30 to 63)	501 (400 to 611)	214 (162 to 276)	286 (220 to 371)	355.1 (178.4 to 517.1)	214.5 (71.7 to 356.1)	583.9 (351.6 to 870)
		Rate	0 to 9	61.3 (44 to 81.9)	63.3 (42.8 to 92.5)	59.3 (38 to 85.8)	32.5 (22.7 to 46.4)	32.6 (21.5 to 49.2)	32.5 (21.5 to 46.5)	-46.9 (-64.7 to -19.8)	-48.5 (-69.3 to -15.1)	-45.3 (-66.6 to -4.9)

Country	Measure	Metric	Age	Year 1990 Both	1990 Female	1990 Male	2019 Both	2019 Female	2019 Male	% Change (1990 to 2019) Both	% Change Female	% Change Male
			10 to 24	83 (63.3 to 110.3)	109.4 (82.2 to 150.1)	65.5 (46.2 to 91.1)	80.4 (53 to 115)	100.4 (67.6 to 139)	72.4 (43.3 to 110.6)	-3.1 (-28.5 to 25)	-8.2 (-33.4 to 17.9)	10.5 (-25.4 to 54.2)
			25 to 49	142.7 (111.5 to 182.6)	213.9 (165.9 to 274)	119.5 (88.8 to 161.3)	151.8 (108 to 206.2)	208.5 (150.6 to 281.5)	136.6 (95.9 to 188.3)	6.4 (-15.3 to 32.3)	-2.5 (-26.6 to 22.1)	14.3 (-14.7 to 47.9)
			50 to 74	868.5 (695.2 to 1233.4)	1343.3 (993.8 to 2234.6)	691.7 (526.7 to 925.2)	516.5 (401 to 659.8)	800 (624.8 to 1016.2)	438.7 (328.6 to 580.9)	-40.5 (-58.1 to -18.9)	-40.4 (-63.6 to -15)	-36.6 (-55.8 to -9.8)
			75 plus	6667 (5180.1 to 11388.1)	6996 (5095.8 to 13212.5)	6192.7 (4423.7 to 9330)	7478.5 (5982.3 to 9126.3)	11040.9 (8367.3 to 14198.1)	6023.2 (4627.8 to 7816.9)	12.2 (-31.4 to 52.1)	57.8 (-13.8 to 128.9)	-2.7 (-35.8 to 37.9)
Saudi Arabia	Deaths	Number	0 to 9	65 (40 to 92)	30 (17 to 47)	35 (20 to 53)	13 (9 to 20)	6 (4 to 9)	7 (4 to 12)	-79.3 (-87.9 to -60.2)	-79.6 (-90.3 to -57)	-79.1 (-90 to -48.5)
			10 to 24	69 (48 to 92)	40 (26 to 57)	28 (19 to 41)	96 (71 to 128)	59 (39 to 83)	37 (26 to 52)	39.7 (-10.7 to 112.5)	45.4 (-17.9 to 138.4)	31.5 (-21.2 to 117.3)
			25 to 49	330 (231 to 451)	148 (102 to 208)	182 (121 to 255)	1400 (986 to 1933)	655 (447 to 927)	746 (495 to 1087)	323.8 (173.6 to 578.4)	341.4 (164.6 to 630.6)	309.4 (150.2 to 588)
			50 to 74	1248 (937 to 1663)	470 (358 to 622)	777 (559 to 1073)	3739 (2894 to 4649)	1570 (1163 to 2035)	2170 (1629 to 2838)	199.7 (99.2 to 328.9)	233.7 (112.8 to 391.1)	179.1 (72.7 to 340.2)
			75 plus	765 (609 to 948)	327 (253 to 418)	439 (327 to 582)	1681 (1347 to 2022)	711 (517 to 911)	970 (762 to 1222)	119.6 (55.2 to 195.4)	117.5 (36.7 to 211.1)	121.1 (42.2 to 237.8)
		Rate	0 to 9	1.4 (0.8 to 1.9)	1.3 (0.7 to 2)	1.4 (0.8 to 2.2)	0.3 (0.2 to 0.4)	0.3 (0.2 to 0.4)	0.3 (0.2 to 0.5)	-78.8 (-87.6 to -59.2)	-78.6 (-89.8 to -54.9)	-79.1 (-90 to -48.4)
			10 to 24	1.3 (0.9 to 1.8)	1.7 (1.1 to 2.4)	1 (0.7 to 1.5)	1.2 (0.9 to 1.5)	1.6 (1.1 to 2.3)	0.8 (0.6 to 1.1)	-14.2 (-45.2 to 30.5)	-7.5 (-47.8 to 51.5)	-21.5 (-52.9 to 29.7)
			25 to 49	6.7 (4.7 to 9.2)	8 (5.5 to 11.1)	6 (4 to 8.4)	7.6 (5.3 to 10.5)	9.1 (6.2 to 12.8)	6.6 (4.4 to 9.7)	12.4 (-27.4 to 79.9)	13.9 (-31.7 to 88.6)	10.4 (-32.5 to 85.6)

Country	Measure	Metric	Age	Year						% Change (1990 to 2019)		
				1990			2019					
				Both	Female	Male	Both	Female	Male	Both	Female	Male
			50 to 74	108 (81.1 to 144)	103.5 (78.7 to 136.9)	111 (79.8 to 153.2)	91.3 (70.7 to 113.6)	96.1 (71.2 to 124.6)	88.2 (66.2 to 115.3)	-15.5 (-43.8 to 21)	-7.1 (-40.8 to 36.7)	-20.6 (-50.9 to 25.3)
			75 plus	573.4 (456.3 to 709.9)	504.8 (391.5 to 646.5)	637.8 (475.9 to 845.6)	720.3 (577.2 to 866.4)	727.8 (529.5 to 933.4)	714.8 (561.3 to 900.4)	25.6 (-11.2 to 69)	44.2 (-9.4 to 106.2)	12.1 (-27.9 to 71.2)
	DALYs	Number	0 to 9	6577 (4390 to 9047)	3127 (1997 to 4694)	3450 (2132 to 5104)	2694 (1780 to 3979)	1329 (824 to 2028)	1365 (861 to 2081)	-59 (-75.6 to -31.5)	-57.5 (-77 to -25.5)	-60.4 (-78.3 to -25.7)
			10 to 24	6686 (5106 to 8437)	3880 (2771 to 5100)	2807 (2066 to 3783)	11816 (8836 to 15297)	6813 (4956 to 9009)	5003 (3513 to 6795)	76.7 (29.7 to 135.2)	75.6 (18.8 to 147.3)	78.3 (22.5 to 149.5)
			25 to 49	20153 (15226 to 25915)	9378 (6892 to 12447)	10775 (7783 to 14186)	94454 (72299 to 122543)	45394 (34155 to 59302)	49060 (35750 to 65214)	368.7 (228.5 to 561.7)	384.1 (227.3 to 602.4)	355.3 (210.9 to 572)
			50 to 74	35792 (27264 to 47136)	13863 (10727 to 18067)	21929 (15842 to 29742)	118249 (93401 to 145964)	50329 (38297 to 63644)	67921 (52305 to 86386)	230.4 (123.6 to 359.7)	263 (143.1 to 417)	209.7 (101.7 to 366.4)
			75 plus	10229 (8237 to 12671)	4476 (3562 to 5611)	5752 (4232 to 7560)	21976 (17845 to 26138)	8999 (6747 to 11336)	12977 (10331 to 16201)	114.8 (56.3 to 184.9)	101 (35 to 181.5)	125.6 (47.2 to 236.3)
		Rate	0 to 9	63.8 (42.6 to 87.8)	61.5 (39.3 to 92.4)	66 (40.8 to 97.7)	27.9 (18.4 to 41.2)	28.5 (17.6 to 43.4)	27.3 (17.2 to 41.6)	-56.3 (-73.9 to -27)	-53.7 (-75 to -18.9)	-58.7 (-77.3 to -22.3)
			10 to 24	131.5 (100.4 to 165.9)	165.5 (118.2 to 217.5)	102.4 (75.4 to 138)	142.7 (106.7 to 184.8)	184.8 (134.4 to 244.3)	109 (76.5 to 148)	8.6 (-20.3 to 44.5)	11.6 (-24.5 to 57.2)	6.4 (-26.9 to 49)
			25 to 49	411.4 (310.8 to 529)	503.2 (369.8 to 667.9)	355.1 (256.5 to 467.5)	511.4 (391.4 to 663.5)	628.6 (473 to 821.2)	436.1 (317.8 to 579.7)	24.3 (-12.9 to 75.5)	24.9 (-15.5 to 81.3)	22.8 (-16.1 to 81.3)
			50 to 74	1629 (1240.8 to 2145.2)	1611.6 (1247 to 2100.3)	1640.1 (1184.8 to 2224.5)	1490.2 (1177.1 to 1839.5)	1585.9 (1206.7 to 2005.5)	1426.4 (1098.5 to 1814.2)	-8.5 (-38.1 to 27.3)	-1.6 (-34.1 to 40.1)	-13 (-43.4 to 31)
			75 plus	5241.1 (4220.4 to 6492.7)	4641.7 (3693.2 to 5818)	5826.6 (4286.7 to 7657.5)	6408.7 (5203.9 to 7622.4)	6048 (4534.4 to 7618.4)	6685.2 (5321.8 to 8346)	22.3 (-11 to 62.2)	30.3 (-12.5 to 82.4)	14.7 (-25.2 to 71)

Country	Measure	Metric	Age	Year						% Change (1990 to 2019)		
				1990			2019					
				Both	Female	Male	Both	Female	Male	Both	Female	Male
Sudan	Deaths	Number	0 to 9	305 (87 to 501)	182 (42 to 349)	123 (31 to 233)	221 (129 to 341)	108 (55 to 177)	113 (62 to 204)	-27.6 (-64.2 to 135.8)	-40.7 (-74.9 to 111.9)	-8.3 (-59.2 to 296.9)
			10 to 24	83 (55 to 119)	50 (30 to 78)	33 (21 to 49)	144 (83 to 226)	74 (41 to 120)	69 (38 to 128)	72.4 (-7.7 to 206)	47.8 (-30.5 to 189.4)	109.8 (9.7 to 331.3)
			25 to 49	272 (194 to 368)	148 (100 to 222)	125 (85 to 172)	523 (297 to 847)	279 (148 to 441)	244 (129 to 442)	91.8 (4.8 to 227.8)	88.6 (-7.6 to 224.1)	95.6 (0.6 to 290.2)
			50 to 74	1218 (913 to 1732)	548 (388 to 836)	670 (481 to 1026)	2072 (1413 to 3250)	938 (606 to 1362)	1134 (697 to 1995)	70.1 (8.8 to 164.1)	71.2 (-3.4 to 162)	69.2 (5.1 to 203)
			75 plus	728 (542 to 1122)	318 (221 to 540)	410 (285 to 685)	1795 (1258 to 2889)	768 (512 to 1171)	1028 (650 to 1886)	146.5 (62.9 to 270.6)	141 (36.7 to 257.1)	150.8 (67.4 to 340.1)
		Rate	0 to 9	4.6 (1.3 to 7.6)	5.8 (1.3 to 11.1)	3.6 (0.9 to 6.8)	2 (1.2 to 3.2)	2.1 (1.1 to 3.4)	2 (1.1 to 3.7)	-55.9 (-78.2 to 43.7)	-64.3 (-84.9 to 27.7)	-43.5 (-74.9 to 144.5)
			10 to 24	1.3 (0.9 to 1.8)	1.6 (0.9 to 2.4)	1 (0.6 to 1.5)	1.1 (0.6 to 1.7)	1.1 (0.6 to 1.8)	1 (0.5 to 1.9)	-17.3 (-55.7 to 46.8)	-26.9 (-65.6 to 43.2)	-2.3 (-48.9 to 100.8)
			25 to 49	5.3 (3.8 to 7.1)	5.5 (3.8 to 8.3)	5 (3.4 to 6.9)	4.2 (2.4 to 6.8)	4.3 (2.3 to 6.8)	4.1 (2.2 to 7.4)	-20.3 (-56.5 to 36.2)	-22.3 (-61.9 to 33.5)	-17.9 (-57.8 to 63.7)
			50 to 74	68.4 (51.3 to 97.3)	65.5 (46.5 to 100.1)	70.9 (50.8 to 108.5)	56.2 (38.3 to 88.1)	55.6 (36 to 80.9)	56.6 (34.8 to 99.6)	-17.8 (-47.5 to 27.5)	-15.1 (-52.1 to 29.9)	-20.1 (-50.4 to 43)
			75 plus	309.4 (230.4 to 476.8)	272.1 (188.5 to 461.1)	346.2 (241 to 579)	376.2 (263.6 to 605.4)	355 (237 to 541.6)	393.8 (248.9 to 722.7)	21.6 (-19.7 to 82.8)	30.5 (-26 to 93.3)	13.7 (-24.1 to 99.5)
	DALYs	Number	0 to 9	27700 (8995 to 44422)	16438 (4346 to 31103)	11262 (3306 to 20651)	22670 (14196 to 33645)	11233 (6578 to 17525)	11437 (6717 to 19376)	-18.2 (-57.1 to 140.5)	-31.7 (-69.5 to 120.6)	1.6 (-51.5 to 274.2)
			10 to 24	7833 (5702 to 10366)	4826 (3299 to 6825)	3008 (2075 to 4154)	16706 (11445 to 23639)	9309 (6408 to 13005)	7397 (4696 to 11937)	113.3 (39.6 to 217.2)	92.9 (12.4 to 202.6)	145.9 (52.2 to 317)

Country	Measure	Metric	Age	Year						% Change (1990 to 2019)		
				1990			2019					
				Both	Female	Male	Both	Female	Male	Both	Female	Male
			25 to 49	16589 (12582 to 21297)	9421 (6965 to 13262)	7168 (5149 to 9513)	37741 (25303 to 54850)	21418 (14130 to 29751)	16322 (10532 to 26458)	127.5 (50.5 to 238.7)	127.3 (38.1 to 238)	127.7 (40.5 to 290.3)
			50 to 74	34202 (26344 to 47014)	15903 (11737 to 23273)	18298 (13638 to 26936)	63599 (45679 to 94343)	29357 (20200 to 40838)	34242 (22620 to 57074)	86 (23.3 to 171.2)	84.6 (12.3 to 166.2)	87.1 (21.1 to 217.6)
			75 plus	10360 (7845 to 15060)	4683 (3404 to 7383)	5678 (4009 to 9367)	25124 (18525 to 37994)	10881 (7763 to 15682)	14243 (9625 to 24522)	142.5 (69.7 to 246.8)	132.4 (43 to 229.5)	150.9 (70.1 to 311.1)
		Rate	0 to 9	186.3 (60.5 to 298.7)	231.3 (61.2 to 437.7)	145.1 (42.6 to 266)	98.8 (61.9 to 146.7)	100.8 (59.1 to 157.3)	96.9 (56.9 to 164.2)	-47 (-72.2 to 55.9)	-56.4 (-80.5 to 40.7)	-33.2 (-68.1 to 146.1)
			10 to 24	121.4 (88.4 to 160.7)	149.6 (102.3 to 211.6)	93.2 (64.3 to 128.7)	124.2 (85.1 to 175.8)	142.7 (98.3 to 199.4)	106.8 (67.8 to 172.3)	2.3 (-33 to 52.2)	-4.6 (-44.4 to 49.7)	14.5 (-29.1 to 94.2)
			25 to 49	321.5 (243.8 to 412.7)	353.2 (261.1 to 497.2)	287.6 (206.6 to 381.6)	304 (203.8 to 441.8)	330.8 (218.2 to 459.5)	274.7 (177.2 to 445.3)	-5.5 (-37.5 to 40.8)	-6.3 (-43.1 to 39.2)	-4.5 (-41 to 63.7)
			50 to 74	1028.1 (791.9 to 1413.3)	1017.2 (750.7 to 1488.6)	1037.8 (773.5 to 1527.7)	906.6 (651.2 to 1344.9)	913.7 (628.7 to 1271)	900.6 (595 to 1501.2)	-11.8 (-41.5 to 28.6)	-10.2 (-45.4 to 29.5)	-13.2 (-43.8 to 47.3)
			75 plus	3135.4 (2374.3 to 4557.8)	2811.6 (2043.7 to 4433)	3464.5 (2446.2 to 5715.7)	3520 (2595.5 to 5323.2)	3324.4 (2371.8 to 4791.3)	3685.7 (2490.6 to 6345.5)	12.3 (-21.4 to 60.5)	18.2 (-27.2 to 67.7)	6.4 (-27.9 to 74.3)
Syrian Arab Republic	Deaths	Number	0 to 9	154 (102 to 204)	72 (45 to 104)	82 (47 to 119)	27 (20 to 35)	13 (9 to 17)	14 (10 to 20)	-82.4 (-88.4 to -71.1)	-82.4 (-89.2 to -69.6)	-82.3 (-89.3 to -65.7)
			10 to 24	152 (121 to 194)	78 (58 to 103)	74 (55 to 99)	83 (62 to 109)	46 (33 to 63)	37 (26 to 50)	-45.4 (-61.8 to -22.6)	-40.8 (-61.2 to -9.4)	-50.3 (-68.1 to -23.7)
			25 to 49	258 (198 to 331)	133 (96 to 180)	125 (95 to 160)	206 (147 to 286)	108 (71 to 160)	99 (70 to 138)	-20 (-46.6 to 23.7)	-18.8 (-50 to 31.9)	-21.2 (-47.2 to 20.9)
			50 to 74	773 (607 to 997)	346 (265 to 448)	427 (316 to 578)	1321 (981 to 1771)	649 (471 to 884)	672 (470 to 927)	70.9 (12.7 to 153.5)	87.3 (23.8 to 179)	57.6 (-3.2 to 148.3)

Country	Measure	Metric	Age	Year						% Change (1990 to 2019)		
				1990			2019					
				Both	Female	Male	Both	Female	Male	Both	Female	Male
			75 plus	610 (494 to 802)	302 (236 to 428)	308 (235 to 422)	1023 (810 to 1272)	519 (399 to 651)	505 (375 to 656)	67.9 (17.2 to 124.2)	71.7 (12.3 to 135.3)	64.1 (2.3 to 135.7)
		Rate	0 to 9	3.6 (2.3 to 4.7)	3.4 (2.1 to 4.9)	3.7 (2.1 to 5.4)	1.1 (0.8 to 1.5)	1.1 (0.8 to 1.5)	1.2 (0.8 to 1.7)	-67.9 (-78.8 to -47.3)	-68 (-80.3 to -44.4)	-67.8 (-80.4 to -37.5)
			10 to 24	3.5 (2.8 to 4.5)	3.7 (2.7 to 4.8)	3.3 (2.5 to 4.5)	1.7 (1.3 to 2.2)	1.9 (1.3 to 2.5)	1.5 (1.1 to 2.1)	-51.2 (-65.9 to -30.9)	-49.3 (-66.7 to -22.3)	-53.7 (-70.3 to -28.9)
			25 to 49	8.5 (6.6 to 11)	8.9 (6.5 to 12.1)	8.2 (6.2 to 10.5)	4.9 (3.5 to 6.8)	4.6 (3.1 to 6.9)	5.2 (3.7 to 7.2)	-42.9 (-61.9 to -11.6)	-48 (-68 to -15.5)	-36.7 (-57.6 to -2.9)
			50 to 74	72.3 (56.7 to 93.2)	67.6 (51.7 to 87.3)	76.6 (56.7 to 103.8)	48.5 (36 to 65)	48.6 (35.3 to 66.2)	48.4 (33.8 to 66.7)	-32.9 (-55.8 to -0.5)	-28.1 (-52.4 to 7.2)	-36.8 (-61.2 to -0.5)
			75 plus	488.2 (396 to 641.9)	510.1 (398.9 to 722.4)	468.5 (357.7 to 642.1)	352 (278.7 to 437.7)	382.8 (294.5 to 480.4)	325.1 (241.6 to 422.7)	-27.9 (-49.6 to -3.7)	-24.9 (-50.9 to 2.8)	-30.6 (-56.7 to -0.3)
	DALYs	Number	0 to 9	14441 (9904 to 18996)	6882 (4494 to 9651)	7559 (4544 to 10771)	3040 (2339 to 3870)	1469 (1088 to 1931)	1571 (1140 to 2100)	-78.9 (-85.6 to -67.9)	-78.7 (-86.2 to -66.8)	-79.2 (-87 to -62.3)
			10 to 24	13111 (10686 to 16169)	7015 (5493 to 8788)	6095 (4686 to 7919)	8493 (6620 to 10709)	4897 (3699 to 6313)	3596 (2718 to 4768)	-35.2 (-51.2 to -15.7)	-30.2 (-50.6 to -4.4)	-41 (-58.3 to -15.8)
			25 to 49	15983 (12621 to 19877)	8607 (6570 to 11126)	7375 (5793 to 9233)	14594 (11287 to 18954)	8215 (6103 to 10984)	6380 (4844 to 8508)	-8.7 (-33.6 to 24.8)	-4.6 (-32.2 to 35.5)	-13.5 (-37.8 to 22.6)
			50 to 74	22937 (18402 to 28904)	10585 (8218 to 13258)	12352 (9401 to 16470)	42665 (33286 to 54907)	21024 (16080 to 27294)	21641 (16098 to 28480)	86 (30 to 164)	98.6 (39.7 to 184.6)	75.2 (16.3 to 163.2)
			75 plus	7339 (6026 to 9441)	3689 (2957 to 4897)	3650 (2807 to 5040)	14751 (11911 to 18092)	7554 (6006 to 9308)	7198 (5545 to 9223)	101 (47.1 to 160.7)	104.8 (45.5 to 170.9)	97.2 (25.5 to 178)
		Rate	0 to 9	153.5 (105.3 to 202)	150.2 (98.1 to 210.6)	156.7 (94.2 to 223.3)	62.4 (48 to 79.4)	62 (45.9 to 81.5)	62.8 (45.5 to 83.9)	-59.4 (-72.1 to -38)	-58.7 (-73.2 to -35.8)	-60 (-74.9 to -27.4)

Country	Measure	Metric	Age	Year						% Change (1990 to 2019)		
				1990			2019					
				Both	Female	Male	Both	Female	Male	Both	Female	Male
			10 to 24	300.8 (245.1 to 370.9)	328.7 (257.4 to 411.8)	274 (210.6 to 356)	174.1 (135.7 to 219.6)	196.7 (148.6 to 253.6)	150.5 (113.8 to 199.6)	-42.1 (-56.4 to -24.7)	-40.1 (-57.6 to -18)	-45.1 (-61.2 to -21.5)
			25 to 49	529.2 (417.9 to 658.2)	578.7 (441.7 to 748)	481.2 (378 to 602.5)	345.1 (266.9 to 448.2)	353.9 (262.9 to 473.2)	334.4 (253.9 to 446)	-34.8 (-52.6 to -10.9)	-38.8 (-56.5 to -13.2)	-30.5 (-50 to -1.5)
			50 to 74	1124.3 (902 to 1416.7)	1081.3 (839.5 to 1354.3)	1163.9 (885.9 to 1551.9)	819.8 (639.6 to 1055)	825.3 (631.2 to 1071.5)	814.5 (605.9 to 1071.9)	-27.1 (-49 to 3.5)	-23.7 (-46.3 to 9.4)	-30 (-53.6 to 5.1)
			75 plus	3821.4 (3137.8 to 4916.4)	4039.3 (3237.3 to 5361.6)	3623.8 (2787.2 to 5004.2)	3498.6 (2825 to 4291)	3935.9 (3129.7 to 4850)	3133.3 (2413.8 to 4014.9)	-8.4 (-33 to 18.8)	-2.6 (-30.8 to 28.9)	-13.5 (-45 to 21.9)
Tunisia	Deaths	Number	0 to 9	54 (30 to 75)	30 (16 to 46)	23 (11 to 37)	11 (8 to 15)	4 (2 to 6)	7 (5 to 10)	-79.2 (-87.7 to -57.3)	-86.5 (-94.4 to -66.1)	-69.6 (-84.1 to -21.5)
			10 to 24	24 (19 to 30)	14 (10 to 18)	10 (7 to 14)	16 (11 to 22)	9 (5 to 12)	8 (5 to 12)	-33.3 (-54.7 to -2.7)	-38.9 (-61.2 to -3.5)	-25.8 (-56 to 22.2)
			25 to 49	75 (61 to 91)	40 (32 to 53)	34 (25 to 45)	141 (94 to 196)	72 (45 to 105)	69 (45 to 99)	89.8 (23.9 to 181)	79.3 (7.3 to 182.6)	102.2 (30 to 209.7)
			50 to 74	477 (389 to 606)	230 (182 to 329)	248 (186 to 334)	1087 (754 to 1507)	547 (364 to 753)	540 (359 to 802)	127.7 (49.7 to 231.3)	138 (53.8 to 254.2)	118.1 (25.6 to 242.1)
			75 plus	401 (327 to 502)	180 (139 to 249)	221 (166 to 290)	1379 (983 to 1794)	730 (506 to 981)	649 (436 to 916)	244 (138.3 to 363.9)	305.4 (170.3 to 478)	193.9 (84.4 to 338.5)
		Rate	0 to 9	2.4 (1.4 to 3.4)	2.8 (1.5 to 4.3)	2.1 (0.9 to 3.3)	0.6 (0.4 to 0.9)	0.5 (0.3 to 0.7)	0.8 (0.5 to 1.1)	-74.1 (-84.7 to -46.9)	-83.1 (-92.9 to -57.3)	-62.7 (-80.4 to -3.6)
			10 to 24	0.9 (0.7 to 1.1)	1.1 (0.8 to 1.4)	0.7 (0.5 to 1)	0.6 (0.5 to 0.9)	0.7 (0.4 to 1)	0.6 (0.4 to 0.9)	-28.3 (-51.2 to 4.8)	-33.8 (-58 to 4.5)	-20.6 (-52.9 to 30.8)
			25 to 49	3.1 (2.5 to 3.8)	3.3 (2.6 to 4.4)	2.8 (2.1 to 3.7)	3.2 (2.1 to 4.4)	3.2 (2 to 4.6)	3.2 (2.1 to 4.6)	3.3 (-32.6 to 52.9)	-4.4 (-42.8 to 50.7)	12.3 (-27.8 to 72)

Country	Measure	Metric	Age	Year						% Change (1990 to 2019)		
				1990			2019					
				Both	Female	Male	Both	Female	Male	Both	Female	Male
			50 to 74	46.8 (38.1 to 59.4)	46.2 (36.6 to 66)	47.3 (35.6 to 63.8)	43.4 (30.1 to 60.2)	43.3 (28.8 to 59.6)	43.5 (28.9 to 64.7)	-7.1 (-38.9 to 35.1)	-6.2 (-39.4 to 39.5)	-7.9 (-47 to 44.4)
			75 plus	310.8 (253.4 to 389.1)	296.4 (228.5 to 410.5)	323.5 (242.8 to 425.7)	359.3 (256.3 to 467.5)	352.7 (244.7 to 474.1)	367.1 (246.8 to 518.6)	15.6 (-19.9 to 55.9)	19 (-20.7 to 69.7)	13.5 (-28.8 to 69.3)
	DALYs	Number	0 to 9	5117 (3113 to 7000)	2873 (1600 to 4294)	2244 (1135 to 3460)	1491 (1071 to 2011)	608 (379 to 889)	883 (623 to 1233)	-70.9 (-81.4 to -47.5)	-78.8 (-88.9 to -55.6)	-60.7 (-77.3 to -13)
			10 to 24	2669 (2158 to 3271)	1583 (1239 to 1989)	1086 (826 to 1382)	2345 (1686 to 3187)	1282 (895 to 1758)	1063 (745 to 1500)	-12.1 (-33 to 10.4)	-19 (-39.7 to 7)	-2.1 (-29.9 to 32.4)
			25 to 49	5449 (4562 to 6491)	3110 (2555 to 3827)	2339 (1864 to 2885)	11604 (8686 to 14900)	6329 (4649 to 8300)	5275 (3902 to 6865)	113 (63.7 to 172.3)	103.5 (50.4 to 169.6)	125.5 (67.8 to 201.7)
			50 to 74	14047 (11826 to 17321)	6874 (5634 to 9421)	7172 (5641 to 9161)	34689 (25871 to 45756)	17253 (12466 to 22855)	17436 (12644 to 23975)	147 (77.4 to 237.3)	151 (73.5 to 244.5)	143.1 (60 to 250.7)
			75 plus	5689 (4693 to 7047)	2565 (2033 to 3422)	3124 (2399 to 4040)	18678 (13942 to 23514)	9671 (7028 to 12400)	9007 (6437 to 12089)	228.3 (136.9 to 328.1)	277.1 (165.8 to 418.3)	188.3 (91.5 to 308.2)
		Rate	0 to 9	108.6 (66.1 to 148.6)	125.3 (69.8 to 187.3)	92.8 (46.9 to 143.1)	40.5 (29.1 to 54.6)	34.3 (21.4 to 50.2)	46.3 (32.6 to 64.6)	-62.7 (-76.2 to -32.8)	-72.6 (-85.7 to -42.6)	-50.2 (-71.3 to 10.2)
			10 to 24	99.9 (80.7 to 122.4)	120.3 (94.1 to 151.2)	80 (60.9 to 101.8)	94.4 (67.9 to 128.3)	105.5 (73.6 to 144.7)	83.8 (58.7 to 118.3)	-5.4 (-27.8 to 18.9)	-12.4 (-34.7 to 15.9)	4.8 (-25 to 41.8)
			25 to 49	226.3 (189.4 to 269.5)	256.9 (211.1 to 316.2)	195.3 (155.6 to 240.8)	262.2 (196.3 to 336.6)	278.9 (204.9 to 365.7)	244.6 (181 to 318.3)	15.9 (-10.9 to 48.1)	8.5 (-19.8 to 43.8)	25.3 (-6.8 to 67.6)
			50 to 74	728.9 (613.7 to 898.8)	729.5 (597.9 to 999.7)	728.2 (572.8 to 930.2)	732.9 (546.6 to 966.7)	723 (522.4 to 957.7)	743 (538.8 to 1021.6)	0.6 (-27.8 to 37.3)	-0.9 (-31.5 to 36)	2 (-32.9 to 47.2)
			75 plus	3104.7 (2561.3 to 3845.7)	2916.8 (2312.2 to 3892)	3278.1 (2516.6 to 4239.4)	3177.6 (2371.8 to 4000.3)	3020.9 (2195.2 to 3873.2)	3365.1 (2405.1 to 4516.9)	2.3 (-26.2 to 33.5)	3.6 (-27 to 42.4)	2.7 (-31.8 to 45.3)

Country	Measure	Metric	Age	Year						% Change (1990 to 2019)		
				1990			2019					
				Both	Female	Male	Both	Female	Male	Both	Female	Male
Turkey	Deaths	Number	0 to 9	727 (423 to 1010)	369 (192 to 609)	358 (174 to 549)	87 (64 to 112)	39 (27 to 51)	48 (34 to 66)	-88.1 (-92.7 to -78.2)	-89.6 (-94.6 to -78.7)	-86.6 (-92.6 to -68.4)
			10 to 24	264 (202 to 360)	135 (101 to 194)	129 (90 to 187)	109 (87 to 140)	59 (46 to 75)	51 (38 to 70)	-58.5 (-73.3 to -40.3)	-56.6 (-73.4 to -34.6)	-60.5 (-76 to -36.8)
			25 to 49	959 (796 to 1183)	399 (306 to 552)	560 (432 to 705)	840 (650 to 1076)	332 (250 to 434)	508 (388 to 658)	-12.4 (-37.9 to 20.4)	-16.8 (-44.9 to 24.1)	-9.3 (-37.1 to 32.8)
			50 to 74	5225 (4173 to 6980)	2357 (1798 to 3342)	2868 (2110 to 4003)	7610 (6017 to 9594)	3679 (2879 to 4744)	3931 (3008 to 5082)	45.6 (-2.3 to 104.3)	56.1 (-0.1 to 124.7)	37.1 (-13.1 to 108.1)
			75 plus	4432 (3281 to 7039)	2181 (1631 to 3770)	2251 (1535 to 3775)	10547 (8385 to 12821)	6040 (4681 to 7589)	4507 (3534 to 5568)	137.9 (40.4 to 228.4)	176.9 (52.9 to 294)	100.2 (14 to 216.7)
		Rate	0 to 9	5 (2.9 to 6.9)	5.2 (2.7 to 8.6)	4.8 (2.3 to 7.3)	0.9 (0.6 to 1.1)	0.8 (0.6 to 1.1)	0.9 (0.7 to 1.3)	-82.7 (-89.4 to -68.3)	-84.8 (-92.2 to -69)	-80.5 (-89.2 to -54.1)
			10 to 24	1.4 (1.1 to 1.9)	1.5 (1.1 to 2.1)	1.3 (0.9 to 1.9)	0.6 (0.5 to 0.7)	0.6 (0.5 to 0.8)	0.5 (0.4 to 0.7)	-58.5 (-73.4 to -40.4)	-56.2 (-73.2 to -34.1)	-60.9 (-76.2 to -37.5)
			25 to 49	5.3 (4.4 to 6.6)	4.5 (3.5 to 6.3)	6.1 (4.7 to 7.7)	2.6 (2 to 3.3)	2.1 (1.6 to 2.8)	3.1 (2.3 to 4)	-51.4 (-65.5 to -33.2)	-53.4 (-69.2 to -30.5)	-50 (-65.4 to -26.8)
			50 to 74	72.2 (57.7 to 96.4)	63.9 (48.7 to 90.5)	80.9 (59.5 to 112.9)	44.4 (35.1 to 56)	41.9 (32.8 to 54)	47 (36 to 60.8)	-38.5 (-58.8 to -13.7)	-34.4 (-58 to -5.5)	-41.9 (-63.2 to -11.8)
			75 plus	451.7 (334.4 to 717.4)	393.7 (294.3 to 680.4)	527.1 (359.3 to 883.8)	378.5 (300.9 to 460.1)	374.9 (290.5 to 471)	383.4 (300.6 to 473.6)	-16.2 (-50.6 to 15.6)	-4.8 (-47.4 to 35.5)	-27.3 (-58.6 to 15.1)
	DALYs	Number	0 to 9	66587 (40066 to 92327)	33837 (18363 to 54776)	32750 (16701 to 49550)	10406 (7967 to 13265)	4808 (3464 to 6306)	5599 (4196 to 7243)	-84.4 (-89.8 to -72.4)	-85.8 (-92.5 to -72.2)	-82.9 (-90.3 to -63.1)
			10 to 24	25539 (20362 to 33121)	13719 (10527 to 18420)	11820 (8923 to 16125)	16441 (12327 to 21138)	9184 (6803 to 12013)	7258 (5170 to 9642)	-35.6 (-54.8 to -16.3)	-33.1 (-53.4 to -10.3)	-38.6 (-59.8 to -13.6)

Country	Measure	Metric	Age	Year						% Change (1990 to 2019)		
				1990			2019					
				Both	Female	Male	Both	Female	Male	Both	Female	Male
			25 to 49	59792 (50171 to 71957)	27201 (21767 to 35202)	32591 (26302 to 40191)	71850 (57319 to 88634)	33666 (26151 to 41957)	38184 (29676 to 47916)	20.2 (-6.9 to 51.7)	23.8 (-8.1 to 61.4)	17.2 (-12.5 to 56.2)
			50 to 74	150209 (123186 to 194414)	68412 (53644 to 94729)	81797 (62267 to 109747)	240238 (195085 to 294259)	114826 (93490 to 142646)	125413 (99282 to 156290)	59.9 (13.1 to 114.1)	67.8 (15.9 to 126.7)	53.3 (2.4 to 121.2)
			75 plus	55336 (42769 to 84654)	27537 (21241 to 45096)	27799 (19435 to 45734)	140607 (115525 to 168753)	80754 (64705 to 97447)	59853 (48480 to 73035)	154.1 (58.7 to 238.1)	193.3 (70.7 to 297.6)	115.3 (23.1 to 227.7)
		Rate	0 to 9	212.4 (127.8 to 294.6)	221.4 (120.2 to 358.4)	203.9 (104 to 308.5)	50.1 (38.3 to 63.8)	47.5 (34.2 to 62.3)	52.5 (39.3 to 67.9)	-76.4 (-84.7 to -58.4)	-78.5 (-88.6 to -58.1)	-74.3 (-85.3 to -44.4)
			10 to 24	134.4 (107.2 to 174.4)	147.5 (113.2 to 198.1)	121.9 (92 to 166.3)	86.4 (64.8 to 111.1)	99.5 (73.7 to 130.2)	74.1 (52.8 to 98.4)	-35.7 (-54.9 to -16.4)	-32.6 (-53.1 to -9.6)	-39.2 (-60.2 to -14.5)
			25 to 49	332.6 (279.1 to 400.3)	308.4 (246.8 to 399.1)	355.9 (287.2 to 438.9)	221.9 (177.1 to 273.8)	213.7 (166 to 266.3)	229.8 (178.6 to 288.3)	-33.3 (-48.3 to -15.8)	-30.7 (-48.6 to -9.7)	-35.4 (-51.8 to -13.9)
			50 to 74	1083.2 (888.4 to 1402)	969.2 (760 to 1342)	1201.4 (914.6 to 1612)	744 (604.1 to 911.3)	694.9 (565.8 to 863.2)	795.4 (629.7 to 991.3)	-31.3 (-51.4 to -8.1)	-28.3 (-50.5 to -3.2)	-33.8 (-55.8 to -4.5)
			75 plus	3639.8 (2813.1 to 5568.2)	3183.7 (2455.7 to 5213.8)	4241.7 (2965.4 to 6978.3)	3215 (2641.5 to 3858.6)	3206.2 (2569 to 3869)	3227 (2613.8 to 3937.7)	-11.7 (-44.8 to 17.5)	0.7 (-41.4 to 36.5)	-23.9 (-56.5 to 15.8)
United Arab Emirates	Deaths	Number	0 to 9	8 (6 to 12)	3 (2 to 5)	5 (3 to 7)	5 (3 to 13)	2 (1 to 6)	3 (1 to 8)	-36 (-66.4 to 34)	-37.4 (-76.1 to 37.6)	-35 (-73.2 to 68.7)
			10 to 24	10 (7 to 13)	4 (3 to 6)	6 (4 to 8)	18 (11 to 30)	8 (4 to 14)	9 (5 to 18)	84.5 (8.7 to 202)	114.6 (2.2 to 266.7)	64.2 (-18.1 to 213.3)
			25 to 49	66 (48 to 93)	14 (9 to 20)	52 (36 to 78)	610 (364 to 1050)	119 (67 to 181)	491 (271 to 920)	821.1 (442.9 to 1393.7)	761.8 (325.3 to 1363.2)	836.6 (423.5 to 1510.8)
			50 to 74	94 (61 to 120)	37 (21 to 52)	58 (36 to 78)	709 (433 to 1140)	191 (100 to 271)	518 (302 to 935)	653.6 (375.5 to 1060.1)	422.3 (185.3 to 720.7)	800.7 (443.7 to 1392.7)

Country	Measure	Metric	Age	Year						% Change (1990 to 2019)		
				1990			2019					
				Both	Female	Male	Both	Female	Male	Both	Female	Male
			75 plus	29 (16 to 37)	12 (7 to 17)	17 (9 to 23)	91 (54 to 137)	34 (16 to 47)	57 (32 to 99)	215.2 (113.4 to 364.3)	178 (54.3 to 330.5)	242.3 (114.1 to 460.5)
		Rate	0 to 9	1.8 (1.2 to 2.7)	1.5 (0.9 to 2.5)	2 (1.3 to 3.2)	0.6 (0.3 to 1.6)	0.5 (0.3 to 1.5)	0.7 (0.3 to 2.1)	-63.2 (-80.7 to -23)	-64.3 (-86.4 to -21.5)	-62.4 (-84.5 to -2.5)
			10 to 24	2.3 (1.7 to 3.1)	2.1 (1.4 to 3.2)	2.4 (1.6 to 3.5)	1.7 (1 to 2.8)	1.6 (0.9 to 2.8)	1.7 (0.9 to 3.3)	-26.5 (-56.7 to 20.3)	-22.5 (-63.1 to 32.5)	-29 (-64.6 to 35.4)
			25 to 49	7.3 (5.3 to 10.3)	6 (4 to 8.8)	7.8 (5.3 to 11.5)	9.7 (5.8 to 16.7)	8.5 (4.8 to 13)	10 (5.5 to 18.7)	32.2 (-22.1 to 114.4)	43.2 (-29.4 to 143.1)	28.4 (-28.2 to 120.9)
			50 to 74	98.4 (63.6 to 124.9)	127.9 (74.6 to 180.2)	85.8 (53 to 115.8)	65.3 (39.8 to 104.9)	77.6 (40.7 to 110)	61.6 (35.9 to 111.2)	-33.7 (-58.1 to 2.1)	-39.3 (-66.9 to -4.7)	-28.2 (-56.6 to 19.1)
			75 plus	692.5 (392.4 to 879.8)	677.4 (379.9 to 969.3)	703.9 (393.7 to 967)	564.7 (331.7 to 850.1)	571.6 (261.8 to 801.4)	560.8 (315.1 to 970.5)	-18.4 (-44.8 to 20.1)	-15.6 (-53.2 to 30.7)	-20.3 (-50.2 to 30.5)
	DALYs	Number	0 to 9	780 (566 to 1127)	338 (225 to 529)	442 (304 to 683)	694 (411 to 1334)	310 (189 to 616)	383 (200 to 831)	-11 (-44.6 to 49.7)	-8.1 (-52.6 to 58)	-13.2 (-51.9 to 75.8)
			10 to 24	867 (680 to 1093)	376 (277 to 519)	490 (346 to 674)	1855 (1244 to 2770)	921 (616 to 1411)	933 (558 to 1601)	114 (46.3 to 202.5)	144.7 (43.8 to 253)	90.4 (17.3 to 216)
			25 to 49	4053 (3107 to 5468)	967 (705 to 1333)	3086 (2246 to 4349)	37199 (24653 to 58680)	7830 (5138 to 11232)	29369 (18554 to 49485)	817.8 (497.5 to 1253)	709.5 (392.9 to 1108.4)	851.7 (494.8 to 1421.7)
			50 to 74	2850 (1955 to 3596)	1066 (661 to 1469)	1785 (1166 to 2404)	24548 (16316 to 37712)	6401 (3753 to 8696)	18147 (11500 to 30906)	761.2 (474.3 to 1159.2)	500.7 (252.4 to 800.4)	916.7 (551 to 1529.6)
			75 plus	415 (249 to 524)	174 (105 to 241)	241 (143 to 330)	1338 (867 to 1915)	504 (274 to 685)	834 (522 to 1356)	222.2 (127.5 to 359)	189.6 (71.9 to 335.7)	245.8 (121 to 454.3)
		Rate	0 to 9	82.2 (59.7 to 118.8)	73.5 (48.9 to 115.1)	90.4 (62.2 to 139.7)	46.2 (27.4 to 88.9)	42.5 (25.9 to 84.3)	49.8 (26 to 107.9)	-43.8 (-65 to -5.4)	-42.1 (-70.2 to -0.5)	-45 (-69.5 to 11.5)

Country	Measure	Metric	Age	Year						% Change (1990 to 2019)		
				1990			2019					
				Both	Female	Male	Both	Female	Male	Both	Female	Male
			10 to 24	205.5 (161.3 to 259.2)	204.9 (150.9 to 282.5)	205.9 (145.3 to 283)	175.1 (117.4 to 261.5)	181.1 (121.1 to 277.5)	169.5 (101.3 to 290.7)	-14.8 (-41.7 to 20.4)	-11.6 (-48 to 27.6)	-17.7 (-49.3 to 36.6)
			25 to 49	448 (343.4 to 604.4)	418.4 (304.9 to 576.4)	458.1 (333.4 to 645.7)	590.1 (391.1 to 930.9)	562.6 (369.2 to 807)	597.9 (377.8 to 1007.5)	31.7 (-14.2 to 94.2)	34.5 (-18.1 to 100.7)	30.5 (-18.4 to 108.7)
			50 to 74	1531.6 (1050.6 to 1932.5)	1943.3 (1205.2 to 2679.3)	1359.7 (888.3 to 1831.1)	1141.7 (758.9 to 1754)	1321.2 (774.8 to 1795)	1089.5 (690.4 to 1855.5)	-25.5 (-50.3 to 9)	-32 (-60.1 to 1.9)	-19.9 (-48.7 to 28.4)
			75 plus	7428.4 (4459.9 to 9379.2)	7322.2 (4403.2 to 10116.1)	7507 (4457.3 to 10284.4)	6024.5 (3901.7 to 8620.3)	6410.1 (3485.1 to 8704)	5813 (3640.5 to 9456.3)	-18.9 (-42.7 to 15.5)	-12.5 (-48 to 31.7)	-22.6 (-50.5 to 24.1)
Yemen	Deaths	Number	0 to 9	131 (47 to 216)	74 (19 to 150)	57 (17 to 103)	108 (68 to 163)	56 (31 to 94)	52 (31 to 85)	-18 (-50.9 to 132.2)	-24.8 (-58 to 163.3)	-9.2 (-54 to 222.9)
			10 to 24	29 (14 to 50)	16 (7 to 31)	14 (6 to 22)	66 (38 to 98)	33 (18 to 54)	32 (18 to 50)	122.4 (14.5 to 386.3)	112.7 (1.8 to 412.1)	133.5 (16.7 to 448.5)
			25 to 49	134 (83 to 200)	66 (38 to 115)	68 (39 to 104)	341 (219 to 498)	169 (101 to 268)	172 (110 to 259)	154.6 (60.6 to 317.9)	157.1 (45 to 337.8)	152.2 (51.6 to 337)
			50 to 74	602 (434 to 841)	272 (186 to 432)	330 (233 to 476)	1321 (965 to 1783)	627 (447 to 893)	693 (486 to 969)	119.4 (56.3 to 210.6)	130.4 (53.8 to 241.6)	110.4 (40.9 to 216.8)
			75 plus	253 (185 to 368)	142 (96 to 232)	111 (77 to 177)	841 (642 to 1152)	380 (283 to 542)	461 (330 to 678)	232.3 (146.8 to 337.6)	167.8 (78 to 281.1)	314.5 (195.7 to 488.1)
		Rate	0 to 9	2.5 (0.9 to 4.1)	2.9 (0.8 to 5.9)	2.1 (0.6 to 3.8)	1.2 (0.8 to 1.8)	1.3 (0.7 to 2.2)	1.1 (0.7 to 1.9)	-51.3 (-70.9 to 37.8)	-55.7 (-75.3 to 55.1)	-45.7 (-72.5 to 93)
			10 to 24	0.7 (0.3 to 1.2)	0.8 (0.3 to 1.5)	0.6 (0.3 to 1)	0.6 (0.4 to 1)	0.7 (0.4 to 1.1)	0.6 (0.4 to 1)	-7.6 (-52.4 to 102)	-13.1 (-58.4 to 109.2)	-1.4 (-50.7 to 131.6)
			25 to 49	4.3 (2.6 to 6.4)	4.1 (2.3 to 7.1)	4.4 (2.5 to 6.8)	3.6 (2.3 to 5.2)	3.5 (2.1 to 5.6)	3.6 (2.3 to 5.4)	-16.2 (-47.1 to 37.6)	-13.7 (-51.3 to 46.9)	-18.6 (-51.1 to 41.1)

Country	Measure	Metric	Age	Year						% Change (1990 to 2019)		
				1990			2019					
				Both	Female	Male	Both	Female	Male	Both	Female	Male
			50 to 74	60.1 (43.4 to 83.9)	56.1 (38.3 to 89)	63.8 (45 to 92.1)	50.5 (36.9 to 68.2)	47.3 (33.7 to 67.4)	53.7 (37.7 to 75.1)	-15.9 (-40.1 to 19)	-15.6 (-43.7 to 25.2)	-15.8 (-43.6 to 26.8)
			75 plus	266.4 (195 to 387)	230.9 (156.7 to 377.3)	331.4 (229.3 to 526.4)	281.4 (214.8 to 385.5)	246.4 (183.8 to 351.4)	318.7 (228.6 to 468.9)	5.6 (-21.5 to 39.1)	6.7 (-29.1 to 51.8)	-3.8 (-31.4 to 36.4)
	DALYs	Number	0 to 9	12474 (5217 to 19802)	6984 (2164 to 13521)	5491 (1964 to 9556)	12342 (8535 to 17472)	6426 (4147 to 9945)	5916 (3942 to 8817)	-1.1 (-36.5 to 141.2)	-8 (-46.6 to 165.1)	7.8 (-38.8 to 210.4)
			10 to 24	3355 (2093 to 4817)	1908 (1158 to 3011)	1447 (861 to 2101)	9607 (6681 to 12717)	5628 (3908 to 7529)	3979 (2674 to 5512)	186.3 (93.8 to 354.6)	195 (85.9 to 356.2)	174.9 (73.6 to 365.1)
			25 to 49	8651 (5956 to 11959)	4650 (3112 to 7284)	4001 (2508 to 5723)	26564 (19337 to 35198)	14900 (10599 to 20380)	11664 (8233 to 16105)	207 (115.9 to 335.1)	220.4 (113.7 to 360.9)	191.5 (94.7 to 348.3)
			50 to 74	17568 (13018 to 23923)	8100 (5717 to 12313)	9468 (6771 to 13432)	41192 (31564 to 53987)	20220 (15478 to 27991)	20972 (15431 to 28148)	134.5 (72.2 to 224.5)	149.6 (73 to 248.3)	121.5 (53.7 to 223.1)
			75 plus	3675 (2749 to 5153)	2099 (1504 to 3219)	1576 (1115 to 2502)	12532 (9951 to 16205)	5871 (4589 to 7791)	6660 (4955 to 9364)	241 (162.8 to 337.3)	179.7 (97.9 to 279.6)	322.7 (202.8 to 487.6)
		Rate	0 to 9	107.8 (45.1 to 171.1)	124.2 (38.5 to 240.4)	92.2 (33 to 160.5)	66.1 (45.7 to 93.6)	70.6 (45.5 to 109.2)	61.9 (41.2 to 92.2)	-38.6 (-60.6 to 49.6)	-43.2 (-67 to 63.7)	-32.9 (-61.9 to 93.3)
			10 to 24	79.5 (49.6 to 114.1)	93.9 (57 to 148.2)	66.1 (39.3 to 95.9)	94.5 (65.7 to 125.1)	113.1 (78.5 to 151.3)	76.7 (51.5 to 106.3)	19 (-19.5 to 88.9)	20.5 (-24.1 to 86.4)	16.1 (-26.7 to 96.4)
			25 to 49	274.9 (189.2 to 380)	288.9 (193.3 to 452.6)	260.2 (163.1 to 372.1)	277.9 (202.3 to 368.2)	310.7 (221 to 425)	244.9 (172.8 to 338.1)	1.1 (-28.9 to 43.3)	7.5 (-28.3 to 54.7)	-5.9 (-37.1 to 44.7)
			50 to 74	922.3 (683.4 to 1256)	884.4 (624.2 to 1344.3)	957.5 (684.8 to 1358.4)	829.7 (635.8 to 1087.4)	803.2 (614.8 to 1111.9)	856.9 (630.6 to 1150.2)	-10 (-33.9 to 24.5)	-9.2 (-37 to 26.7)	-10.5 (-37.9 to 30.6)
			75 plus	2735.7 (2046.7 to 3836.2)	2360.7 (1690.8 to 3619.7)	3470.3 (2455 to 5510.9)	2888.9 (2293.9 to 3735.7)	2603.4 (2034.9 to 3454.6)	3197.9 (2378.9 to 4495.8)	5.6 (-18.6 to 35.4)	10.3 (-22 to 49.7)	-7.8 (-34 to 28.1)

Data in parentheses are 95% Uncertainty Intervals (95% UIs)
