# Supplementary material for: Regional burden of chronic kidney disease in North Africa and Middle East during 1990–2019; Results from Global Burden of Disease study 2019
Source: Front Public Health. 2022 Oct 11;10:1015902. doi: 10.3389/fpubh.2022.1015902 (PMC9592811; doi:10.3389/fpubh.2022.1015902)
Supplement: Supplementary file 8 [file Data_Sheet_8.PDF]

Location	Measure	Risk factor	Attributed age-standardized rate (per 100,000)						% Change (1990 to 2019)		
			1990			2019					
			Both	Female	Male	Both	Female	Male	Both	Female	Male
North Africa and Middle East	Deaths	Non-optimal temperature	2.6 (1.4 to 3.8)	2.5 (1.3 to 3.8)	2.8 (1.4 to 4.2)	2.4 (1.3 to 3.4)	2.4 (1.4 to 3.4)	2.4 (1.3 to 3.4)	-8.5 (-31.2 to 29.5)	-2.6 (-31.5 to 49.2)	-14.6 (-38.4 to 25.9)
		Lead exposure	1.6 (1 to 2.4)	1.3 (0.7 to 2)	2 (1.3 to 3)	1.5 (0.9 to 2.1)	1.2 (0.7 to 1.8)	1.7 (1.2 to 2.5)	-9.6 (-26.4 to 8.2)	-6.5 (-30.4 to 11.8)	-12.8 (-30.8 to 10.1)
		Diet high in sodium	0.5 (0.1 to 2)	0.3 (0.1 to 1.3)	0.6 (0.1 to 2.7)	0.4 (0.1 to 1.7)	0.3 (0.1 to 1.2)	0.6 (0.1 to 2.3)	-10.9 (-31.3 to 19.8)	-7.7 (-38.2 to 29)	-14 (-37 to 30.1)
		High fasting plasma glucose	11.8 (9.2 to 15.2)	11.2 (8.7 to 15.5)	12.5 (9.6 to 16.8)	10.5 (8.2 to 13)	10.4 (7.9 to 13.3)	10.5 (8.2 to 13.8)	-10.9 (-29 to 7.4)	-6.4 (-30.4 to 11)	-15.5 (-35 to 10)
		High body-mass index	10.2 (5.8 to 15.7)	11.7 (7 to 17.4)	8.7 (4.5 to 14.7)	12.1 (7.3 to 17.2)	13.4 (8.2 to 18.9)	10.7 (6.3 to 16.2)	18.7 (-8.2 to 52.8)	15 (-16 to 44.1)	24.1 (-8.3 to 77)
		High systolic blood pressure	22 (18.6 to 28.3)	21.2 (17.8 to 29.7)	23 (19.1 to 30.3)	20 (16.9 to 23.5)	20.3 (16.6 to 24.1)	19.7 (16.5 to 24.7)	-8.9 (-27.9 to 8.7)	-3.9 (-30.3 to 13.2)	-14.1 (-34.2 to 11.1)
		Kidney dysfunction	34.7 (30.7 to 44.1)	33.6 (29.7 to 45.5)	36.2 (31.1 to 45.6)	30.4 (26.3 to 35.4)	30.9 (25.7 to 35.5)	29.9 (25.7 to 36.9)	-12.6 (-29.4 to 3.9)	-8.2 (-31.1 to 7.5)	-17.3 (-35.8 to 6.4)
	DALYs	Non-optimal temperature	54.9 (27.3 to 77.6)	53.9 (26.8 to 76.6)	56.1 (28.1 to 80.1)	46.1 (24.3 to 66.3)	46.9 (25.3 to 66.6)	45.3 (21.9 to 68.2)	-16 (-36.5 to 22.4)	-13.1 (-34.8 to 35.1)	-19.1 (-41.3 to 22.8)
		Lead exposure	34.2 (21.9 to 49.2)	28.1 (16.3 to 43)	40.3 (26.6 to 58.8)	28.7 (17.7 to 41.7)	23.9 (13 to 36.3)	33.5 (21.8 to 48.7)	-15.9 (-30.2 to -2.2)	-15 (-34.3 to -0.3)	-17 (-32.5 to 2.3)
		Diet high in sodium	10.9 (2.2 to 43.8)	7.9 (2.6 to 30.1)	14 (1.7 to 56.9)	10.3 (2.1 to 40.1)	7.5 (2.4 to 28.9)	13.1 (1.6 to 52)	-5.4 (-26.2 to 24.1)	-5.4 (-33.3 to 32)	-6.1 (-26.5 to 35.2)
		High fasting plasma glucose	261.5 (209.7 to 315)	255.9 (205.9 to 324)	267.9 (213.3 to 335.4)	240 (192.4 to 295.3)	239.3 (187 to 295.4)	240.7 (191.5 to 308.1)	-8.2 (-25 to 8.3)	-6.5 (-28.2 to 10.4)	-10.2 (-29.2 to 13.1)
		High body-mass index	245.4 (149.1 to 352.1)	287.7 (184.4 to 404.1)	203.7 (112.5 to 314.8)	300.1 (200 to 404.5)	331.5 (224.7 to 440.2)	269.6 (170.9 to 379.9)	22.3 (-1.5 to 52.7)	15.2 (-10.5 to 40.6)	32.3 (1.6 to 81.8)

Location	Measure	Risk factor	Attributed age-standardized rate (per 100,000)						% Change (1990 to 2019)		
			1990			2019					
			Both	Female	Male	Both	Female	Male	Both	Female	Male
		High systolic blood pressure	443.3 (383.1 to 537)	441.9 (377.2 to 563.3)	446.1 (376.8 to 556.1)	424.7 (354.4 to 496.8)	432.5 (351 to 514.8)	417.1 (347.1 to 515.2)	-4.2 (-21.3 to 12)	-2.1 (-24.1 to 14)	-6.5 (-26.2 to 16.9)
		Kidney dysfunction	834.9 (762.3 to 947.2)	853.6 (771.8 to 1006.8)	819.2 (737.4 to 939.5)	744.4 (646.1 to 851.8)	770 (653.6 to 879.5)	720.3 (622.1 to 872.5)	-10.8 (-23.9 to 2.4)	-9.8 (-25.3 to 3.3)	-12.1 (-27.1 to 7.5)
	YLLs	Non-optimal temperature	54.9 (27.3 to 77.6)	53.9 (26.8 to 76.6)	56.1 (28.1 to 80.1)	46.1 (24.3 to 66.3)	46.9 (25.3 to 66.6)	45.3 (21.9 to 68.2)	-16 (-36.5 to 22.4)	-13.1 (-34.8 to 35.1)	-19.1 (-41.3 to 22.8)
		Lead exposure	30.5 (19.3 to 44.2)	24.4 (14.2 to 37.2)	36.6 (23.9 to 54)	24 (14.8 to 34.9)	19.9 (10.9 to 30.5)	28 (18.1 to 40.9)	-21.3 (-35.9 to -5.6)	-18.6 (-38.4 to -2.3)	-23.4 (-39.1 to -2.6)
		Diet high in sodium	9.7 (1.9 to 39.4)	6.8 (2.2 to 26.2)	12.6 (1.5 to 51.9)	8.4 (1.7 to 32.8)	6 (1.9 to 23.3)	10.7 (1.3 to 42.9)	-13.4 (-33 to 16.5)	-11.5 (-39.7 to 26.9)	-15.1 (-35.2 to 24.9)
		High fasting plasma glucose	240.5 (189.6 to 292.9)	233.6 (184.8 to 297.4)	248.2 (193.4 to 317)	206.6 (159.8 to 262)	207.6 (156.8 to 260.2)	205.7 (159.5 to 276.3)	-14.1 (-31.1 to 3.3)	-11.1 (-34 to 6.8)	-17.1 (-36 to 7)
		High body-mass index	216.6 (130.1 to 316)	249.7 (157.8 to 350.3)	184.1 (101.9 to 287.8)	244.5 (159.5 to 329.9)	269.4 (179.2 to 363.9)	220.2 (134.5 to 316.4)	12.9 (-11.5 to 44.4)	7.9 (-20.2 to 35.2)	19.6 (-10.5 to 69.9)
		High systolic blood pressure	396.3 (341.9 to 487.4)	387.6 (328.9 to 507)	406.5 (345 to 512.6)	350.1 (290.3 to 419.4)	355.7 (282.2 to 429.2)	344.5 (283.9 to 442.5)	-11.7 (-29.1 to 5.5)	-8.2 (-31.4 to 9.6)	-15.2 (-34.5 to 10.1)
		Kidney dysfunction	731.4 (665.2 to 840.8)	730.5 (657 to 873.7)	735.1 (660.4 to 852.8)	590.3 (502.9 to 694.7)	602.8 (494.7 to 698.9)	578.4 (494.5 to 722.5)	-19.3 (-32.7 to -4.3)	-17.5 (-34.2 to -2.8)	-21.3 (-36.7 to 0.2)
	YLDs	Lead exposure	3.7 (2.1 to 6)	3.7 (1.9 to 6.1)	3.7 (2.2 to 5.8)	4.7 (2.6 to 7.8)	4 (2 to 6.9)	5.5 (3 to 8.8)	27.8 (16.4 to 39.3)	9 (-1.5 to 18.1)	46.1 (31.5 to 60.8)
		Diet high in sodium	1.2 (0.2 to 4.7)	1.1 (0.3 to 4)	1.4 (0.2 to 5.4)	1.9 (0.4 to 7.4)	1.4 (0.4 to 5.5)	2.4 (0.3 to 9.6)	58.7 (28.9 to 84.4)	33.5 (7.6 to 69.7)	77.7 (50.8 to 128.3)
		High fasting plasma glucose	21 (15.2 to 28.5)	22.3 (16 to 29.9)	19.7 (14.1 to 27.3)	33.4 (23.6 to 46)	31.7 (22.7 to 42.7)	35 (24.1 to 49.6)	59 (50.3 to 68.5)	42.5 (34 to 51.6)	77.5 (66.1 to 89.6)

Location	Measure	Risk factor	Attributed age-standardized rate (per 100,000)						% Change (1990 to 2019)		
			1990			2019					
			Both	Female	Male	Both	Female	Male	Both	Female	Male
		High body-mass index	28.8 (15.9 to 45.7)	38 (21.6 to 59.7)	19.7 (9.9 to 32.3)	55.6 (33.1 to 84.8)	62.1 (37.7 to 94)	49.5 (27.9 to 77.3)	93.3 (71.1 to 126.5)	63.5 (46.8 to 86.7)	151.5 (112.4 to 226.1)
		High systolic blood pressure	47 (32.9 to 62.8)	54.3 (38 to 72.9)	39.6 (27.7 to 53.7)	74.6 (52 to 101)	76.8 (54 to 102.6)	72.5 (49.6 to 100.1)	58.6 (49 to 68.7)	41.3 (31.5 to 51.3)	83.3 (71.6 to 94.7)
		Kidney dysfunction	103.5 (76.1 to 135.6)	123.1 (90.8 to 162)	84.1 (60.9 to 111.5)	154 (113.1 to 202.3)	167.3 (123.8 to 215.9)	141.9 (101.3 to 191.7)	48.8 (41.3 to 56.6)	35.8 (28.8 to 42.9)	68.8 (59 to 78.4)
Afghanistan	Deaths	Non-optimal temperature	4.6 (1.5 to 8.4)	4.4 (1.5 to 8.2)	4.6 (1.6 to 8.7)	4.2 (1.7 to 7.7)	4.2 (1.6 to 8.1)	4.1 (1.5 to 7.7)	-8.7 (-37.7 to 28)	-5.1 (-41.3 to 39.2)	-11.5 (-37.5 to 24.3)
		Lead exposure	4.9 (3.2 to 7.8)	3.9 (2.4 to 6.8)	5.8 (3.7 to 9.6)	5.3 (3.6 to 8.6)	4.5 (2.8 to 7.9)	6.2 (3.9 to 10.4)	9.3 (-16.4 to 39.7)	16.7 (-22.4 to 59.5)	7 (-19.6 to 38.3)
		Diet high in sodium	0.8 (0.1 to 3.5)	0.6 (0.1 to 2.3)	1.1 (0.1 to 4.6)	0.7 (0.1 to 2.8)	0.5 (0.1 to 2.1)	0.9 (0.1 to 3.9)	-14.3 (-48.7 to 69.4)	-9.4 (-60.3 to 110.6)	-13.3 (-52.6 to 91)
		High fasting plasma glucose	19.9 (13.8 to 30.2)	19.1 (13 to 31.8)	20.4 (13.7 to 31.6)	18 (12.5 to 27.6)	18.1 (11.8 to 30.6)	17.8 (11.5 to 28.2)	-9.6 (-31.5 to 16.8)	-5.3 (-38 to 32)	-12.7 (-34.7 to 12.8)
		High body-mass index	10.6 (4.7 to 19.8)	13.2 (6.4 to 24.4)	8.1 (2.8 to 17.6)	15.1 (8.4 to 26.4)	17.4 (9.6 to 31.4)	12.6 (6 to 22.9)	42.9 (-2.6 to 126.9)	31.5 (-18.9 to 111)	54.8 (2.6 to 189.7)
		High systolic blood pressure	32.7 (23.5 to 49.8)	31.3 (21.6 to 52.9)	33.9 (23.3 to 52.4)	29.9 (21.8 to 46)	29.8 (20.1 to 50.1)	29.8 (19.9 to 47.7)	-8.8 (-31.1 to 16.5)	-4.8 (-38.1 to 33)	-12.2 (-34 to 13)
		Kidney dysfunction	55.9 (42 to 81.6)	54.6 (39.8 to 87.8)	56.7 (40.4 to 83.7)	49.4 (37.1 to 74.8)	50.1 (35.2 to 84.9)	48.5 (34.1 to 75.1)	-11.7 (-33.2 to 12.5)	-8.3 (-38.8 to 24.9)	-14.4 (-35 to 8.2)
	DALYs	Non-optimal temperature	109 (39.2 to 195.1)	114.8 (40.2 to 208.1)	101.6 (35.7 to 182)	91.1 (36.6 to 162.2)	97.3 (36.7 to 188.1)	84.8 (32.7 to 157.3)	-16.5 (-43.4 to 20.1)	-15.3 (-48.7 to 28.5)	-16.6 (-42.6 to 20.6)
		Lead exposure	107.5 (69.9 to 162.4)	91.5 (57.8 to 148.9)	121.2 (78.3 to 187.4)	109.6 (75.1 to 163.8)	97.6 (63 to 151.3)	122.8 (79 to 191.6)	2 (-22.8 to 30.3)	6.7 (-27.5 to 44.6)	1.3 (-24.4 to 30.6)

Location	Measure	Risk factor	Attributed age-standardized rate (per 100,000)						% Change (1990 to 2019)		
			1990			2019					
			Both	Female	Male	Both	Female	Male	Both	Female	Male
		Diet high in sodium	19 (2.8 to 79.4)	14.2 (3.1 to 58.5)	23.3 (2.2 to 99.5)	16.6 (2.6 to 64.6)	12.9 (2.9 to 50.1)	20.7 (2 to 82.8)	-12.7 (-48.6 to 70.6)	-9.3 (-57.7 to 103.3)	-11 (-48.1 to 72.4)
		High fasting plasma glucose	475.5 (339.8 to 668)	488.6 (343 to 733.5)	454.9 (311.6 to 666.5)	422.5 (301.1 to 594.4)	446.9 (304.6 to 691.4)	398.1 (270.5 to 582.5)	-11.1 (-33.8 to 16.8)	-8.5 (-39 to 26.7)	-12.5 (-36.6 to 16.7)
		High body-mass index	273.8 (125.6 to 484.1)	354.9 (180.3 to 622.8)	195.1 (68.4 to 389.2)	381.6 (226.6 to 619.1)	454.1 (266.1 to 758.3)	302.8 (155.1 to 511.3)	39.4 (-4.1 to 117.3)	27.9 (-18 to 97.8)	55.2 (4.3 to 190.9)
		High systolic blood pressure	700.3 (514.9 to 981.1)	716.5 (506.5 to 1065.8)	677.4 (477.4 to 978.2)	636.5 (481.7 to 896.7)	667.7 (466.1 to 1067.3)	603.3 (422.5 to 900.2)	-9.1 (-32.4 to 17.2)	-6.8 (-38.9 to 31.2)	-10.9 (-33.8 to 15.4)
		Kidney dysfunction	1436.9 (1121.2 to 1871.3)	1536.3 (1177.5 to 2125.2)	1316.1 (987.6 to 1747.7)	1230.4 (958.4 to 1680.5)	1332.8 (1004.5 to 2027.8)	1127.8 (838.6 to 1582)	-14.4 (-34.6 to 8.7)	-13.2 (-39.8 to 14.4)	-14.3 (-34.3 to 10)
	YLLs	Non-optimal temperature	109 (39.2 to 195.1)	114.8 (40.2 to 208.1)	101.6 (35.7 to 182)	91.1 (36.6 to 162.2)	97.3 (36.7 to 188.1)	84.8 (32.7 to 157.3)	-16.5 (-43.4 to 20.1)	-15.3 (-48.7 to 28.5)	-16.6 (-42.6 to 20.6)
		Lead exposure	100.7 (65.2 to 153.3)	84.2 (51.7 to 139.8)	114.9 (73.4 to 180.7)	97.8 (65.8 to 152.3)	86.2 (54.3 to 139.2)	110.4 (68.7 to 178.6)	-2.9 (-28.2 to 27.9)	2.4 (-33.9 to 43.9)	-3.9 (-30.2 to 27.3)
		Diet high in sodium	17.8 (2.6 to 74.8)	13 (2.8 to 54.3)	22 (2 to 94.9)	14.7 (2.4 to 57.9)	11.3 (2.4 to 43.6)	18.6 (1.8 to 75.3)	-17.1 (-51.8 to 62.7)	-13.3 (-61.6 to 97.2)	-15.7 (-52.5 to 65.8)
		High fasting plasma glucose	455.6 (322.8 to 648)	466.4 (323 to 709.3)	437.4 (294.7 to 648.7)	392.5 (275.3 to 564.3)	416.2 (274.1 to 659.7)	368.5 (242.2 to 554.9)	-13.9 (-37.2 to 15)	-10.8 (-42.6 to 25.9)	-15.8 (-39.5 to 15)
		High body-mass index	255.2 (116.3 to 455.9)	327.6 (163.4 to 576.5)	185.2 (64.5 to 370.3)	340.5 (196.9 to 566.9)	402.2 (228.1 to 693.4)	272.9 (137.2 to 470.9)	33.4 (-10.9 to 114.4)	22.8 (-24.6 to 94.9)	47.4 (-4.1 to 181.2)
		High systolic blood pressure	657 (476.6 to 934.1)	663.4 (463.2 to 1017.1)	644 (447.3 to 942.5)	570 (416.2 to 829.8)	593.3 (397 to 996.2)	544.5 (367.3 to 831.6)	-13.3 (-37.1 to 14.2)	-10.6 (-43.6 to 28.8)	-15.5 (-38 to 11.7)
		Kidney dysfunction	1333.6 (1024.7 to 1745.7)	1404.8 (1058.3 to 1992.5)	1242.8 (920.2 to 1681.5)	1077.9 (821.6 to 1523.5)	1150.7 (830.6 to 1851)	1004.1 (721.9 to 1464)	-19.2 (-39.9 to 4.7)	-18.1 (-45.3 to 12.3)	-19.2 (-39.8 to 6.8)

Location	Measure	Risk factor	Attributed age-standardized rate (per 100,000)						% Change (1990 to 2019)		
			1990			2019					
			Both	Female	Male	Both	Female	Male	Both	Female	Male
	YLDs	Lead exposure	6.9 (4.3 to 10.2)	7.3 (4.4 to 11)	6.3 (4 to 9.5)	11.9 (7.6 to 17.4)	11.4 (7 to 16.6)	12.4 (8 to 18.4)	73.1 (54.9 to 93.5)	55.7 (37.2 to 76.8)	96.5 (68.9 to 126.5)
		Diet high in sodium	1.2 (0.2 to 4.9)	1.2 (0.3 to 4.7)	1.2 (0.1 to 5)	1.8 (0.3 to 7.5)	1.6 (0.4 to 6.2)	2.1 (0.2 to 8.6)	51.8 (-2.2 to 145.7)	34.6 (-25.4 to 160.8)	73.1 (11.7 to 212.5)
		High fasting plasma glucose	19.9 (13.9 to 27.1)	22.2 (15.4 to 30)	17.5 (12 to 24.9)	30.1 (21 to 42)	30.7 (21.2 to 42.3)	29.7 (19.9 to 42.5)	51.5 (37.1 to 67.4)	38.6 (21.2 to 57.4)	69.9 (46.5 to 97.2)
		High body-mass index	18.6 (8.2 to 33.6)	27.3 (12.9 to 47.1)	10 (3.4 to 20)	41.1 (22.6 to 65.3)	51.9 (29.3 to 82.9)	29.9 (14.9 to 50.8)	121 (78 to 217.6)	89.8 (52.9 to 164.9)	200.1 (119.7 to 428.4)
		High systolic blood pressure	43.3 (29.9 to 58.8)	53.1 (35.9 to 73.2)	33.3 (23.2 to 47)	66.6 (46.7 to 91.7)	74.4 (50.1 to 101.7)	58.8 (39.9 to 84)	53.8 (34.6 to 74.5)	40.1 (16.7 to 68.3)	76.3 (49.4 to 103.2)
		Kidney dysfunction	103.3 (75.8 to 135.4)	131.5 (96.2 to 172.8)	73.3 (52.2 to 98.7)	152.5 (111.8 to 202.4)	182.1 (133.7 to 237)	123.6 (87.1 to 170.7)	47.5 (36.7 to 59.1)	38.5 (26.8 to 51.1)	68.6 (51 to 88.1)
Algeria	Deaths	Non-optimal temperature	2.8 (-0.1 to 5.3)	3 (-0.1 to 6.1)	2.6 (-0.1 to 5.1)	2.5 (0.3 to 4.6)	3 (0.3 to 5.8)	2.1 (0.2 to 3.9)	-9.7 (-58.4 to 92.2)	2.2 (-46.9 to 120.6)	-18.9 (-58 to 77.9)
		Lead exposure	1.3 (0.7 to 2.2)	1.2 (0.5 to 2.5)	1.4 (0.8 to 2.4)	1.1 (0.6 to 1.8)	1.1 (0.5 to 2.1)	1.1 (0.6 to 1.8)	-17.7 (-36.5 to 7.8)	-5.7 (-32.5 to 31.2)	-24.5 (-44.5 to 6.1)
		Diet high in sodium	0.6 (0.1 to 2.4)	0.4 (0.1 to 1.8)	0.7 (0.1 to 3)	0.4 (0.1 to 1.7)	0.4 (0.1 to 1.4)	0.5 (0.1 to 2.1)	-21.8 (-52.5 to 51.7)	-13.3 (-55 to 77.4)	-26.3 (-63.1 to 79.8)
		High fasting plasma glucose	12.6 (9.1 to 18.5)	13.1 (8.6 to 23)	12.2 (8.9 to 16.7)	10.4 (7.4 to 14.3)	12.1 (8.4 to 18.4)	9.1 (6.4 to 12.9)	-17.5 (-37.2 to 9.9)	-7.6 (-32.5 to 26.7)	-25.4 (-45.4 to 4.4)
		High body-mass index	9.8 (4.6 to 17.4)	12.5 (6 to 24.2)	7.4 (3.2 to 13.7)	11.6 (6.1 to 18.3)	15.2 (7.8 to 25.5)	8.9 (4.5 to 14.7)	19.2 (-14.2 to 80.3)	21.8 (-16.5 to 82)	20.4 (-21.6 to 110.5)
		High systolic blood pressure	25.9 (19.6 to 35.7)	27.7 (19.4 to 46)	24.5 (18.6 to 32.5)	21.1 (16.2 to 28)	25.5 (18.8 to 36.8)	18 (13.5 to 24.4)	-18.6 (-36.2 to 6.9)	-8.1 (-32.3 to 25.5)	-26.4 (-45.2 to 3.4)

Location	Measure	Risk factor	Attributed age-standardized rate (per 100,000)						% Change (1990 to 2019)		
			1990			2019					
			Both	Female	Male	Both	Female	Male	Both	Female	Male
		Kidney dysfunction	38.7 (30.3 to 52.1)	41.6 (29.9 to 66.9)	36.5 (28.6 to 47.4)	31.6 (24.8 to 41.2)	38.3 (28.6 to 54.3)	26.8 (20.5 to 35.7)	-18.5 (-36.6 to 5.9)	-8 (-31.6 to 23.1)	-26.6 (-45.4 to 1.9)
	DALYs	Non-optimal temperature	52.6 (-1.1 to 102.3)	56.9 (-1.2 to 123.1)	48.7 (-1.1 to 95.7)	43 (4.8 to 79.7)	50.4 (5.3 to 95.7)	36.9 (4.7 to 66.8)	-18.2 (-61.8 to 72.7)	-11.5 (-55.4 to 90.4)	-24.3 (-60.2 to 68.8)
		Lead exposure	24 (11.7 to 40.8)	22 (8.6 to 44.1)	26 (14.2 to 42.1)	17.9 (8.7 to 30.7)	17.5 (6.9 to 32.3)	18.7 (10 to 31)	-25.2 (-42.6 to -3.7)	-20.8 (-42.7 to 6.1)	-28 (-47.3 to -3)
		Diet high in sodium	11.3 (1.8 to 47.2)	8.9 (2 to 36.7)	13.6 (1.3 to 58.1)	9.4 (1.6 to 36.7)	7.7 (1.9 to 29.1)	11.1 (1.1 to 45.4)	-16.3 (-48.6 to 44.2)	-13.2 (-53.8 to 76.1)	-18.6 (-52.6 to 62.4)
		High fasting plasma glucose	257.5 (187.6 to 373.3)	273.9 (188.5 to 455.6)	241.8 (179.7 to 327.6)	217.4 (162.1 to 291.7)	244.5 (175.9 to 350.2)	193.8 (141 to 267.6)	-15.5 (-36.1 to 10.6)	-10.7 (-35.5 to 21.2)	-19.9 (-40.9 to 9.5)
		High body-mass index	230.7 (126.4 to 382.1)	297.3 (164 to 532.6)	165.5 (78.7 to 290.9)	271.1 (167.7 to 393.7)	336.7 (209.7 to 502.5)	212.6 (123 to 317.1)	17.5 (-13.4 to 66.4)	13.3 (-18.1 to 60.3)	28.4 (-12.8 to 110.3)
		High systolic blood pressure	467.3 (355.2 to 643)	508.5 (363.9 to 802.9)	430.4 (332.2 to 571.5)	389.1 (300.9 to 505.1)	446.2 (336.4 to 616.6)	342.8 (262.6 to 463.2)	-16.7 (-34.4 to 6.6)	-12.2 (-34.2 to 17)	-20.4 (-39.9 to 7.3)
		Kidney dysfunction	839.7 (668.6 to 1130.1)	928.6 (701.2 to 1448.4)	757.9 (606.7 to 965.9)	701.5 (568 to 889.9)	814.7 (634.2 to 1082.3)	604.5 (475.1 to 793.4)	-16.5 (-34 to 4.9)	-12.3 (-34.4 to 12.1)	-20.2 (-38.9 to 5.9)
	YLLs	Non-optimal temperature	52.6 (-1.1 to 102.3)	56.9 (-1.2 to 123.1)	48.7 (-1.1 to 95.7)	43 (4.8 to 79.7)	50.4 (5.3 to 95.7)	36.9 (4.7 to 66.8)	-18.2 (-61.8 to 72.7)	-11.5 (-55.4 to 90.4)	-24.3 (-60.2 to 68.8)
		Lead exposure	21.5 (10.4 to 37.3)	19.3 (7.4 to 40.6)	23.6 (12.6 to 38.9)	14.7 (7.1 to 25.7)	14.6 (5.8 to 27.7)	15.1 (7.9 to 25.5)	-31.5 (-49 to -8.1)	-24.4 (-47 to 5)	-36.2 (-54.2 to -10)
		Diet high in sodium	10 (1.6 to 42.6)	7.7 (1.7 to 32.9)	12.3 (1.2 to 52.4)	7.4 (1.3 to 29.3)	6.2 (1.5 to 23.7)	8.6 (0.8 to 36.3)	-25.8 (-54.8 to 34.3)	-20.1 (-57.9 to 62.3)	-29.6 (-60.5 to 45.9)
		High fasting plasma glucose	237.2 (167.5 to 352)	251.4 (166.5 to 430.8)	223.9 (163 to 308.8)	184.4 (131.6 to 256.1)	212 (142.8 to 315.8)	160.3 (110.1 to 229.4)	-22.3 (-43.3 to 4.7)	-15.7 (-40.3 to 17.9)	-28.4 (-49 to 1.3)

Location	Measure	Risk factor	Attributed age-standardized rate (per 100,000)						% Change (1990 to 2019)		
			1990			2019					
			Both	Female	Male	Both	Female	Male	Both	Female	Male
		High body-mass index	201.8 (106.8 to 343.3)	256.4 (137.9 to 478.5)	148.7 (69.1 to 264.1)	214.3 (130.3 to 317.2)	269.7 (163.6 to 415.5)	165.8 (93 to 261.2)	6.2 (-24.8 to 56.6)	5.2 (-27.4 to 56.7)	11.5 (-26.9 to 92.2)
		High systolic blood pressure	418.6 (310.6 to 594.1)	448.7 (312.5 to 738.8)	392.1 (297.5 to 537)	316.1 (240.1 to 424.4)	368.8 (266.6 to 535.5)	274.1 (203.1 to 384.7)	-24.5 (-42.7 to 0.5)	-17.8 (-40 to 13.4)	-30.1 (-49.5 to -1.8)
		Kidney dysfunction	736.5 (573.9 to 1018.1)	800.2 (580.4 to 1314.5)	679 (529.7 to 884.8)	544.3 (424.3 to 720.4)	638.2 (471.6 to 900.5)	465.8 (353.7 to 641.7)	-26.1 (-43.8 to -3.3)	-20.2 (-42.3 to 7.1)	-31.4 (-50 to -3.1)
	YLDs	Lead exposure	2.5 (1.1 to 4.4)	2.7 (1 to 5)	2.4 (1.2 to 4)	3.3 (1.4 to 6)	2.8 (1 to 5.5)	3.7 (1.8 to 6.5)	28 (9.3 to 46.1)	4.7 (-16.4 to 22.2)	52 (24.7 to 81.7)
		Diet high in sodium	1.3 (0.2 to 5)	1.2 (0.3 to 4.6)	1.3 (0.1 to 5.3)	2 (0.3 to 7.7)	1.6 (0.4 to 6.1)	2.4 (0.2 to 9.6)	59.1 (-2.7 to 157.9)	31.4 (-29.5 to 162.9)	81.6 (15.9 to 234.7)
		High fasting plasma glucose	20.2 (14.3 to 28.2)	22.5 (15.9 to 31.2)	18 (11.9 to 26.1)	33 (22.8 to 46.9)	32.5 (22.9 to 45.8)	33.5 (22.6 to 49.3)	63.2 (45.8 to 81.6)	44.3 (24 to 66)	86.3 (58.7 to 117.3)
		High body-mass index	28.9 (15.3 to 46.5)	40.9 (23 to 64.9)	16.8 (7.6 to 29.3)	56.7 (33 to 88.2)	67 (40.2 to 101.6)	46.8 (26.1 to 75.3)	96.6 (68.1 to 142.7)	63.9 (37.6 to 98.7)	177.9 (113.5 to 328.2)
		High systolic blood pressure	48.7 (33.6 to 66.2)	59.7 (40.5 to 82.7)	38.4 (26.4 to 52.8)	73 (50.6 to 100.9)	77.4 (53.4 to 106.8)	68.7 (46.7 to 97.8)	49.7 (32 to 69.4)	29.7 (7.6 to 53.7)	79.1 (53.9 to 109.2)
		Kidney dysfunction	103.1 (75.4 to 137.4)	128.4 (94.1 to 172.2)	79 (56.3 to 104.7)	157.3 (113.6 to 209.6)	176.5 (129.9 to 232.7)	138.6 (96.9 to 188.9)	52.5 (39.5 to 66.2)	37.4 (23.3 to 51.9)	75.6 (55.9 to 98.1)
Bahrain	Deaths	Non-optimal temperature	2.5 (-1.9 to 6)	2.6 (-2 to 6.1)	2.5 (-1.8 to 6.1)	2.5 (-0.6 to 5.4)	2.5 (-0.7 to 5.4)	2.5 (-0.7 to 5.4)	-0.5 (-197 to 81.2)	-1.3 (-191.6 to 84)	-0.6 (-196.8 to 85)
		Lead exposure	0.9 (0.4 to 1.6)	0.8 (0.2 to 1.5)	1.1 (0.5 to 1.9)	0.9 (0.4 to 1.5)	0.7 (0.2 to 1.4)	1 (0.5 to 1.8)	-6.6 (-28.2 to 19.6)	-9.9 (-36 to 22.2)	-6.7 (-30.1 to 22.4)
		Diet high in sodium	0.6 (0.1 to 2.3)	0.4 (0.1 to 1.7)	0.7 (0.1 to 3.1)	0.5 (0.1 to 2.1)	0.4 (0.1 to 1.5)	0.7 (0.1 to 2.8)	-9.6 (-47 to 54.7)	-14.6 (-57.6 to 74.5)	-10.5 (-55.5 to 107.6)

Location	Measure	Risk factor	Attributed age-standardized rate (per 100,000)						% Change (1990 to 2019)		
			1990			2019					
			Both	Female	Male	Both	Female	Male	Both	Female	Male
		High fasting plasma glucose	14 (10.8 to 18)	14 (10.3 to 18.1)	14.2 (10.6 to 20)	12.6 (9.5 to 16.3)	12.5 (9.1 to 16.1)	12.9 (9.4 to 17.3)	-9.6 (-30 to 14.2)	-11 (-35.1 to 15.9)	-9.4 (-31.5 to 17.8)
		High body-mass index	14.4 (8.4 to 22)	16.6 (10.1 to 24.3)	12.3 (6.3 to 21.5)	14.4 (7.8 to 22.2)	15.8 (8.5 to 24)	13.3 (6.7 to 21.5)	0 (-26.1 to 31.1)	-4.9 (-34 to 26.8)	7.8 (-24.1 to 49.8)
		High systolic blood pressure	25.7 (20.9 to 32.2)	26.1 (19.7 to 33.1)	25.7 (20.1 to 34.9)	23.7 (18.7 to 29.7)	23.8 (18 to 30)	23.8 (18.4 to 31.7)	-7.5 (-27.8 to 16)	-8.6 (-31.9 to 21.2)	-7.2 (-29.9 to 21.1)
		Kidney dysfunction	38.5 (32 to 46.8)	39 (30.4 to 48.2)	38.6 (31.2 to 51.1)	34.8 (27.9 to 42.2)	35.1 (27.2 to 42.6)	34.8 (27.3 to 44.8)	-9.5 (-29.6 to 13.1)	-10.1 (-34 to 16)	-9.7 (-31.4 to 17.1)
	DALYs	Non-optimal temperature	44.3 (-34.2 to 104.9)	46.4 (-35.8 to 111.1)	43.1 (-32.3 to 102)	39.3 (-9.8 to 85.1)	39.8 (-10.3 to 84.2)	39.3 (-10.8 to 85.2)	-11.3 (-188.6 to 60)	-14.4 (-181.7 to 61.2)	-8.8 (-188.9 to 66.1)
		Lead exposure	17.2 (6.2 to 29.8)	15 (3.6 to 28.3)	19.6 (8.1 to 33.5)	14.3 (5 to 25.6)	11.4 (2.5 to 22.4)	16.9 (7 to 29.4)	-16.9 (-34.5 to 3)	-24.1 (-46.5 to -2.2)	-13.9 (-34.6 to 10.3)
		Diet high in sodium	11.8 (1.8 to 47)	9 (2 to 35.4)	14.5 (1.4 to 60.1)	11 (1.7 to 44.1)	7.5 (1.8 to 29.9)	13.7 (1.4 to 55.8)	-6.4 (-45.8 to 53.5)	-16.4 (-59.2 to 70.5)	-4.9 (-45.7 to 95.2)
		High fasting plasma glucose	274.6 (218.9 to 344.7)	287.5 (216.2 to 368.4)	268.1 (204.4 to 360.6)	242.3 (191 to 304.9)	240 (183.8 to 301.3)	246.6 (187.5 to 316.6)	-11.8 (-30.3 to 10.4)	-16.5 (-37.7 to 8.1)	-8 (-29.4 to 17.4)
		High body-mass index	312.5 (199.3 to 438.2)	374.9 (246.9 to 516.1)	260.2 (151.1 to 407.1)	311.6 (200 to 438.3)	341.6 (224 to 470.5)	290.8 (178 to 423.4)	-0.3 (-21.4 to 26.2)	-8.9 (-31.1 to 17.6)	11.8 (-15.6 to 48.6)
		High systolic blood pressure	468.9 (385.1 to 577.9)	499.2 (389.8 to 619)	447.9 (355 to 600.7)	432.7 (350.9 to 529.1)	435.6 (339.8 to 535.8)	433 (344.3 to 548.3)	-7.7 (-25.9 to 14.5)	-12.7 (-33 to 15)	-3.3 (-24.8 to 23.5)
		Kidney dysfunction	781.7 (663.4 to 936.3)	838.3 (673.4 to 1025.7)	743.8 (613.2 to 960.5)	711.9 (588.8 to 844.7)	725.5 (589.9 to 865.6)	707.2 (570.8 to 875.9)	-8.9 (-26.2 to 11)	-13.4 (-32 to 7.9)	-4.9 (-24.9 to 18.3)
	YLLs	Non-optimal temperature	44.3 (-34.2 to 104.9)	46.4 (-35.8 to 111.1)	43.1 (-32.3 to 102)	39.3 (-9.8 to 85.1)	39.8 (-10.3 to 84.2)	39.3 (-10.8 to 85.2)	-11.3 (-188.6 to 60)	-14.4 (-181.7 to 61.2)	-8.8 (-188.9 to 66.1)

Location	Measure	Risk factor	Attributed age-standardized rate (per 100,000)						% Change (1990 to 2019)		
			1990			2019					
			Both	Female	Male	Both	Female	Male	Both	Female	Male
		Lead exposure	15.2 (5.5 to 26.2)	13 (3.1 to 24.5)	17.6 (7.3 to 30.2)	11.7 (4 to 21.1)	9.4 (2.1 to 18.7)	13.7 (5.6 to 23.9)	-23.2 (-40.7 to -2.3)	-27.7 (-50.6 to -3.7)	-21.8 (-41.9 to 4)
		Diet high in sodium	10.3 (1.6 to 41.4)	7.7 (1.7 to 30.8)	12.8 (1.2 to 53.3)	8.5 (1.3 to 34.4)	5.9 (1.3 to 23.9)	10.6 (1 to 43.6)	-17.3 (-54.6 to 36.3)	-23.5 (-64 to 58.9)	-17 (-54.4 to 75.8)
		High fasting plasma glucose	250.3 (193.2 to 319.6)	261.1 (190.3 to 341.1)	245.3 (181.8 to 337.7)	202.7 (151.1 to 261.7)	204 (148.8 to 263.9)	204 (147.7 to 273.2)	-19 (-38 to 3.6)	-21.9 (-43.1 to 4.7)	-16.8 (-37.5 to 9)
		High body-mass index	271.8 (169.6 to 387)	321.2 (207.1 to 450)	230 (130.6 to 365.3)	241.4 (149 to 346.4)	266.8 (171.4 to 381.1)	223.3 (130.6 to 334.4)	-11.2 (-32.4 to 17)	-17 (-41.2 to 12.2)	-2.9 (-29.2 to 35.5)
		High systolic blood pressure	413.5 (332.4 to 519.6)	433.6 (328.6 to 551.6)	401.2 (314.3 to 547.9)	344.8 (268.3 to 431.2)	349.4 (264.6 to 440.4)	343.7 (261.9 to 456.2)	-16.6 (-35.9 to 6.7)	-19.4 (-40.2 to 10.3)	-14.3 (-35.2 to 13.1)
		Kidney dysfunction	669.6 (556.6 to 817.4)	700.7 (543.2 to 865.2)	651.7 (525.9 to 865.5)	540.7 (434.4 to 663.7)	546.9 (423.4 to 669.5)	540.7 (421.9 to 697.7)	-19.2 (-37.2 to 2.2)	-22 (-42.4 to 3)	-17 (-36.2 to 8.4)
	YLDs	Lead exposure	2 (0.6 to 4)	2 (0.4 to 4.2)	2 (0.8 to 3.7)	2.7 (0.8 to 5.4)	2 (0.4 to 4.5)	3.2 (1 to 6.1)	30.7 (8.2 to 53.2)	-0.7 (-27.6 to 19.8)	54.4 (23.6 to 82.9)
		Diet high in sodium	1.5 (0.2 to 5.8)	1.3 (0.3 to 5.3)	1.6 (0.2 to 6.3)	2.5 (0.3 to 9.5)	1.6 (0.4 to 6.3)	3.1 (0.3 to 12.6)	68.8 (-1.6 to 156.1)	25 (-31.2 to 155.9)	90.2 (15.2 to 251.4)
		High fasting plasma glucose	24.3 (16.9 to 33.8)	26.3 (18.5 to 36.6)	22.8 (15.2 to 32.5)	39.6 (26.6 to 57.3)	36 (24.7 to 51.2)	42.6 (27.7 to 63)	63.1 (45.1 to 84.6)	36.6 (18.9 to 58.3)	87 (61.4 to 117.5)
		High body-mass index	40.7 (23.5 to 63)	53.7 (31.2 to 83.2)	30.2 (16.2 to 48.2)	70.2 (41.5 to 107.9)	74.9 (45.4 to 112.5)	67.5 (37.7 to 108.1)	72.7 (49.3 to 102)	39.3 (16.7 to 65.6)	123.3 (89.4 to 172.5)
		High systolic blood pressure	55.4 (38.8 to 75.2)	65.6 (44.7 to 89.1)	46.7 (32 to 65.4)	87.9 (60.9 to 122.5)	86.2 (59.1 to 119.7)	89.3 (60.4 to 127.3)	58.6 (37.8 to 82.7)	31.5 (9.8 to 58.1)	91.1 (64 to 122.3)
		Kidney dysfunction	112.1 (81.8 to 147.9)	137.5 (100 to 181.4)	92.1 (65.8 to 123.6)	171.2 (122.3 to 228.7)	178.7 (129.7 to 235.4)	166.5 (115 to 228.8)	52.7 (37.6 to 68.9)	29.9 (15.4 to 45.3)	80.9 (60 to 103.7)

Location	Measure	Risk factor	Attributed age-standardized rate (per 100,000)						% Change (1990 to 2019)		
			1990			2019					
			Both	Female	Male	Both	Female	Male	Both	Female	Male
Egypt	Deaths	Non-optimal temperature	2 (-0.2 to 3.6)	2.2 (-0.2 to 4.2)	1.7 (-0.2 to 3.1)	2.7 (0.6 to 4.8)	3.5 (0.8 to 6.6)	2.2 (0.5 to 4.2)	34.7 (-92.3 to 145.4)	56.2 (-91.5 to 200.2)	27.1 (-91.9 to 134.3)
		Lead exposure	2.1 (1.2 to 3.1)	1.9 (0.9 to 3.2)	2.3 (1.5 to 3.3)	2.6 (1.5 to 4.1)	2.9 (1.2 to 4.8)	2.5 (1.5 to 4.4)	25.5 (-6.7 to 63.5)	49.8 (-1.4 to 101.2)	11.5 (-24 to 53.5)
		Diet high in sodium	0.6 (0.1 to 2.3)	0.5 (0.1 to 2)	0.6 (0.1 to 2.8)	0.7 (0.1 to 2.7)	0.6 (0.1 to 2.5)	0.7 (0.1 to 2.9)	17.8 (-37.2 to 108.5)	30.5 (-45.1 to 164.2)	8 (-44.8 to 126.6)
		High fasting plasma glucose	13 (8.5 to 17.4)	14.5 (7.7 to 20.8)	11.5 (8.2 to 16.1)	15.6 (9.2 to 23.2)	19.6 (8.7 to 29.5)	13.1 (8 to 22.2)	20.1 (-13 to 59.2)	35.9 (-11.8 to 82.6)	13.6 (-25.9 to 59.5)
		High body-mass index	13 (6.6 to 20.7)	17.1 (8.3 to 27.6)	8.9 (4.4 to 15.1)	19 (9.4 to 30.7)	26.7 (10.8 to 43.5)	14 (6.6 to 25.1)	46.2 (3.1 to 102.9)	56.4 (-5 to 119.3)	57.1 (-3.8 to 157.8)
		High systolic blood pressure	23.8 (15.8 to 30.5)	26.7 (14.6 to 37)	20.7 (15.5 to 27.8)	29 (17.3 to 41)	38.8 (17.6 to 55.7)	23.3 (15 to 38.2)	22.1 (-11 to 59.7)	45.3 (-6.7 to 94.3)	12.2 (-25 to 56.7)
		Kidney dysfunction	39.4 (27.3 to 49.3)	44.5 (25.5 to 58.9)	34.2 (26.9 to 44.8)	43.9 (27 to 61.8)	57.5 (26.8 to 81.2)	36 (23.2 to 58.5)	11.5 (-17 to 44.5)	29.3 (-13.3 to 67.9)	5.2 (-28.3 to 45.7)
	DALYs	Non-optimal temperature	40.6 (-3.7 to 72.8)	46.5 (-4 to 86.8)	34.8 (-3.3 to 63.2)	50.9 (12 to 92.5)	62.1 (13.9 to 119.4)	43.2 (10.4 to 84.8)	25.2 (-92.7 to 130.7)	33.7 (-92.5 to 156.1)	23.9 (-92.3 to 132)
		Lead exposure	43.5 (26.4 to 63.7)	40.9 (20.1 to 64.4)	45.9 (30.2 to 65.1)	50.5 (28.4 to 78.2)	51.7 (22.3 to 85.2)	50.4 (29.2 to 84.5)	16.3 (-12 to 48.3)	26.3 (-13.2 to 64.5)	9.8 (-22.1 to 47.4)
		Diet high in sodium	12.2 (1.8 to 49.6)	10.3 (1.9 to 42.7)	14.1 (1.4 to 57.7)	14.9 (2 to 60)	13 (2.2 to 52.2)	16.6 (1.5 to 65.9)	22.1 (-36.8 to 114.5)	25.4 (-47.7 to 154.5)	18 (-32.6 to 133.6)
		High fasting plasma glucose	284.8 (195.2 to 370.1)	318.2 (182.7 to 440.9)	251.2 (184.1 to 345.2)	345.5 (212.8 to 505.6)	405.1 (200.8 to 590.4)	302.4 (192.9 to 495.6)	21.3 (-11.6 to 58.1)	27.3 (-14.5 to 69.1)	20.4 (-17 to 63)
		High body-mass index	308.4 (176.5 to 464.7)	404.6 (220.4 to 611.5)	211.9 (114.2 to 339.3)	457.5 (256.5 to 692)	595.2 (289.3 to 915.5)	353.8 (187.7 to 599.9)	48.4 (7.6 to 102.4)	47.1 (-3.5 to 99.8)	66.9 (9.7 to 160.2)

Location	Measure	Risk factor	Attributed age-standardized rate (per 100,000)						% Change (1990 to 2019)		
			1990			2019					
			Both	Female	Male	Both	Female	Male	Both	Female	Male
		High systolic blood pressure	466.5 (327.5 to 583.9)	529.4 (308.3 to 715.7)	402.5 (308 to 527.4)	601.5 (382.5 to 832.5)	743.9 (382 to 1050.3)	502.7 (335.2 to 798.3)	28.9 (-4.6 to 67.3)	40.5 (-4.6 to 86.3)	24.9 (-11.6 to 68.5)
		Kidney dysfunction	916.4 (675.8 to 1090.3)	1062.8 (672.7 to 1346.4)	770.4 (626.6 to 985.6)	1009.5 (660.4 to 1386.3)	1218 (664.5 to 1653.1)	862.4 (577.2 to 1319.5)	10.2 (-13.8 to 37.8)	14.6 (-15.7 to 45)	11.9 (-17.6 to 46.9)
	YLLs	Non-optimal temperature	40.6 (-3.7 to 72.8)	46.5 (-4 to 86.8)	34.8 (-3.3 to 63.2)	50.9 (12 to 92.5)	62.1 (13.9 to 119.4)	43.2 (10.4 to 84.8)	25.2 (-92.7 to 130.7)	33.7 (-92.5 to 156.1)	23.9 (-92.3 to 132)
		Lead exposure	38.8 (22.8 to 57.5)	36.1 (16.6 to 58.3)	41.5 (26.8 to 59.8)	43.3 (23.3 to 70.1)	45.3 (18 to 76.3)	42.5 (22.8 to 76.1)	11.5 (-19.9 to 47.2)	25.7 (-19 to 69.3)	2.4 (-32.6 to 43.8)
		Diet high in sodium	10.9 (1.6 to 44.8)	9.1 (1.6 to 38.2)	12.7 (1.2 to 52.7)	12.5 (1.6 to 52.3)	11.1 (1.6 to 45.3)	13.8 (1.2 to 56.8)	15.2 (-39.7 to 106.1)	22.7 (-50 to 153)	8.8 (-39.9 to 122.5)
		High fasting plasma glucose	262.1 (172.6 to 346)	292.5 (156.4 to 413.6)	231.6 (165.3 to 322)	306.7 (175.8 to 464.2)	366.6 (162.3 to 550.5)	263.6 (155.4 to 458.7)	17 (-17.8 to 57.2)	25.3 (-18.8 to 70.6)	13.9 (-26.6 to 60.7)
		High body-mass index	274.4 (152.3 to 416.9)	356.7 (183 to 553.7)	191.8 (101.7 to 312.8)	389.8 (202.4 to 616.4)	513.8 (219.5 to 812.8)	297 (144.5 to 535.7)	42.1 (-2.8 to 99)	44.1 (-11.9 to 101)	54.8 (-7.3 to 148.6)
		High systolic blood pressure	420.6 (283 to 535.1)	473 (258.2 to 652.9)	367.2 (274.8 to 488.7)	516.2 (301.3 to 746.8)	650 (291.2 to 945.7)	424.5 (255.3 to 709.1)	22.7 (-13.5 to 63.9)	37.4 (-13.3 to 88.6)	15.6 (-25.6 to 61.7)
		Kidney dysfunction	807.9 (569.1 to 974.6)	926 (543.9 to 1198.4)	690.2 (545.3 to 902.5)	837 (493.1 to 1200.3)	1023.9 (470.1 to 1463.8)	708.7 (432.2 to 1170.5)	3.6 (-23.6 to 35.4)	10.6 (-25 to 45.2)	2.7 (-30.8 to 42.8)
	YLDs	Lead exposure	4.6 (2.6 to 7.3)	4.9 (2.5 to 8)	4.4 (2.6 to 6.6)	7.2 (4.1 to 11.6)	6.4 (3.4 to 10.6)	7.9 (4.5 to 12.5)	56.8 (39.3 to 77.8)	31.2 (14.7 to 51.9)	79.9 (53.5 to 109)
		Diet high in sodium	1.3 (0.2 to 5.3)	1.3 (0.3 to 5)	1.4 (0.1 to 5.7)	2.4 (0.4 to 9.4)	1.8 (0.4 to 7.2)	2.8 (0.3 to 10.9)	79.4 (-1.6 to 170.3)	44.5 (-30 to 181.1)	103.6 (29.2 to 266.4)
		High fasting plasma glucose	22.7 (16.4 to 31.5)	25.7 (18.3 to 35.5)	19.7 (13.4 to 27.9)	38.8 (26.9 to 54.1)	38.6 (26.8 to 53.4)	38.8 (26.1 to 55.7)	70.8 (54.1 to 89.2)	49.9 (32.2 to 69.6)	97.2 (72.9 to 125.9)

Location	Measure	Risk factor	Attributed age-standardized rate (per 100,000)						% Change (1990 to 2019)		
			1990			2019					
			Both	Female	Male	Both	Female	Male	Both	Female	Male
		High body-mass index	34 (18.6 to 54.7)	47.9 (27.3 to 73.8)	20.1 (9.9 to 34.2)	67.8 (40 to 104.1)	81.3 (49.2 to 121)	56.8 (31.9 to 89.7)	99.3 (73.2 to 143.1)	69.8 (47.2 to 103.6)	182.5 (123.9 to 291.3)
		High systolic blood pressure	46 (32.2 to 62.8)	56.4 (38.8 to 78.9)	35.4 (24.1 to 48.8)	85.3 (58.1 to 116.3)	94 (64.3 to 126.7)	78.2 (52.6 to 109.7)	85.5 (61.3 to 112)	66.5 (40.1 to 99.3)	121.2 (89.2 to 155.2)
		Kidney dysfunction	108.5 (80 to 144.2)	136.9 (99.8 to 181.3)	80.3 (57.9 to 108.1)	172.4 (126.2 to 228.8)	194.1 (142.8 to 254.3)	153.7 (109.5 to 208.7)	59 (45.8 to 73.2)	41.8 (28.1 to 56.5)	91.5 (71 to 113.7)
Iran (Islamic Republic of)	Deaths	Non-optimal temperature	2.1 (1.4 to 2.8)	2 (1.3 to 2.8)	2.2 (1.5 to 3)	1.8 (1.3 to 2.4)	1.8 (1.2 to 2.4)	1.9 (1.3 to 2.5)	-12 (-31.6 to 12.3)	-8.2 (-34.6 to 16.7)	-15.3 (-29.1 to 7.3)
		Lead exposure	1.6 (1.1 to 2.1)	1.2 (0.8 to 1.9)	1.9 (1.4 to 2.6)	1.4 (1 to 1.8)	1.1 (0.7 to 1.5)	1.7 (1.2 to 2.2)	-11.1 (-28.3 to -1)	-9.2 (-40.3 to 6.2)	-13 (-27.2 to 0.8)
		Diet high in sodium	0.4 (0.1 to 1.4)	0.2 (0.1 to 1)	0.5 (0.1 to 2)	0.3 (0.1 to 1.2)	0.2 (0.1 to 0.8)	0.4 (0.1 to 1.5)	-17.6 (-37.9 to 11.8)	-13.8 (-40.9 to 19.2)	-20.3 (-42.5 to 27.9)
		High fasting plasma glucose	8 (6.6 to 9.8)	7.4 (5.7 to 10)	8.7 (7 to 10.5)	6.7 (5.5 to 8)	6.4 (5.1 to 7.6)	7 (5.7 to 8.4)	-16.4 (-32.3 to -7)	-13.1 (-41.2 to 1)	-19.4 (-31 to -6)
		High body-mass index	5.8 (3.2 to 9.4)	6.4 (3.6 to 10.1)	5.2 (2.5 to 8.6)	7.1 (4.1 to 10.2)	7.6 (4.4 to 11)	6.5 (3.7 to 9.7)	20.7 (-3.9 to 57.9)	18.5 (-14.9 to 59.8)	24.2 (-2.7 to 80)
		High systolic blood pressure	14.6 (12.6 to 17.9)	13.7 (11.1 to 19)	15.5 (13 to 18)	12.7 (10.8 to 14.2)	12.4 (10.6 to 14)	12.9 (11 to 14.6)	-13.3 (-32.3 to -4.9)	-9.5 (-42.2 to 4.2)	-16.7 (-29.1 to -3.9)
		Kidney dysfunction	24.1 (21.8 to 28.8)	22.6 (19.6 to 31.5)	25.6 (22.5 to 28.4)	19.8 (17.8 to 21.2)	19.4 (17.3 to 20.9)	20.2 (18 to 22)	-18 (-35.4 to -10.8)	-14.3 (-42.8 to -3.3)	-21.2 (-31.2 to -9)
	DALYs	Non-optimal temperature	43.9 (29.5 to 58.3)	41.1 (27.6 to 56.1)	46.4 (30.9 to 62.3)	33.3 (23.2 to 44)	31.6 (22 to 42)	35.1 (24.2 to 46.5)	-24 (-36.4 to -0.9)	-23.1 (-39.6 to -0.6)	-24.5 (-37.2 to -0.6)
		Lead exposure	34.7 (24.3 to 46.6)	26.9 (17.3 to 39.6)	42.1 (30 to 55.3)	26.6 (18.3 to 35.9)	20.7 (13.2 to 29.1)	32.5 (23.1 to 42.8)	-23.4 (-32.7 to -16)	-23.1 (-40.8 to -13.2)	-22.7 (-32.8 to -11.8)

Location	Measure	Risk factor	Attributed age-standardized rate (per 100,000)						% Change (1990 to 2019)		
			1990			2019					
			Both	Female	Male	Both	Female	Male	Both	Female	Male
		Diet high in sodium	8.6 (1.6 to 32.3)	5.7 (1.7 to 22)	11.5 (1.4 to 43.8)	7.2 (1.4 to 26.5)	4.8 (1.4 to 19.1)	9.6 (1.2 to 35)	-17.1 (-33.8 to 11.1)	-16 (-38.8 to 12.7)	-16.6 (-38.2 to 28.8)
		High fasting plasma glucose	179.4 (149.1 to 209.4)	165.6 (133.7 to 202.6)	192.5 (158.1 to 226.5)	147.6 (123.9 to 171.4)	137.8 (114.9 to 161.1)	157.5 (132.5 to 183.4)	-17.7 (-26.9 to -10.1)	-16.8 (-34.5 to -6.4)	-18.2 (-28.2 to -6.6)
		High body-mass index	148.4 (85.6 to 216.2)	167.6 (103 to 240.8)	130.1 (69.3 to 203.4)	172.9 (113.1 to 234.9)	181.3 (119.7 to 243.5)	164.6 (104.5 to 229.7)	16.5 (-2.2 to 46.5)	8.2 (-13.4 to 36.7)	26.5 (1.1 to 81)
		High systolic blood pressure	302.5 (263.8 to 348.5)	286.6 (240.1 to 356.7)	317 (270 to 364)	264 (228.2 to 298.9)	253.8 (218.5 to 289.5)	274.2 (233.7 to 312.6)	-12.8 (-23.7 to -5.6)	-11.4 (-31.5 to -1.3)	-13.5 (-24.2 to -2.7)
		Kidney dysfunction	620.3 (569.1 to 676.9)	602.1 (537.9 to 694.9)	636 (564.4 to 696)	486.7 (443.9 to 530.5)	475.6 (427.2 to 522.4)	498 (455.2 to 545.9)	-21.5 (-28.8 to -15.4)	-21 (-33.2 to -13.5)	-21.7 (-28.6 to -11.4)
	YLLs	Non-optimal temperature	43.9 (29.5 to 58.3)	41.1 (27.6 to 56.1)	46.4 (30.9 to 62.3)	33.3 (23.2 to 44)	31.6 (22 to 42)	35.1 (24.2 to 46.5)	-24 (-36.4 to -0.9)	-23.1 (-39.6 to -0.6)	-24.5 (-37.2 to -0.6)
		Lead exposure	29.1 (20.2 to 39.3)	21.7 (14 to 31.8)	36.1 (25.6 to 47.4)	21.2 (14.9 to 28.2)	16.2 (10.4 to 22.7)	26.2 (18.5 to 34.6)	-27.1 (-37.7 to -19.1)	-25.2 (-45.9 to -13.8)	-27.5 (-37.8 to -16.2)
		Diet high in sodium	7.2 (1.3 to 27.3)	4.5 (1.3 to 17.7)	9.8 (1.1 to 37.9)	5.5 (1.1 to 20.6)	3.6 (1.1 to 14.5)	7.5 (1 to 27.7)	-23.3 (-40.5 to 3.7)	-20.4 (-44.3 to 9)	-23.6 (-44.1 to 20.2)
		High fasting plasma glucose	157.3 (129.2 to 185.5)	143.3 (112.5 to 179.9)	170.6 (137.9 to 203.5)	122.1 (101.2 to 143.2)	113.8 (92.8 to 135.3)	130.3 (107.4 to 154.4)	-22.4 (-32.4 to -14)	-20.6 (-40.1 to -8.9)	-23.6 (-33.9 to -10.5)
		High body-mass index	121.4 (70.9 to 175.5)	133.2 (80.9 to 194.4)	110.1 (58.1 to 172.4)	132.7 (86.1 to 181.6)	137.2 (90.3 to 183.7)	128.2 (79.9 to 180.4)	9.3 (-9.4 to 38.7)	3 (-19 to 32.2)	16.4 (-7.8 to 67.6)
		High systolic blood pressure	254.7 (221.5 to 296.4)	234.7 (194.9 to 300.3)	273.6 (230.6 to 317.3)	207.6 (179.4 to 232.1)	197.3 (170.1 to 222.6)	217.8 (186.1 to 247.5)	-18.5 (-31.7 to -10.8)	-15.9 (-39.8 to -3.9)	-20.4 (-31.3 to -8.8)
		Kidney dysfunction	507.4 (463.2 to 556.8)	474 (419.7 to 569.5)	538.8 (471.7 to 590.8)	360.5 (333.7 to 382.2)	341.6 (312.5 to 365.7)	379.2 (348.6 to 409.9)	-29 (-36.4 to -22.5)	-27.9 (-42.3 to -18.9)	-29.6 (-36.4 to -18.4)

Location	Measure	Risk factor	Attributed age-standardized rate (per 100,000)						% Change (1990 to 2019)		
			1990			2019					
			Both	Female	Male	Both	Female	Male	Both	Female	Male
	YLDs	Lead exposure	5.6 (3.4 to 8.6)	5.2 (3 to 8.3)	6 (3.7 to 8.8)	5.4 (3.2 to 8.4)	4.5 (2.5 to 7.3)	6.4 (3.8 to 9.8)	-4.1 (-14.3 to 7)	-14.8 (-26.1 to -3.8)	6.8 (-7 to 20.5)
		Diet high in sodium	1.5 (0.3 to 5.4)	1.2 (0.3 to 4.6)	1.7 (0.2 to 6.5)	1.6 (0.3 to 6.1)	1.2 (0.3 to 4.5)	2.1 (0.2 to 7.6)	13.2 (-8.5 to 45.8)	0.8 (-22.4 to 30.9)	23.5 (-8.1 to 85)
		High fasting plasma glucose	22.2 (15.8 to 30)	22.3 (15.9 to 30.1)	21.9 (15.4 to 30.3)	25.6 (18.1 to 35.2)	24 (17.1 to 32.4)	27.2 (18.8 to 37.9)	15.4 (8.5 to 22.3)	7.7 (-0.2 to 16.2)	23.9 (15.7 to 32.6)
		High body-mass index	27 (14.3 to 44.1)	34.4 (18.7 to 55.4)	20 (9.4 to 33.3)	40.2 (23.4 to 62.7)	44.1 (26 to 68.6)	36.3 (20.3 to 57.1)	48.5 (25 to 88.2)	28.3 (6.7 to 62.9)	81.9 (46.8 to 160.5)
		High systolic blood pressure	47.8 (33.9 to 64.3)	51.9 (36.2 to 70.2)	43.4 (30.4 to 59.2)	56.4 (39.9 to 76.2)	56.5 (39.7 to 76.3)	56.4 (39 to 77.1)	17.9 (9.2 to 26.8)	8.9 (-1.1 to 19.1)	29.8 (19.3 to 39.5)
		Kidney dysfunction	112.9 (82.7 to 148.1)	128.1 (94 to 168.2)	97.2 (70.7 to 127.7)	126.2 (91.5 to 165.7)	134 (97.5 to 176.3)	118.8 (85.2 to 158.8)	11.8 (4.9 to 18.9)	4.7 (-3.7 to 12.8)	22.2 (13.5 to 30.2)
Iraq	Deaths	Non-optimal temperature	3.2 (0.8 to 5.6)	3.1 (0.8 to 5.8)	3.4 (0.8 to 6.1)	3.4 (1.1 to 5.7)	2.9 (0.9 to 4.9)	4.1 (1.2 to 7.3)	6.2 (-25.7 to 98.4)	-6.9 (-38.9 to 73.9)	20.8 (-19.6 to 147)
		Lead exposure	1.7 (0.9 to 2.8)	1.3 (0.6 to 2.4)	2.2 (1.2 to 3.5)	1.7 (0.9 to 2.7)	1.1 (0.5 to 1.8)	2.4 (1.3 to 4.1)	-0.9 (-22.1 to 24.2)	-15.5 (-39.5 to 16)	11.1 (-17 to 48.5)
		Diet high in sodium	0.7 (0.1 to 3)	0.5 (0.1 to 2.1)	1 (0.1 to 4.2)	0.7 (0.1 to 2.7)	0.4 (0.1 to 1.6)	1 (0.1 to 4.4)	-4.4 (-46.5 to 69.2)	-20.9 (-63.1 to 99.4)	6.3 (-44.7 to 139.1)
		High fasting plasma glucose	17 (12.8 to 23.6)	16.2 (11.3 to 24)	18.1 (13.2 to 26)	15.5 (11.5 to 20.6)	12.9 (9.2 to 17.5)	18.8 (13.1 to 26.7)	-8.8 (-30.8 to 15)	-20.3 (-44.3 to 11.5)	3.5 (-24.6 to 35.3)
		High body-mass index	17.8 (9.9 to 28)	18.5 (10.3 to 31.4)	17 (8.6 to 29.3)	17.2 (9.6 to 25.6)	15.3 (8.8 to 23)	19.5 (9.9 to 31.9)	-3.5 (-29.3 to 28.2)	-17.6 (-43.7 to 18.6)	14.3 (-21.8 to 62.4)
		High systolic blood pressure	30.5 (23.7 to 41.6)	29 (20.8 to 43.7)	32.4 (24.1 to 47.2)	29.6 (22.7 to 38.3)	24.4 (18.7 to 31.9)	35.9 (25.8 to 52.3)	-3 (-26.4 to 20.3)	-15.7 (-40.3 to 16.4)	11 (-17.8 to 45.8)

Location	Measure	Risk factor	Attributed age-standardized rate (per 100,000)						% Change (1990 to 2019)		
			1990			2019					
			Both	Female	Male	Both	Female	Male	Both	Female	Male
		Kidney dysfunction	46.3 (37.1 to 61.9)	44.6 (33.5 to 66.3)	48.4 (37.3 to 68.8)	41.9 (33.2 to 53.5)	35.3 (27.6 to 45.8)	49.9 (36.9 to 72.5)	-9.5 (-30.1 to 11.5)	-20.8 (-43.5 to 7.8)	3.1 (-24 to 35.6)
	DALYs	Non-optimal temperature	69.1 (17.9 to 120)	69 (17.5 to 125)	69.5 (17.4 to 122.1)	65.2 (20.9 to 111)	57.1 (17.2 to 99.1)	74.5 (23 to 129)	-5.5 (-37.4 to 77.8)	-17.2 (-45.8 to 56.9)	7.2 (-28.9 to 117.3)
		Lead exposure	34.4 (18.1 to 54.9)	26.9 (10.9 to 49.4)	42.6 (23.2 to 67.2)	30.6 (15.9 to 50)	20.6 (8.7 to 36.2)	42 (22.9 to 67.4)	-11 (-29.6 to 9.9)	-23.3 (-43.6 to 3.2)	-1.3 (-25.9 to 29.9)
		Diet high in sodium	16.4 (2.4 to 67.3)	11.7 (2.4 to 49.2)	21.3 (1.9 to 89.6)	15.7 (2.3 to 61.3)	9.5 (2.1 to 37.2)	22.4 (2.1 to 89.6)	-4.3 (-42.9 to 67)	-19.3 (-60.4 to 96.4)	5 (-38.3 to 102.5)
		High fasting plasma glucose	388.9 (297.1 to 512.1)	384.2 (274.9 to 554.9)	395.9 (295.3 to 525.5)	345.9 (264.2 to 446.9)	301.4 (221.7 to 402.3)	396.1 (287.6 to 531.6)	-11.1 (-32.1 to 13.4)	-21.5 (-45 to 9.7)	0 (-26.9 to 33)
		High body-mass index	433.7 (252.3 to 638.4)	461.8 (272.7 to 725.4)	404.9 (223.6 to 642.2)	411.8 (250.3 to 589.1)	383.2 (237.2 to 553.7)	444 (255.6 to 665.9)	-5 (-28.4 to 23)	-17 (-41.4 to 15.5)	9.6 (-21.5 to 51.5)
		High systolic blood pressure	642.6 (504.4 to 832.2)	639.7 (471.5 to 904.9)	648.9 (491.6 to 874.7)	622.5 (485.8 to 784.2)	545.9 (415.1 to 704.4)	710.6 (532.4 to 926.8)	-3.1 (-24.5 to 20.4)	-14.7 (-36.6 to 14.8)	9.5 (-16.9 to 42.2)
		Kidney dysfunction	1126.8 (933.9 to 1407.7)	1154.2 (904.1 to 1603.1)	1104.9 (875.4 to 1414.9)	991.2 (797.3 to 1215)	905.4 (709.6 to 1134)	1091.9 (833.6 to 1403.2)	-12 (-30.2 to 8.2)	-21.6 (-41.4 to 3.2)	-1.2 (-24.6 to 27.1)
	YLLs	Non-optimal temperature	69.1 (17.9 to 120)	69 (17.5 to 125)	69.5 (17.4 to 122.1)	65.2 (20.9 to 111)	57.1 (17.2 to 99.1)	74.5 (23 to 129)	-5.5 (-37.4 to 77.8)	-17.2 (-45.8 to 56.9)	7.2 (-28.9 to 117.3)
		Lead exposure	31.1 (16.2 to 50.3)	23.7 (9.6 to 45.1)	39.1 (21 to 62.4)	26.1 (13.5 to 43.7)	17 (7 to 29.3)	36.4 (19.7 to 59.9)	-16.2 (-35.7 to 7.4)	-28.5 (-49.2 to 0.1)	-6.7 (-32.1 to 26.4)
		Diet high in sodium	14.7 (2.1 to 60.8)	10.3 (2.1 to 44)	19.5 (1.8 to 82.5)	13.1 (1.9 to 52.6)	7.6 (1.6 to 30.3)	19.1 (1.8 to 77.2)	-11.1 (-48.5 to 56)	-26.1 (-64.8 to 82.4)	-2 (-44.1 to 92.8)
		High fasting plasma glucose	362.9 (273.4 to 485.6)	356.4 (248.9 to 527.6)	371.7 (274.7 to 501.7)	305.5 (222.2 to 408.1)	263.4 (182.4 to 365.1)	353 (245.9 to 488)	-15.8 (-38 to 10.9)	-26.1 (-49.6 to 6.8)	-5 (-32.4 to 29.1)

Location	Measure	Risk factor	Attributed age-standardized rate (per 100,000)						% Change (1990 to 2019)		
			1990			2019					
			Both	Female	Male	Both	Female	Male	Both	Female	Male
		High body-mass index	387.4 (223.6 to 586.6)	404.9 (232.5 to 660.7)	369.6 (202.6 to 600.3)	341.7 (201.6 to 504.4)	308.8 (181.8 to 459)	378.3 (208.8 to 593.6)	-11.8 (-35.8 to 18.7)	-23.7 (-48.8 to 12.3)	2.3 (-29.5 to 47.1)
		High systolic blood pressure	578.5 (448.5 to 766.6)	565.2 (404.4 to 832.2)	595.6 (442.8 to 823.8)	521.5 (392 to 683.1)	444 (320.5 to 593.2)	610.4 (436.8 to 829)	-9.8 (-32.7 to 15.6)	-21.5 (-44.5 to 11.8)	2.5 (-26.3 to 36.7)
		Kidney dysfunction	997 (808.4 to 1279.5)	998.4 (753.7 to 1438.2)	1001.3 (774.7 to 1310.8)	802 (615.9 to 1021.4)	702.2 (524 to 941)	916 (676.7 to 1226.5)	-19.6 (-38.9 to 2.9)	-29.7 (-50.2 to -2)	-8.5 (-33.5 to 21.7)
	YLDs	Lead exposure	3.3 (1.6 to 5.9)	3.2 (1.2 to 6)	3.5 (1.9 to 5.9)	4.6 (2.1 to 8.2)	3.7 (1.4 to 6.9)	5.6 (2.9 to 9.7)	37.1 (20.8 to 56.9)	15.6 (-1.5 to 35.5)	58.9 (35.9 to 84.5)
		Diet high in sodium	1.6 (0.3 to 6.4)	1.5 (0.3 to 5.6)	1.8 (0.2 to 7.4)	2.6 (0.4 to 9.9)	1.9 (0.4 to 7.2)	3.3 (0.3 to 12.6)	56.2 (-1.6 to 159.8)	28.9 (-31.3 to 183.1)	79.3 (13.4 to 208.5)
		High fasting plasma glucose	26 (18.6 to 35.9)	27.8 (19.7 to 38.1)	24.2 (16.5 to 34.3)	40.4 (27.8 to 56.6)	38 (26.5 to 53.3)	43 (28.5 to 62.3)	55.3 (40.2 to 72.3)	36.6 (18.7 to 56.4)	78 (54.5 to 105.1)
		High body-mass index	46.3 (25.8 to 73.3)	57 (31.8 to 88.7)	35.3 (18.3 to 58)	70.1 (40.9 to 108.6)	74.4 (43.9 to 114.4)	65.7 (36.7 to 105.2)	51.4 (32.4 to 74.9)	30.6 (12.7 to 53.1)	86.2 (55.2 to 128)
		High systolic blood pressure	64.1 (44.2 to 87.1)	74.5 (50.1 to 103)	53.4 (36.7 to 74.1)	101 (70.7 to 138.6)	102 (71.8 to 137.7)	100.2 (67.3 to 141.7)	57.5 (40.5 to 76.7)	36.9 (17.6 to 60.2)	87.8 (63.3 to 113.9)
		Kidney dysfunction	129.9 (94.7 to 171.4)	155.8 (114 to 204.3)	103.7 (74.2 to 138.7)	189.2 (138.3 to 248.2)	203.1 (149.5 to 262.2)	175.9 (124.9 to 238.8)	45.7 (34.2 to 57.5)	30.4 (18.4 to 42.9)	69.7 (52.5 to 89.4)
Jordan	Deaths	Non-optimal temperature	2.9 (1.5 to 4.1)	3.2 (1.7 to 4.7)	2.5 (1.3 to 3.7)	2.7 (1.6 to 3.8)	2.9 (1.7 to 4.2)	2.5 (1.5 to 3.7)	-5.3 (-27.1 to 28.8)	-8.6 (-35.2 to 28.1)	0.1 (-28.3 to 48.6)
		Lead exposure	1.3 (0.6 to 2.1)	1.3 (0.5 to 2.2)	1.4 (0.7 to 2.1)	1.1 (0.6 to 1.8)	1 (0.4 to 1.8)	1.2 (0.6 to 1.9)	-14.3 (-34 to 8.7)	-18.5 (-43.6 to 12.4)	-11.2 (-36.6 to 21.3)
		Diet high in sodium	0.6 (0.1 to 2.4)	0.5 (0.1 to 2.1)	0.7 (0.1 to 3)	0.5 (0.1 to 2.2)	0.4 (0.1 to 1.6)	0.7 (0.1 to 2.7)	-7 (-48.5 to 66.8)	-15.9 (-61.5 to 86.3)	-3.2 (-49.4 to 118.8)

Location	Measure	Risk factor	Attributed age-standardized rate (per 100,000)						% Change (1990 to 2019)		
			1990			2019					
			Both	Female	Male	Both	Female	Male	Both	Female	Male
		High fasting plasma glucose	15.5 (12.2 to 19.5)	17.2 (12.8 to 22.4)	13.8 (10.8 to 17.9)	13.7 (10.6 to 17)	14.3 (10.5 to 18.4)	13.1 (9.5 to 17.5)	-12.1 (-31.7 to 10.7)	-16.8 (-42.6 to 11.7)	-5.7 (-31.4 to 29.4)
		High body-mass index	15.1 (8.9 to 23)	18.9 (11 to 27.8)	11.4 (5.9 to 17.8)	16.6 (9.9 to 23.7)	18.7 (11.1 to 27.1)	14.8 (8.4 to 22.3)	10 (-19 to 47.7)	-0.7 (-31.3 to 39.7)	30.4 (-9.2 to 97.1)
		High systolic blood pressure	26.1 (21.1 to 31.8)	29.7 (22.7 to 38.3)	22.5 (17.8 to 28)	24.3 (19.9 to 29.4)	26.5 (20.5 to 33.7)	22.5 (17.3 to 28.6)	-6.8 (-26.9 to 16.8)	-10.7 (-36.4 to 21.5)	-0.4 (-28.3 to 35.3)
		Kidney dysfunction	40.9 (34.5 to 48.4)	45.9 (36 to 57.5)	35.9 (29.4 to 43.2)	36.6 (30.7 to 43.2)	39.7 (31.4 to 48.4)	34 (27 to 42.7)	-10.4 (-29.2 to 11.9)	-13.6 (-39.2 to 15.4)	-5.2 (-30.5 to 29.2)
	DALYs	Non-optimal temperature	54.6 (28.7 to 78)	60.9 (31 to 89.5)	48.4 (25.4 to 70.9)	48.2 (28.6 to 68.5)	50.2 (29.4 to 73.2)	46.4 (26.7 to 69.2)	-11.6 (-32.1 to 21.2)	-17.5 (-41.7 to 19.2)	-4.2 (-31.9 to 42)
		Lead exposure	25.1 (11.6 to 41)	24.2 (9.4 to 42.8)	26 (13.4 to 40.9)	19.4 (8.4 to 33.3)	17.1 (5.9 to 31)	21.5 (10.6 to 36.2)	-22.8 (-41 to -4.4)	-29.4 (-52.4 to -6.5)	-17.2 (-39.5 to 11.1)
		Diet high in sodium	12.7 (2.2 to 53.1)	10.8 (2.5 to 45.8)	14.7 (1.5 to 61.5)	12.4 (2 to 49.1)	9 (2.1 to 35.9)	15.3 (1.5 to 60.6)	-3 (-46.4 to 60.7)	-16.2 (-61.1 to 83.5)	4.4 (-38.6 to 102.6)
		High fasting plasma glucose	328 (261.7 to 402)	364.6 (277.4 to 467.7)	292.4 (229.3 to 368.6)	291.1 (231 to 357.4)	294.5 (226.7 to 374.4)	287.3 (217 to 371.9)	-11.3 (-28.8 to 10.4)	-19.2 (-42.1 to 7)	-1.7 (-27 to 33)
		High body-mass index	349.9 (219.3 to 495.1)	437.5 (277.9 to 611.5)	264 (147.8 to 392)	390.8 (262 to 517.3)	426 (288.1 to 573.5)	358.6 (228.8 to 508.1)	11.7 (-11 to 43.7)	-2.6 (-28.4 to 31.1)	35.8 (-1.6 to 96.2)
		High systolic blood pressure	504.3 (412.5 to 608.4)	576 (449.7 to 733.7)	432.8 (345.9 to 538.5)	482.9 (394.2 to 578.8)	506 (396.1 to 631.5)	461.4 (359.1 to 581.3)	-4.2 (-22.6 to 18.6)	-12.1 (-36.3 to 16)	6.6 (-21.9 to 42.7)
		Kidney dysfunction	899.4 (777.6 to 1042.4)	1021.9 (851.8 to 1234.9)	780.2 (653.3 to 932.2)	825.8 (707.8 to 957.1)	873.4 (720.8 to 1055.9)	782.8 (635 to 950.9)	-8.2 (-25.3 to 11.2)	-14.5 (-35.6 to 8.6)	0.3 (-22.7 to 32.2)
	YLLs	Non-optimal temperature	54.6 (28.7 to 78)	60.9 (31 to 89.5)	48.4 (25.4 to 70.9)	48.2 (28.6 to 68.5)	50.2 (29.4 to 73.2)	46.4 (26.7 to 69.2)	-11.6 (-32.1 to 21.2)	-17.5 (-41.7 to 19.2)	-4.2 (-31.9 to 42)

Location	Measure	Risk factor	Attributed age-standardized rate (per 100,000)						% Change (1990 to 2019)		
			1990			2019					
			Both	Female	Male	Both	Female	Male	Both	Female	Male
		Lead exposure	22.4 (10.5 to 36.7)	21.3 (8.2 to 37.3)	23.5 (12.2 to 37.6)	16.2 (7.1 to 28)	14.3 (5 to 26.3)	17.9 (8.8 to 30.4)	-27.7 (-46.5 to -7.8)	-32.7 (-55.6 to -7.2)	-23.8 (-47.6 to 5)
		Diet high in sodium	11.3 (1.9 to 47.3)	9.4 (2.1 to 39.9)	13.2 (1.3 to 56)	10 (1.6 to 40.7)	7.3 (1.6 to 29.3)	12.5 (1.2 to 51.1)	-11.1 (-51.4 to 51.8)	-22.4 (-65.3 to 76.9)	-5.3 (-46.8 to 92.4)
		High fasting plasma glucose	303.7 (238.9 to 375.3)	336.8 (249.1 to 437.3)	271.4 (207.4 to 345.4)	252.8 (194.2 to 315.6)	256.8 (186.5 to 337.2)	248.4 (181.4 to 333.1)	-16.7 (-35.1 to 6.4)	-23.7 (-47.8 to 3.9)	-8.5 (-33.5 to 27.4)
		High body-mass index	307.8 (190.4 to 439.1)	380.1 (231.3 to 537.8)	236.8 (129.2 to 353.6)	316.7 (207.5 to 425.1)	342.9 (223.3 to 470.2)	292.3 (178.2 to 425.1)	2.9 (-21.5 to 36.9)	-9.8 (-37.6 to 27.5)	23.4 (-15.1 to 87.9)
		High systolic blood pressure	449.9 (364.6 to 547.9)	508.3 (385.9 to 656)	391.6 (306 to 493.2)	398.8 (323.8 to 484.6)	417.5 (317.4 to 540.1)	381 (286.2 to 496.7)	-11.4 (-31 to 13.2)	-17.9 (-42.8 to 13)	-2.7 (-31 to 35.7)
		Kidney dysfunction	781 (661.2 to 912)	871.3 (694 to 1078.8)	692.7 (565.4 to 841.1)	653.1 (545.6 to 774.7)	679.6 (533.2 to 846.1)	627.9 (494.1 to 786)	-16.4 (-34.1 to 5.4)	-22 (-45.5 to 4)	-9.4 (-33.6 to 24.5)
	YLDs	Lead exposure	2.7 (1.1 to 4.8)	2.9 (1 to 5.6)	2.5 (1.2 to 4.3)	3.2 (1.3 to 6.1)	2.8 (0.8 to 5.7)	3.6 (1.5 to 6.7)	17.8 (-2.8 to 35.6)	-5.8 (-29.6 to 12.1)	44.7 (13.9 to 72.3)
		Diet high in sodium	1.4 (0.3 to 5.7)	1.4 (0.3 to 5.6)	1.5 (0.1 to 6)	2.3 (0.4 to 9.1)	1.8 (0.4 to 6.9)	2.8 (0.3 to 11)	60.3 (-8.8 to 145.9)	25 (-38.6 to 160)	90.9 (19.3 to 230.2)
		High fasting plasma glucose	24.3 (17.1 to 33.4)	27.8 (19.7 to 38.7)	20.9 (14.3 to 29.6)	38.2 (26.1 to 53)	37.7 (26.1 to 52.9)	38.9 (25.6 to 56.5)	57.1 (40.2 to 74.8)	35.5 (18.2 to 55.3)	85.9 (59.7 to 116.6)
		High body-mass index	42.1 (24.5 to 64.9)	57.3 (34.1 to 85.9)	27.2 (14.6 to 44.3)	74.1 (45.3 to 112.5)	83.1 (50.4 to 123)	66.4 (37.8 to 102.3)	76 (52.1 to 106.5)	45 (24 to 69.7)	143.8 (100.6 to 215)
		High systolic blood pressure	54.4 (37.9 to 73.6)	67.7 (46.8 to 92.6)	41.2 (28 to 56.8)	84.1 (58.7 to 114.8)	88.6 (61.4 to 122.5)	80.4 (54.3 to 113.9)	54.7 (33.9 to 77.7)	30.9 (9.4 to 58.6)	95.3 (67.5 to 127.6)
		Kidney dysfunction	118.5 (86.1 to 156.3)	150.6 (109.3 to 197.9)	87.6 (62.2 to 119.1)	172.7 (125.1 to 230.7)	193.9 (140.8 to 255.5)	154.9 (108.1 to 212.1)	45.8 (33.3 to 59.4)	28.8 (15.6 to 42.7)	76.9 (57.8 to 100.1)

Location	Measure	Risk factor	Attributed age-standardized rate (per 100,000)						% Change (1990 to 2019)		
			1990			2019					
			Both	Female	Male	Both	Female	Male	Both	Female	Male
Kuwait	Deaths	Non-optimal temperature	2.3 (-2.2 to 5.6)	2.6 (-2.3 to 6.4)	2.1 (-1.9 to 5)	1.5 (-0.9 to 3.6)	1.6 (-0.9 to 3.8)	1.5 (-0.9 to 3.5)	-33.6 (-117.2 to -2.1)	-40.4 (-117.6 to -9)	-27.6 (-116.8 to 9.6)
		Lead exposure	0.8 (0.4 to 1.3)	0.8 (0.3 to 1.3)	0.9 (0.5 to 1.4)	0.5 (0.2 to 0.8)	0.4 (0.1 to 0.7)	0.6 (0.3 to 0.9)	-41.3 (-51.6 to -28.1)	-50.7 (-64 to -33.2)	-37.1 (-49.2 to -21.3)
		Diet high in sodium	0.8 (0.1 to 2.9)	0.6 (0.1 to 2.7)	0.9 (0.1 to 3.2)	0.5 (0 to 1.8)	0.3 (0 to 1.5)	0.6 (0 to 2.2)	-39 (-65.6 to -6.6)	-47.9 (-71.8 to -2.7)	-37.3 (-68.7 to 5)
		High fasting plasma glucose	9.7 (7.7 to 11.8)	11 (8 to 13.9)	8.9 (7 to 10.9)	5.6 (4.2 to 7.2)	5.6 (4 to 7.6)	5.6 (4.1 to 7.5)	-42.3 (-52.3 to -28.7)	-48.7 (-60.6 to -28.4)	-37.4 (-49.9 to -22)
		High body-mass index	11.2 (6.6 to 16)	14.6 (8.8 to 20.6)	8.8 (5 to 13.3)	7.3 (4.2 to 10.7)	8.3 (4.9 to 12.5)	6.6 (3.5 to 10.2)	-34.3 (-50.4 to -15.9)	-42.9 (-58.1 to -18.2)	-24 (-44.9 to 3.8)
		High systolic blood pressure	18.2 (14.9 to 21)	20.6 (15.1 to 24.9)	16.5 (13.8 to 19.1)	11 (8.7 to 13.6)	11.2 (8.2 to 14.9)	10.9 (8.5 to 13.8)	-39.5 (-49.7 to -26.2)	-45.8 (-58.8 to -24.8)	-33.7 (-46.7 to -17.2)
		Kidney dysfunction	27.7 (23.8 to 30.5)	31.5 (24.2 to 36.5)	25.1 (21.9 to 28.5)	16.2 (13.3 to 19.5)	16.5 (12.5 to 21.3)	15.9 (12.8 to 19.6)	-41.5 (-51.2 to -29.1)	-47.4 (-59.2 to -27.5)	-36.4 (-48.3 to -21.4)
	DALYs	Non-optimal temperature	45.6 (-41.9 to 108.9)	53.2 (-48.1 to 128.1)	40.9 (-36.9 to 98.3)	26.4 (-15.9 to 61.1)	27.7 (-16.4 to 66.3)	25.4 (-14.9 to 59.8)	-42.2 (-114.9 to -14.7)	-47.9 (-115.4 to -20.7)	-37.9 (-114.4 to -6)
		Lead exposure	17 (7.9 to 27.7)	15.8 (5.1 to 28.1)	18 (9.5 to 27.9)	9.3 (3.9 to 15.7)	7.2 (1.9 to 13.7)	10.6 (5.1 to 17.6)	-45.7 (-55.7 to -35.8)	-54.7 (-68.9 to -39.5)	-40.9 (-52.5 to -28.4)
		Diet high in sodium	17.7 (1.3 to 61.4)	13.8 (1.4 to 59.2)	20.4 (1.2 to 66.8)	11.6 (0.8 to 41)	7.8 (0.8 to 33)	14.2 (0.8 to 47.9)	-34.2 (-59.1 to -3.7)	-43.3 (-63.2 to -8.5)	-30.5 (-59.6 to 9.7)
		High fasting plasma glucose	215.6 (175.1 to 254.2)	251.2 (189.8 to 309.3)	194.9 (158.6 to 232.1)	130.7 (103.6 to 161.5)	133.5 (100.9 to 172.7)	128.5 (99.1 to 162.9)	-39.4 (-48.6 to -26.2)	-46.8 (-57.6 to -27.5)	-34 (-45.9 to -19.8)
		High body-mass index	267.9 (173.6 to 364.4)	359.2 (233.5 to 475.6)	211.9 (129.6 to 301.1)	194.5 (130.5 to 261.3)	222.4 (152.5 to 298.6)	175.4 (114.1 to 244.5)	-27.4 (-40.4 to -10.1)	-38.1 (-50.9 to -16.9)	-17.2 (-35.4 to 6.8)

Location	Measure	Risk factor	Attributed age-standardized rate (per 100,000)						% Change (1990 to 2019)		
			1990			2019					
			Both	Female	Male	Both	Female	Male	Both	Female	Male
		High systolic blood pressure	365.8 (308.7 to 423.5)	425.6 (327.1 to 512.4)	327.5 (277.4 to 379.5)	238.1 (194.6 to 289.6)	244 (189.1 to 307)	234.1 (186.2 to 286.7)	-34.9 (-44.6 to -21.6)	-42.7 (-54.8 to -23.6)	-28.5 (-41.1 to -13.7)
		Kidney dysfunction	646.9 (579.5 to 704.4)	764.3 (630.5 to 860.4)	573 (514.8 to 640.7)	418.1 (349.9 to 488.7)	444.1 (364 to 541.6)	397.2 (325 to 475.2)	-35.4 (-44.2 to -24.8)	-41.9 (-52 to -25.9)	-30.7 (-41.9 to -17.8)
	YLLs	Non-optimal temperature	45.6 (-41.9 to 108.9)	53.2 (-48.1 to 128.1)	40.9 (-36.9 to 98.3)	26.4 (-15.9 to 61.1)	27.7 (-16.4 to 66.3)	25.4 (-14.9 to 59.8)	-42.2 (-114.9 to -14.7)	-47.9 (-115.4 to -20.7)	-37.9 (-114.4 to -6)
		Lead exposure	14.7 (6.9 to 23.8)	13.6 (4.5 to 24.1)	15.6 (8.4 to 23.9)	6.8 (2.9 to 11.7)	5.3 (1.4 to 10.5)	7.7 (3.7 to 12.7)	-54.1 (-63.6 to -43.6)	-61 (-73.6 to -45.2)	-50.5 (-61.4 to -36.9)
		Diet high in sodium	15.2 (1.1 to 53.8)	11.7 (1.1 to 50.6)	17.6 (1 to 57.8)	8.1 (0.5 to 30.2)	5.5 (0.5 to 23.7)	9.8 (0.5 to 34.2)	-46.8 (-68 to -19.8)	-53.5 (-71.4 to -19.7)	-44.3 (-69.9 to -11.2)
		High fasting plasma glucose	193.1 (152.4 to 231.9)	225.9 (165.3 to 284.7)	174 (139.4 to 210.2)	98.6 (75 to 126.5)	102.7 (72.9 to 138.8)	95.8 (70 to 127)	-48.9 (-58.1 to -36.5)	-54.5 (-65.4 to -34.5)	-44.9 (-57 to -30.6)
		High body-mass index	229.4 (147.9 to 311.9)	306.2 (195.5 to 406.5)	182.2 (110 to 259.5)	134.2 (88.6 to 182.6)	154.7 (102.4 to 220)	120.4 (75.9 to 172)	-41.5 (-54.1 to -24.3)	-49.5 (-62.2 to -26.2)	-34 (-50.8 to -10.7)
		High systolic blood pressure	316.6 (261.6 to 364.8)	366.9 (267.4 to 446.9)	284.7 (240.7 to 333.2)	169.9 (135.6 to 209.2)	175.8 (130.7 to 236)	166.2 (129.9 to 210.8)	-46.3 (-55.9 to -33.3)	-52.1 (-63.9 to -31.9)	-41.6 (-53.7 to -26.1)
		Kidney dysfunction	542.1 (473.7 to 594.3)	631.8 (496.4 to 726.5)	487 (435.5 to 545.5)	275.7 (229.1 to 332.9)	290.3 (225.8 to 373.2)	265.4 (210.9 to 330.7)	-49.1 (-57.8 to -37.7)	-54.1 (-64.5 to -35.8)	-45.5 (-56.3 to -31.5)
	YLDs	Lead exposure	2.3 (1 to 4.2)	2.2 (0.7 to 4.4)	2.4 (1.1 to 4.2)	2.5 (0.9 to 4.9)	1.9 (0.4 to 4)	2.9 (1.2 to 5.4)	7.4 (-13.8 to 26.9)	-16.5 (-40.3 to 0.5)	22 (-5 to 47.1)
		Diet high in sodium	2.5 (0.2 to 8.9)	2.1 (0.2 to 8.7)	2.8 (0.2 to 9.3)	3.6 (0.2 to 12.8)	2.4 (0.2 to 9.6)	4.4 (0.2 to 15)	41.6 (-7.2 to 95.3)	14 (-20.6 to 71.5)	55.2 (-7 to 132.4)
		High fasting plasma glucose	22.5 (15.5 to 31.2)	25.3 (17.7 to 35.7)	20.8 (13.4 to 29.9)	32.1 (21.7 to 46.1)	30.8 (21.4 to 43.4)	32.7 (21.3 to 48.8)	42.6 (24.7 to 62.2)	21.8 (4.6 to 42.4)	57 (32.3 to 87.1)

Location	Measure	Risk factor	Attributed age-standardized rate (per 100,000)						% Change (1990 to 2019)		
			1990			2019					
			Both	Female	Male	Both	Female	Male	Both	Female	Male
		High body-mass index	38.6 (22.4 to 58.5)	53 (32.1 to 78.5)	29.6 (16 to 47)	60.4 (36.3 to 91.3)	67.6 (42.6 to 100)	55.1 (31.5 to 86.2)	56.5 (34.9 to 85.4)	27.5 (7.6 to 51.3)	85.7 (52.9 to 132.5)
		High systolic blood pressure	49.2 (34.1 to 65.9)	58.7 (41 to 78.2)	42.7 (28.9 to 59.6)	68.2 (47.3 to 93.7)	68.2 (47.6 to 92.9)	67.9 (45.3 to 96.3)	38.5 (20.7 to 57.2)	16.1 (-2 to 37)	58.9 (34.5 to 86.7)
		Kidney dysfunction	104.8 (76.2 to 139.2)	132.5 (97.5 to 172.3)	86 (60.8 to 116.5)	142.4 (101.8 to 190.4)	153.9 (109.5 to 203.1)	131.8 (91.6 to 180.4)	35.8 (21.9 to 50.5)	16.1 (3.4 to 30.9)	53.3 (33.9 to 74.9)
Lebanon	Deaths	Non-optimal temperature	3.2 (2 to 4.5)	3.1 (1.9 to 4.6)	3.2 (2 to 4.6)	2.1 (1.2 to 3.2)	2.1 (1.3 to 3)	2.2 (1.2 to 3.7)	-31.8 (-52.3 to -10.3)	-32 (-53.8 to -12)	-31.9 (-59.5 to 7)
		Lead exposure	1.2 (0.7 to 1.9)	1 (0.5 to 1.7)	1.5 (0.8 to 2.2)	0.8 (0.4 to 1.2)	0.6 (0.2 to 1)	1 (0.5 to 1.7)	-37.8 (-58.2 to -14.9)	-39.8 (-60.3 to -20.8)	-35 (-62.7 to 4.1)
		Diet high in sodium	0.5 (0.1 to 1.8)	0.3 (0.1 to 1.3)	0.6 (0.1 to 2.5)	0.3 (0.1 to 1.4)	0.2 (0.1 to 0.9)	0.5 (0 to 2)	-29.9 (-59.1 to 30.2)	-31.3 (-66.2 to 38.3)	-26.9 (-65.7 to 80.6)
		High fasting plasma glucose	11.1 (8.5 to 14.3)	10.8 (7.9 to 14.8)	11.4 (8.6 to 14.9)	7.6 (5.3 to 10.4)	7.4 (4.7 to 9.8)	7.9 (4.6 to 13.3)	-31.3 (-52.1 to -9.1)	-31.8 (-53.8 to -10.9)	-30.8 (-59 to 10.5)
		High body-mass index	10.4 (5.8 to 16.1)	11.7 (6.7 to 17.9)	8.9 (4.5 to 14.7)	9.4 (5.3 to 14)	9.9 (5.5 to 14.8)	8.7 (4.1 to 15.2)	-9.4 (-38.9 to 24.4)	-15.3 (-43.9 to 17.3)	-2.1 (-44.3 to 61.9)
		High systolic blood pressure	20.4 (16.6 to 25.5)	19.6 (14.9 to 26.4)	21.3 (16.9 to 26.6)	15.4 (11.1 to 20.3)	14.6 (9.9 to 18.3)	16.3 (9.6 to 26.2)	-24.6 (-47.1 to -0.6)	-25.4 (-49.7 to -1.4)	-23.7 (-55.3 to 20.7)
		Kidney dysfunction	32.4 (27 to 39.6)	32.2 (25.6 to 42.7)	32.8 (27.3 to 39.8)	23.3 (16.8 to 30.3)	23.1 (15.8 to 27.9)	23.6 (14.2 to 37.7)	-28.1 (-49.7 to -5.9)	-28.3 (-51 to -7.3)	-28.2 (-57.3 to 13.9)
	DALYs	Non-optimal temperature	61.6 (39 to 87.7)	62.1 (38.2 to 90.3)	61.1 (38.5 to 87.5)	39 (22.9 to 58.5)	38.7 (22.7 to 55.4)	39.4 (21 to 67)	-36.6 (-54.5 to -16.5)	-37.8 (-57.1 to -18.4)	-35.5 (-59.3 to 1.7)
		Lead exposure	23.4 (12 to 36.3)	19.2 (8.5 to 33.1)	27.8 (15.7 to 41.6)	13.4 (5.9 to 22.1)	10.2 (3.5 to 18.7)	17.4 (8.3 to 30.1)	-42.5 (-60.5 to -24.8)	-46.8 (-65.6 to -30.6)	-37.5 (-59.8 to -8.2)

Location	Measure	Risk factor	Attributed age-standardized rate (per 100,000)						% Change (1990 to 2019)		
			1990			2019					
			Both	Female	Male	Both	Female	Male	Both	Female	Male
		Diet high in sodium	10.4 (1.7 to 40.9)	7.6 (1.7 to 30.5)	13.4 (1.4 to 53.5)	8.1 (1.3 to 32)	5.6 (1.4 to 21.8)	11.2 (1.1 to 45.4)	-22.1 (-50.5 to 38.6)	-26.5 (-61.5 to 54.5)	-16.5 (-53 to 66.8)
		High fasting plasma glucose	241.9 (189.9 to 306.9)	242.5 (183.1 to 319.9)	241.7 (184.1 to 312)	176.7 (129.3 to 235.3)	171.4 (121.5 to 222.8)	183.5 (120.8 to 283.6)	-26.9 (-45.8 to -7.8)	-29.3 (-49.9 to -8.3)	-24.1 (-49.3 to 11.4)
		High body-mass index	247.6 (148.7 to 360.9)	284.5 (173.3 to 411.8)	209.1 (114.3 to 318.1)	238 (148.2 to 328.6)	249.9 (161.2 to 342)	223.2 (126 to 356.5)	-3.9 (-28.9 to 27.5)	-12.2 (-36.1 to 15.3)	6.7 (-29.1 to 65.9)
		High systolic blood pressure	405 (332.8 to 498.7)	397.6 (310.6 to 510.2)	413.3 (331.4 to 514.6)	334.6 (252.2 to 429.6)	316.5 (234.9 to 389.6)	356.4 (242.9 to 536.6)	-17.4 (-38 to 5.2)	-20.4 (-42.1 to 2.8)	-13.8 (-42 to 24.8)
		Kidney dysfunction	736.4 (629.7 to 867.2)	763.9 (628.4 to 954.6)	708.4 (594.9 to 846.2)	574.7 (449.1 to 717.1)	579.6 (441.3 to 689.6)	570.7 (404.1 to 847.6)	-22 (-40.1 to -3.2)	-24.1 (-43 to -6.1)	-19.4 (-43.2 to 16.1)
	YLLs	Non-optimal temperature	61.6 (39 to 87.7)	62.1 (38.2 to 90.3)	61.1 (38.5 to 87.5)	39 (22.9 to 58.5)	38.7 (22.7 to 55.4)	39.4 (21 to 67)	-36.6 (-54.5 to -16.5)	-37.8 (-57.1 to -18.4)	-35.5 (-59.3 to 1.7)
		Lead exposure	20.8 (10.8 to 32.2)	16.6 (7.4 to 28.7)	25.1 (14.2 to 38.1)	10.6 (4.5 to 18)	8 (2.7 to 14.9)	13.7 (6.3 to 25.4)	-49.2 (-67.1 to -29.7)	-51.8 (-69.9 to -35)	-45.6 (-68.4 to -13.5)
		Diet high in sodium	9.2 (1.5 to 36.7)	6.5 (1.5 to 26.1)	12.1 (1.2 to 49.1)	6.1 (1 to 25.3)	4.2 (1 to 16.3)	8.5 (0.8 to 36.7)	-33.2 (-59.5 to 22.8)	-35.4 (-67.3 to 39.7)	-29.4 (-61.7 to 45)
		High fasting plasma glucose	221.3 (170.2 to 286.5)	220 (162.2 to 296)	223 (167.7 to 290.9)	143.5 (98.4 to 200.2)	140 (90.4 to 187.8)	147.8 (89.4 to 246.5)	-35.2 (-54.9 to -14.2)	-36.4 (-57.6 to -14.3)	-33.7 (-59.7 to 4.6)
		High body-mass index	216.8 (128.2 to 318.3)	244.4 (148.1 to 359)	188 (101.1 to 289.2)	181.2 (111.1 to 259.4)	189.6 (116.5 to 266.2)	170.7 (88 to 295.6)	-16.4 (-43.1 to 17)	-22.4 (-48.4 to 7.1)	-9.2 (-46 to 53.8)
		High systolic blood pressure	359.3 (291.6 to 447.7)	346.1 (263.5 to 453.5)	374.1 (293.8 to 474)	259 (182.8 to 348.6)	244.2 (166.3 to 305.3)	276.6 (165.7 to 454.7)	-27.9 (-49.9 to -3.7)	-29.4 (-52.4 to -6.1)	-26 (-55.5 to 16.2)
		Kidney dysfunction	633.6 (532.7 to 762.5)	639.7 (515.5 to 818.2)	628.4 (520.5 to 762.5)	423.1 (307.2 to 555.7)	419.7 (287.5 to 512.8)	427.6 (261.2 to 693.6)	-33.2 (-52.4 to -11.5)	-34.4 (-54.7 to -14.3)	-32 (-57.2 to 7.1)

Location	Measure	Risk factor	Attributed age-standardized rate (per 100,000)						% Change (1990 to 2019)		
			1990			2019					
			Both	Female	Male	Both	Female	Male	Both	Female	Male
	YLDs	Lead exposure	2.6 (1.2 to 4.5)	2.6 (1 to 4.7)	2.6 (1.3 to 4.3)	2.9 (1.2 to 5.4)	2.2 (0.7 to 4.5)	3.7 (1.7 to 6.5)	9.9 (-13.6 to 28.3)	-14.5 (-39.6 to 3.7)	40.1 (10.7 to 67.1)
		Diet high in sodium	1.2 (0.2 to 4.7)	1.1 (0.2 to 4.4)	1.4 (0.1 to 5.4)	2 (0.3 to 7.7)	1.4 (0.3 to 5.3)	2.7 (0.2 to 10.8)	60.6 (-0.4 to 154.3)	25 (-28.9 to 150.4)	98 (17.7 to 271.5)
		High fasting plasma glucose	20.6 (14.4 to 28.6)	22.5 (15.8 to 31.1)	18.6 (12.6 to 26.7)	33.3 (22.8 to 46.5)	31.4 (21.5 to 43.6)	35.7 (23.5 to 51.3)	61.2 (44.8 to 81.9)	39.2 (21.4 to 60.1)	91.3 (62.6 to 123.2)
		High body-mass index	30.8 (16.6 to 49.2)	40.1 (21.6 to 63.4)	21.1 (10.7 to 34.8)	56.7 (33.4 to 86.8)	60.3 (36.2 to 89.6)	52.5 (28.8 to 83.4)	84 (57.1 to 120.6)	50.4 (25.8 to 82.4)	149.1 (104.4 to 226.7)
		High systolic blood pressure	45.7 (31.7 to 61.6)	51.6 (34.9 to 71.1)	39.3 (26.7 to 55.6)	75.7 (52 to 104.1)	72.3 (49.6 to 98)	79.8 (52.5 to 113.5)	65.6 (45.3 to 89.8)	40.2 (17.1 to 70.7)	103.2 (74.4 to 133.7)
		Kidney dysfunction	102.7 (74.9 to 137.3)	124.2 (89.7 to 163.5)	80 (56.5 to 108.3)	151.5 (108.6 to 200.6)	160 (115.2 to 210.2)	143.1 (99.2 to 193.1)	47.5 (33.8 to 61.1)	28.9 (15.2 to 44.4)	79 (58.2 to 100.6)
Libya	Deaths	Non-optimal temperature	1.5 (-0.1 to 2.8)	1.6 (-0.1 to 3)	1.4 (-0.1 to 2.7)	1.8 (0.3 to 3.3)	1.9 (0.4 to 3.6)	1.6 (0.3 to 3.2)	19.4 (-72.6 to 148.6)	24.4 (-70.2 to 160.1)	12.9 (-75.8 to 131.9)
		Lead exposure	1 (0.5 to 1.6)	0.9 (0.4 to 1.6)	1.2 (0.6 to 1.9)	0.8 (0.3 to 1.5)	0.7 (0.2 to 1.5)	0.9 (0.4 to 1.7)	-20.7 (-46 to 6.5)	-19 (-51.2 to 10.3)	-21.8 (-49.3 to 12.5)
		Diet high in sodium	0.5 (0.1 to 1.8)	0.3 (0.1 to 1.4)	0.6 (0.1 to 2.4)	0.5 (0.1 to 1.9)	0.4 (0.1 to 1.4)	0.5 (0 to 2.3)	0.9 (-39.6 to 89.2)	4.6 (-54.2 to 139.2)	-0.8 (-49.2 to 114.4)
		High fasting plasma glucose	10.2 (7.1 to 14)	10.6 (7.1 to 15)	9.9 (6.7 to 14)	10.6 (6.7 to 15.1)	11.4 (6.7 to 16.4)	9.7 (6.2 to 15.7)	3.5 (-25.9 to 38.2)	7.3 (-28.1 to 46)	-1.4 (-32.7 to 46.4)
		High body-mass index	10.7 (6.2 to 16.4)	13 (7.5 to 19.8)	8.6 (4.5 to 14.3)	13.4 (7.6 to 20.8)	15.9 (8.9 to 24.5)	10.9 (5.7 to 19.5)	25.4 (-9.7 to 75)	22.9 (-19.5 to 70.3)	26.6 (-15.3 to 98.9)
		High systolic blood pressure	20.1 (15 to 27.2)	21.4 (14.7 to 31.5)	19 (13.5 to 26.4)	20.9 (13.7 to 28.8)	23 (13.9 to 32.6)	18.8 (12.6 to 30.3)	4 (-24 to 39.7)	7.5 (-27.1 to 44)	-0.7 (-31.6 to 44.1)

Location	Measure	Risk factor	Attributed age-standardized rate (per 100,000)						% Change (1990 to 2019)		
			1990			2019					
			Both	Female	Male	Both	Female	Male	Both	Female	Male
		Kidney dysfunction	29.8 (22.4 to 39.1)	31.3 (22.4 to 45.2)	28.4 (20.7 to 38.9)	30.4 (20.5 to 41.3)	33.4 (20.7 to 46.2)	27.4 (18.6 to 43.5)	2.1 (-25.3 to 34.9)	6.4 (-26.5 to 42.8)	-3.6 (-32.5 to 40.8)
	DALYs	Non-optimal temperature	30.4 (-1.3 to 57.4)	33.2 (-1.3 to 63.2)	28 (-1.2 to 54.6)	34.8 (6.7 to 62.5)	39 (7.3 to 72.1)	30.7 (5.8 to 61.1)	14.4 (-74 to 134.6)	17.3 (-72.1 to 139.5)	9.6 (-76.9 to 124.3)
		Lead exposure	21.1 (10.5 to 34)	18.8 (7.5 to 33.8)	23.1 (12.6 to 37.4)	14.8 (5.4 to 28)	13 (3.3 to 26.7)	16.6 (7.2 to 30.6)	-29.7 (-55.1 to -5.4)	-30.9 (-61.3 to -5.9)	-28.5 (-53.4 to 0.9)
		Diet high in sodium	10.4 (1.6 to 40.4)	8.4 (1.8 to 33.2)	12.2 (1.1 to 49)	11.2 (1.7 to 43.7)	9 (2 to 36.5)	13.4 (1.2 to 53.7)	8 (-35.9 to 101.6)	7 (-51.9 to 132.4)	9.4 (-36.6 to 108.8)
		High fasting plasma glucose	231.8 (168.7 to 307)	252.3 (175.3 to 342.7)	215.1 (153.5 to 297.8)	247.9 (168.3 to 336.6)	271.4 (171.5 to 379.5)	225.3 (153.7 to 346.7)	7 (-20.3 to 41.4)	7.6 (-25 to 42.7)	4.8 (-26.4 to 48.2)
		High body-mass index	263.9 (162.5 to 378.4)	330.2 (207.8 to 467.6)	206.2 (115.5 to 321.5)	339.2 (214.1 to 484.9)	404.7 (249 to 571.7)	275.9 (156.7 to 446.3)	28.6 (-2.6 to 70.6)	22.6 (-14.6 to 64.1)	33.8 (-5 to 98.7)
		High systolic blood pressure	420.4 (324.4 to 536.1)	468.5 (345.5 to 617.1)	379 (277.5 to 504.4)	463.9 (332.4 to 610.4)	515.1 (343.3 to 689)	414.2 (293.2 to 619.7)	10.3 (-17 to 43.5)	9.9 (-21.7 to 45.1)	9.3 (-21.5 to 53.3)
		Kidney dysfunction	717.8 (571.9 to 880.7)	800.9 (617.7 to 1021.1)	647.4 (497.7 to 839.5)	755.7 (552.5 to 970.7)	847.5 (581 to 1100.5)	667.4 (494.6 to 970.9)	5.3 (-18.2 to 33.6)	5.8 (-21.9 to 35.6)	3.1 (-23.1 to 42.4)
	YLLs	Non-optimal temperature	30.4 (-1.3 to 57.4)	33.2 (-1.3 to 63.2)	28 (-1.2 to 54.6)	34.8 (6.7 to 62.5)	39 (7.3 to 72.1)	30.7 (5.8 to 61.1)	14.4 (-74 to 134.6)	17.3 (-72.1 to 139.5)	9.6 (-76.9 to 124.3)
		Lead exposure	18.4 (9.2 to 29.9)	16.2 (6.4 to 29.5)	20.5 (11 to 33.6)	12.2 (4.4 to 23.7)	10.8 (2.6 to 22.9)	13.6 (5.7 to 26.5)	-33.7 (-59.3 to -7.2)	-33.1 (-65.5 to -4.4)	-33.9 (-59.9 to -2.4)
		Diet high in sodium	9 (1.3 to 35.9)	7.2 (1.5 to 28.2)	10.8 (1 to 43.7)	9.1 (1.3 to 36.7)	7.3 (1.5 to 30.1)	10.8 (0.9 to 44.6)	0.3 (-41.6 to 93.1)	2.4 (-55.6 to 129.5)	-0.1 (-45.7 to 100.4)
		High fasting plasma glucose	210.5 (147.1 to 284.1)	228.7 (152.1 to 320.7)	195.8 (133.9 to 277.4)	214.3 (137.3 to 301.8)	238 (140.6 to 341.2)	191.5 (121.1 to 307.7)	1.8 (-27.7 to 40)	4.1 (-31.1 to 43.9)	-2.2 (-35.2 to 44.2)

Location	Measure	Risk factor	Attributed age-standardized rate (per 100,000)						% Change (1990 to 2019)		
			1990			2019					
			Both	Female	Male	Both	Female	Male	Both	Female	Male
		High body-mass index	228.6 (137.5 to 330.3)	282.3 (174.1 to 409.7)	181.8 (99.4 to 290.1)	277.5 (165 to 410.2)	332.9 (192.8 to 487.4)	223.7 (121.2 to 386.6)	21.4 (-13.9 to 68.7)	17.9 (-23 to 65.9)	23 (-19 to 94.3)
		High systolic blood pressure	367.8 (276.4 to 480.4)	403.8 (284.3 to 549.7)	337.1 (238.2 to 463.9)	380.9 (250.4 to 523.4)	425.4 (252.2 to 594.1)	337.4 (224 to 541.6)	3.5 (-26 to 40.1)	5.4 (-29.9 to 45)	0.1 (-31.9 to 49.1)
		Kidney dysfunction	613.1 (469.2 to 770.8)	670.2 (488.2 to 880.3)	565.1 (423.6 to 754)	600 (403.1 to 810.5)	673.3 (417.3 to 921.6)	529.1 (362.2 to 824.4)	-2.1 (-29 to 31.4)	0.5 (-31.5 to 35.7)	-6.4 (-35.4 to 36.8)
	YLDs	Lead exposure	2.6 (1.2 to 4.6)	2.7 (1 to 5)	2.6 (1.3 to 4.3)	2.6 (1 to 5.2)	2.2 (0.6 to 4.7)	3 (1.3 to 5.6)	-1.2 (-24.9 to 17.2)	-17.1 (-46.3 to 0.7)	14.9 (-12.8 to 40.4)
		Diet high in sodium	1.3 (0.2 to 5.1)	1.2 (0.3 to 4.7)	1.4 (0.1 to 5.7)	2.1 (0.3 to 8)	1.7 (0.4 to 6.8)	2.6 (0.2 to 9.9)	59.2 (-0.5 to 164.1)	33.8 (-28.6 to 152)	81.2 (16.4 to 230)
		High fasting plasma glucose	21.3 (14.8 to 29.1)	23.6 (16.5 to 32.2)	19.3 (12.9 to 27.7)	33.6 (23.2 to 47)	33.4 (23.1 to 46.1)	33.9 (22.6 to 49.3)	58.2 (41.2 to 76.5)	41.6 (22.9 to 61.3)	75.6 (50.4 to 107.8)
		High body-mass index	35.3 (19.9 to 55.5)	47.9 (27.3 to 74.3)	24.4 (13 to 39.2)	61.8 (37.3 to 92.3)	71.8 (43.8 to 108.5)	52.2 (30.2 to 81.3)	75.1 (54.2 to 106.3)	49.9 (27.4 to 77.8)	114.1 (77 to 174.7)
		High systolic blood pressure	52.6 (37.1 to 70.6)	64.8 (44.5 to 89.2)	41.9 (28.8 to 57.1)	83.1 (58.1 to 114.3)	89.7 (62.7 to 122.5)	76.8 (53.1 to 107.4)	57.8 (42 to 76.9)	38.5 (18.7 to 62.7)	83.2 (59.5 to 111.2)
		Kidney dysfunction	104.8 (76.2 to 139.1)	130.7 (95.1 to 173.5)	82.3 (58.1 to 111)	155.7 (113.5 to 207)	174.2 (125.6 to 231.3)	138.3 (98.3 to 189)	48.6 (36.6 to 61.5)	33.3 (19.1 to 47.7)	68.1 (50 to 88.2)
Morocco	Deaths	Non-optimal temperature	2.3 (1.6 to 3.5)	2.2 (1.5 to 3.6)	2.5 (1.7 to 4)	2.9 (2 to 4)	2.9 (1.9 to 4.1)	2.8 (1.9 to 4.3)	23.2 (-8.1 to 56.1)	32 (-9.4 to 76.7)	13.2 (-17.7 to 53)
		Lead exposure	1.2 (0.7 to 1.9)	0.9 (0.5 to 1.7)	1.5 (0.8 to 2.5)	1.4 (0.8 to 2.2)	1.2 (0.6 to 2)	1.7 (0.9 to 2.7)	19.4 (-7.6 to 49.5)	23.9 (-15.3 to 65.1)	13.6 (-17.7 to 50.2)
		Diet high in sodium	0.5 (0.1 to 2)	0.3 (0.1 to 1.3)	0.6 (0.1 to 2.8)	0.6 (0.1 to 2.3)	0.4 (0.1 to 1.7)	0.7 (0.1 to 3)	18.7 (-31.6 to 121.5)	25.7 (-44.2 to 178.8)	13.2 (-44.8 to 149)

Location	Measure	Risk factor	Attributed age-standardized rate (per 100,000)						% Change (1990 to 2019)		
			1990			2019					
			Both	Female	Male	Both	Female	Male	Both	Female	Male
		High fasting plasma glucose	9.3 (6.8 to 13.1)	8.6 (6 to 13.2)	10.2 (7.1 to 15.8)	11.7 (8.4 to 16)	11.6 (8 to 16.1)	11.9 (7.9 to 17.7)	25.6 (-7.9 to 58.1)	34.4 (-8.6 to 82.1)	16.1 (-16 to 54.9)
		High body-mass index	7.6 (4 to 12.7)	8.4 (4.5 to 13.9)	6.9 (3.1 to 13.1)	13.1 (7.6 to 20.4)	14.9 (8.5 to 22.4)	11.3 (5.5 to 19)	73.1 (20.4 to 147.9)	78.4 (15.9 to 165.4)	65.1 (10.7 to 164)
		High systolic blood pressure	20.4 (16.2 to 29.6)	19.8 (15.2 to 31.4)	21.4 (15.8 to 31.6)	25.2 (19.4 to 33.2)	26 (19.2 to 35.9)	24.4 (17.1 to 35.2)	23.3 (-7.8 to 55.1)	31.1 (-11.4 to 75.4)	13.8 (-17.9 to 52.3)
		Kidney dysfunction	29.4 (24.6 to 41.4)	27.8 (22.2 to 43.3)	31.7 (24.7 to 45.3)	35.8 (27.9 to 46.3)	36.3 (27.4 to 49)	35.4 (25.1 to 50.2)	21.8 (-8.9 to 51.6)	30.5 (-9.2 to 71.2)	11.9 (-18 to 48.9)
	DALYs	Non-optimal temperature	49 (34.1 to 68.1)	48.7 (33.3 to 69.6)	49.7 (33.4 to 70.6)	54.6 (36.5 to 74.6)	57.7 (37.3 to 80.6)	51.6 (33.4 to 76.6)	11.3 (-16.9 to 41.3)	18.4 (-19 to 61.3)	3.7 (-25.2 to 36.6)
		Lead exposure	23.7 (12.9 to 38.5)	19.8 (9.8 to 33.8)	28 (15.5 to 45.1)	25.5 (13.1 to 41.6)	21.3 (9.2 to 37)	30 (16 to 47.6)	7.6 (-17.8 to 32.6)	7.7 (-25.2 to 41.4)	7.1 (-20.8 to 38.5)
		Diet high in sodium	10.4 (1.6 to 43.1)	7.6 (1.7 to 30.9)	13.4 (1.2 to 56.3)	12.6 (2 to 49.9)	9.5 (2.1 to 38.6)	15.8 (1.5 to 64.7)	20.8 (-29.1 to 120.5)	25.7 (-43.7 to 177.5)	17.9 (-33.7 to 133.1)
		High fasting plasma glucose	210.1 (157.8 to 278)	204 (149.5 to 286.6)	217.5 (158.1 to 308.3)	262.8 (194.2 to 342.7)	269.1 (192.8 to 359.5)	257.1 (177.6 to 363.2)	25.1 (-7.4 to 56.2)	32 (-8.6 to 77.5)	18.2 (-13.7 to 53.7)
		High body-mass index	188.7 (106.2 to 288.3)	218.2 (126.4 to 332)	159 (79.9 to 274.2)	321.3 (196.8 to 457.5)	373.5 (235.1 to 525.8)	268.6 (149.1 to 410.1)	70.3 (23.2 to 134.2)	71.1 (16.8 to 142.9)	68.9 (15.5 to 161.5)
		High systolic blood pressure	422.5 (347.7 to 550.8)	436.3 (343.2 to 610.7)	411.1 (313.8 to 564)	523.8 (405.6 to 654.1)	562.1 (428 to 718.1)	485.8 (350.3 to 645.3)	24 (-5.3 to 53.5)	28.8 (-7.8 to 69.5)	18.2 (-12.4 to 54.6)
		Kidney dysfunction	706 (615.6 to 865.7)	721.9 (603 to 936.4)	693.7 (567.2 to 885.4)	844.6 (673.2 to 1035.9)	899.7 (705.6 to 1101.7)	790.6 (593.6 to 1025.3)	19.6 (-7 to 44.6)	24.6 (-8.6 to 58.6)	14 (-12.8 to 43.6)
	YLLs	Non-optimal temperature	49 (34.1 to 68.1)	48.7 (33.3 to 69.6)	49.7 (33.4 to 70.6)	54.6 (36.5 to 74.6)	57.7 (37.3 to 80.6)	51.6 (33.4 to 76.6)	11.3 (-16.9 to 41.3)	18.4 (-19 to 61.3)	3.7 (-25.2 to 36.6)

Location	Measure	Risk factor	Attributed age-standardized rate (per 100,000)						% Change (1990 to 2019)		
			1990			2019					
			Both	Female	Male	Both	Female	Male	Both	Female	Male
		Lead exposure	21.2 (11.4 to 34.9)	17.2 (8.5 to 29.6)	25.6 (13.9 to 42.1)	21.6 (10.9 to 35.1)	18 (7.9 to 32.5)	25.3 (13.2 to 41.3)	1.5 (-24.4 to 28)	4.4 (-31.3 to 42.5)	-1.1 (-29.5 to 31.6)
		Diet high in sodium	9.3 (1.4 to 38.6)	6.5 (1.4 to 26.6)	12.2 (1.1 to 51.4)	10.4 (1.6 to 41.3)	7.9 (1.7 to 32.3)	13 (1.2 to 54.6)	11.8 (-36.6 to 114.6)	20.1 (-47.2 to 176.8)	7.1 (-42.3 to 115.2)
		High fasting plasma glucose	193.1 (141 to 260.2)	185.4 (131.7 to 264.9)	202.1 (142.5 to 290.9)	228.7 (162.1 to 309.3)	236.7 (161.8 to 329.3)	221.4 (144.5 to 326.8)	18.5 (-15 to 51.8)	27.7 (-15.8 to 78)	9.6 (-22.7 to 45.5)
		High body-mass index	167 (92.5 to 257)	189.3 (106.5 to 294.6)	144.8 (70.9 to 253.6)	267 (159.3 to 390.7)	310.7 (190.1 to 441.1)	223 (116.6 to 354.2)	59.9 (11.5 to 126.9)	64.2 (5.7 to 144.9)	54 (1.4 to 145.3)
		High systolic blood pressure	376.1 (301.1 to 498.1)	379 (291.3 to 546.7)	376.1 (282.6 to 531.7)	436.8 (327.2 to 563.2)	468.2 (343.3 to 617.5)	405.9 (276 to 566.2)	16.1 (-15.1 to 48.1)	23.5 (-17.5 to 69.8)	7.9 (-23.2 to 44)
		Kidney dysfunction	617.5 (531.5 to 776.4)	613.4 (496 to 828.1)	626.2 (502.7 to 816.3)	679.4 (523.7 to 859.1)	718.5 (530.5 to 906.4)	641.6 (450.5 to 874.4)	10 (-18 to 37.8)	17.1 (-20.1 to 56.5)	2.5 (-25.8 to 34.1)
	YLDs	Lead exposure	2.5 (1.2 to 4.2)	2.6 (1.1 to 4.6)	2.4 (1.3 to 4)	4 (1.9 to 6.8)	3.3 (1.4 to 6.2)	4.7 (2.4 to 7.8)	59.5 (37.6 to 82.1)	29.4 (6.5 to 48.9)	93.5 (61.1 to 126.7)
		Diet high in sodium	1.1 (0.2 to 4.3)	1 (0.2 to 4.2)	1.2 (0.1 to 4.8)	2.2 (0.3 to 8.5)	1.7 (0.4 to 6.4)	2.7 (0.2 to 10.9)	96.2 (13.9 to 217.7)	60.7 (-16.3 to 213.3)	128.3 (40.4 to 331.3)
		High fasting plasma glucose	17 (12.1 to 24.2)	18.6 (12.9 to 25.7)	15.4 (10.4 to 22.1)	34.1 (23.6 to 48.1)	32.5 (22.6 to 45.4)	35.8 (24.1 to 52.2)	100.1 (79.6 to 124.9)	75 (53.4 to 100.4)	131.7 (98.4 to 172.9)
		High body-mass index	21.7 (11.3 to 35.3)	29 (15.3 to 46.7)	14.3 (6.5 to 24.3)	54.3 (30.6 to 85.6)	62.7 (36.4 to 96.6)	45.6 (24.3 to 74)	149.9 (108.1 to 217.8)	116.5 (77.1 to 175.5)	219.7 (149.1 to 368.2)
		High systolic blood pressure	46.4 (32 to 62.6)	57.2 (39.5 to 78.5)	35 (23.9 to 49)	87 (59.9 to 117.9)	93.9 (65.1 to 127.2)	79.9 (54 to 111.3)	87.3 (66.5 to 109.9)	64 (40.8 to 89.7)	128.4 (97.3 to 161.6)
		Kidney dysfunction	88.4 (63.9 to 117.4)	108.5 (79 to 141.4)	67.5 (48 to 91.1)	165.2 (119.3 to 219.1)	181.2 (131.1 to 239.6)	149 (104.8 to 203.7)	86.7 (70.9 to 102.3)	67.1 (49.9 to 84.7)	120.6 (96.7 to 148.1)

Location	Measure	Risk factor	Attributed age-standardized rate (per 100,000)						% Change (1990 to 2019)		
			1990			2019					
			Both	Female	Male	Both	Female	Male	Both	Female	Male
Oman	Deaths	Non-optimal temperature	1.2 (-3.6 to 4.1)	1.2 (-3.5 to 4.1)	1.4 (-4.2 to 4.6)	1.6 (-2.6 to 4.4)	1.6 (-2.7 to 4.4)	1.8 (-2.6 to 4.9)	35.2 (-201.7 to 113.8)	37.4 (-206.4 to 119)	29.2 (-198.9 to 109.7)
		Lead exposure	0.6 (0.3 to 1)	0.5 (0.3 to 0.9)	0.8 (0.4 to 1.3)	0.7 (0.4 to 1.1)	0.6 (0.3 to 1)	0.9 (0.5 to 1.3)	12.5 (-22.1 to 52.4)	9.4 (-27.2 to 53.2)	11 (-32.9 to 62.6)
		Diet high in sodium	0.2 (0 to 1)	0.2 (0 to 0.7)	0.3 (0 to 1.5)	0.3 (0.1 to 1.2)	0.2 (0.1 to 0.9)	0.4 (0 to 1.6)	20.8 (-32.5 to 138.5)	22.7 (-43.6 to 171.5)	13.7 (-45.9 to 182)
		High fasting plasma glucose	6 (4.3 to 8.2)	5.7 (4 to 7.9)	6.7 (4.7 to 10)	7.1 (5.5 to 9)	6.9 (5 to 8.9)	7.8 (5.9 to 10.3)	19.9 (-15.2 to 64.8)	21.8 (-16.8 to 71.1)	15.3 (-26 to 72.4)
		High body-mass index	3.6 (1.7 to 6.1)	4.2 (2.1 to 7)	3.1 (1.2 to 5.9)	8 (4.6 to 12)	8.6 (5.1 to 12.7)	7.8 (3.5 to 12.5)	123.9 (40.4 to 298.4)	103.6 (27.6 to 237.2)	151.4 (38.5 to 456.2)
		High systolic blood pressure	9.7 (7.3 to 13)	9.1 (6.7 to 12.4)	11.3 (8.3 to 16.3)	13.6 (11.3 to 16.3)	13.3 (10.4 to 16.6)	14.6 (11.5 to 18)	40.1 (-1.2 to 91.5)	46.8 (-1.8 to 105.8)	29.4 (-18.8 to 93.8)
		Kidney dysfunction	16.8 (12.9 to 22.3)	16.2 (12.4 to 21.6)	18.9 (14.1 to 26.9)	19.9 (17.1 to 22.8)	19.4 (15.6 to 23.2)	21.4 (17.4 to 25.9)	18 (-15.1 to 58.5)	19.8 (-17.5 to 62.4)	13 (-28.3 to 65.7)
	DALYs	Non-optimal temperature	22.5 (-66.7 to 76.8)	22.9 (-69.8 to 79.7)	23.3 (-67.7 to 79.1)	26.1 (-37.9 to 71.4)	27.4 (-42.4 to 74.4)	26 (-36.5 to 72)	15.8 (-187.7 to 82.2)	19.8 (-189.4 to 84.8)	11.4 (-187 to 78.6)
		Lead exposure	13.6 (7 to 21.8)	12.5 (5.7 to 20.7)	15.1 (8.2 to 24)	13.4 (6.4 to 21.5)	12 (4.8 to 20.4)	15 (7.6 to 23.2)	-1.6 (-28.2 to 26)	-4 (-32.7 to 25.9)	-0.6 (-33.9 to 37.1)
		Diet high in sodium	5.8 (0.9 to 24)	4.5 (1 to 18.7)	7.3 (0.7 to 32.1)	7.3 (1.2 to 28.9)	5.6 (1.3 to 22.3)	8.9 (0.9 to 37.4)	24.9 (-26.1 to 126.4)	25.8 (-37.4 to 184.2)	21 (-29.6 to 160.9)
		High fasting plasma glucose	135.4 (101.3 to 178.8)	136.8 (102.6 to 181)	139.6 (100.7 to 200.7)	157.3 (128.1 to 188.2)	161 (126.7 to 197.9)	159.2 (126.8 to 194.8)	16.2 (-11.8 to 50.2)	17.7 (-12.1 to 56.1)	14.1 (-19.9 to 59.6)
		High body-mass index	99.8 (52.2 to 158.8)	127.4 (71.4 to 196.3)	78.3 (33 to 137.3)	213.1 (140.5 to 294)	243.8 (162.1 to 335.5)	191.9 (118.1 to 276.2)	113.5 (49.8 to 238.6)	91.4 (34.3 to 189.3)	145.1 (57.4 to 384.6)

Location	Measure	Risk factor	Attributed age-standardized rate (per 100,000)						% Change (1990 to 2019)		
			1990			2019					
			Both	Female	Male	Both	Female	Male	Both	Female	Male
		High systolic blood pressure	207.9 (159.8 to 268.2)	208.7 (155.9 to 274.5)	214.9 (159 to 297)	299.2 (249.8 to 351.4)	317 (256.4 to 384.1)	291.7 (235.8 to 353.1)	43.9 (10.3 to 85.6)	51.9 (12.6 to 100.9)	35.8 (-5.2 to 87.2)
		Kidney dysfunction	413.4 (332.6 to 518.6)	443.8 (356.1 to 561.2)	402 (311 to 542.7)	491.3 (428.6 to 558.9)	533.3 (452.5 to 617.9)	472 (394.2 to 554.3)	18.8 (-6.6 to 46.5)	20.2 (-6 to 47.7)	17.4 (-16.3 to 56.6)
	YLLs	Non-optimal temperature	22.5 (-66.7 to 76.8)	22.9 (-69.8 to 79.7)	23.3 (-67.7 to 79.1)	26.1 (-37.9 to 71.4)	27.4 (-42.4 to 74.4)	26 (-36.5 to 72)	15.8 (-187.7 to 82.2)	19.8 (-189.4 to 84.8)	11.4 (-187 to 78.6)
		Lead exposure	10.7 (5.4 to 17.3)	9.3 (4.3 to 16.1)	12.5 (6.6 to 20.6)	9.5 (4.5 to 15.1)	8.2 (3.3 to 14.1)	11 (5.6 to 17.4)	-11.4 (-38.1 to 21.5)	-12 (-42.8 to 24.2)	-11.9 (-46.3 to 29.6)
		Diet high in sodium	4.6 (0.7 to 19.7)	3.3 (0.7 to 14.4)	6 (0.6 to 26.5)	4.8 (0.8 to 20)	3.7 (0.8 to 15.2)	6 (0.6 to 26.1)	5.6 (-40.5 to 107.7)	10.3 (-49.2 to 158)	0.6 (-46.5 to 131.4)
		High fasting plasma glucose	114.1 (81 to 157.7)	113.5 (80.3 to 157.3)	120 (82.1 to 179)	117.7 (91.2 to 145.4)	121.8 (90.2 to 156.2)	118.9 (89.2 to 154.4)	3.1 (-25.7 to 42.5)	7.3 (-25 to 52.9)	-0.9 (-33.8 to 49.3)
		High body-mass index	76.4 (38.7 to 123.6)	93.7 (49.2 to 149.3)	63.2 (25.2 to 115.7)	139.5 (87.5 to 195.2)	156.7 (99.6 to 220.9)	128.3 (75.4 to 194.5)	82.5 (20.6 to 211.2)	67.3 (8.6 to 176)	103 (21.8 to 322.7)
		High systolic blood pressure	166.9 (124.9 to 224.4)	161.2 (116.6 to 221.1)	180.5 (128.3 to 260.7)	205.6 (169.9 to 245.5)	213.3 (166.4 to 266)	206.3 (164.1 to 257.4)	23.2 (-11 to 70.1)	32.3 (-9.8 to 87.4)	14.3 (-24.3 to 70.5)
		Kidney dysfunction	311.3 (240.7 to 412.9)	317.2 (239.5 to 424.7)	321.5 (236.9 to 463.3)	314.7 (271.9 to 362)	330.8 (269.3 to 393.8)	313.5 (256.4 to 382.9)	1.1 (-26.3 to 35.9)	4.3 (-25.9 to 42.1)	-2.5 (-35.4 to 43.3)
	YLDs	Lead exposure	2.9 (1.4 to 4.9)	3.1 (1.3 to 5.6)	2.6 (1.3 to 4.3)	3.9 (1.6 to 7)	3.8 (1.4 to 7.1)	4 (1.7 to 7.2)	34.9 (9.3 to 54.1)	19.9 (-8.4 to 40.9)	54.3 (22.2 to 84)
		Diet high in sodium	1.3 (0.2 to 5.1)	1.2 (0.3 to 4.7)	1.3 (0.1 to 5.5)	2.5 (0.4 to 9.6)	2 (0.4 to 7.6)	2.8 (0.3 to 11.6)	95.3 (18.4 to 201.9)	69.9 (-5.9 to 250.2)	112.8 (32.9 to 285.1)
		High fasting plasma glucose	21.3 (15.1 to 29.2)	23.3 (16.3 to 31.8)	19.6 (13.6 to 27.5)	39.6 (27.4 to 54.6)	39.3 (27.3 to 53.6)	40.3 (26.9 to 57.4)	85.9 (66.6 to 106.9)	68.2 (48.1 to 91.8)	106 (76.8 to 140.3)

Location	Measure	Risk factor	Attributed age-standardized rate (per 100,000)						% Change (1990 to 2019)		
			1990			2019					
			Both	Female	Male	Both	Female	Male	Both	Female	Male
		High body-mass index	23.4 (11.9 to 39.1)	33.7 (17.4 to 54.6)	15.1 (6.6 to 27.5)	73.7 (45.8 to 112.3)	87.1 (53.8 to 133.2)	63.6 (37.7 to 100.6)	214.8 (137 to 370.6)	158.4 (94.6 to 266.3)	321.5 (197.3 to 699.4)
		High systolic blood pressure	41 (28.4 to 56.2)	47.5 (32.1 to 65.1)	34.4 (23.7 to 47.6)	93.6 (65.6 to 126.3)	103.7 (71.2 to 142.1)	85.5 (58.4 to 118.5)	128.3 (102.1 to 159.2)	118.4 (82.3 to 162.8)	148.4 (109.3 to 192.6)
		Kidney dysfunction	102.1 (74 to 134.5)	126.6 (92.3 to 164.9)	80.5 (58.4 to 109.1)	176.6 (130.1 to 234.1)	202.5 (149.2 to 267.5)	158.5 (113.8 to 215.2)	73.1 (59.2 to 87.9)	60 (46.1 to 75)	96.8 (73.8 to 120.3)
Palestine	Deaths	Non-optimal temperature	2.9 (0.9 to 4.7)	2.7 (0.8 to 4.4)	3.2 (1 to 5.4)	2.1 (0.8 to 3.1)	1.9 (0.8 to 2.9)	2.4 (1 to 3.7)	-29.4 (-49.1 to 6.7)	-30.5 (-49.7 to 6.5)	-26.2 (-49 to 12.1)
		Lead exposure	2.1 (1.3 to 3.2)	1.6 (0.9 to 2.6)	2.8 (1.7 to 4.2)	1.4 (0.8 to 2)	1 (0.5 to 1.6)	2 (1.2 to 2.8)	-35.5 (-50.8 to -17.7)	-36.9 (-53.5 to -16.2)	-30 (-49.7 to -9.4)
		Diet high in sodium	0.7 (0.1 to 2.7)	0.5 (0.1 to 1.8)	1 (0.1 to 4.1)	0.5 (0.1 to 2)	0.3 (0.1 to 1.3)	0.7 (0.1 to 3)	-28.2 (-59.2 to 27)	-30.6 (-68.4 to 55.6)	-24.4 (-60.4 to 54.1)
		High fasting plasma glucose	15.9 (11.7 to 21.1)	14.7 (10.5 to 19.7)	17.7 (12.9 to 24)	11.9 (9.4 to 14.8)	10.8 (8.4 to 13.7)	13.8 (10.6 to 17.3)	-25.3 (-43 to -3.5)	-26.2 (-44.2 to -0.7)	-22.3 (-42.4 to 2.7)
		High body-mass index	12 (6 to 19.8)	13.4 (7.3 to 20.9)	10.5 (4.3 to 19.1)	11.1 (6.3 to 17)	11.7 (6.9 to 17.2)	10.6 (5.3 to 17.4)	-7.5 (-32.7 to 33.2)	-12.9 (-38.4 to 26.1)	1 (-30.2 to 60.6)
		High systolic blood pressure	28.6 (22.6 to 35.6)	26.2 (19.6 to 33.4)	32 (25.3 to 40.7)	20.8 (17 to 24.7)	18.4 (14.7 to 22.5)	25.1 (19.9 to 30.3)	-27.2 (-43.5 to -7)	-29.7 (-47.2 to -5.6)	-21.7 (-43.6 to 2.1)
		Kidney dysfunction	46.8 (37.8 to 57.4)	43.6 (34 to 54.3)	51.6 (41.7 to 64.6)	33.6 (28.5 to 38.6)	30.8 (25.9 to 36)	38.7 (32 to 45.2)	-28.3 (-44.8 to -8.8)	-29.3 (-45.7 to -7.2)	-25 (-44.2 to -2.9)
	DALYs	Non-optimal temperature	60.6 (18.5 to 99.5)	58.6 (17.3 to 95.8)	63.6 (19.1 to 105.1)	39.4 (15.7 to 59.8)	36.7 (15 to 55.9)	43.2 (17.5 to 66.5)	-35 (-51.9 to -0.9)	-37.4 (-54.3 to -3.8)	-32.1 (-52.7 to 5.1)
		Lead exposure	40.9 (23.5 to 62.7)	32.2 (16 to 52.2)	51.9 (31.4 to 77.1)	24.8 (13.6 to 38)	18.8 (8.5 to 31.2)	33 (19.5 to 48.7)	-39.4 (-53.9 to -22.4)	-41.7 (-56.8 to -23.7)	-36.3 (-53 to -17.3)

Location	Measure	Risk factor	Attributed age-standardized rate (per 100,000)						% Change (1990 to 2019)		
			1990			2019					
			Both	Female	Male	Both	Female	Male	Both	Female	Male
		Diet high in sodium	15.2 (2.4 to 59)	10.8 (2.4 to 41.3)	20.6 (2 to 84.5)	11.7 (1.9 to 46.2)	7.8 (1.8 to 31.5)	16.2 (1.7 to 64)	-23 (-55.3 to 30.5)	-27.6 (-65.3 to 61.1)	-21 (-53.3 to 49.4)
		High fasting plasma glucose	353 (263.7 to 464.3)	335 (244.7 to 447.5)	377.2 (274.7 to 503.6)	269 (216.5 to 326.7)	248.1 (197.4 to 306.4)	296.5 (236.1 to 363.8)	-23.8 (-40.8 to -1.9)	-26 (-43.1 to -2.3)	-21.4 (-41.2 to 3.5)
		High body-mass index	310.6 (170.4 to 479)	354.9 (206.2 to 526.5)	258.5 (119.5 to 435.4)	289.9 (180.7 to 411.6)	306.3 (199.1 to 423.2)	271.7 (156.4 to 413.8)	-6.7 (-28.7 to 27.1)	-13.7 (-35.1 to 17.3)	5.1 (-24.4 to 61.5)
		High systolic blood pressure	574.4 (449.3 to 717.6)	543.3 (406.2 to 689.3)	616.1 (478.1 to 791.4)	434.2 (358.8 to 515.7)	393.6 (318.6 to 480.9)	490 (394.7 to 589)	-24.4 (-41.2 to -4.4)	-27.5 (-44.7 to -4.3)	-20.5 (-40 to 3)
		Kidney dysfunction	1093.4 (884.3 to 1330.9)	1082.5 (863.9 to 1325.4)	1119.8 (892.7 to 1396.3)	809.9 (707.3 to 918.1)	775.6 (672.3 to 886.6)	862.7 (741.5 to 999.5)	-25.9 (-40.9 to -7.8)	-28.4 (-42.7 to -9.9)	-23 (-39.7 to -1.9)
	YLLs	Non-optimal temperature	60.6 (18.5 to 99.5)	58.6 (17.3 to 95.8)	63.6 (19.1 to 105.1)	39.4 (15.7 to 59.8)	36.7 (15 to 55.9)	43.2 (17.5 to 66.5)	-35 (-51.9 to -0.9)	-37.4 (-54.3 to -3.8)	-32.1 (-52.7 to 5.1)
		Lead exposure	37.3 (21.3 to 57.9)	28.7 (14.2 to 46.8)	48.2 (28.6 to 72.9)	20.7 (11.4 to 31.6)	15.4 (7 to 25.5)	28 (16.3 to 41)	-44.5 (-59.1 to -27.3)	-46.4 (-62 to -27.6)	-41.8 (-58.7 to -22.4)
		Diet high in sodium	13.7 (2.1 to 53.6)	9.5 (2 to 36.7)	18.9 (1.8 to 78.8)	9.6 (1.5 to 38.6)	6.2 (1.4 to 24.9)	13.5 (1.4 to 52.8)	-30.4 (-60.4 to 20.9)	-34.3 (-70.4 to 52)	-28.8 (-59.4 to 38.4)
		High fasting plasma glucose	328.6 (239.2 to 441.6)	309.4 (219.5 to 420.5)	354.2 (251.8 to 484.2)	232.7 (182.6 to 292.4)	214 (165.1 to 271.6)	257.6 (199.5 to 323.2)	-29.2 (-46 to -6.4)	-30.8 (-48.4 to -5.9)	-27.3 (-46.5 to -2.1)
		High body-mass index	278.5 (149.3 to 435.7)	313.2 (178.2 to 468.1)	237.8 (107.9 to 403)	235.8 (143.9 to 341.6)	245.6 (155 to 340.7)	225 (125.2 to 347.9)	-15.3 (-38.6 to 19.4)	-21.6 (-43.7 to 11.1)	-5.4 (-33.9 to 47.4)
		High systolic blood pressure	522.9 (399.1 to 662.6)	486.3 (356.8 to 623.7)	571.2 (436.6 to 742.4)	360 (291.7 to 433.3)	321.9 (254.9 to 396.9)	412.9 (327.5 to 506.5)	-31.2 (-47.4 to -10.1)	-33.8 (-51.2 to -9.9)	-27.7 (-46.9 to -3.8)
		Kidney dysfunction	970.5 (768.3 to 1204.2)	939.3 (732.6 to 1172.3)	1019.4 (797.9 to 1297.2)	641.4 (547.2 to 737)	597.7 (505 to 697)	703.6 (589.9 to 823.1)	-33.9 (-48.9 to -14.5)	-36.4 (-51 to -16.1)	-31 (-48 to -9.1)

Location	Measure	Risk factor	Attributed age-standardized rate (per 100,000)						% Change (1990 to 2019)		
			1990			2019					
			Both	Female	Male	Both	Female	Male	Both	Female	Male
	YLDs	Lead exposure	3.6 (1.9 to 6.1)	3.5 (1.6 to 6)	3.7 (2.1 to 6.1)	4.1 (2 to 7.3)	3.4 (1.4 to 6.3)	5 (2.6 to 8.6)	14.3 (-2.2 to 31.2)	-2.9 (-23 to 14.1)	33.7 (10.3 to 58.6)
		Diet high in sodium	1.4 (0.2 to 5.6)	1.3 (0.3 to 4.9)	1.6 (0.2 to 6.5)	2.1 (0.3 to 8)	1.6 (0.4 to 6.2)	2.8 (0.3 to 10.8)	47.8 (-12.3 to 130.7)	22.2 (-35.4 to 145)	68.8 (11.6 to 186.6)
		High fasting plasma glucose	24.4 (17.4 to 33.5)	25.6 (17.9 to 35.4)	23.1 (15.9 to 32.4)	36.3 (25.2 to 51.2)	34.1 (23.6 to 47.3)	39 (26.6 to 56.8)	48.8 (33.6 to 65.5)	33.3 (15.3 to 51.8)	68.9 (45.6 to 98.1)
		High body-mass index	32.2 (16.5 to 52.5)	41.7 (22.2 to 66.8)	20.8 (9.4 to 36.9)	54.2 (30.4 to 85.8)	60.8 (34.1 to 94.5)	46.7 (24.2 to 76.3)	68.4 (44.1 to 107.1)	45.6 (21.9 to 83)	124.9 (80.9 to 212.2)
		High systolic blood pressure	51.5 (35.9 to 68.5)	57 (39.1 to 78.4)	44.9 (30.5 to 61.4)	74.2 (52.4 to 101.4)	71.7 (48.9 to 99)	77.1 (52 to 107.5)	44 (26.9 to 63.2)	25.9 (5.2 to 52.6)	71.8 (46 to 101.1)
		Kidney dysfunction	123 (90.2 to 161.3)	143.2 (104.5 to 188.6)	100.4 (71.9 to 132.6)	168.5 (124.5 to 224.1)	177.9 (131.7 to 232.1)	159.1 (112.7 to 219)	37 (26.1 to 49.1)	24.3 (11.7 to 37.6)	58.5 (41 to 78.3)
Qatar	Deaths	Non-optimal temperature	4.4 (-9.4 to 14.1)	5 (-10 to 17.4)	3.9 (-8.8 to 12.7)	4.4 (-4.4 to 11.4)	7 (-7 to 18)	3.7 (-3.7 to 9.4)	1.4 (-178.8 to 124.5)	38.1 (-228.9 to 221.8)	-7.3 (-174.4 to 101.5)
		Lead exposure	0.8 (0.1 to 1.8)	0.9 (0.1 to 2.2)	0.9 (0.2 to 1.8)	1 (0.3 to 1.8)	1.3 (0.2 to 2.5)	0.9 (0.3 to 1.6)	15.1 (-27.4 to 138.1)	44.9 (-24.5 to 223.7)	3.1 (-30.1 to 84)
		Diet high in sodium	0.7 (0.1 to 3)	0.6 (0.1 to 2.6)	0.8 (0.1 to 3.8)	0.7 (0.1 to 2.9)	0.7 (0.2 to 2.9)	0.7 (0.1 to 2.9)	0.7 (-48.6 to 89.5)	17.6 (-48.5 to 175.6)	-16.4 (-58.8 to 115.9)
		High fasting plasma glucose	17.9 (13.2 to 30.6)	20.5 (14.1 to 39.8)	16.6 (11.6 to 25.9)	16.5 (12.5 to 21.7)	25.1 (18.3 to 33.1)	13.7 (10 to 18.3)	-7.8 (-41.8 to 25.3)	22.6 (-35.6 to 76.1)	-17.6 (-44.8 to 17.5)
		High body-mass index	19.7 (10.9 to 35.9)	25.5 (14.2 to 49.7)	15.3 (7.5 to 27)	20.7 (9.9 to 31.9)	34.2 (15.6 to 52.6)	16.3 (7 to 26.4)	4.9 (-40.9 to 52.1)	34.3 (-35.1 to 102.8)	7.1 (-39 to 67.6)
		High systolic blood pressure	32.7 (24.8 to 56.4)	36.9 (25.9 to 71.2)	29.8 (21.6 to 45.2)	32.3 (25.4 to 40.7)	49.7 (38.3 to 62.6)	26.8 (20.4 to 35.1)	-1.4 (-38.8 to 35.5)	34.7 (-29.2 to 93.8)	-10.3 (-39.2 to 27.5)

Location	Measure	Risk factor	Attributed age-standardized rate (per 100,000)						% Change (1990 to 2019)		
			1990			2019					
			Both	Female	Male	Both	Female	Male	Both	Female	Male
		Kidney dysfunction	48.9 (38.4 to 84.3)	56 (40.5 to 107.6)	44.2 (32.8 to 65.7)	45.9 (37.1 to 56)	71.8 (57.6 to 87.5)	37.7 (29.2 to 47.5)	-6.2 (-41.8 to 28.1)	28.3 (-32.2 to 81.9)	-14.7 (-41.5 to 20.2)
	DALYs	Non-optimal temperature	72.1 (-160.8 to 222.6)	87.7 (-177.5 to 293)	63.1 (-142.8 to 195.5)	61.1 (-60.6 to 159.3)	96.7 (-93.2 to 250.7)	49.2 (-49.7 to 127.1)	-15.3 (-167.1 to 91.4)	10.3 (-201.3 to 158.5)	-22 (-160.2 to 71.6)
		Lead exposure	14.7 (2.3 to 31.3)	15.8 (0.9 to 38)	14.4 (2.8 to 30.2)	14.3 (3.5 to 27.6)	17.6 (2.4 to 37)	13.2 (3.8 to 24.6)	-3.2 (-33.8 to 81.3)	12 (-36.4 to 186.7)	-8.3 (-33.9 to 55.3)
		Diet high in sodium	14 (2.1 to 58.2)	12.3 (2.5 to 52.1)	15.6 (1.5 to 70.5)	12.9 (1.9 to 50.8)	12.3 (3 to 49.6)	13 (1.3 to 52.5)	-7.8 (-49.2 to 64.6)	-0.5 (-53.1 to 130.5)	-16.8 (-53.2 to 75)
		High fasting plasma glucose	332 (250.6 to 519.8)	402 (286.7 to 719.4)	296.7 (217.5 to 430.4)	276.2 (214.9 to 354.4)	404.3 (309.4 to 525.5)	232.9 (176.9 to 304.5)	-16.8 (-43.9 to 10.9)	0.6 (-41.6 to 39.9)	-21.5 (-44.4 to 8.2)
		High body-mass index	399.9 (241 to 655.7)	546.8 (337.5 to 964.8)	308.9 (173.4 to 496.3)	388.5 (245.5 to 547.9)	603.7 (376.6 to 851.8)	316.4 (193.1 to 456)	-2.9 (-35.5 to 34.4)	10.4 (-37.1 to 56.4)	2.4 (-29.9 to 50.1)
		High systolic blood pressure	566.4 (437 to 887.9)	673.2 (487.4 to 1201.5)	500.9 (372.7 to 709)	514.9 (417.7 to 637.9)	747.2 (582.5 to 938.1)	437.4 (343.3 to 553.6)	-9.1 (-39.1 to 21.8)	11 (-34.7 to 56.4)	-12.7 (-38 to 19.8)
		Kidney dysfunction	927.8 (751.6 to 1439.5)	1133.4 (864.8 to 1960)	809.8 (625.9 to 1128.6)	803.6 (662.8 to 963.8)	1190.8 (971 to 1450.5)	675.8 (542.9 to 838.3)	-13.4 (-40.4 to 12)	5.1 (-36.6 to 40.3)	-16.5 (-38.9 to 11.6)
	YLLs	Non-optimal temperature	72.1 (-160.8 to 222.6)	87.7 (-177.5 to 293)	63.1 (-142.8 to 195.5)	61.1 (-60.6 to 159.3)	96.7 (-93.2 to 250.7)	49.2 (-49.7 to 127.1)	-15.3 (-167.1 to 91.4)	10.3 (-201.3 to 158.5)	-22 (-160.2 to 71.6)
		Lead exposure	13.1 (2 to 28.3)	14 (0.8 to 34.6)	12.9 (2.6 to 27.3)	12 (3.2 to 23)	15.9 (2.3 to 32.8)	10.7 (3.3 to 20.2)	-8.7 (-39.1 to 75.5)	13.6 (-38.4 to 202.7)	-16.9 (-41.6 to 48.5)
		Diet high in sodium	12.3 (1.8 to 52.3)	10.8 (2.2 to 46.1)	13.9 (1.3 to 63.7)	10.1 (1.5 to 42)	10.5 (2.5 to 43)	9.9 (1 to 41.8)	-18 (-54.7 to 55)	-2.9 (-56 to 134.4)	-28.7 (-61.6 to 61)
		High fasting plasma glucose	305 (223.8 to 490)	370.9 (258.1 to 684.2)	272 (192.8 to 409.2)	234.4 (175.4 to 311.3)	364.9 (267.8 to 483.5)	190 (136.9 to 257)	-23.2 (-48.5 to 5.4)	-1.6 (-45 to 42.2)	-30.1 (-52 to 0.5)

Location	Measure	Risk factor	Attributed age-standardized rate (per 100,000)						% Change (1990 to 2019)		
			1990			2019					
			Both	Female	Male	Both	Female	Male	Both	Female	Male
		High body-mass index	352.6 (208.6 to 585.3)	479.6 (287.9 to 890.8)	273.4 (150.7 to 450.3)	307.8 (183.9 to 447.1)	513.9 (300.1 to 736.8)	238.4 (136.6 to 355.3)	-12.7 (-46.2 to 27.2)	7.1 (-42.5 to 61.6)	-12.8 (-44.1 to 33.1)
		High systolic blood pressure	506.5 (381.1 to 819)	600.2 (416.4 to 1122)	449.9 (326.6 to 655)	424.7 (330.6 to 534.9)	662 (504.4 to 847.9)	345.5 (258.6 to 451.1)	-16.1 (-45.2 to 17)	10.3 (-38.6 to 62.5)	-23.2 (-47.5 to 10.7)
		Kidney dysfunction	807 (639 to 1312.1)	977 (711.9 to 1797.8)	710.2 (529.3 to 1010.3)	629.6 (503.5 to 778.1)	996.7 (791.5 to 1246.5)	508.1 (385.9 to 655)	-22 (-48.8 to 5.9)	2 (-42.5 to 44.4)	-28.5 (-49.9 to 1.4)
	YLDs	Lead exposure	1.6 (0.2 to 3.5)	1.8 (0.1 to 4.1)	1.5 (0.3 to 3.2)	2.3 (0.5 to 5.1)	1.8 (0.1 to 4.5)	2.5 (0.6 to 5.4)	42.2 (19.2 to 141.8)	-0.5 (-26.7 to 79.2)	67.4 (38.3 to 149.6)
		Diet high in sodium	1.7 (0.2 to 6.6)	1.5 (0.3 to 6)	1.8 (0.2 to 7.1)	2.8 (0.3 to 10.8)	1.8 (0.4 to 7)	3.1 (0.3 to 12.2)	67.4 (-7.7 to 143.9)	16.9 (-37 to 137.1)	77.1 (12.7 to 207.8)
		High fasting plasma glucose	26.9 (19 to 37.6)	31.1 (21.8 to 42.8)	24.7 (16.9 to 35.4)	41.8 (28.2 to 60.4)	39.4 (27.8 to 55.3)	42.9 (28 to 63.2)	55.3 (35.9 to 76)	26.7 (9.7 to 45.2)	73.3 (48.2 to 101.5)
		High body-mass index	47.3 (27.6 to 73)	67.2 (41 to 101.8)	35.5 (19.4 to 57.1)	80.7 (49.2 to 119.6)	89.8 (55.6 to 131.4)	77.9 (46.4 to 118.6)	70.5 (45.2 to 103.1)	33.6 (14.1 to 57)	119.5 (80.7 to 175.8)
		High systolic blood pressure	59.9 (42 to 81.7)	72.9 (50.1 to 99.7)	51 (35.6 to 70.9)	90.2 (62.4 to 125.7)	85.2 (59.3 to 117.5)	91.9 (61.9 to 131.3)	50.5 (30.9 to 73)	16.7 (-2.4 to 39.8)	80.3 (56.2 to 109.2)
		Kidney dysfunction	120.8 (87.3 to 163.6)	156.5 (114.2 to 206.6)	99.5 (70.5 to 136)	173.9 (124.7 to 233.6)	194.1 (141.4 to 249.5)	167.7 (116 to 229.3)	44 (28.6 to 59.7)	24.1 (10.5 to 38.3)	68.5 (49.4 to 90)
Saudi Arabia	Deaths	Non-optimal temperature	3.2 (-1.5 to 6.5)	2.9 (-1.3 to 5.9)	3.5 (-1.6 to 7.3)	3.9 (-0.8 to 7.7)	4 (-0.9 to 7.9)	3.9 (-0.9 to 7.7)	23.3 (-132 to 200.6)	37.7 (-135 to 239.3)	12.1 (-129.7 to 170)
		Lead exposure	2.1 (1.2 to 3.2)	1.3 (0.6 to 2.1)	2.8 (1.7 to 4.2)	2.1 (1.2 to 3.2)	1.4 (0.6 to 2.5)	2.6 (1.6 to 3.9)	0.1 (-30.3 to 40.2)	9.4 (-27.7 to 52.2)	-6.5 (-38.6 to 38.7)
		Diet high in sodium	0.8 (0.1 to 3.2)	0.5 (0.1 to 1.9)	1 (0.1 to 4.3)	0.9 (0.1 to 3.6)	0.6 (0.1 to 2.3)	1.1 (0.1 to 4.5)	12.4 (-38.5 to 113.3)	22.4 (-47.2 to 184.5)	4.9 (-45.9 to 145.3)

Location	Measure	Risk factor	Attributed age-standardized rate (per 100,000)						% Change (1990 to 2019)		
			1990			2019					
			Both	Female	Male	Both	Female	Male	Both	Female	Male
		High fasting plasma glucose	16.7 (12.1 to 22.7)	14.9 (10.9 to 20.4)	18.5 (12.9 to 25.6)	18.1 (13.4 to 23.7)	18.1 (12.9 to 24.6)	18.3 (13.2 to 24.4)	8.4 (-24.5 to 49.9)	21.3 (-20.6 to 70.4)	-1.1 (-35.2 to 48.8)
		High body-mass index	15.6 (8.8 to 24.2)	16.7 (9.9 to 24.9)	14.8 (7.4 to 24.2)	25 (15 to 35.5)	27.4 (17.1 to 39.6)	23.4 (13.5 to 34.6)	60.6 (9.8 to 145)	63.7 (6.8 to 146.6)	57.8 (-2.2 to 164.2)
		High systolic blood pressure	30.2 (23.4 to 38)	26.2 (20 to 33.7)	34.2 (25.6 to 44.5)	35.3 (27.8 to 43.6)	33.2 (24.4 to 43)	37 (29 to 46)	17 (-16.9 to 60.1)	26.9 (-17.2 to 81.8)	8.3 (-27.6 to 61.9)
		Kidney dysfunction	47.9 (37.7 to 59.4)	43.5 (34.6 to 54.4)	52.4 (39.2 to 67.2)	52.9 (42.8 to 63.1)	53.5 (40.3 to 68.1)	52.7 (42.4 to 64.6)	10.4 (-20.8 to 50.2)	22.8 (-18 to 69.8)	0.6 (-33.1 to 47.6)
	DALYs	Non-optimal temperature	65.3 (-31 to 136.3)	62.6 (-29.5 to 131)	68.2 (-32.3 to 145.5)	74.1 (-16.2 to 146.4)	78.3 (-16.4 to 154.4)	71.4 (-16.2 to 140.9)	13.5 (-129.5 to 172.8)	25.1 (-131.8 to 202)	4.7 (-127.8 to 165.4)
		Lead exposure	44.4 (25.1 to 67.2)	29.2 (12.5 to 49)	56.2 (33.7 to 84)	38.5 (20.1 to 60)	26.4 (9.5 to 47.8)	46.9 (27.1 to 70.5)	-13.4 (-40.1 to 18.1)	-9.7 (-41.3 to 23.2)	-16.6 (-44.8 to 21.4)
		Diet high in sodium	17.3 (2.5 to 70.8)	11.2 (2.5 to 45.5)	22 (2.1 to 91.6)	19.4 (2.8 to 76.7)	13.2 (3 to 52.2)	23.6 (2.3 to 97)	12.1 (-35.3 to 101.8)	17.4 (-49.3 to 175.3)	7.4 (-39 to 120.3)
		High fasting plasma glucose	379.5 (275.1 to 511.7)	361.4 (266.1 to 490.1)	397.6 (278.1 to 547.4)	406.9 (307.8 to 528.8)	420.8 (310.1 to 556.4)	398.8 (297.3 to 522.6)	7.2 (-24.4 to 47.1)	16.5 (-22.4 to 63.4)	0.3 (-32.9 to 46.6)
		High body-mass index	382.1 (229.8 to 570.1)	433.7 (265.6 to 627.1)	348.4 (185.4 to 543)	590.9 (396.6 to 799.5)	664.1 (449 to 913.4)	543.1 (355.5 to 748.6)	54.7 (7.7 to 125.7)	53.1 (6.6 to 124.4)	55.9 (1.3 to 154.7)
		High systolic blood pressure	623.7 (481.9 to 793.1)	579.2 (443 to 750.6)	665.5 (495.8 to 861.7)	711.5 (565.8 to 866.5)	684.3 (510.2 to 879.3)	730.6 (571.2 to 912.7)	14.1 (-17.6 to 53.2)	18.1 (-20.1 to 65)	9.8 (-26.1 to 58.2)
		Kidney dysfunction	1106.8 (880.7 to 1366.7)	1095.8 (868.4 to 1360.3)	1128.8 (861 to 1447.5)	1197.7 (982.8 to 1421.3)	1272.1 (1002.2 to 1585.5)	1149.3 (931.2 to 1396.5)	8.2 (-20.7 to 42.6)	16.1 (-18.6 to 56.7)	1.8 (-29.9 to 45.6)
	YLLs	Non-optimal temperature	65.3 (-31 to 136.3)	62.6 (-29.5 to 131)	68.2 (-32.3 to 145.5)	74.1 (-16.2 to 146.4)	78.3 (-16.4 to 154.4)	71.4 (-16.2 to 140.9)	13.5 (-129.5 to 172.8)	25.1 (-131.8 to 202)	4.7 (-127.8 to 165.4)

Location	Measure	Risk factor	Attributed age-standardized rate (per 100,000)						% Change (1990 to 2019)		
			1990			2019					
			Both	Female	Male	Both	Female	Male	Both	Female	Male
		Lead exposure	40.5 (22.7 to 61.6)	25.7 (11 to 43.1)	52 (30.9 to 78.7)	33.5 (17.3 to 53.6)	23 (8.3 to 42.1)	40.9 (23.3 to 62.1)	-17.3 (-44.1 to 17.8)	-10.6 (-44.1 to 26.5)	-21.4 (-49.2 to 19.2)
		Diet high in sodium	15.8 (2.2 to 65.9)	9.8 (2.2 to 40)	20.3 (1.9 to 87.1)	16.6 (2.3 to 67.9)	11.2 (2.5 to 45)	20.2 (2 to 83.9)	5.3 (-41.4 to 96.9)	13.9 (-53.3 to 175.2)	-0.3 (-45.5 to 112.8)
		High fasting plasma glucose	353.6 (251.6 to 486.7)	332.7 (237.2 to 462)	373.6 (252.6 to 525.1)	361.6 (262.8 to 472.4)	377.5 (264.6 to 507.5)	352.1 (254.9 to 473.7)	2.3 (-29.5 to 43.9)	13.5 (-27.1 to 62.9)	-5.7 (-39.3 to 41.8)
		High body-mass index	344.9 (203.6 to 526.1)	381.8 (228.6 to 563.2)	321.6 (169.4 to 511.5)	506.1 (329.9 to 694.2)	568.6 (379.3 to 793.1)	465.3 (293.4 to 657.9)	46.7 (-0.7 to 122.1)	48.9 (-2.2 to 128.1)	44.7 (-8 to 146)
		High systolic blood pressure	569.6 (427.4 to 734.7)	516.3 (384.1 to 680)	618 (450.6 to 813.2)	617.2 (479.6 to 768)	595 (433.4 to 778.6)	633.2 (491.6 to 807.3)	8.4 (-23.9 to 49.9)	15.3 (-25.5 to 68.5)	2.5 (-32.5 to 54.7)
		Kidney dysfunction	988.8 (767.8 to 1249.5)	949.9 (739.6 to 1206.3)	1031.2 (762 to 1341.4)	1003.5 (798.4 to 1220.6)	1059.6 (802.4 to 1361.2)	967.8 (766 to 1217)	1.5 (-28.7 to 39.7)	11.5 (-26.3 to 58.6)	-6.1 (-38 to 41.3)
	YLDs	Lead exposure	3.9 (2.1 to 6.5)	3.5 (1.3 to 6.3)	4.2 (2.4 to 6.6)	4.9 (2.3 to 8.7)	3.4 (1.1 to 6.6)	6 (3 to 10.3)	26.5 (4.2 to 47.4)	-3.1 (-28.2 to 16.3)	42.5 (14.9 to 69.5)
		Diet high in sodium	1.6 (0.2 to 6.1)	1.4 (0.3 to 5.2)	1.7 (0.2 to 6.8)	2.8 (0.4 to 10.9)	1.9 (0.4 to 7.7)	3.4 (0.3 to 13.2)	80.7 (7.3 to 170.2)	42.3 (-27.6 to 184.5)	99.6 (26.3 to 259.1)
		High fasting plasma glucose	25.9 (18.6 to 35.9)	28.7 (20.6 to 39.3)	24.1 (16.3 to 34.1)	45.3 (30.9 to 63.6)	43.3 (30.1 to 59.5)	46.6 (31.3 to 67.5)	74.6 (55.1 to 96.7)	50.9 (30.1 to 75.5)	93.8 (67.3 to 123.4)
		High body-mass index	37.1 (20.9 to 58.4)	51.9 (30 to 80.3)	26.9 (13.8 to 43.4)	84.8 (52.2 to 125.1)	95.5 (58.7 to 138)	77.8 (46.4 to 117.7)	128.2 (89 to 192.3)	84 (52 to 134.1)	189.5 (125.8 to 303.1)
		High systolic blood pressure	54.1 (37.6 to 74.1)	63 (42.9 to 87.4)	47.6 (32.7 to 65.3)	94.3 (65.1 to 130.5)	89.3 (61.9 to 122.9)	97.4 (66.3 to 137.2)	74.3 (52 to 98.9)	41.8 (17.9 to 71.9)	104.8 (76.4 to 138.9)
		Kidney dysfunction	117.9 (85.6 to 153.8)	145.9 (106.3 to 189.8)	97.7 (70.6 to 129.1)	194.2 (140.8 to 258.3)	212.5 (157.5 to 281.6)	181.6 (126.9 to 245.8)	64.7 (49.7 to 81.6)	45.7 (29.7 to 64)	85.9 (64.8 to 109.2)

Location	Measure	Risk factor	Attributed age-standardized rate (per 100,000)						% Change (1990 to 2019)		
			1990			2019					
			Both	Female	Male	Both	Female	Male	Both	Female	Male
Sudan	Deaths	Non-optimal temperature	1.4 (-2.6 to 3.8)	1.4 (-2.4 to 3.8)	1.5 (-2.8 to 4.1)	1.8 (-1 to 4.1)	1.7 (-0.9 to 4.1)	1.8 (-1.1 to 4.9)	22.2 (-220.8 to 148.8)	26.5 (-215.1 to 150)	17.6 (-225.4 to 158.1)
		Lead exposure	2.2 (1.5 to 3.6)	1.7 (1 to 2.9)	2.7 (1.8 to 4.6)	2.5 (1.5 to 4.2)	1.9 (1.2 to 3)	2.9 (1.7 to 5.5)	10.3 (-24.2 to 66.1)	12.7 (-34 to 58.7)	6.5 (-29 to 79.6)
		Diet high in sodium	0.4 (0.1 to 1.9)	0.3 (0.1 to 1.2)	0.6 (0.1 to 2.6)	0.5 (0.1 to 1.9)	0.3 (0.1 to 1.3)	0.6 (0 to 2.5)	2.7 (-46.1 to 107.9)	4.3 (-58.9 to 136.9)	-0.8 (-53.7 to 147.8)
		High fasting plasma glucose	8.4 (5.6 to 12.3)	7.7 (4.9 to 12.3)	9.1 (6.1 to 14.2)	8.8 (5.5 to 13.9)	8.4 (5.2 to 12.3)	9.2 (5.3 to 16.7)	5.3 (-30.3 to 58.1)	8.6 (-36.7 to 55.4)	1.7 (-33.7 to 74.9)
		High body-mass index	5.3 (2.4 to 10)	6.4 (3.1 to 11.7)	4.2 (1.5 to 9)	9.6 (5.1 to 16.7)	10.4 (5.5 to 16.9)	8.9 (4 to 18.7)	81.8 (10.1 to 217.2)	62 (-11 to 168.3)	109.6 (21.1 to 379.8)
		High systolic blood pressure	18.9 (14.3 to 27.7)	17.7 (12.7 to 28.2)	20.1 (14.8 to 31.7)	20.3 (14.3 to 31.8)	19.9 (13.1 to 29.5)	20.8 (13.3 to 37.3)	7.7 (-28.3 to 58.3)	12.2 (-35.2 to 59.9)	3.1 (-31 to 74.4)
		Kidney dysfunction	28.5 (22.1 to 40.3)	26.8 (19.8 to 40.7)	30.4 (23.2 to 45.5)	28.4 (20.3 to 44.3)	27.4 (18 to 40.7)	29.4 (19.1 to 51.8)	-0.5 (-33.1 to 45.8)	2 (-40.6 to 41.1)	-3.4 (-35.9 to 60.7)
	DALYs	Non-optimal temperature	32.9 (-60.8 to 84.5)	32.9 (-59.6 to 86.1)	32.9 (-58.9 to 85.8)	35.9 (-20.9 to 84.7)	35.9 (-19.3 to 84.4)	35.9 (-21.7 to 93.7)	9.1 (-207.9 to 125.3)	9 (-205.2 to 141)	9 (-215.8 to 141.6)
		Lead exposure	48.8 (32.7 to 73.2)	39.8 (24.7 to 63.7)	57.3 (38 to 89.5)	49.3 (31 to 80.9)	40.1 (24.9 to 61)	57.1 (34.1 to 101.1)	1 (-27.6 to 47.4)	0.8 (-36.5 to 37.2)	-0.3 (-30.9 to 59.7)
		Diet high in sodium	10.3 (1.5 to 42.7)	7.3 (1.5 to 30.2)	13.1 (1.2 to 55.2)	10.8 (1.5 to 43.3)	7.6 (1.6 to 31.3)	13.5 (1.1 to 54.8)	4.3 (-43.7 to 96.1)	3.4 (-53.3 to 122.6)	3.1 (-44 to 135.5)
		High fasting plasma glucose	198.3 (139.6 to 280.7)	192.8 (127.7 to 287.6)	203.5 (139.5 to 303.3)	207 (140 to 311.3)	202.8 (133.9 to 285.8)	210.2 (131 to 352.2)	4.4 (-29 to 50.1)	5.2 (-35.5 to 48.5)	3.3 (-30 to 70.3)
		High body-mass index	139.9 (66.8 to 242.1)	177.6 (92.7 to 304.3)	106.1 (39.5 to 206.3)	242.9 (141.3 to 386)	273.1 (165.2 to 409.8)	217.1 (107.7 to 397)	73.7 (9.2 to 190.3)	53.8 (-9.4 to 143.3)	104.7 (24 to 344.5)

Location	Measure	Risk factor	Attributed age-standardized rate (per 100,000)						% Change (1990 to 2019)		
			1990			2019					
			Both	Female	Male	Both	Female	Male	Both	Female	Male
		High systolic blood pressure	406.6 (312.6 to 556.8)	408.1 (300.2 to 606.1)	406.3 (307.2 to 609.2)	441.1 (319.9 to 648)	450 (313.5 to 616.3)	433.6 (291.9 to 712.8)	8.5 (-25.2 to 53.1)	10.2 (-31.2 to 52)	6.7 (-26.2 to 71.6)
		Kidney dysfunction	738 (596 to 942.2)	760.2 (580.2 to 1037.6)	717.1 (571.7 to 950.4)	717.3 (535 to 1013.1)	729.7 (534.3 to 974.2)	704.4 (494.2 to 1139.2)	-2.8 (-31.4 to 36.2)	-4 (-37.9 to 28.1)	-1.8 (-30.6 to 54.7)
	YLLs	Non-optimal temperature	32.9 (-60.8 to 84.5)	32.9 (-59.6 to 86.1)	32.9 (-58.9 to 85.8)	35.9 (-20.9 to 84.7)	35.9 (-19.3 to 84.4)	35.9 (-21.7 to 93.7)	9.1 (-207.9 to 125.3)	9 (-205.2 to 141)	9 (-215.8 to 141.6)
		Lead exposure	43.7 (28.7 to 67.7)	34.5 (20.8 to 57.1)	52.4 (34.2 to 84)	41.1 (25.1 to 70.8)	32.8 (19.4 to 52.3)	48.2 (27.1 to 89.3)	-6 (-36.9 to 44.4)	-4.9 (-45.2 to 37)	-8.1 (-40 to 58.5)
		Diet high in sodium	9.3 (1.3 to 38.4)	6.3 (1.2 to 26.4)	12 (1.1 to 51.1)	8.9 (1.2 to 37.1)	6.1 (1.3 to 25.7)	11.3 (0.9 to 47.9)	-3.9 (-49.9 to 90)	-3.4 (-60.1 to 122.2)	-5.7 (-51.4 to 128.2)
		High fasting plasma glucose	182.3 (125.6 to 264.3)	175.1 (112.1 to 271)	189.1 (127.1 to 290.2)	179.1 (111.7 to 278.2)	175.6 (107.6 to 257)	181.8 (105 to 321)	-1.8 (-36.6 to 47.4)	0.2 (-42.2 to 47.5)	-3.8 (-38.9 to 68.1)
		High body-mass index	124.2 (58 to 221.5)	154.7 (78 to 272.2)	97 (34.8 to 194.3)	200.3 (108.3 to 337.5)	222.1 (125.2 to 351.9)	182 (84 to 353.4)	61.3 (-3.1 to 180.3)	43.6 (-22.7 to 138.8)	87.6 (7.5 to 326.7)
		High systolic blood pressure	365.2 (274.5 to 514.2)	357.2 (250.6 to 552.3)	373.9 (277.4 to 570.4)	367 (254.2 to 564.8)	368.2 (237.8 to 534)	366.7 (231.8 to 644.8)	0.5 (-34.9 to 50.2)	3.1 (-41.1 to 49.7)	-1.9 (-36.7 to 68.3)
		Kidney dysfunction	653.8 (517.3 to 856.4)	656.9 (484.8 to 928)	651.8 (504 to 882.3)	579.7 (409.1 to 865.6)	575.2 (379.3 to 812.6)	583.2 (379.8 to 1005.4)	-11.3 (-41.1 to 32)	-12.4 (-49 to 23.4)	-10.5 (-40.9 to 51.7)
	YLDs	Lead exposure	5.1 (3.1 to 7.6)	5.3 (3.2 to 8.2)	4.9 (3.1 to 7.2)	8.2 (5 to 12.5)	7.3 (4.3 to 11.3)	8.9 (5.5 to 13.8)	61.1 (43.9 to 81.9)	38.2 (20.9 to 56.6)	83 (55.8 to 114.7)
		Diet high in sodium	1.1 (0.2 to 4.2)	1 (0.2 to 3.9)	1.1 (0.1 to 4.5)	1.9 (0.3 to 7.2)	1.5 (0.3 to 5.6)	2.2 (0.2 to 8.7)	75.3 (-1.1 to 177.5)	46.3 (-26.8 to 174.9)	96.7 (23.1 to 258.4)
		High fasting plasma glucose	16 (11.1 to 22.3)	17.6 (12.3 to 24.5)	14.5 (9.7 to 20.5)	28 (19.4 to 39)	27.2 (19.1 to 37.6)	28.4 (18.9 to 41.4)	74.8 (55.9 to 95)	54.6 (34.4 to 77.9)	96.5 (66.5 to 129.4)

Location	Measure	Risk factor	Attributed age-standardized rate (per 100,000)						% Change (1990 to 2019)		
			1990			2019					
			Both	Female	Male	Both	Female	Male	Both	Female	Male
		High body-mass index	15.7 (7.2 to 27.4)	22.9 (11 to 38.9)	9.1 (3.3 to 17.9)	42.7 (23.5 to 67.6)	51 (28.8 to 80.2)	35.2 (18.4 to 57.2)	172.1 (115.3 to 303.5)	122.9 (71.3 to 219.8)	288.1 (179.5 to 640.7)
		High systolic blood pressure	41.5 (28.7 to 55.9)	50.9 (35.4 to 69.6)	32.4 (22.1 to 44.8)	74.1 (52.1 to 102.5)	81.8 (57.2 to 112.8)	67 (46.3 to 94.4)	78.6 (60.5 to 99.3)	60.8 (38.1 to 85.9)	106.8 (79.9 to 140.8)
		Kidney dysfunction	84.2 (61.3 to 111.7)	103.3 (74.4 to 139.3)	65.3 (47.1 to 87.5)	137.6 (100 to 183.3)	154.5 (112.6 to 201.8)	121.2 (86.7 to 164.9)	63.5 (51.2 to 77.2)	49.5 (35.4 to 64.9)	85.6 (65.4 to 108.6)
Syrian Arab Republic	Deaths	Non-optimal temperature	2.9 (1.8 to 4.1)	3 (1.8 to 4.2)	2.9 (1.8 to 4.2)	2.3 (1.4 to 3.3)	2.6 (1.7 to 3.7)	2.1 (1.3 to 3)	-21.7 (-45 to 9.2)	-11.1 (-37 to 22.9)	-27 (-52.5 to 10.2)
		Lead exposure	1.8 (1.1 to 2.7)	1.5 (0.9 to 2.3)	2.1 (1.3 to 3.2)	1.5 (0.9 to 2.1)	1.4 (0.8 to 2.1)	1.6 (1 to 2.2)	-20.2 (-43.6 to 8.4)	-7.4 (-35.2 to 24.3)	-26.4 (-52.5 to 7.5)
		Diet high in sodium	0.5 (0.1 to 2.2)	0.4 (0.1 to 1.5)	0.7 (0.1 to 3)	0.4 (0.1 to 1.7)	0.3 (0.1 to 1.3)	0.5 (0.1 to 2)	-23.9 (-54.7 to 42.4)	-15.7 (-57.8 to 67.1)	-27.9 (-65.1 to 56.9)
		High fasting plasma glucose	11.6 (8.6 to 15.4)	11.3 (8.4 to 15.2)	11.8 (8.5 to 16.4)	8.7 (6.3 to 11.7)	9.5 (6.7 to 12.8)	8.3 (5.9 to 11.5)	-24.9 (-47.2 to 3.1)	-16.5 (-41.6 to 13.9)	-29.5 (-54.6 to 3.8)
		High body-mass index	10.8 (5.9 to 16.9)	12.7 (7.1 to 19.5)	9 (4.3 to 15.7)	10.2 (5.7 to 15.7)	12.8 (6.8 to 19.7)	8.6 (4.4 to 13.8)	-5 (-33.8 to 40.5)	0.2 (-32.4 to 46.8)	-4.6 (-39.9 to 56.8)
		High systolic blood pressure	22.9 (18.2 to 29.3)	23.2 (17.9 to 30.7)	22.6 (17 to 30.9)	18.2 (13.8 to 22.9)	21 (16 to 26.7)	16.8 (12.4 to 21.7)	-20.4 (-43.6 to 7.1)	-9.4 (-36.6 to 24.5)	-25.7 (-50.8 to 6.9)
		Kidney dysfunction	37 (30.4 to 46.1)	37.3 (30.2 to 47.5)	36.7 (28.9 to 48.7)	27.8 (22.1 to 34.6)	31.8 (25.1 to 39.3)	25.7 (19.6 to 32.7)	-24.9 (-46.2 to 1.1)	-14.8 (-39.5 to 14.8)	-30 (-53.6 to 0.6)
	DALYs	Non-optimal temperature	66.4 (41.3 to 93.9)	66 (41.2 to 94.5)	66.6 (41.3 to 95.1)	44.2 (27.9 to 63.8)	47.3 (30.2 to 68.5)	42.4 (26.4 to 61.5)	-33.3 (-52.9 to -5.4)	-28.4 (-50.1 to 3.8)	-36.3 (-57.2 to -4.6)
		Lead exposure	38.6 (23.4 to 57)	31.4 (16.8 to 48.4)	45.1 (28.1 to 67.1)	26.9 (15.6 to 39.4)	23.7 (12.5 to 36.5)	30.3 (18.3 to 44.3)	-30.2 (-48.8 to -7.3)	-24.3 (-43.6 to -0.6)	-32.8 (-53.9 to -6.2)

Location	Measure	Risk factor	Attributed age-standardized rate (per 100,000)						% Change (1990 to 2019)		
			1990			2019					
			Both	Female	Male	Both	Female	Male	Both	Female	Male
		Diet high in sodium	12.5 (2 to 50.2)	9 (2.1 to 35.2)	15.8 (1.6 to 66.6)	9.5 (1.5 to 37.7)	7.1 (1.7 to 30.3)	11.8 (1.2 to 45.7)	-24.3 (-52.7 to 33.3)	-20.9 (-60.7 to 55.7)	-25.1 (-55.7 to 45.6)
		High fasting plasma glucose	273.1 (204.7 to 353.9)	268.6 (200.7 to 352.6)	276.9 (201.8 to 368.2)	200.8 (150 to 264.3)	205.8 (152 to 275.7)	198.5 (146.1 to 264.9)	-26.5 (-46.3 to 0)	-23.4 (-44.2 to 4.4)	-28.3 (-50.1 to 2.2)
		High body-mass index	269.5 (155.8 to 401.8)	317.5 (190.4 to 464.5)	226.1 (113.6 to 365.3)	254.5 (153.7 to 361.5)	295.8 (183.6 to 411.1)	220.9 (126.8 to 331.3)	-5.6 (-30.5 to 36)	-6.8 (-32.1 to 31.4)	-2.3 (-33.8 to 56.3)
		High systolic blood pressure	476.7 (379.1 to 601.5)	482.8 (375 to 614.1)	471.3 (361.1 to 619.7)	376.9 (292.3 to 475.5)	404.4 (312.6 to 510.9)	360.5 (277.2 to 460.7)	-20.9 (-42 to 5.1)	-16.2 (-38.5 to 13.8)	-23.5 (-46.8 to 7.6)
		Kidney dysfunction	955.1 (803.1 to 1143.6)	979.6 (803.1 to 1178.6)	931.7 (761.7 to 1132.9)	684.9 (556.8 to 836.8)	737.2 (600.5 to 900.7)	646.1 (511.2 to 805.6)	-28.3 (-44.4 to -6.3)	-24.7 (-42.3 to -1.2)	-30.7 (-49.3 to -5.1)
	YLLs	Non-optimal temperature	66.4 (41.3 to 93.9)	66 (41.2 to 94.5)	66.6 (41.3 to 95.1)	44.2 (27.9 to 63.8)	47.3 (30.2 to 68.5)	42.4 (26.4 to 61.5)	-33.3 (-52.9 to -5.4)	-28.4 (-50.1 to 3.8)	-36.3 (-57.2 to -4.6)
		Lead exposure	34.3 (20.6 to 51.4)	27 (14.5 to 41.8)	41 (25.1 to 62.1)	21.9 (12.6 to 32.8)	19.4 (10.3 to 30.6)	24.7 (14.6 to 36.9)	-36.2 (-56.5 to -11.9)	-28.3 (-49.6 to -1)	-39.8 (-61 to -12.2)
		Diet high in sodium	11.1 (1.8 to 46.4)	7.7 (1.8 to 29.8)	14.3 (1.4 to 60.8)	7.6 (1.2 to 30.9)	5.6 (1.3 to 23.9)	9.5 (0.9 to 38.4)	-32.2 (-59 to 20.3)	-27.1 (-66 to 49.4)	-33.7 (-61.6 to 33.3)
		High fasting plasma glucose	250.8 (183.6 to 329)	243.9 (177.3 to 324.9)	257 (182.5 to 347.7)	168.8 (120.1 to 232.1)	174.3 (122.2 to 243)	166.2 (115.9 to 230)	-32.7 (-53 to -5.8)	-28.5 (-51.6 to 1.3)	-35.3 (-57.7 to -4.1)
		High body-mass index	237.1 (135.8 to 357.2)	272.4 (160.5 to 401.8)	205.1 (102.3 to 332.8)	202.6 (116.5 to 294.6)	234.6 (139.8 to 339.2)	177.8 (97.6 to 275.2)	-14.5 (-40.6 to 29.8)	-13.9 (-41.2 to 28.3)	-13.3 (-45.2 to 45.3)
		High systolic blood pressure	425.4 (335.6 to 547.4)	420.4 (321.3 to 544.6)	430 (323.4 to 579.7)	305.8 (228.7 to 398.9)	328.7 (244.8 to 434.1)	293.8 (216.6 to 389.5)	-28.1 (-49.7 to 0.5)	-21.8 (-46.3 to 9.8)	-31.7 (-55 to 0.8)
		Kidney dysfunction	837.4 (689.9 to 1021.6)	833.1 (668.2 to 1028.9)	840.8 (674.3 to 1047)	535.4 (417.4 to 691)	571.8 (437.1 to 743.7)	513.6 (387.2 to 666.1)	-36.1 (-53.5 to -11.5)	-31.4 (-51.1 to -3.7)	-38.9 (-57.9 to -11.8)

Location	Measure	Risk factor	Attributed age-standardized rate (per 100,000)						% Change (1990 to 2019)		
			1990			2019					
			Both	Female	Male	Both	Female	Male	Both	Female	Male
	YLDs	Lead exposure	4.2 (2.3 to 6.8)	4.3 (2.1 to 7.3)	4.1 (2.4 to 6.5)	5 (2.7 to 8.3)	4.3 (2.1 to 7.5)	5.6 (3.2 to 9)	19.1 (2.8 to 37)	0.4 (-13.1 to 17)	36.9 (13.3 to 62.1)
		Diet high in sodium	1.4 (0.2 to 5.5)	1.3 (0.3 to 5.2)	1.5 (0.1 to 5.8)	1.9 (0.3 to 7.5)	1.5 (0.3 to 5.9)	2.3 (0.2 to 9.2)	39.4 (-13.9 to 117.6)	16 (-36.8 to 115.4)	58.9 (2 to 183.9)
		High fasting plasma glucose	22.2 (15.7 to 30.5)	24.7 (17.4 to 34)	19.9 (13.8 to 28.1)	32 (22.3 to 44.6)	31.5 (21.6 to 44.1)	32.3 (21.4 to 47)	44 (28.2 to 61.4)	27.4 (9.7 to 45.1)	62.1 (38.3 to 91.4)
		High body-mass index	32.5 (17.7 to 52.3)	45.1 (24.7 to 70.7)	20.9 (10.2 to 35.8)	51.9 (30.1 to 80.5)	61.2 (36.6 to 93.7)	43 (23 to 68.7)	59.8 (37.5 to 92.2)	35.7 (14.2 to 63.9)	105.6 (62.1 to 178.8)
		High systolic blood pressure	51.3 (35.6 to 70)	62.4 (42.2 to 86.1)	41.3 (28.4 to 57.9)	71.1 (49.8 to 97.8)	75.7 (52 to 104.9)	66.7 (45.8 to 94.2)	38.5 (20.7 to 58.7)	21.4 (2.1 to 47.1)	61.5 (36.5 to 90.1)
		Kidney dysfunction	117.7 (85.6 to 155.2)	146.5 (107.4 to 192.2)	90.9 (65.1 to 121.5)	149.5 (109 to 196.7)	165.4 (120.5 to 216.3)	132.5 (94.4 to 177.5)	27.1 (16.1 to 39.9)	12.9 (1.3 to 26.4)	45.8 (28.1 to 65.3)
Tunisia	Deaths	Non-optimal temperature	1.6 (0.5 to 2.4)	1.5 (0.4 to 2.4)	1.7 (0.5 to 2.6)	1.7 (0.8 to 2.8)	1.7 (0.8 to 2.7)	1.8 (0.9 to 3)	9 (-28.6 to 90.3)	9.4 (-28.9 to 97.4)	8 (-31.8 to 100.3)
		Lead exposure	1.1 (0.7 to 1.6)	0.9 (0.5 to 1.4)	1.3 (0.8 to 2)	1 (0.6 to 1.6)	0.8 (0.4 to 1.3)	1.3 (0.7 to 2.1)	-6.6 (-36.1 to 26.6)	-6.1 (-37.4 to 31.1)	-5.2 (-39.5 to 38.8)
		Diet high in sodium	0.4 (0.1 to 1.4)	0.2 (0.1 to 1)	0.5 (0.1 to 2)	0.3 (0.1 to 1.4)	0.2 (0.1 to 1)	0.5 (0 to 1.8)	-3.6 (-46.6 to 85.6)	-2 (-56.5 to 103.7)	-2 (-49.7 to 138.5)
		High fasting plasma glucose	7 (5.2 to 9.2)	6.7 (4.9 to 9.3)	7.3 (5.2 to 9.9)	6.9 (4.7 to 9.9)	6.7 (4.3 to 9.8)	7.3 (4.7 to 10.9)	-0.1 (-31.8 to 37.7)	0.7 (-33.1 to 39.6)	-1 (-36.1 to 43.7)
		High body-mass index	6.8 (3.7 to 10.9)	7.8 (4.5 to 12.2)	5.8 (2.7 to 10.1)	8.8 (4.6 to 13.8)	9.4 (5 to 14.7)	8 (3.7 to 13.6)	29.7 (-12.5 to 92.7)	21.6 (-21 to 78)	37.6 (-15.3 to 133.3)
		High systolic blood pressure	15 (12.2 to 18.6)	14.5 (11.3 to 19.9)	15.7 (12 to 19.6)	15.1 (10.9 to 20.2)	14.7 (10.1 to 20)	15.7 (10.6 to 22.3)	0.8 (-29.7 to 38.1)	0.8 (-31.5 to 40.6)	0.1 (-35.1 to 43.9)

Location	Measure	Risk factor	Attributed age-standardized rate (per 100,000)						% Change (1990 to 2019)		
			1990			2019					
			Both	Female	Male	Both	Female	Male	Both	Female	Male
		Kidney dysfunction	24.3 (20.6 to 29.4)	23.5 (19.6 to 31.7)	25.4 (20.5 to 31.2)	23.6 (16.9 to 31.1)	22.9 (15.8 to 30.8)	24.5 (16.8 to 33.9)	-2.7 (-31.9 to 31.3)	-2.2 (-33.1 to 34.8)	-3.6 (-36.6 to 37.5)
	DALYs	Non-optimal temperature	31.4 (9.3 to 47.4)	31.7 (9 to 48.8)	31.3 (9.2 to 48.9)	31.1 (14.8 to 50.5)	30.3 (13.9 to 48.9)	31.9 (15.2 to 54)	-1.2 (-34.2 to 80)	-4.4 (-38.2 to 67.1)	1.8 (-34.4 to 91.2)
		Lead exposure	21.8 (13.3 to 33)	17.8 (9.3 to 28.3)	25.7 (16 to 38.6)	18.7 (10.2 to 29.6)	14.7 (6.9 to 24.4)	23.1 (12.8 to 37.2)	-14.2 (-39.3 to 12.9)	-17.4 (-41.5 to 10.6)	-10 (-38.6 to 25.1)
		Diet high in sodium	7.8 (1.2 to 31.5)	5.6 (1.3 to 22.6)	9.9 (1 to 40.4)	8.1 (1.3 to 31.5)	5.7 (1.3 to 23)	10.7 (1.1 to 41.1)	3.4 (-38.9 to 84.7)	1.1 (-52.7 to 106.8)	7.5 (-38.8 to 120.1)
		High fasting plasma glucose	154.6 (119.4 to 196.1)	153 (116.4 to 207.3)	156.6 (116.2 to 204.4)	161.6 (116.6 to 216.3)	156.1 (109.3 to 211.7)	167.8 (120.6 to 235.2)	4.5 (-23.5 to 39.5)	2 (-26.7 to 36.9)	7.2 (-25.4 to 49.4)
		High body-mass index	165.9 (99.5 to 253.4)	194.8 (120.9 to 285.5)	138.9 (72.1 to 226)	218.6 (130.3 to 312.6)	234 (145.4 to 333.6)	202 (113.4 to 310.3)	31.7 (-5.7 to 82.7)	20.1 (-14.8 to 63.8)	45.4 (-2.6 to 127.9)
		High systolic blood pressure	297.4 (243.5 to 360)	301.9 (236.3 to 392.8)	294.8 (228.7 to 369.5)	315.6 (238.9 to 406.9)	309.8 (228 to 410.2)	322.8 (236 to 442.6)	6.1 (-21.2 to 40.5)	2.6 (-26.8 to 38.1)	9.5 (-23.3 to 52.3)
		Kidney dysfunction	577.2 (504 to 666.4)	594.2 (511.7 to 737.4)	562.5 (476 to 665.4)	568.6 (441 to 718.9)	561.6 (430.5 to 713)	577.2 (440.4 to 756.7)	-1.5 (-25 to 25.5)	-5.5 (-28 to 21.5)	2.6 (-24.8 to 37.5)
	YLLs	Non-optimal temperature	31.4 (9.3 to 47.4)	31.7 (9 to 48.8)	31.3 (9.2 to 48.9)	31.1 (14.8 to 50.5)	30.3 (13.9 to 48.9)	31.9 (15.2 to 54)	-1.2 (-34.2 to 80)	-4.4 (-38.2 to 67.1)	1.8 (-34.4 to 91.2)
		Lead exposure	18.7 (11.3 to 28.4)	15 (7.8 to 24.2)	22.3 (13.9 to 34.1)	14.9 (7.7 to 24.4)	11.8 (5.4 to 19.9)	18.3 (9.6 to 30.7)	-20.3 (-47.2 to 10.5)	-21.1 (-48.2 to 11.1)	-17.8 (-49 to 22.6)
		Diet high in sodium	6.6 (1 to 26.9)	4.7 (1 to 18.5)	8.6 (0.9 to 35.1)	6.2 (0.9 to 24.7)	4.3 (0.9 to 17.7)	8.2 (0.8 to 32.2)	-6.6 (-47.6 to 77.4)	-6.5 (-58.6 to 100.9)	-4.1 (-47.9 to 106.3)
		High fasting plasma glucose	134.5 (100.1 to 175.1)	132.3 (97.1 to 185)	137.1 (98.7 to 185.2)	129.3 (86 to 182.9)	126.3 (80.5 to 181)	133 (86.6 to 198.7)	-3.9 (-35.2 to 35.9)	-4.6 (-37.5 to 36.1)	-3 (-37.5 to 42.9)

Location	Measure	Risk factor	Attributed age-standardized rate (per 100,000)						% Change (1990 to 2019)		
			1990			2019					
			Both	Female	Male	Both	Female	Male	Both	Female	Male
		High body-mass index	140.2 (82.7 to 215.3)	162.6 (98.9 to 244.3)	119.5 (61 to 196.3)	169 (94.9 to 255.1)	181.2 (107.3 to 268.1)	155.9 (82.6 to 253.1)	20.5 (-19.6 to 76.9)	11.5 (-27.5 to 60.9)	30.5 (-21.1 to 121.2)
		High systolic blood pressure	255.4 (205.8 to 317.2)	254.9 (197.6 to 347.7)	257.7 (194.6 to 329.3)	248 (176.6 to 336.4)	243.4 (167.7 to 334.2)	254 (171.3 to 363.2)	-2.9 (-33.8 to 36.8)	-4.5 (-36.2 to 37.1)	-1.5 (-37 to 45.8)
		Kidney dysfunction	483 (414.9 to 566.7)	487.1 (412.2 to 624.4)	481.2 (395.5 to 586.2)	426.1 (304.4 to 571.2)	416.5 (290.7 to 559.4)	437.3 (305.5 to 614.6)	-11.8 (-38.7 to 20.6)	-14.5 (-41.9 to 17.4)	-9.1 (-38.6 to 30.5)
	YLDs	Lead exposure	3.1 (1.6 to 5.1)	2.8 (1.3 to 4.9)	3.4 (1.9 to 5.2)	3.8 (1.9 to 6.5)	2.9 (1.3 to 5.4)	4.8 (2.5 to 8)	22 (3.1 to 39.5)	2.2 (-16.7 to 20.3)	41.7 (16.7 to 65.8)
		Diet high in sodium	1.2 (0.2 to 4.6)	1 (0.2 to 3.8)	1.4 (0.1 to 5.6)	1.9 (0.3 to 7.1)	1.3 (0.3 to 5.1)	2.5 (0.2 to 9.7)	60.7 (3.2 to 154.5)	38.1 (-23.6 to 154.8)	80.4 (17.9 to 210.2)
		High fasting plasma glucose	20.1 (14.1 to 27.8)	20.7 (14.3 to 28.3)	19.4 (13.2 to 27.8)	32.2 (22.1 to 44.9)	29.8 (20.6 to 41)	34.8 (23.2 to 50.1)	60.5 (43.5 to 81.3)	44 (26 to 65.6)	79.1 (54.7 to 111.4)
		High body-mass index	25.7 (13.8 to 40.9)	32.2 (18.2 to 50.5)	19.4 (9.5 to 32.1)	49.6 (28.1 to 76.8)	52.8 (31.1 to 80.6)	46.1 (24.3 to 74.6)	92.8 (64.7 to 134.7)	63.7 (37.2 to 99.8)	137.5 (91.5 to 219.7)
		High systolic blood pressure	42 (29.3 to 56.3)	47 (32.2 to 64)	37.1 (25.4 to 51)	67.5 (47.4 to 91.4)	66.4 (45.9 to 90.2)	68.8 (47.1 to 96.1)	60.7 (41.5 to 82.5)	41.3 (17.5 to 70.6)	85.7 (57.5 to 115.5)
		Kidney dysfunction	94.2 (68.2 to 124.4)	107.1 (77.4 to 141.6)	81.2 (58.2 to 108.3)	142.4 (103.7 to 189.2)	145 (106.5 to 189.9)	139.9 (99 to 190.6)	51.3 (38.7 to 65.2)	35.4 (21.5 to 51.1)	72.3 (54.5 to 92.9)
Turkey	Deaths	Non-optimal temperature	3.5 (1.9 to 5.7)	3.1 (1.6 to 5)	4.1 (2.1 to 7)	2.2 (1.2 to 3.5)	2.1 (1.1 to 3.3)	2.3 (1.2 to 3.7)	-36.9 (-59.4 to -13.2)	-30.1 (-56.4 to -2.9)	-43.3 (-65.4 to -15.4)
		Lead exposure	0.8 (0.3 to 1.7)	0.6 (0.2 to 1.3)	1.1 (0.4 to 2.2)	0.5 (0.2 to 0.9)	0.4 (0.1 to 0.8)	0.6 (0.2 to 1.1)	-39.8 (-63.5 to -16.6)	-33.4 (-64.3 to -7.7)	-44.1 (-66.6 to -15)
		Diet high in sodium	0.4 (0.1 to 1.7)	0.2 (0.1 to 0.9)	0.5 (0.1 to 2.7)	0.2 (0.1 to 1)	0.2 (0.1 to 0.7)	0.3 (0 to 1.6)	-35.1 (-66.4 to 30.5)	-25.8 (-66.4 to 51.3)	-40.1 (-74.3 to 50.3)

Location	Measure	Risk factor	Attributed age-standardized rate (per 100,000)						% Change (1990 to 2019)		
			1990			2019					
			Both	Female	Male	Both	Female	Male	Both	Female	Male
		High fasting plasma glucose	12.5 (9.3 to 18.4)	10.6 (7.8 to 16.8)	14.8 (10.5 to 22.9)	8.2 (6.2 to 10.5)	7.7 (5.6 to 10.1)	8.7 (6.5 to 11.4)	-34.8 (-57.8 to -11.6)	-27.7 (-55.6 to -1.2)	-41.2 (-64.1 to -12.9)
		High body-mass index	11.6 (6.5 to 19.1)	11.9 (6.9 to 19.5)	11.3 (5.6 to 20.4)	9.7 (5.4 to 14.7)	10.1 (5.7 to 15.1)	9.2 (4.9 to 14.6)	-16.4 (-46.7 to 16.7)	-15.6 (-49.6 to 17.4)	-17.9 (-50.5 to 30.3)
		High systolic blood pressure	22.5 (17.3 to 33.1)	19.5 (15 to 30.1)	26.1 (18.9 to 40.6)	15.5 (12.1 to 19.3)	15 (11.5 to 18.8)	16.2 (12.5 to 20.2)	-30.9 (-55.7 to -5.5)	-23.3 (-54.1 to 6.8)	-38 (-62.3 to -6.4)
		Kidney dysfunction	35.2 (28.3 to 50.3)	30.6 (24.3 to 46.5)	40.9 (30.7 to 60.6)	23.5 (19 to 28.7)	22.6 (18 to 27.9)	24.5 (19.5 to 30.1)	-33.3 (-56.2 to -9.6)	-26.2 (-54.3 to -0.1)	-40 (-62.7 to -11.3)
	DALYs	Non-optimal temperature	76.3 (40.9 to 116)	67.7 (35.6 to 105.6)	86.2 (46.9 to 135.1)	40 (20.7 to 62.1)	37.6 (19.1 to 58.6)	42.5 (22.2 to 66.2)	-47.6 (-63.4 to -29.5)	-44.5 (-62.5 to -24.1)	-50.7 (-67.3 to -28.4)
		Lead exposure	16.4 (5.8 to 31.2)	12.6 (3.3 to 25)	20.8 (7.9 to 40.3)	9 (2.5 to 17.3)	7.3 (1.5 to 14.9)	11 (3.6 to 20.2)	-45.1 (-66.8 to -26.7)	-41.7 (-67.7 to -21.9)	-47.1 (-68.7 to -23.8)
		Diet high in sodium	8.3 (1.9 to 35.6)	5.4 (1.8 to 20.1)	11.6 (1.6 to 53.6)	5.7 (1.4 to 23.7)	4 (1.4 to 14.9)	7.6 (1.1 to 35.3)	-31.3 (-60.9 to 24.9)	-25.6 (-62.4 to 50.5)	-34.2 (-69.2 to 48)
		High fasting plasma glucose	276.9 (214.3 to 378)	238.5 (181.2 to 351.3)	320.1 (235.9 to 456.1)	182.7 (142.1 to 229.8)	167.6 (130.5 to 212.4)	199.2 (152.5 to 253)	-34 (-53.7 to -13.8)	-29.7 (-53.4 to -6.8)	-37.8 (-58.6 to -12.8)
		High body-mass index	274.5 (163.9 to 414.1)	285.1 (176.4 to 434.5)	262.4 (140 to 428.7)	237.3 (150.2 to 334.4)	241.3 (155.8 to 338.2)	231.3 (138.2 to 337.2)	-13.5 (-39.8 to 15)	-15.4 (-42.7 to 11.8)	-11.9 (-42.6 to 32.9)
		High systolic blood pressure	450.6 (359.2 to 616.7)	399.3 (314.6 to 567.1)	509.3 (382.4 to 722.1)	321.2 (258.2 to 393.2)	303.9 (242.3 to 374.3)	339.6 (267.5 to 416.6)	-28.7 (-50 to -6.1)	-23.9 (-49.9 to 1)	-33.3 (-56.4 to -7.1)
		Kidney dysfunction	853.9 (726 to 1083.2)	778.1 (649.4 to 1036.9)	941.5 (750.6 to 1240.3)	562 (473.6 to 667.6)	540.2 (453.4 to 645.1)	586 (486.6 to 698.6)	-34.2 (-51.3 to -17.5)	-30.6 (-50.3 to -12.2)	-37.8 (-55.9 to -16.3)
	YLLs	Non-optimal temperature	76.3 (40.9 to 116)	67.7 (35.6 to 105.6)	86.2 (46.9 to 135.1)	40 (20.7 to 62.1)	37.6 (19.1 to 58.6)	42.5 (22.2 to 66.2)	-47.6 (-63.4 to -29.5)	-44.5 (-62.5 to -24.1)	-50.7 (-67.3 to -28.4)

Location	Measure	Risk factor	Attributed age-standardized rate (per 100,000)						% Change (1990 to 2019)		
			1990			2019					
			Both	Female	Male	Both	Female	Male	Both	Female	Male
		Lead exposure	14.8 (5.2 to 28.6)	11.1 (3 to 22.4)	19.1 (7.2 to 37.9)	7.2 (2 to 14)	5.8 (1.2 to 12)	8.8 (2.9 to 16.3)	-51.7 (-72.2 to -32.8)	-47.5 (-72.4 to -26.6)	-54.2 (-72.9 to -30.5)
		Diet high in sodium	7.5 (1.7 to 32.5)	4.7 (1.6 to 17.9)	10.6 (1.4 to 49.8)	4.4 (1 to 18.6)	3 (1 to 11.3)	5.9 (0.8 to 28.2)	-41.1 (-68.1 to 10.5)	-35.2 (-68.2 to 35.6)	-44 (-75.2 to 30.6)
		High fasting plasma glucose	256.7 (194.7 to 356.2)	218.5 (162.8 to 330.2)	299.5 (216 to 433.4)	149.7 (112.7 to 194.4)	137.4 (101.4 to 179.5)	162.7 (118.9 to 212.5)	-41.7 (-60.9 to -21.1)	-37.1 (-60.1 to -13.1)	-45.7 (-65.4 to -20.6)
		High body-mass index	245.4 (145.6 to 377)	249.6 (153.1 to 392.1)	240.3 (127.2 to 402.9)	182.9 (111.2 to 264.7)	184 (115.8 to 263)	180 (102.2 to 268.8)	-25.5 (-50.7 to 2.5)	-26.3 (-53.1 to 2.6)	-25.1 (-53.7 to 16.5)
		High systolic blood pressure	405.9 (316.2 to 565)	351.5 (271.6 to 522.7)	468.1 (342.7 to 679.7)	251.9 (195.2 to 314.8)	236.7 (182.4 to 300)	267.9 (203.4 to 339)	-37.9 (-58.6 to -14.3)	-32.7 (-58 to -6.3)	-42.8 (-63.9 to -15.8)
		Kidney dysfunction	758.7 (631.1 to 987.2)	672.9 (551.1 to 930.6)	856.7 (672.4 to 1151.1)	420.3 (341.3 to 513.6)	394.9 (316.4 to 488.3)	446.9 (357 to 553.4)	-44.6 (-60.6 to -27)	-41.3 (-60.2 to -21.8)	-47.8 (-65 to -26.7)
	YLDs	Lead exposure	1.6 (0.5 to 3.1)	1.5 (0.4 to 3)	1.7 (0.6 to 3.1)	1.9 (0.5 to 3.9)	1.5 (0.3 to 3.5)	2.3 (0.7 to 4.6)	16.6 (-14.6 to 35.3)	0.7 (-36.4 to 20.7)	33.9 (-1.5 to 60.2)
		Diet high in sodium	0.8 (0.2 to 3.2)	0.7 (0.2 to 2.5)	1 (0.1 to 4.3)	1.3 (0.3 to 5.4)	1 (0.3 to 3.5)	1.7 (0.2 to 7.5)	57.3 (-5.4 to 151.1)	39.3 (-20.8 to 184.3)	72.2 (-7.9 to 225.7)
		High fasting plasma glucose	20.3 (14.6 to 27.2)	20 (14.2 to 27)	20.6 (14.5 to 28.2)	33.1 (22.8 to 46.4)	30.2 (20.9 to 41.9)	36.5 (24.2 to 51.3)	63.2 (44.6 to 84.5)	50.8 (29.7 to 76.5)	77 (50.7 to 106.8)
		High body-mass index	29.1 (16.2 to 45.9)	35.5 (20.4 to 55.2)	22.1 (11.5 to 35.5)	54.5 (32.2 to 82.4)	57.3 (34.9 to 84.9)	51.3 (28.5 to 82)	87 (59 to 121.7)	61.4 (36.9 to 93.1)	131.8 (87.6 to 206.5)
		High systolic blood pressure	44.7 (31.7 to 59.8)	47.8 (33.7 to 64.8)	41.2 (28.9 to 55.7)	69.3 (47.9 to 94.4)	67.2 (47.1 to 92.9)	71.7 (47.5 to 100.6)	55 (35.5 to 75.3)	40.6 (19.9 to 65.4)	74.1 (49 to 99.7)
		Kidney dysfunction	95.2 (69.9 to 125.6)	105.2 (77.7 to 139.6)	84.8 (61.6 to 112)	141.8 (102.5 to 187.2)	145.3 (106.4 to 192)	139.1 (97 to 187.5)	48.9 (35.5 to 64.1)	38.1 (23.4 to 56.8)	64.1 (44 to 84.8)

Location	Measure	Risk factor	Attributed age-standardized rate (per 100,000)						% Change (1990 to 2019)		
			1990			2019					
			Both	Female	Male	Both	Female	Male	Both	Female	Male
United Arab Emirates	Deaths	Non-optimal temperature	5.5 (-14.4 to 18.6)	5.6 (-15.5 to 19.6)	5.4 (-13.7 to 18.5)	4.9 (-6.6 to 14.9)	5.3 (-7.8 to 16.3)	4.7 (-6.8 to 15.4)	-10.8 (-205.3 to 64.6)	-4.8 (-218.5 to 71.1)	-13.1 (-202.1 to 53.1)
		Lead exposure	1.5 (0.6 to 2.7)	1.4 (0.4 to 2.8)	1.6 (0.6 to 2.8)	0.9 (0.2 to 1.9)	0.9 (0.2 to 1.8)	0.9 (0.2 to 2.1)	-40.4 (-63.7 to -14.4)	-39 (-70.9 to -10.6)	-41.9 (-65.7 to -12)
		Diet high in sodium	1 (0.1 to 4.4)	0.7 (0.1 to 3.1)	1.3 (0.1 to 5.8)	0.8 (0.1 to 3.2)	0.5 (0.1 to 2.2)	0.9 (0.1 to 3.9)	-21.7 (-57.7 to 53.8)	-22.8 (-68.3 to 74.5)	-27.4 (-63.7 to 72.8)
		High fasting plasma glucose	22.4 (13.9 to 29.5)	22.6 (13 to 32.5)	22.3 (13.2 to 31)	17.6 (10.4 to 28.3)	18.6 (9.3 to 26.4)	17.1 (10.1 to 31.1)	-21.4 (-47.2 to 12.6)	-17.6 (-52.3 to 19.9)	-23.2 (-48.8 to 21.2)
		High body-mass index	23.8 (13 to 36.4)	27.4 (13.9 to 42.9)	21.4 (11.2 to 34.8)	23.5 (12.4 to 38.6)	26.7 (12.5 to 40.9)	22.1 (10.8 to 41.2)	-1.6 (-35.3 to 46.1)	-2.4 (-44.2 to 50.4)	3.6 (-35.1 to 70.5)
		High systolic blood pressure	42.8 (25.1 to 53.7)	43.8 (24.1 to 60.7)	42.3 (24.1 to 56.2)	31.4 (18.4 to 49.3)	33.9 (16.2 to 46.4)	30.3 (17.8 to 53.8)	-26.7 (-49.7 to 3.7)	-22.7 (-55.2 to 14.6)	-28.3 (-51.7 to 11.3)
		Kidney dysfunction	61.2 (37.5 to 75.7)	63.3 (38.3 to 86.7)	60 (35.3 to 78.1)	48.1 (29.1 to 73.6)	52.1 (25.9 to 70.1)	46.4 (28.6 to 80.2)	-21.5 (-46.6 to 11.6)	-17.7 (-51.6 to 19.6)	-22.6 (-47.6 to 21.2)
	DALYs	Non-optimal temperature	108.1 (-282.2 to 365.7)	109.6 (-301 to 377.6)	107.5 (-275.4 to 371.4)	96.5 (-129.8 to 293.7)	103.1 (-147.9 to 309.1)	93.8 (-132.8 to 303.5)	-10.7 (-202.6 to 63.3)	-5.9 (-213.9 to 68.3)	-12.7 (-203.8 to 54.6)
		Lead exposure	29.3 (10.4 to 52)	27.4 (7.5 to 52.9)	30.5 (11.6 to 54.9)	15.6 (3.2 to 33.4)	14.7 (2.1 to 32.2)	16 (3.5 to 36.4)	-46.9 (-72.8 to -24.6)	-46.5 (-78.1 to -21.5)	-47.7 (-72.5 to -20.3)
		Diet high in sodium	22.4 (2.8 to 96.1)	15.4 (3.1 to 64.6)	26.9 (2.3 to 118.7)	18.9 (2.3 to 75)	12.5 (2.6 to 49.2)	21.4 (2 to 88.6)	-15.6 (-50.7 to 50.4)	-19 (-67.9 to 93.5)	-20.2 (-55 to 68.8)
		High fasting plasma glucose	478.9 (324.6 to 624.2)	487.2 (300.5 to 691)	475.2 (306.2 to 653.4)	401 (266.2 to 625.3)	416.2 (237.2 to 576.9)	394.6 (253 to 680.6)	-16.3 (-42.3 to 20.1)	-14.6 (-48.7 to 23.6)	-17 (-43.2 to 27.5)
		High body-mass index	549.1 (326.6 to 784.1)	636.9 (364.8 to 952)	494 (268.9 to 765.2)	565.2 (334.2 to 862.8)	633 (351.4 to 896.3)	538.9 (312.7 to 902.5)	2.9 (-29 to 47)	-0.6 (-38.9 to 43)	9.1 (-26.7 to 73.8)

Location	Measure	Risk factor	Attributed age-standardized rate (per 100,000)						% Change (1990 to 2019)		
			1990			2019					
			Both	Female	Male	Both	Female	Male	Both	Female	Male
		High systolic blood pressure	848.8 (545.6 to 1050.2)	866.8 (527.4 to 1164.9)	836.9 (531.1 to 1088.7)	656.9 (429.6 to 990.3)	687.5 (410 to 927.5)	642.7 (419.9 to 1071)	-22.6 (-44.8 to 7.8)	-20.7 (-51 to 13)	-23.2 (-45.8 to 15.9)
		Kidney dysfunction	1343.2 (954.7 to 1636.8)	1400 (960 to 1849.7)	1312.1 (880.1 to 1667.6)	1136.9 (787.6 to 1645.1)	1207.9 (757 to 1568.7)	1108.1 (756.9 to 1769.3)	-15.4 (-40.1 to 17.9)	-13.7 (-46.2 to 20.1)	-15.5 (-40.1 to 26.9)
	YLLs	Non-optimal temperature	108.1 (-282.2 to 365.7)	109.6 (-301 to 377.6)	107.5 (-275.4 to 371.4)	96.5 (-129.8 to 293.7)	103.1 (-147.9 to 309.1)	93.8 (-132.8 to 303.5)	-10.7 (-202.6 to 63.3)	-5.9 (-213.9 to 68.3)	-12.7 (-203.8 to 54.6)
		Lead exposure	26.9 (9.6 to 47.8)	24.8 (6.7 to 49)	28.2 (10.6 to 51.8)	13.4 (2.7 to 30.1)	12.7 (1.7 to 28.2)	13.7 (2.9 to 32.5)	-50.2 (-75.3 to -26.8)	-48.8 (-79.9 to -21.2)	-51.5 (-75.7 to -23.3)
		Diet high in sodium	20.5 (2.6 to 90.2)	13.8 (2.7 to 59.6)	24.8 (2 to 109.5)	16.1 (1.9 to 65.3)	10.6 (2 to 42.5)	18.2 (1.6 to 77.2)	-21.7 (-56.1 to 45.5)	-23.3 (-70.9 to 89.4)	-26.6 (-58.8 to 55.1)
		High fasting plasma glucose	450.1 (293.2 to 592.1)	456 (271.1 to 661.4)	447.8 (281.6 to 625.4)	357.4 (221.3 to 582.5)	374.8 (197.5 to 538.7)	349.9 (210.1 to 637)	-20.6 (-47.9 to 17.9)	-17.8 (-54.1 to 22.8)	-21.9 (-49.8 to 24.4)
		High body-mass index	500.3 (287.7 to 728)	571.6 (311 to 873.4)	454.8 (243.6 to 710.4)	480.9 (271.6 to 773.7)	539.1 (270.3 to 791)	458.1 (244.1 to 822.5)	-3.9 (-38.3 to 43.8)	-5.7 (-47.1 to 42.2)	0.7 (-36.9 to 68.7)
		High systolic blood pressure	778.2 (478.9 to 979.5)	785 (446.5 to 1084.7)	773.5 (462.3 to 1028.8)	564.6 (343.2 to 888)	593.3 (304.2 to 831.8)	551.1 (337.7 to 970.6)	-27.5 (-51.3 to 5.3)	-24.4 (-56.8 to 11.9)	-28.8 (-53.6 to 12.5)
		Kidney dysfunction	1212.5 (823.9 to 1503.4)	1238.2 (801.1 to 1686.9)	1199.1 (756.6 to 1564.4)	952.8 (612.6 to 1458.9)	1006.8 (573.6 to 1378.5)	931 (594.5 to 1595.4)	-21.4 (-47.3 to 14.1)	-18.7 (-53.5 to 19.6)	-22.4 (-48.4 to 23.6)
	YLDs	Lead exposure	2.4 (0.8 to 4.8)	2.6 (0.7 to 5.3)	2.3 (0.8 to 4.4)	2.2 (0.5 to 4.8)	2 (0.3 to 4.6)	2.3 (0.5 to 4.9)	-10.2 (-47.1 to 9.3)	-25.4 (-64.7 to -7.1)	-0.1 (-40.7 to 26.3)
		Diet high in sodium	1.9 (0.3 to 7.6)	1.6 (0.3 to 6.4)	2.1 (0.2 to 8.5)	2.9 (0.4 to 11)	1.9 (0.4 to 7.3)	3.3 (0.3 to 12.6)	49.6 (-10.2 to 130.2)	18.6 (-42.7 to 147.9)	54.8 (-0.6 to 192.1)
		High fasting plasma glucose	28.8 (19.9 to 40.4)	31.2 (21.9 to 43.3)	27.4 (18.3 to 39.5)	43.6 (29.2 to 61.7)	41.4 (28.2 to 58)	44.8 (29 to 64.4)	51.6 (35.7 to 69.3)	32.7 (16 to 52)	63.4 (41.4 to 89.8)

Location	Measure	Risk factor	Attributed age-standardized rate (per 100,000)						% Change (1990 to 2019)		
			1990			2019					
			Both	Female	Male	Both	Female	Male	Both	Female	Male
		High body-mass index	48.8 (28.6 to 74.3)	65.3 (38.8 to 98.4)	39.2 (21.7 to 63)	84.3 (52.6 to 126.2)	93.9 (58.9 to 135.9)	80.7 (49.3 to 123.8)	72.8 (49.1 to 104.7)	43.8 (22.6 to 69.9)	105.9 (69.9 to 156.2)
		High systolic blood pressure	70.5 (49.7 to 96.4)	81.9 (56.8 to 111.1)	63.4 (43.7 to 89)	92.3 (64.3 to 127.1)	94.2 (63.9 to 129.7)	91.5 (61.9 to 127.8)	30.9 (17.4 to 46.1)	15.1 (-0.1 to 33.2)	44.5 (26.6 to 67.3)
		Kidney dysfunction	130.7 (94.9 to 173.2)	161.8 (118.4 to 210.7)	113 (81.4 to 154.3)	184.2 (133 to 246.9)	201.1 (148.4 to 264.9)	177.1 (124.9 to 242.7)	40.9 (28.8 to 54.1)	24.3 (12.6 to 37.3)	56.7 (40.6 to 75.2)
Yemen	Deaths	Non-optimal temperature	1.3 (-2.4 to 3.4)	1.1 (-2.3 to 3.4)	1.5 (-3.1 to 3.9)	1.3 (-0.5 to 2.8)	1.1 (-0.5 to 2.6)	1.4 (-0.6 to 3.1)	-1.3 (-230.7 to 183.2)	0.3 (-226.7 to 204.6)	-8.5 (-226.4 to 163.7)
		Lead exposure	2.2 (1.4 to 3.2)	1.6 (1 to 2.6)	3.1 (2 to 4.9)	2.4 (1.6 to 3.4)	1.7 (1.1 to 2.5)	3.1 (2.1 to 4.6)	7.8 (-18.5 to 41.2)	5.4 (-27.2 to 46.3)	-0.4 (-25.9 to 35.8)
		Diet high in sodium	0.4 (0.1 to 1.5)	0.2 (0.1 to 1)	0.6 (0.1 to 2.3)	0.3 (0.1 to 1.4)	0.2 (0.1 to 0.9)	0.5 (0.1 to 2)	-5.2 (-45.8 to 76.6)	-5.8 (-55.2 to 101.9)	-13.2 (-52.9 to 97.9)
		High fasting plasma glucose	8 (5.4 to 12)	7 (4.5 to 11.7)	9.7 (6.5 to 14.6)	7.6 (5.4 to 10.4)	6.8 (4.7 to 9.8)	8.5 (5.7 to 12.2)	-5.4 (-28.4 to 25.9)	-3.8 (-32.9 to 32.3)	-11.9 (-34.9 to 20.7)
		High body-mass index	3.2 (1.1 to 6.6)	3.7 (1.5 to 7.3)	2.7 (0.7 to 6.7)	5.1 (2.4 to 8.7)	5.5 (2.6 to 9.2)	4.6 (2 to 8.8)	56.3 (4.7 to 176.2)	46.4 (-5.7 to 158.5)	70.3 (5 to 287.6)
		High systolic blood pressure	14.8 (10.8 to 21)	13.3 (9.2 to 21.2)	17.9 (12.7 to 26.4)	14.5 (11 to 19.8)	13.1 (9.7 to 18.7)	16.1 (11.6 to 22.6)	-2.3 (-26.5 to 27.5)	-1 (-32.1 to 39.2)	-10 (-32.6 to 22.7)
		Kidney dysfunction	24.8 (18.6 to 34.2)	22.1 (15.8 to 34.5)	29.6 (21.9 to 42.1)	23.2 (18 to 30.5)	21 (15.9 to 28.9)	25.7 (19 to 35.3)	-6.5 (-28.5 to 22.8)	-5.3 (-32.5 to 31.5)	-13.2 (-34.2 to 17.4)
	DALYs	Non-optimal temperature	28.4 (-54 to 74.6)	26.2 (-51.8 to 75.9)	31.7 (-61.9 to 79.1)	25.8 (-11.1 to 56.7)	24.1 (-10.3 to 52.9)	27.7 (-12.3 to 62.4)	-9 (-215.2 to 157)	-7.8 (-223.8 to 167.2)	-12.7 (-210.1 to 144.1)
		Lead exposure	50.3 (33.1 to 70.9)	37.9 (23 to 61.1)	66 (43.9 to 96)	50.1 (34.7 to 70.4)	37.6 (24.6 to 54.6)	63.3 (43.8 to 89.7)	-0.4 (-23.4 to 29)	-0.8 (-27.6 to 30.7)	-4.1 (-27.8 to 28.9)

Location	Measure	Risk factor	Attributed age-standardized rate (per 100,000)						% Change (1990 to 2019)		
			1990			2019					
			Both	Female	Male	Both	Female	Male	Both	Female	Male
		Diet high in sodium	8.8 (1.3 to 34.6)	5.9 (1.3 to 24)	12.3 (1.2 to 48.9)	8.5 (1.4 to 33.3)	5.9 (1.4 to 23.6)	11.3 (1.2 to 45.8)	-2.9 (-42.4 to 72.7)	-0.4 (-53 to 108.6)	-8.1 (-45 to 81.6)
		High fasting plasma glucose	193.9 (133.6 to 282.2)	176.8 (118.2 to 279.2)	217.3 (149.1 to 310)	184.9 (133.5 to 253.5)	172.5 (123.7 to 242.4)	198.2 (140.5 to 273.7)	-4.7 (-27.4 to 27.6)	-2.4 (-31.2 to 33.4)	-8.8 (-33 to 25.2)
		High body-mass index	87.6 (31.7 to 168.8)	105.9 (43.6 to 202.8)	69.1 (19.8 to 152.9)	143.7 (71.8 to 232.8)	163.7 (83.3 to 260.5)	123.4 (55.8 to 214.3)	64.1 (14.5 to 176.2)	54.5 (3.9 to 158.4)	78.6 (14.9 to 291.3)
		High systolic blood pressure	323 (235 to 433.3)	304.7 (213.4 to 463.1)	354 (256.2 to 494)	323.2 (248.5 to 417)	313.7 (237 to 423.6)	334.1 (250.6 to 441.3)	0 (-23.8 to 30.3)	3 (-27.2 to 39.3)	-5.6 (-29.7 to 28.6)
		Kidney dysfunction	641.2 (494.5 to 835.6)	618.9 (457.3 to 906.1)	682.7 (515.5 to 913.6)	611.6 (486.8 to 769.3)	604.3 (477.5 to 798.4)	621.2 (480.8 to 794.2)	-4.6 (-26.1 to 22.9)	-2.4 (-28.8 to 28.3)	-9 (-30.5 to 21.9)
	YLLs	Non-optimal temperature	28.4 (-54 to 74.6)	26.2 (-51.8 to 75.9)	31.7 (-61.9 to 79.1)	25.8 (-11.1 to 56.7)	24.1 (-10.3 to 52.9)	27.7 (-12.3 to 62.4)	-9 (-215.2 to 157)	-7.8 (-223.8 to 167.2)	-12.7 (-210.1 to 144.1)
		Lead exposure	44.4 (28.7 to 64.1)	32 (19 to 53.4)	60.2 (39.2 to 91.4)	41 (27.5 to 59.5)	29.4 (18.4 to 43.8)	53.3 (35.3 to 77.7)	-7.8 (-32.2 to 25.1)	-8.2 (-35.8 to 30.1)	-11.5 (-37 to 23)
		Diet high in sodium	7.8 (1.1 to 31.1)	5 (1 to 20.6)	11.2 (1 to 44.8)	6.9 (1 to 28.3)	4.5 (1 to 18.9)	9.4 (0.9 to 38.9)	-10.9 (-48.9 to 65.5)	-9.1 (-59.2 to 103)	-15.5 (-51 to 71.9)
		High fasting plasma glucose	177 (116 to 265.2)	158.7 (100.6 to 259.8)	201.7 (133.5 to 293)	158 (108.6 to 222.8)	145.4 (98.2 to 212.6)	171.4 (115.9 to 245.8)	-10.7 (-34.5 to 23.1)	-8.4 (-36.5 to 31.8)	-15 (-39.7 to 20.9)
		High body-mass index	76.5 (26.5 to 151.4)	90.2 (36.4 to 178.3)	63 (17.5 to 140.1)	115.7 (57.5 to 191.2)	127.3 (63.5 to 209)	103.8 (45.4 to 184.4)	51.2 (1.8 to 167.4)	41.2 (-8.4 to 148.2)	64.9 (2 to 278.8)
		High systolic blood pressure	286.5 (203.9 to 392.9)	261.7 (176.9 to 418.3)	325.6 (231.2 to 467.6)	264.9 (198.3 to 360.1)	247.9 (177.9 to 355.5)	283.7 (204 to 393.5)	-7.5 (-31.6 to 25.6)	-5.3 (-35.6 to 35.5)	-12.9 (-37.1 to 21.8)
		Kidney dysfunction	553.4 (411.9 to 747.8)	513.4 (358.1 to 793.4)	615.1 (454.2 to 841.3)	476.6 (362.3 to 632.4)	446.1 (331.5 to 628.8)	509.7 (377.4 to 686.8)	-13.9 (-36.1 to 15.9)	-13.1 (-38.4 to 20.8)	-17.1 (-39.1 to 15.4)

Location	Measure	Risk factor	Attributed age-standardized rate (per 100,000)						% Change (1990 to 2019)		
			1990			2019					
			Both	Female	Male	Both	Female	Male	Both	Female	Male
	YLDs	Lead exposure	5.9 (3.7 to 8.7)	5.9 (3.5 to 8.9)	5.8 (3.7 to 8.6)	9.1 (5.6 to 13.6)	8.2 (5 to 12.6)	10.1 (6.3 to 15.2)	54.9 (36.8 to 74.4)	39.4 (19 to 66.5)	72.5 (48.1 to 100.7)
		Diet high in sodium	1 (0.2 to 3.9)	0.9 (0.2 to 3.6)	1.1 (0.1 to 4.6)	1.6 (0.3 to 6.2)	1.4 (0.3 to 5.3)	1.9 (0.2 to 7.5)	57.6 (0.2 to 137.4)	45.5 (-25.6 to 161.4)	67.1 (8.6 to 211.3)
		High fasting plasma glucose	17 (12.1 to 23.2)	18.2 (13 to 24.8)	15.6 (10.6 to 21.8)	26.9 (18.6 to 37.6)	27.1 (18.6 to 38.6)	26.7 (17.8 to 37.8)	58.7 (40.9 to 78.6)	49.4 (26.2 to 79.6)	71.2 (49.2 to 96.8)
		High body-mass index	11.1 (4.2 to 21.7)	15.8 (6.4 to 29.9)	6.1 (1.7 to 12.9)	28 (12.7 to 47.2)	36.4 (16.8 to 63.4)	19.6 (8.7 to 34.8)	153.1 (93.9 to 294.8)	130.9 (70.4 to 262.9)	220.9 (124.7 to 529)
		High systolic blood pressure	36.5 (25 to 49.6)	43 (29.2 to 58.6)	28.5 (19.3 to 39.5)	58.2 (39.8 to 80.7)	65.8 (42.8 to 92.1)	50.5 (34 to 71.5)	59.6 (38.4 to 84.1)	53.1 (23.9 to 90.8)	77.4 (50 to 106.6)
		Kidney dysfunction	87.8 (64 to 115.5)	105.6 (77 to 140.5)	67.5 (47.2 to 90.2)	135 (97.7 to 178)	158.2 (112.8 to 212.8)	111.5 (80 to 152.6)	53.7 (39.6 to 71)	49.8 (33 to 76.2)	65.2 (48.1 to 83.9)

Data in parentheses are 95% Uncertainty Intervals (95% UIs)
